# Persistence of the *ABCC6* genes and the emergence of the bony skeleton in vertebrates

**DOI:** 10.1038/s41598-018-24370-7

**Published:** 2018-04-16

**Authors:** Bruna Parreira, João C. R. Cardoso, Rita Costa, Ana Rita Couto, Jácome Bruges-Armas, Deborah M. Power

**Affiliations:** 1 Serviço Especializado de Epidemiologia e Biologia Molecular (SEEBMO), Hospital de Santo Espírito da Ilha Terceira, Azores, Portugal; 2 Centre of Marine Sciences (CCMAR), Universidade do Algarve, Campus de Gambelas, 8005-139 Faro, Portugal; 3 CEDOC – Chronic Diseases Research Center, Universidade Nova de Lisboa, Lisbon, Portugal; 4 Key Laboratory of Exploration and Utilization of Aquatic Genetic Resources, Ministry of Education, Shanghai Ocean University, Shanghai, China

## Abstract

The ATP-binding cassette transporter 6 (*ABCC6*) gene encodes a cellular transmembrane protein transporter (MRP6) that is involved in the regulation of tissue calcification in mammals. Mutations in *ABCC6* are associated with human ectopic calcification disorders. To gain insight into its evolution and involvement in tissue calcification we conducted a comparative analysis of the *ABCC6* gene and the related gene *ABCC1* from invertebrates to vertebrates where a bony endoskeleton first evolved. Taking into consideration the role of *ABCC6* in ectopic calcification of human skin we analysed the involvement of both genes in the regeneration of scales, mineralized structures that develop in fish skin. The *ABCC6* gene was only found in bony vertebrate genomes and was absent from Elasmobranchs, Agnatha and from invertebrates. In teleost fish the *abcc6* gene duplicated but the two genes persisted only in some teleost genomes. Six disease causing amino acid mutations in human MRP6 are a normal feature of abcc6 in fish, suggesting they do not have a deleterious effect on the protein. After scale removal the *abcc6* (5 and 10 days) and *abcc1* (10 days) gene expression was up-regulated relative to the intact control skin and this coincided with a time of intense scale mineralization.

## Introduction

The ATP-Binding Cassette (ABC) transporters are a large and ancient family of active transporter proteins present in a broad spectrum of organisms from bacteria to vertebrates^1,2^. These transporters promote the translocation across the cell membrane and organelle compartments of various substrates, from ions and small molecules to large polymers^3^. ABC mediated transport is suggested to occur via ATP hydrolysis^4,5^, although the exact mechanism still remains poorly understood^6–10^. The ABC transporters are characterized by four core domains, two transmembrane domains (TMD) and two highly conserved nucleotide binding domains (NBD), that are part of its functional complex. Half transporters with a single TMD and single NBD also exist and are functional if they assemble as homodimers or heterodimers^1,11^. The NBDs, are the motor domains of ABC transporters and contain a phosphate binding loop (Walker A), a magnesium binding site (Walker B), a switch region with a histidine loop, a Q-motif and a conserved signature motif (LSGGQ)^12,13^. In human an estimated 49 ABC members exist and they are divided into seven subgroups (ABCA to ABCG), and some are responsible for, or are involved in human diseases and cancer treatment resistance, as they cause extrusion of anticancer drugs from tumor cells^14,15^.

The ABC subfamily C (ABCC) members are large proteins (1325–1582 amino acids in length) encoded by 13 genes. Nine genes (*ABCC 1–6* and *10–12*) encode multidrug resistance-associated proteins (MRP), three genes (*ABCC 7–*9) encode ion channel proteins or regulators of potassium channels and one pseudogene (*ABCC13*)^2,16,17]^. Evolutionary analysis reveals that with the exception of the pseudogene, two major ABCC subfamily clusters exist in humans: one that contains *ABCC1*, 2, *3*, *6*, *8* and *9* and another with *ABCC 4*, *5*, *7*, *10*, *11* and 12^2^.

The *ABCC6* gene encodes the multidrug resistance protein 6 (MRP6), which arose in genomes by tandem gene duplication, a process that also produced the *ABCC1* (MRP1) gene^11^, which in humans prevents soft tissue calcification^18^. Calcification is an essential physiological mechanism in the process of skeletal tissue formation, which is tightly controlled and restricted to specific body regions. Although the calcification process is incompletely understood, inhibitors and promoters are thought to act synergistically to maintain calcification of bone and dental tissue^19^. Calcification disorders occur when calcium is abnormally deposited in soft tissues causing ectopic calcification^20^. Biallelic inactivating mutations of the *ABCC6* gene lead in most cases to Pseudoxanthoma elasticum (PXE; OMIM#264800), a rare autosomal recessive genetic disorder characterized by aberrant mineralization of soft connective tissue (eyes, cardiovascular system and skin)^21–24^. *ABCC6* mutations are also linked to some cases of Generalized Arterial Calcification of Infancy (GACI; OMIM #614473)^25^, a calcification disorder that affects the circulatory system^26^ and is associated with mutations of the Ectonucleotide Pyrophosphatase/Phosphodiesterase 1 (*ENPP1*) gene^27^. Manifestations of PXE are associated with modified plasma inorganic pyrophosphate (PPi) levels and it has been suggested that PPi may be the factor that prevents mineralization in PXE patients, since it is a key regulator of ectopic mineralization inhibiting hydroxyapatite crystal growth^28–31^. Nonetheless, the precise function of *ABCC6* in the calcification process and pathologies is still controversial and may involve other disease mediators^32^.

Homologues of the human *ABCC6* gene also occur in other vertebrates but their role in tissue calcification remains poorly understood. In recent years, research on ABC transporters in fish has advanced, facilitated by the sequencing of fish genomes^33,34^. Teleost fish are the largest and most successful group of extant vertebrates and they are used as models to study bone mineralization, as they share with other vertebrates many of the basic features of cartilage (chondrogenesis) and bone (osteogenesis) formation^35–42]^. So far, zebrafish is the only teleost fish in which the *abcc6* gene has been studied and associated with tissue mineralization^38,39^. In zebrafish, two *abcc6* genes were identified and zebrafish *abcc6a* mutants develop ectopic calcification generally around the perichondral bone in the craniofacial and axial skeleton^39^. In contrast to humans where the *ABCC6* gene is mostly detected in the liver, in zebrafish expression of *abcc6a* was strongly linked with tissues actively involved in mineralization suggesting that in fish *abcc6a* functions locally and that ligand transport is not liver derived^39^. Furthermore, *enpp1* mutants exhibited ectopic calcification in soft tissues, including the skin, cartilage elements, heart, intracranial space and notochord sheet^35^.

The *ABCC6* gene is located on human chromosome 16 between its two almost identical pseudogenes, *ABCC6P1* and *ABCC6P2* which arose by segmental duplications in primate genomes and are proposed to regulate expression of the functional gene^43–45^. In mammals, *ABCC6* is highly abundant in the basolateral membrane of hepatocytes and to a lesser extent in the proximal kidney tubules^46,47^. Very low to undetectable levels of *ABCC6* expression occur in brain, retina, vessel walls and the skin (a tissue affected by PXE)^21,46,48,49^. In order to better understand the potential association of *abcc6* genes with vertebrate calcification and tissue mineralization we characterized the evolution of the orthologue of the human *ABCC6* gene in other vertebrates and in invertebrates and the expression of its transcripts during scale regeneration in fish. The involvement of *abcc6* in scale formation/regeneration, a common mineralization process in fish, has never been addressed. Scales are important skin appendages imbricated in the dermis and are a reservoir of minerals, provide protection and assist hydrodynamics. When scales are lost, they regenerate and the formation of a new scale and the scale pocket is initiated almost immediately after damage^50^. The potential involvement of *abcc6* and the sequence related *abcc1* in the process of scale regeneration was analyzed in the sea bream by measuring transcript abundance at several time points after damage was inflicted.

## Material and Methods

### Database searches and sequence retrieval

The genomes of several vertebrates and invertebrates (with publicly available data) were analyzed for orthologues of the human *ABCC6* gene (ENSG00000091262) and the sequence related family members, *ABCC1* (ENSG00000103222) and *ABCC3* (ENSG00000108846). A total of 30 vertebrate genomes representatives of different classes (see Supplementary Table 1), were searched using the BLAST algorithm or database annotations for orthologues of the human *ABCC6*, *ABCC1* and *ABCC3* genes. The majority of the data accessed was deposited in the Ensembl database and the most recent assembly update was used (http://ensembl.org/, 2018). The genomes analyzed included 8 mammals (chimpanzee, *Pan troglodytes*; gorilla, *Gorilla gorilla*; mouse, *Mus musculus*; dog, *Canis lupus familiaris*; armadillo, *Dasypus novemcinctus*; cow, *Bos taurus*; opossum, *Monodelphis domestica*; platypus, *Ornithorhynchus anatinus*); a bird, the chicken (*Gallus gallus*); a reptile, the green anole lizard (*Anolis carolinensis*); an amphibian, Xenopus (*Xenopus tropicalis*); a lobe-finned fish, the coelacanth (*Latimeria chalumnae*); 9 teleosts (Actinopterigii) and 1 non-teleost ray finned fish, the spotted gar (*Lepisosteus oculatus*) and two agnathans (the sea lamprey, *Petromyzon marinus* and the arctic lamprey, *Lethenteron camtschaticum*, GCA_000466285.1, available from NCBI, 2018). The teleost genomes explored were: tilapia, *Oreochromis niloticus*; platyfish, *Xiphophorus maculates*; cod, *Gadus morhua*; stickleback, *Gasterosteus aculeatus*; medaka, *Oryzias latipes*; tetraodon, *Tetraodon nigroviridis*; blind cave fish, *Astyanax mexicanus*; zebrafish, *Danio rerio*, and the sea bass, *Dicentrarchus labrax* (http://seabass.mpipz.de/)^51^. The genomes of two cartilaginous fish which diverged prior to the emergence of bony vertebrates (Class Chondrichthyes), the elephant shark *(Callorhinchus milii*, http://esharkgenome.imcb.a-star.edu.sg/, 2018) and whale shark (*Rhincodon typus*, GCF_001642345.1, available from NCBI, 2018), were also explored as well as the transcriptomes of two other cartilaginous fishes, the small spotted catshark (*Scyliorhinus canicula*) and the little skate (*Leucoraja erinácea*), available from Skatebase (http://skatebase.org/, 2018). The short scaffolds of the little skate genome limited gene identification.

To trace the likely evolutionary origin of the *ABCC6* gene, the genomes of several invertebrates were also analyzed by accessing the Ensembl Metazoa database (http://metazoa.ensembl.org/index.html, 2018). Invertebrate genomes analyzed (n = 12, Supplementary Table 2) included the deuterostomes basal to the vertebrates, the urochordate Ciona (*Ciona intestinalis* and *Ciona savigny*), the cephalochordate Amphioxus (*Branchiostoma floridae*) and the hemichordate, the Sea urchin (*Strongylocentrotus purpuratus*) and also several protostome genomes: the Owl limpet (*Lottia gigantea*), the Leech (*Helobdella robusta*), the Fruit fly (*Drosophila melanogaster)*, the Honey bee (*Apis mellifera*), the Flour beetle (*Tribolium castaneum)*, the Mosquito (*Anopheles gambiae*), the crustacean Daphnia (*Daphnia pulex*) and a roundworm, the Nematode (*Caenorhabditis elegans)*. The amino acid sequences of putative candidate genes were retrieved by selecting those with the lowest e-value similarity score (e < −20) and their similarity to the query protein and putative identity was confirmed by searching against the NCBI human protein database (http://blast.ncbi.nlm.nih.gov/Blast.cgi, 2018), using the Blastp algorithm.

### Multiple sequence alignment and phylogenetic analysis

The full-length human and vertebrate MRP6 deduced proteins were aligned using ClustalW and the amino acids that are normally changed by the principal nucleotide mutations responsible for the manifestation of PXE (http://www.hgmd.cf.ac.uk/; accessed May 2016) were mapped across different species. The deduced nucleotide sequence of the full-length human *ABCC6* gene (ENSG00000091262) and the two pseudogenes *ABCC6P1* (ENSG00000256340) and *ABCC6P2* (ENSG00000255277) were also aligned to identify differences between the sequences. The GeneDoc 2.7 software was used to calculate the percentage of sequence identity/similarity between the homologue genes of the different species.

Phylogenic trees were constructed using the deduced amino acid sequence alignment of the retrieved *ABCC6*, *ABCC1* and *ABCC*3 genes (see Supplementary Tables 1 and 2). The sequences of other human ABCC transporters: ABCC 2, 4, 5, 8, 9, 10, 11, 12 and CFTR were also included. Sequences were aligned in the AliView platform^52^ using MUSCLE^53^ and manually edited to remove sequence gaps and poorly aligned regions. The final edited alignment was used as the input for the construction of phylogenetic tree by: Bayesian inference (BI) in MrBayes^54^ and Maximum Likelihood (ML) implemented in PhyML 3.0 software (http://www.atgc-montpellier.fr/phyml/)^55^, using the SMS automatic model selection^56^, for protein evolutionary analysis according to the AIC (Akaike Information Criterion).

BI analysis was performed using an LG substitution model (Aamodel = LG) and 1.000.000 generation sampling and probability values to support tree branching. The ML tree was built with an LG+I+G+F substitution model with the following parameters: gamma shape- 4 rate categories (G=0.972) and proportion of invariable sites (I=0.035). The statistical support for tree branching was assessed using 100 bootstrap replicates. Both, BI and ML phylogenetic trees were displayed and annotated in the FigTree program and rooted with the protostome and other human ABCC branches.

### Gene structure and gene synteny

The gene structure of human *ABCC*6 and its two pseudogenes (*ABCC6P*1 and *ABCC6P*2) and the spotted gar *abcc6* gene were retrieved from ENSEMBL and compared. The gene environment of the vertebrate *ABCC6* genes was characterized using as a template the human gene annotation in ENSEMBL (GRCh38, same assembly as in UCSC genome browser). Gene orthologues were identified in the mammals, gorilla (*Gorilla gorilla*), opossum *(Monodelphis domestica)* and platypus *(Ornithorhynchus anatinus)*, in the bird, chicken (*Gallus gallus)*, in the ray-finned fishes, spotted gar (*Lepisosteus oculatus)*, zebrafish (*Danio rerio*), Tetraodon (*Tetraodon nigroviridis*), medaka (*Oryzias latipes*) and in two cartilaginous fishes, the elephant shark (*Callorhinchus milii)* and whale shark (*Rhincodon typus*) using genome annotations, complemented with sequence homology searches.

### Transcriptome database searches

To increase knowledge about the physiological importance of the *ABCC* genes, searches on vertebrate transcriptome data were also performed. The distribution of non-mammalian *ABCC6* transcripts was characterized using transcriptome and EST (Expressed sequence tag) databases for the human, bird, reptile, amphibian and teleosts and a digital expression map was developed to identify overlapping tissue expression of the *ABCC6* gene. Searches were performed in the lineage-specific NCBI EST databases for human (taxid: 9606); birds (taxid: 8782); reptiles (taxid:8504); amphibians (taxid:8292) and teleost fishes (taxid:32443) using the human, chicken, anole lizard, Xenopus and zebrafish *abcc6* sequences, respectively. The identity of the retrieved sequences was confirmed by homology with the human orthologues. Searches were also performed in other gene expression databases: Geisha (http://geisha.arizona.edu/geisha/), Xenbase (http://www.xenbase.org/entry/), Expression Atlas Database (https://www.ebi.ac.uk/gxa/home), GeneCards (http://www.genecards.org/), Ensembl (http://www.ensembl.org/index.html) and complemented with published data^39^. In addition, available sea bass scale and skin transcriptomes were also analysed for *abcc6* and *abcc1* transcripts^57^.

### Polymerase chain reaction (PCR) and quantitative-PCR (qPCR)

We further explored the potential physiological role of *abcc* transcripts in fish skin during scale regeneration. *Abcc1* and *abcc6* genes were studied in the regenerating skin/scale of sea bream (*Sparus aurata*) over 28 days as previously described^58,59^. The experiments were carried out following international and national guidelines for animal care and experimentation, under a “Group-I” license from the Portuguese Government Central Veterinary service to CCMAR and conducted by a certified investigator (DMP).

Briefly, adult sea bream of the same age class (1 year), were maintained in 500 L replicate tanks (n = 8) in an open seawater circuit and supplied with a constant flow of aerated seawater at 18 °C + 1 °C. At time zero all fish in the 8 experimental tanks were anaesthetized in 2-phenoxyethanol (0.01%), rinsed in seawater and the scales were removed from the left flank of the body by gently stroking the skin with forceps to minimize damage to the dermis. Samples (N = 8/time point) of intact skin (untouched right hand flank) and damaged skin (left hand flank) were collected at 0, 5, 10 and 28 days after scale removal. At each sample point fish were removed from 2 tanks and killed with an overdose of 2-phenoxyethanol (0.1% in seawater) and then rinsed in clean seawater. The skin was then dissected from below the dorsal fin directly above the lateral line using a scalpel and forceps and removing the muscle before snap freezing the sample in liquid nitrogen. In this way, the same fish provided control and regenerating skin samples that were directly compared. No mortality or skin lesions occurred in any of the fish.

Specific primers for sea bream *abcc6* (forward, Sb_abcc6fwd ttagagacaagacccgcat and reverse, Sb_abcc6rev tggcaaaggtgtggatgaag) and *abcc1* (forward, Sb_*abcc1fwd* tatgagtcacctcaacaaagc and reverse, Sb_*abcc1rev* tccgttcatactggattacca) were designed using sequences retrieved from the CCMAR sea bream transcriptome database^60^. Preliminary analysis of tissue expression was established by PCR of *abcc6* and *abcc1* using sea bream cDNA from bone, skin, larva and scale. The thermocycle was as follows: 95 ºC, 3 min; (95 ºC 30 sec, 60 ºC 30 sec, 72 ºC 30 sec) cycled 40 times and a final extension at 72 ºC for 5 min. The amplified PCR products were sequenced to confirm their identity before testing gene expression during regeneration.

To investigate the potential involvement of *abcc6* and *abcc1* in sea bream skin regeneration a qPCR experiment was run using cDNA from both intact and damaged skin of 6 individuals for each time point. The qPCR was performed for a 10 μl final reaction volume, in duplicate, using 1× SsoFast-Evagreen Supermix (Biorad) and 300 nM of forward and reverse primers. The PCR reaction was performed using a CFX Connect™ Real-Time PCR Detection System (Bio-Rad) and the following program: 30s at 95 °C, 45 cycles of 5 s at 95 °C and 15 s at 60 °C. Negative controls were also run and included a no-template control (NTC). A final melt-curve was carried out between 60 °C and 95 °C and produced a single product dissociation curve for each gene. Relative expression of the analysed genes was compared using the delta Ct values normalized with the geometric mean of the reference genes *rps18* (forward primer agggtgttggcagacgttac and reverse primer cttctgcctgttgaggaacc) and β*-actin* (forward primer ccctgccccacgccatcc and reverse primer tctcggctgtggtggtgaagg). The reference gene expression did not vary significantly between any of the samples. Results are presented as 1/delta Ct.

### Statistical analysis

The relative expression data from qPCR was evaluated by two-way ANOVA with a Tukey’s multiple comparisons test using the software GraphPad Prism, version 7.0a for Mac OS X (GraphPad Software, La Jolla California USA, www.graphpad.com). The significance cut-off was set at p < 0.05 and results are presented as mean ± standard error of the mean (sem).

## Results

### *ABCC6* members in vertebrates

**Figure 1:**
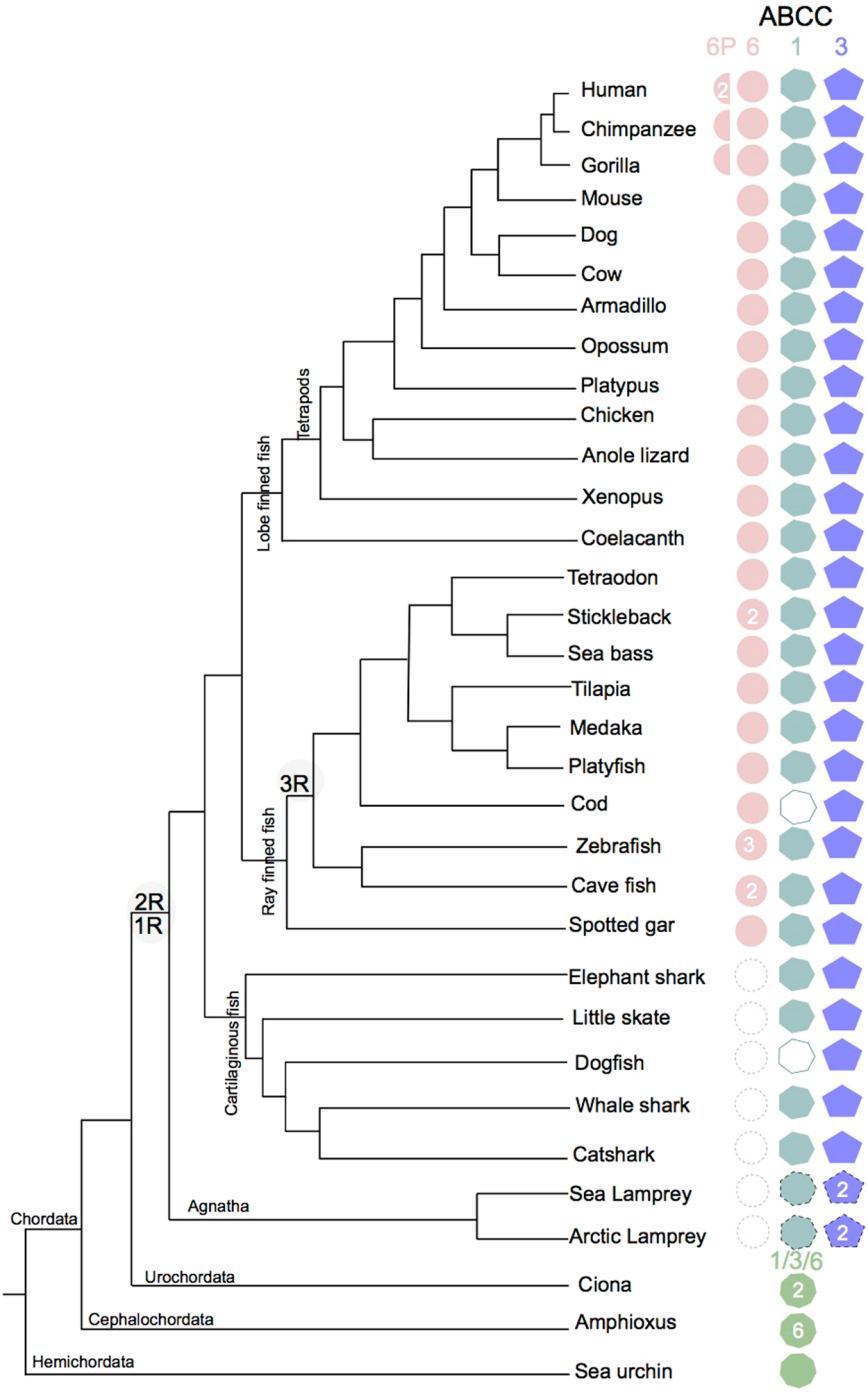
Dendrogram representing the *ABCC6*, *ABCC1* and *ABCC3* genes identified in deuterostomes. Gene family members are represented by different colours and shapes and the number of genes found is indicated. Open symbols represent genes that were not identified but are likely to exist and half circles the *ABCC6* pseudogenes. Dashed symbols indicate uncertain identity and dashed circles represent *abcc6* absence. The figure was drawn considering the relative evolutionary relationship between the different species represented using information from^77–80^. The two major genome duplication events (1R and 2R) that occurred early in the vertebrate radiation and the teleost specific genome duplication event (3R) are indicated. Gene accession numbers are available from Supplementary Tables 1 and 2.

Orthologues of the human *ABCC6* gene were identified in all the teleost and tetrapod genomes analyzed (Fig. 1 and Supplementary Table 1). Searches in the genome of the elephant shark (*Callorhinchus milii*) and whale shark (*Rhincodon typus*) and in transcriptome data from two other cartilaginous fish species, the small spotted catshark (*Scyliorhinus canicula*) and Little skate (*Leucoraja erinácea*), failed to retrieve putative *abcc6* genes or transcripts. In the Agnatha, sea lamprey (*Petromyzon marinus*) and arctic lamprey (*Lethenteron camtschaticum*) as previously reported, *abcc6-like* genes were absent^61^ (Fig. 1 and Supplementary Table 1). In some teleost species such as the stickleback (*Gasterosteus aculeatus*), blind cave fish (*Astyanax mexicanus*) and zebrafish (*Danio rerio*) more than one *abcc6* gene was identified. In stickleback and blind cave fish two *abcc6* genes (*abcc6a* and *abcc6b*) were found and in zebrafish three seem to exist (*abcc6a*, *abcc6b1* and *abcc6b2*) (Fig. 1).

No putative *abcc6-like* pseudogenes were identified in the vertebrate genomes analysed with the exception of the primates. Searches in the chimpanzee (*Pan troglodytes*) and gorilla (*Gorilla gorilla*) identified orthologues of the human *ABCC6* full-length gene as well as other genome regions which may encode putative *ABCC6* pseudogenes. In the chimpanzee, *ABCC6-like* putative genome regions were found in Scaffold KV421237.1 (ENSPTRG00000048009) and Scaffold AACZ04003842.1 and in the gorilla genome a region up-stream (2.3 Mb) of the predicted *ABCC6* gene (ENSGGOG00000009623) on chromosome 16 was also identified.

*ABCC6*-related family members, the *ABCC1* and *ABCC*3 genes, were also retrieved from databases for evolutionary comparisons because, a) they are similar to *ABCC6* and b) *ABCC1* in the human genome is localized in close proximity to the *ABCC6* locus. Orthologues of the human *ABCC1* and *ABCC*3 genes were identified in almost all vertebrates with the exception of *abcc1* in cod (*Gadus morhua*) (Fig. 1), probably because its genome assembly is incomplete. In sea lamprey a single *abcc1-like* and two *abcc3-like* transcripts (KM232930.1 and KM232931.1) have previously been described^61^, and the locus for the first transcript was found in the genome assembly (ENSPMAG00000000892) and the orthologues were also retrieved from the arctic lamprey genome (*abcc1-like*, KE994284.1 and two *abcc3-like*, KME993868.1 and KE993993.1). Searches in the urochordate, cephalochordate, hemichordate and protostome genomes were also carried out to characterize the origin of the *abcc*6, 1 and 3 gene family members and putative *abcc*-like genes were retrieved and many species seem to possess multiple copies (Fig. 1 and Supplementary Table 2).

### Sequence conservation of the amino acids altered in PXE disease

Multiple sequence alignment of the deduced amino acid sequence of the MRP6 proteins revealed a relatively high conservation overall, and several highly-conserved regions were identified (Supplementary Fig. 1). Comparison of the deduced full-length MRP6 mature protein sequence from human with the orthologue in the chicken indicated that they shared 52% amino acid identity. The MRP6 proteins in human and the coelacanth were 48% identical and 42–47% identical with the protein orthologues in the ray-finned fishes, stickleback, sea bass, tetraodon, tilapia, medaka, platyfish, cod, zebrafish, blind cave fish and spotted gar (Supplementary Table 3).

One hundred and thirty-eight missense/nonsense mutations, associated exclusively with PXE disease only, were selected from the publicly available list of the Human Gene Mutation Database (HGMD) and the positions of the altered amino acids were mapped in the MRP6 sequence alignment and compared across different vertebrates (Supplementary Fig. 1). Mutations have been identified throughout the protein, however the “hot spots” associated with human PXE are located within the cytoplasmic region of human MRP6 and only one was found within a TM domain (TM12). Comparisons with the vertebrate homologue amino acid positions revealed that the positions mapped in the alignment where the human mutations occur are in general well conserved across other vertebrates. More than 50 amino acid positions are totally conserved (Supplementary Fig. 1), suggesting that they were under high conservative selection pressure during the vertebrate radiation.

**Figure 2:**
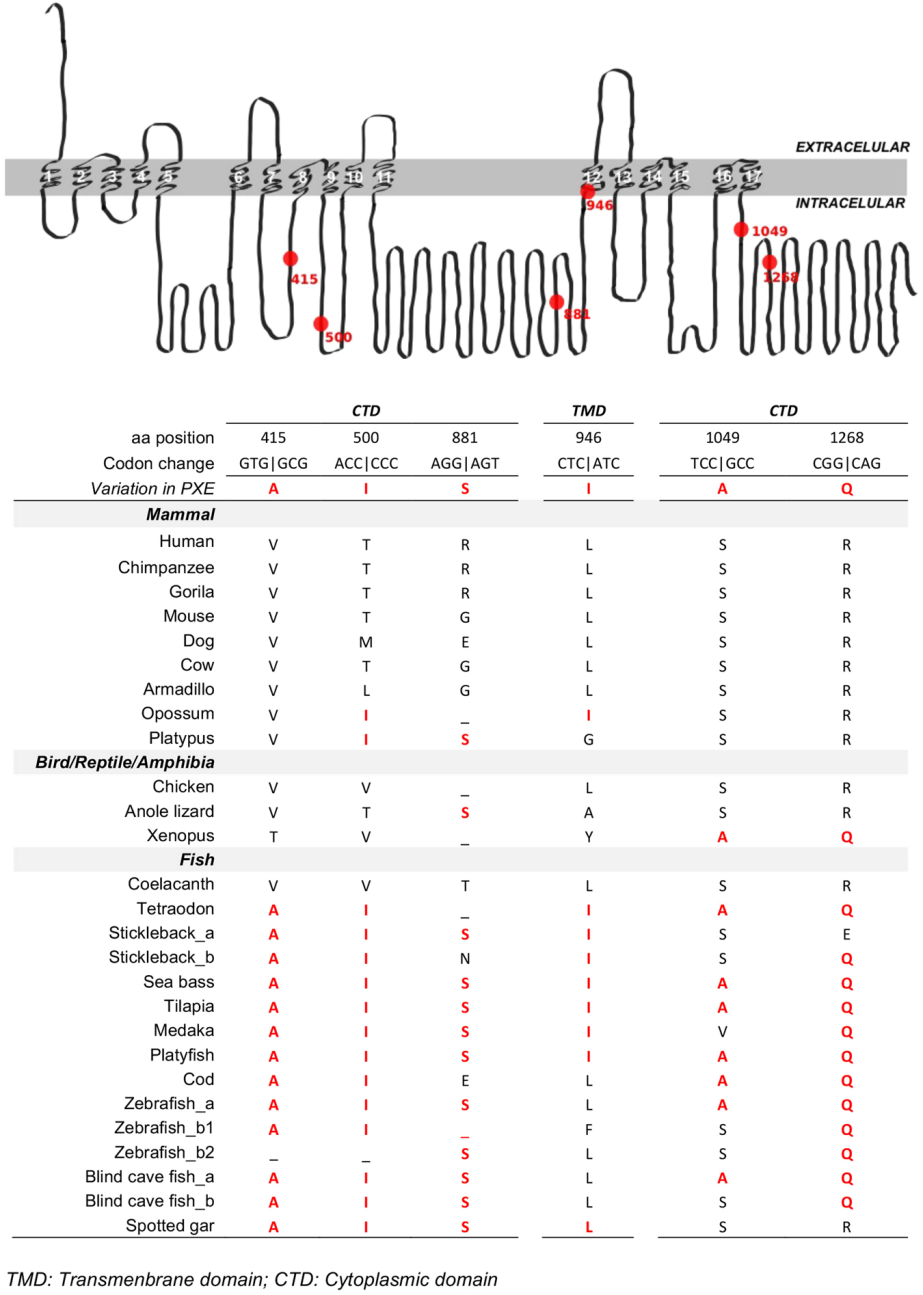
The predicted structure of human MRP6 (**A**) and comparison of the amino acids that cause PXE in humans with those present in other species (**B**). (**A**) The 9 amino acid positions found in human PXE variants are colored in red and transmembrane domains are numbered (1 to 17). (**B**) The amino acids that are common between the human PXE diseases and conserved in other vertebrates are coloured in red. TMD: Transmembrane domain, CTD: Cytoplasmic domain, EXT: Extracellular  domain.

Interestingly, in some species, especially within the fishes, there are positions in which the amino acids correspond to mutated amino acids in human MRP6. The amino acid positions, common between the fish and the mutated human MRP6 protein, map to the cytoplasmic region (415; 500; 881; 946; 1049 and 1268) and the transmembrane region (946) and are represented in Fig. 2. Comparisons of *ABCC6* and *ABCC1* revealed that the location of the *ABCC6* point mutations linked with PXE are generally associated with amino acids that are highly conserved across the related family member, suggesting that they may be important in the maintenance of ABCC family function (Supplementary Fig. 1).

### Phylogeny of *ABCC6*

**Figure 3:**
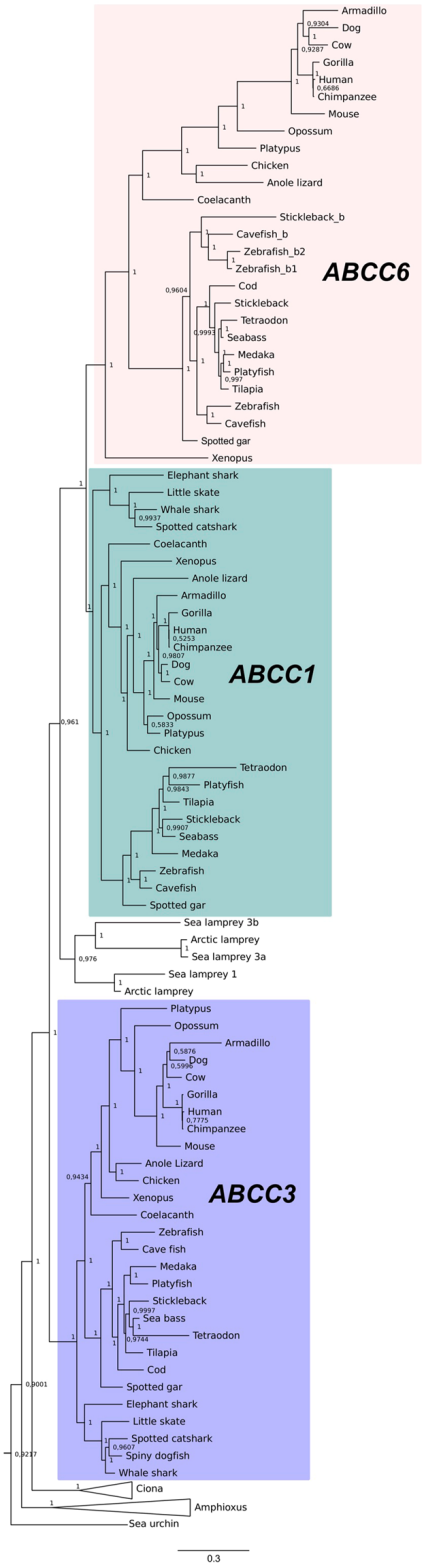
Phylogenetic tree of the ABCC6 and sequence related ABCC1 and ABCC3. The phylogenetic tree was constructed using Bayesian inference (BI) built in MrBayes 3.2 and branch support values (posterior probability values) are shown. To facilitate interpretation, the three major vertebrate clades are boxed with different colours and the Ciona and Amphioxus branches are collapsed. The tree was rooted with the protostome and other human ABCC family members. The original tree is available in Supplementary Fig. 1 and the accession numbers of the sequences are given in Supplementary Tables 1 and 2.

Phylogenetic analysis was performed based on the alignment of the deduced vertebrate MRP6, MRP1 and MRP3 mature proteins and the putative orthologues in deuterostomes basal to vertebrates and protostomes. Both, BI (Fig. 3 and Supplementary Fig. 2) and ML (Supplementary Fig. 3) trees displayed similar topologies and showed that *ABCC6* and the two other family members cluster in independent branches (with strong bootstrap support) and that they shared common ancestry prior to the vertebrate radiation (Fig. 3 and Supplementary Figs 2 and 3).

Both trees also suggest that the vertebrate *ABCC3* members diverged earlier than the *ABCC1* and *ABCC6* genes that arose from a subsequent gene duplication event. Several gene duplications also occurred in invertebrates and also in some vertebrates, such as the teleosts. Based on the sequence clustering in the phylogenetic trees, the two *abcc6* gene copies (*abcc6a* and *abcc6b*) in the stickleback, zebrafish and the blind cave fish seem to be the result of the teleost specific genome duplication and that the existence of a third *abcc6* gene in the zebrafish may be the result of a duplication of the *abcc6b* paralogues (*abcc6b1* and *abcc6b2*). The Xenopus *abcc6* gene does not cluster as expected based on the consensus for species evolution, suggesting that *abcc6* in this species suffered distinct selective pressures or that there may be errors in the genome assembly and predicted gene.

The clustering of sequences in the tree also confirmed that orthologues of the human *ABCC6* genes are absent in cartilaginous fishes, but *abcc1* and *abcc3* genes are present. The position in the tree of the three lamprey sequences differed according to the method used and while in the BI tree they radiate basal to the vertebrate *ABCC1/ABCC6* cluster. In the ML tree *ABCC1/ABCC6* are basal to the three major clades (Fig. 3, Supplementary Fig. 3) and so gene identity based upon phylogeny remains unclear. The invertebrate branch contained several *abcc*-like genes that do not group with any of the three vertebrate ABCC clusters. Inclusion in the phylogenetic tree of the other human ABCC transporters suggested that the identified urochordate, cephalochordate and hemichordate sequences shared common ancestry with vertebrate *ABCC1*, *3* and *6* genes (Fig. 3, Supplementary Figs 2 and 3).

### Gene structure

**Figure 4:**
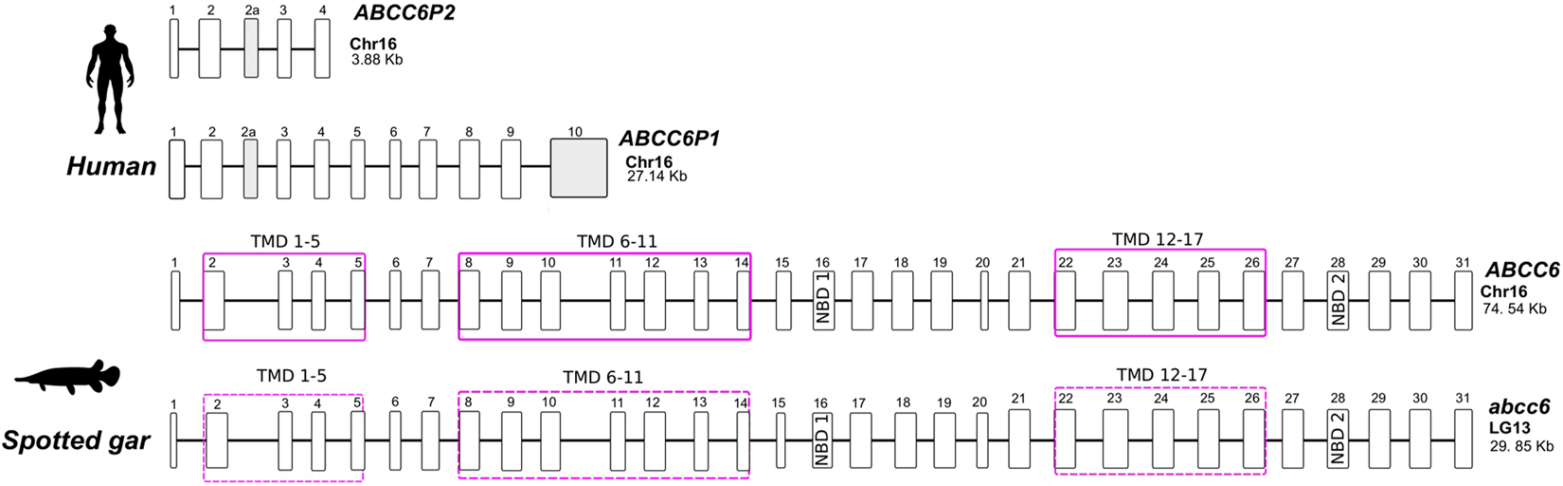
Comparison of the gene organization of the *ABCC6* gene in human and a ray finned fish, the spotted gar. Gene sizes are indicated, exons (E) are numbered and represented by boxes and the solid black line denotes the introns. The predicted transmembrane domains (TMD) in spotted gar are dashed and were predicted using the TMHMM Server v. 2.0 (http://www.cbs.dtu.dk/services/TMHMM/) and the sequence alignments. Exons are drawn to scale but the introns are not to scale. The E2’ exon is exclusive to the pseudogenes, *ABCC6P1* and *P2*, and is absent from the full-length genes of the human and spotted gar. The size of exons and introns are listed in Supplementary Table 5. NDB: Nucleotide binding domain.

The gene structure of the human *ABCC6* genes (including the two pseudogenes *ABCC6P1* and *ABCC6P2*) was compared with the fish, spotted gar *abcc6* gene. In human, the full *ABCC6* gene structure is composed of 31 exons and spans 74.56 kb in chromosome 16. The two pseudogenes are much smaller and map in close proximity to the full-length *ABCC6* gene. *ABCC6P1* is composed of 11 exons and *ABCC6P2* of 5 exons which span 27.14 Kb and 3.88 Kb, respectively (Fig. 4). Sequence alignment of the nucleotide sequences of human *ABCC6* and its pseudogenes revealed that they are highly identical and that the pseudogenes share ≈95% similarity with the functional gene (data not shown). An extra exon (E2’), present in the two pseudogenes, is not transcribed in the full-length *ABCC6* gene transcript (Fig. 4, Supplementary Table 4). Comparisons of the transcript sequences between the *ABCC6* gene and *ABCC6P1* revealed that exons E1 to E10 in *ABCC6* are identical to *ABCC6P1* and only exon E11 in *ABCC6P1* shows no homology with the *ABCC6* gene. For *ABCC6P2* the overlapping exons are identical. Comparison of the predicted gene structure of the human and spotted gar (a slowly evolving fish genome, that has not suffered tetraploidization, 3R) revealed that they share the same exon number, possess an identical gene structure and share the same protein motifs with the human *ABCC6* gene (Fig. 4, Supplementary Table 4). Overall, this suggests that pressure was maintained to preserve gene structure during evolution.

### Gene linkage across vertebrates

**Figure 5:**
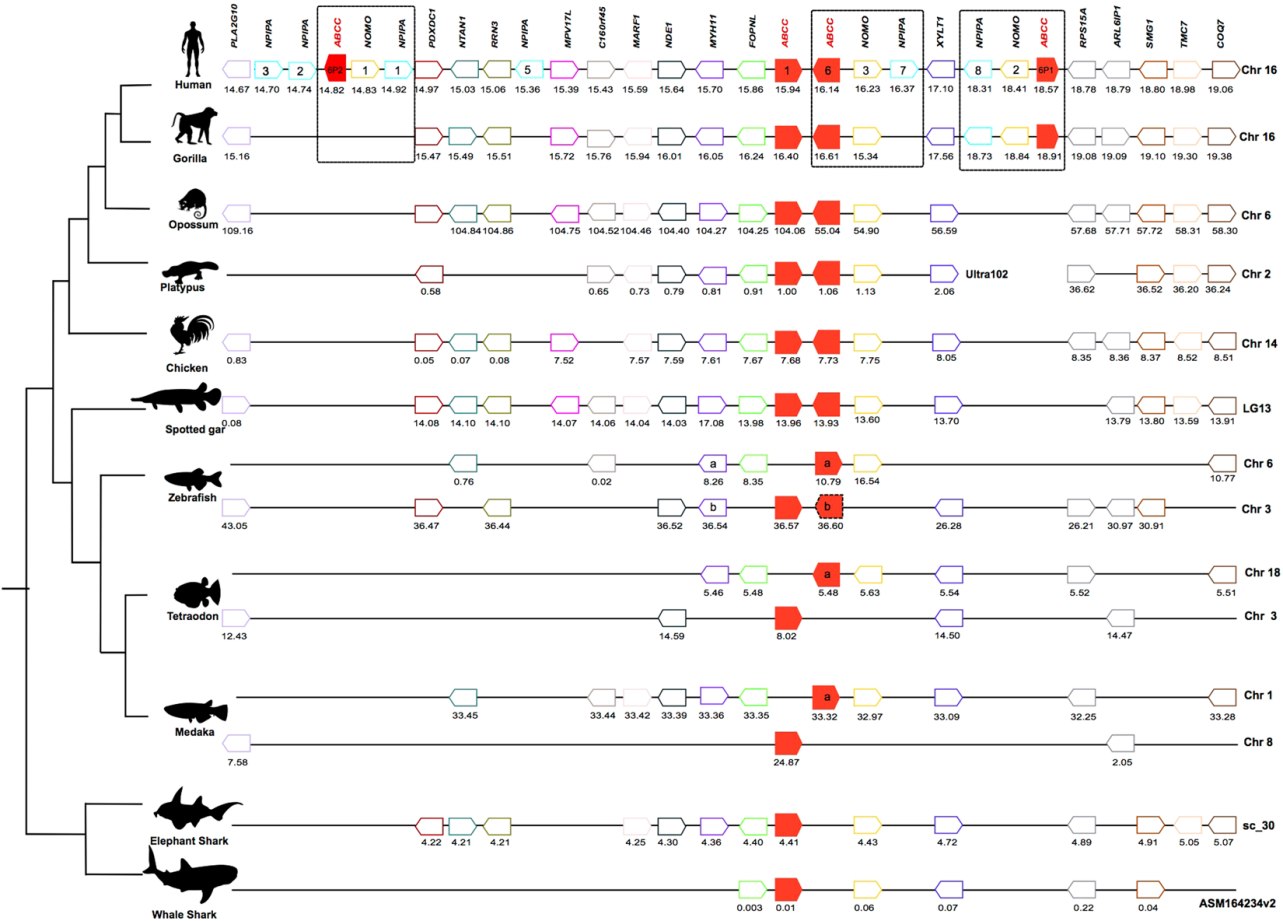
Gene synteny analysis of the *ABCC6* gene environment across vertebrates. The *ABCC6* genes and pseudogenes (*ABCC6P1* and *ABCC6P2*) and the sequence related *ABCC1* gene are represented in red. The neighbouring genes are represented by other coloured boxes and the arrowheads represent transcript orientation predicted in the genome of each species. Members of the same family are indicated in the same colour and the members of families with multiple genes are indicated inside the arrowhead box. The genome fragments analysed and position of each gene is indicated below (Mb). The genome regions of the *ABCC6* gene and the neighbouring genes that are likely to have duplicated and originated the primate *ABCC6* pseudogenes in the human and gorilla genomes are boxed. The zebrafish *abcc6b* gene is dashed because two genes have been predicted (*abcc6b1* and *abcc6b2*).

The gene environment of the human *ABCC6* genes and pseudogenes was characterized and compared with other vertebrates to better understand gene family evolution and origin during the vertebrate radiation (Fig. 5). At least 18 conserved genes flank the vertebrate *ABCC6* gene suggesting that evolution of this chromosome segment in vertebrates was under conservative pressure, however in primate genomes gene insertions occurred. In human, both *ABCC6* genes and pseudogenes are on chromosome 16 and the arrangement of the neighbouring genes suggest that the two pseudogenes originated from a local genome segmental duplication (and gene inversion for *ABCC6P1*) which also involved the duplication of members of other gene families (*NOMO* and *NPIPA*). Similarly, in the genome of the gorilla a homologue genome region to the human *ABCC6P1* was found but *ABCC6P2* was absent (Fig. 5).

No genome regions homologous to that containing the primate pseudogenes or orthologues of the *NPIPA* genes, which duplicated with the *ABCC6* gene, were found in other vertebrates. A single copy of the *NOMO* gene, which is triplicated in the human genome (*NOMO 1*, *2* and *3*), was found in most of the other species analysed, and the gene mapped next to the *ABCC6* gene. Mapping of both *ABCC6* and *ABCC1* genes across the vertebrates suggests that both genes were the result of a tandem gene duplication followed by gene inversion and that this event occurred early in vertebrate evolution. A similar gene organization was generally found across all the species in which both genes were identified except in some teleosts where they map to different chromosomes (Fig. 5). In zebrafish, tetraodon and medaka, the conserved gene environment flanking *abcc6* was shared between two genome regions as the result of the teleost specific genome duplication and the resulting gene copies were subsequently deleted and only a few persisted.

Analysis of the cartilaginous fish genomes showed that the gene environment was conserved and similar to the other vertebrates, and that the absence of an orthologue of the human *ABCC6* gene was most likely a consequence of a single gene deletion. In the current lamprey genome assemblies, it was not possible to identify a homologue *abcc6* gene environment due to the short size of the genome fragments identified.

### Expression analysis in non-mammals

The tissue distribution of *ABCC6*, *ABCC1* and *ABCC3* transcripts in non-mammalian vertebrates (Supplementary Table 5) revealed they were present in tissues important for calcium homeostasis. In the chicken, *ABCC6* was expressed in the epiphyseal growth plate but also in the kidney, an organ, with an important role in calcium balance in vertebrates. In reptiles, an EST was found in the kidney and in teleost fish *abcc6* transcripts were found in the craniofacial bone elements, fins and the intervertebral discs. *ABCC1* and *ABCC3* had a wider tissue distribution than *ABCC6* (Supplementary Table 5).

### Expression of *abcc6* and *abcc1* during sea bream scale formation

**Figure 6:**
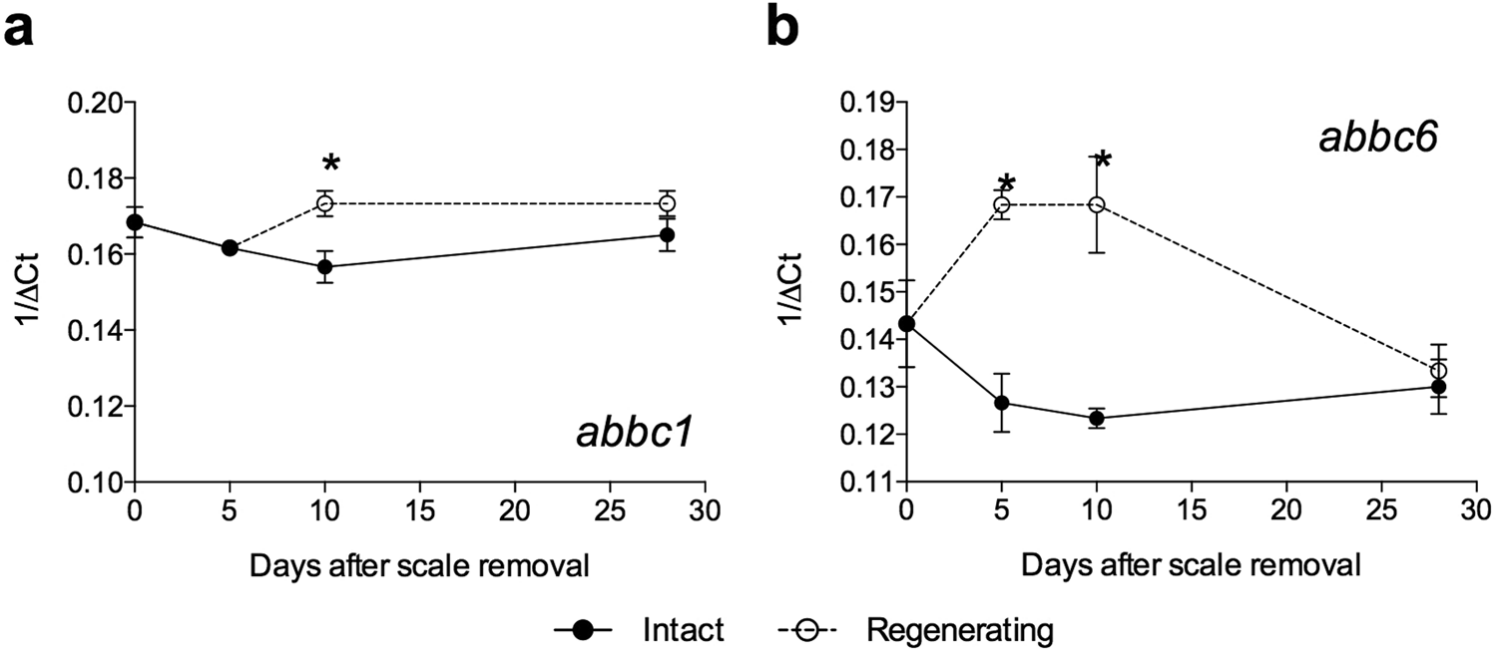
Relative expression profile of *abcc1* (**a**) and *abcc6* (**b**) during sea bream skin regeneration over 28 days after scale removal. Significant differences (p < 0.05) between intact and regenerating skin at each time point after scale removal are marked with (*).

Both transcripts for *abcc6* and *abcc1* were expressed in sea bream skin during the 28-day scale regeneration study. By the end of 28-days the scales in the damaged flank were similar to the undamaged scales in the other flank as has previously been reported^58,59^. Gene transcript abundance of the *abcc1* gene in intact and regenerating skin was generally similar during the 28-day regeneration experiment. The exception was at day 10 after scale removal when *abcc1* gene transcripts were significantly up-regulated (p = 0.0055) in the regenerating skin relative to intact skin (Fig. 6).

In the case of the *abcc6* gene transcripts a significant up-regulation occurred in skin at day 5 (p = 0.0004) and 10 (p = 0.0002) after scale removal relative to intact skin at the corresponding time points. The timing of the change in expression of the *abcc1* and *abcc6* transcripts was co-incident with the period at which the individual scale area increased and the mineralized layers were deposited in the scale (day 10–28, not shown). The results indicate that *abcc1* and *abcc6* genes are involved in skin regeneration and scale formation in teleosts.

## Discussion

In the present study, the evolution of the vertebrate *ABCC6* gene was studied and its potential involvement in fish skin/scale regeneration was investigated. Orthologues of the human *ABCC6* gene were explored in several vertebrate genomes and phylogenetic analysis revealed that human and other vertebrate *ABCC6* genes shared common ancestry and emerged early in the course of the vertebrate radiation. The *abcc6* gene was only found in bony vertebrate genomes and evidence suggests that this gene was deleted from cartilaginous fish, as no putative *abcc6* genes or transcripts were found. A teleost specific duplication of the *abcc6* gene occurred but the gene duplicates only persisted in the genomes of some species, suggesting that lineage or species-specific gene deletion occurred during their radiation. In the mapping of the human PXE mutations six amino acid positions were common between the fish and the mutated human MRP6 protein, and were located in cytoplasmic and transmembrane regions. This may indicate that the amino acids that cause the disease in humans, are not pathological in fish and this may be linked to differences in function but also the differing structure and physiology of bone. Expression analysis of the *abcc1 and 6* genes during the regeneration of damaged skin caused by scale removal revealed that these genes appear to be involved in the later stages of regeneration when intense scale growth and mineralization occur^58^. Our results provide evidence that *abcc6* gene persistence paralleled the acquisition of a bony skeleton and with the existing literature in mammals^62,63^, highlights its potential importance in the regulation of cellular calcium levels. However, the exact functional role of the *abcc* gene family in teleost skin, that has calcified scales imbricated in the dermis, remains to be determined.

Orthologues of the human *ABCC6* gene persisted in the genomes of different vertebrates which is consistent with the hypothesis of conserved function^34^. This is further supported by the high sequence and gene structure similarities between the non-mammalian and human *ABCC6* genes. In tetrapods and in the majority of teleosts and also in other fish, the coelacanth and spotted gar, a single *abcc6* gene was found. However, in the stickleback and blind cave fish two paralogue genes persisted and in the zebrafish genome three were identified. During the vertebrate radiation, duplication and deletion events have shaped genome content^64^. Genome tetraploidization occurred early during the emergence of vertebrates and prior to the divergence of the jawless fish^65,66^. In the teleosts, a subsequent genome duplication event occurred and is considered to explain the presence of gene duplicates in teleost genomes relative to tetrapods^67^. However, only some duplicates persisted in teleosts potentially due to functional redundancy^68^. The reason why duplicated *abcc6* genes persisted in stickleback, blind cave fish and zebrafish and are absent from the genomes of other teleosts is unknown, and it remains to be established if the duplicate gene copies are functional. The use of morpholinos to knockdown *abcc6a*, but not *abcc6b*, caused a visible phenotype with pericardial edema and a curled tail and was associated with death in zebrafish, suggesting that *abcc6a* is essential for normal zebrafish development^38^. This is further supported by the identification in zebrafish of a mutation in the *abcc6a* gene that causes hypermineralisation of the skeleton^39^. In zebrafish, the existence of three gene copies for *abcc6* is intriguing as this is the only teleost with a sequenced genome in which this occurs. Mapping of the duplicated *abcc6b* genes in the zebrafish genome revealed that they share the same chromosome position and thus are likely to be the result of a genome misassembly. This is also suggested by the absence of zebrafish transcripts for *abcc6b* in public databases and thus the existence of *abcc6b* paralogue transcripts remains to be confirmed. Lineage-specific gene duplications frequently occur in ABC transporter genes in teleosts^34^ and further studies are needed to elucidate their functional significance.

Our phylogenetic evolutionary analysis revealed that *ABCC1* and *ABCC6* shared a common ancestral origin with the *ABCC3* members. The tree topology obtained in our study contradicts the phylogenetic analysis recently published about the lamprey ABCC system^61^, in which the vertebrate *ABCC6* cluster radiates basal to *ABCC1*, *ABCC3* and *ABCC2*. The extensive genome analysis performed in our study revealed that, 1) the vertebrate *ABCC6*, *ABCC1* and *ABCC3* genes shared common ancestry prior to the vertebrate radiation as putative *abcc1/3/6-like* genes were found in the genomes of deuterostomes basal to vertebrates and that, 2) the vertebrate gene family members arose from the two rounds of genome doublings that occurred early in the vertebrate radiation^65,66^. This originated *ABCC3* and the ABCC1/6 genes that subsequently duplicated to give rise to *ABCC1* and *ABCC6*. In lampreys, putative *abcc1/3/6*-like genes exist and our analysis was unable to assign a specific identity to the cyclostome genes (which were previously named, *abcc1*, *abcc3a* and *abcc3b)*^61^. Orthology assignment between lamprey and gnathostome genes are difficult to establish as lamprey genomes are GC-rich in the exon coding domains relative to other vertebrates and this induces protein-codon usage bias and sequence convergence of coding sequences^69,70^. To further explore the emergence of *ABCC1*, *3* and *6* and *ABCC2* members we extended our phylogenetic analysis to include the orthologues of human *ABCC2* from other vertebrates and the resulting tree (Supplementary Fig. 4) confirmed the topology of the other trees and indicates that *ABCC2* members i) diverged prior to the chordate *ABCC 1/3/6* clade and ii) share a common origin with the vertebrate *ABCC13* family.

In human, two *ABCC6* pseudogenes have been described^43,44^. The complete and partial genes are highly similar in sequence but the pseudogenes are much shorter than the coding gene. Orthologues of the human pseudogenes were also predicted to exist in other primates such as the gorilla and chimpanzee and this suggests that they arose from a recent segmental duplication^45^, and our analysis indicates that this also involved the duplication of genes members of the *NPIPA* and *NOMO* families. The non-identification of putative *ABCC6* pseudogenes in non-primate vertebrate genomes and also the absence of members of the NPIPA family or extra copies of NOMA in other vertebrates suggests that duplication of this genome region only occurred in the evolutionary transition that led to primates^71^. The reason why *ABCC6* pseudogenes persist in primate genomes is not clear, however in humans they have been found to regulate *ABCC6* gene transcription^43^.

The emergence of the bony skeleton, resulting from an intricate physiological mechanism, was of paramount importance in the evolution of the vertebrates. The zebrafish skeleton shows similarity with human bones in terms of cells, matrix proteins, and molecular signaling pathways^72^. In zebrafish embryos, the *abcc6a* duplicate was expressed in the kupffer’s vesicles and in the tail bud, while the *abcc6b* duplicate was expressed in the enveloping layer and embryonic kidney proximal straight tubules^38^. *Abcc6a* expression is also found in mineralized tissue specifically in the osteoblasts (bone forming cells) and missense mutations of *abcc6a* caused hypermineralization of the axial skeleton, resulting in mineralized structures in the intervertebral space^39^. The mutation (L1429R) in zebrafish *abcc6a* resulted in a modification of a highly conserved region of NBD-2 that contains the Walker B motif, essential for binding to ATP^73^. The L1429 variant also occurs in the cow (L1426R; rs440576475), but no phenotype information is available. In humans, in the corresponding variant (L1425P; rs150230403) a proline substitution occurs instead of a leucine, and was not associated with PXE or any other disease. However, the two algorithm tools, Sorting Intolerant From Tolerant (SIFT^74^) and Polymorphism Phenotyping (PolyPhen^75^), that are used to predict the impact of an amino acid substitution on protein structure and function, suggested that this variant has a deleterious and damaging effect on the MRP6 protein. Modification in the preceding amino acid (variant I1424T) in human was associated with PXE. The way in which *ABCC6* is involved in the lesions of PXE is strongly linked to the *ABCC6* gene and reduced amounts of PPi *ABCC6* overexpression induces nucleotide release *in vitro*, which is rapidly converted by ENPP1 into PPi^29^. It has been reported that ATP secretion from the liver of *Abcc6*^−/−^ mice was dramatically lower when compared to wild type mice, and this suggested that MRP6 is an ATP efflux transporter^28^. ATP is converted into AMP and PPi and represents the main source of mineralization inhibiting PPi in plasma, which fully explains why the absence of *ABCC6* results in the ectopic mineralization observed in patients with PXE^28^ and in *Abcc6*^*−/−*^ mice^63,76^.

We revealed that during sea bream skin/scale regeneration *abcc6* and the evolutionary related *abcc1* gene expression was up-regulated relative to the control (intact skin) at 5 and 10 days after damage. This is intriguing as the change in gene expression was coincident with the phase of intense scale growth and mineralization and is in agreement with the high expression of *abcc6a* detected in zebrafish at sites of mineralisation, unlike the mammals where *ABCC6* is mainly produced by the liver^39^. Further studies are required to characterize in more detail the role of *abcc6* and *1* in teleost skin/scale regeneration.

Our preliminary expression analysis in a teleost skin regeneration model revealed that the *abcc6* gene is likely to be involved in controlling scale mineralization during skin/scale regeneration. In fact, it should be noted that mineralization in PXE and its mouse model *Abcc6*^−/−^, is not noted at birth, but develops later in life. Probably the *abcc6* gene in fish skin acts as a promoter of scale mineralization, since its expression increases at the stage of intense mineralization. We hypothesize that it may also act as an inhibitor of pathological ectopic calcification through a PPi dependent mechanism. Overall, the presence of the *abcc6* gene in teleost fishes, its presence only in the genomes of organisms with a bony skeleton, and up-regulation during intense mineralization of the scales suggests that this gene emerged associated with the need for more sophisticated mechanisms to control intracellular and extracellular PPi. Future studies will be aimed at understanding, at a molecular and cellular level, the function of *abcc6* in developing and regenerating teleost skin.

## Electronic supplementary material

Supplementary data
