# Supplementary material for: Persistence of the ABCC6 genes and the emergence of the bony skeleton in vertebrates
Source: Sci Rep. 2018 Apr 16;8:6027. doi: 10.1038/s41598-018-24370-7 (PMC5902450; doi:10.1038/s41598-018-24370-7)
Supplement: Supplementary file 1 — Supplementary data [file 41598_2018_24370_MOESM1_ESM.docx]

**Persistence of the *ABCC6* genes and the emergence of the bony skeleton in vertebrates**

Bruna Parreira ^1+^, João CR Cardoso ^2+^, Rita Costa ^2^, Ana Rita Couto ^1^, Jácome Bruges-Armas ^1,3^, Deborah M Power ^2,4*^

(1) Serviço Especializado de Epidemiologia e Biologia Molecular (SEEBMO), Hospital de Santo Espírito da Ilha Terceira, Azores, Portugal;

(2) Centre of Marine Sciences (CCMAR), Universidade do Algarve, Campus de Gambelas, 8005-139 Faro, Portugal;

(3) CEDOC – Chronic Diseases Research Center, Universidade Nova de Lisboa, Portugal.

(4) Key Laboratory of Exploration and Utilization of Aquatic Genetic Resources, Ministry of Education, Shanghai Ocean University, Shanghai, China

^+^These authors contributed equally to the work

**LEGENDS**

**Supplementary Figure 1. Sequence alignment of the MRP1 and MRP6 proteins in gnathostomes.** The selected 138 amino acids mutated in human PXE disease are colored in red and conserved amino acids are annotated with different shades of grey according to the degree of conservation (lighter, poorly conserved to darker, highly conserved). The alignment was manually edited to remove large sequence gaps. A list of abbreviations and accession numbers are given in Supplementary Table 1.

**Supplementary Figure 2. Bayesian inference (BI) phylogenetic tree of the ABCC 6, 1 and 3 members with the other human ABCC family members.** The phylogenetic tree was built in MrBayes. The tree was rooted using the protostome and other human *ABCC* sequences. The vertebrate *ABCC1*, *3* and *6* clades are boxed with different colors. Invertebrate sequences are identified by their common names following the last digits of their accession number. Accession numbers of the other human *ABCC* family members are indicated in the tree.

**Supplementary Figure 3. Maximum likelihood (ML) phylogenetic tree of the *ABCC6*, *1* and *3* members with the other human ABCC family members.** The phylogenetic tree was built using the ML method in PhyML 3.0 software with 100 bootstrap. The tree was rooted using the protostome and other human *ABCC* sequences. The vertebrate *ABCC1*, *3* and *6* clades are boxed with different colors. Invertebrate sequences are identified by their common names following the last digits of their accession number. Accession numbers of the other human *ABCC* family members are indicated in the tree.

**Supplementary Figure 4. Maximum likelihood (ML) phylogenetic tree of the *ABCC2* genes with the other *ABCC* family members.** The phylogenetic tree was built using the ML method in PhyML 3.0 software with 100 bootstrap. The tree was rooted using the human *ABCC* 4/5/8/9/10/11/12/CFTR cluster. Members of the *ABCC13* family (that is a pseudogene in human) were also included in the analysis. The orthologues of human *ABCC2* were retrieved from the ENSEMBL database based on sequence similarity. The *ABCC1*, *3* and *6* clades were collapsed and *ABCC2* and *ABCC13* clades are boxed in different colours. Invertebrate sequences are identified by their common names following the last digits of their accession number. Accession numbers of the *ABCC2* and *ABCC13* are indicated in the tree.

**Supplementary Table 1. Accession numbers of the vertebrate *ABCC6*, *ABCC1* and *ABCC3* genes and transcripts.** Most vertebrate gene sequences were retrieved from the Ensembl (<http://ensembl.org/>, accessed 2018) database. The Elephant shark orthologues were obtained from (<http://imcbgenomeprojects.imcb.a-star.edu.sg/>, accessed 2018), the Whale shark and Arctic lamprey genes were retrieved from the NCBI species genome assemblies, the little skate, small spotted catshark transcripts were retrieved from Skatebase ([http://skatebase.org](http://skatebase.org/)) and the dogfish transcripts from NCBI (cartilaginous fishes, taxid:7777). The Sea lamprey transcripts were annotated according to (60).

**Supplementary Table 2. Accession numbers of the *ABCC* genes retrieved from ciona, amphioxus and sea urchin and from several protostome genomes.** Sequences were retrieved from the Ensembl Metazoa database (<http://metazoa.ensembl.org/index.html>).

**Supplementary Table 3. The percentage of amino acid sequence identity/similarity of vertebrate MRP6 and MRP1.**

**Supplementary Table 4. Exon and intron size of the human *ABCC6* genes (full-length and the two pseudogenes) and the orthologues in a ray-finned fish, the spotted gar.** The predicted exon (E) and intron (I) sizes were retrieved from the ENSEMBL gene annotation and are given in base pairs. The gene structure for the human *ABCC6* (ENST00000205557.11), *ABCC6P1* (ENST00000546162.6) and *ABCC6P2* (ENST00000526069.1) and spotted gar (ENSLOCT00000008702.1) transcripts are represented. Exon sizes are highlighted in bold. E2a represents the exon that is only present in human *ABCC6* pseudogenes. * from ATG to the STOP codon.

**Supplementary Table 5. The expression profile of the *ABCC6*, *ABCC1* and *ABCC3* transcripts in human, bird, reptile, amphibian and a teleost, the zebrafish.** ESTs were obtained from the NCBI EST database. Searches were also performed in other databases: Geisha (<http://geisha.arizona.edu/geisha/>); Xenbase (<http://www.xenbase.org/entry/>); Expression Atlas (<https://www.ebi.ac.uk/gxa/home>), GeneCards (http://www.genecards.org/), Ensembl <http://www.ensembl.org/index.html>), complemented with published data (37).

**Supplementary Data 1.** **Sequence alignment of the** **ABCC 6, 1 and 3 used for phylogenetic tree construction.** The alignment was manually edited to remove gaps and poorly aligned regions.

**Supplementary Figure 1**

* 20 * 40 * 60 * 80 * 100
Hsa_6 : MAAPAEPCAGQGVWNQTEPEPAATSLLSLCFLRTAGVWVPPMYLWVLGPIYLLFIHHHGRGYLRMSPLFKAKMVLGFALIVLCTSSVAVALWKIQQGTPEAPEFL : 105
Ptr_6 : --------------------------------------------------------------------------------------------------------- : -
Ggo_6 : MAAPAEPCAGQGVWNQTEPEPATTSLLSLCFLRTAGVWVPPMYLWVLGPIYLLFIHHHGRGYLRMSPLFKAKMVLGFALIVLCTSSVALTLWKMQQGTPEAPEFL : 105
Mmu_6 : MATPGEQCAGLRVWNQTEQEPAAYHLLSLCFVRAASSWVPPMYLWVLGPIYLLYIHRHGRCYLRMSHLFKTKMVLGLALILLYTFNVAVPLWRIHQGVPQAPELL : 105
Cluf_6 : MAAPAEPCLGQAVWNWTEPEPTAAHLLNVCFLKTAGVWMPPMYLWVLGPIYLLYIHRRGKGYLRMSPLFKAKMVLGLALIILCTSSVSVALWRIQRGMPQAPEIL : 105
Bta_6 : MAGQGEPCAGPGVWNQTEPEPAAARLLSLCFLKTAGVWVPPMYLWVLGPIHLLYIHRHDKGYIQMSRLFKAKMVLGFALIILCTSSVSVTLWRIQQGTPQALEFL : 105
Dno_6 : -------------------------------------------------------------------------VLGFALIVLSTSNVSVALWKIHRGTPQAPELL : 32
Mdo_6 : GSGVCNPVLGN--WNWNETQSLGLQMSSLCLMNAAVAWLPSVYLWVISPFYFLYLRYNNKGYIRMSCLFKTKMVLGFTLVLLCFSNIIFTLWKIKKGIPQAPEFL : 103
Oan_6 : ----------------------------------------------------------------MSRLFKVKMVLSFALMSLCFFNLCFTLWKIKQGTPQALELL : 41
Gga_6 : GRLCGSREGSAGLWDWNQTWYTDSPRFTWCFENTVLSWIPCAYLWICFPFYYLYLQHKNKGYIRMSHIFKIKMVLGFLLVILCFSNVFFVLWEISQGIPRPPAFF : 105
Aca_6 : --------------------------------------------------------------------------LGFVFLAVYFASTCYILWEANHGIQQDPGLA : 31
Xtr_6 : ---------------------------SQCFHNSVLNWIPSIFLFICSPFYVLYLRRHGRGYIRMSALSKAKTCLGALLVLVCYTELFYTVWNMTHNVRQAPVFL : 78
Lch_6 : MDQFCRVDGSDPFWDWNQTWFTDRPDFTGCFQITILVWLPCTFLWLCSPFYCWYLQRHGNRYIRMSRLYKAKTVLGAILILLCVSEFFITVWEINQGTLRALAFL : 105
Tn1_6 : MDAFCQLSGLDPLWDWNRTWYTTNPDLTQCFQNTVLVWVPCIYLWLLAPFYCLHLYCHDHGRIQMSGLFAAKTVLGFLLASFGCVEFFYILLERSQEIHQHMVFL : 105
Gac_6a : MEDLCSVSGLDPLWDWNLTWYTSQPDLTQCFQHTVLVWSPCVYLWICSPFYLLYLWRPDRGVIPLSKLCCSKTLLGLSLASFGLVEMLFLLVTKNEEIQKHSLIV : 105
Gac_6b : MDAFCRISGLDPLWDWNRTWYTANPDLTQCFQNTVLVWVPCIYLWLLAPFYCLHLYCHDSGRIRMSCLCCAKTMLGFLLASFGFVEFFYILLERSQEIEQHMVFL : 105
Dla_6 : MDETYNLTVLPQFLDWNRTWYTANPDLTQCFQNTVLVWVPCIYLWLLAPFYCLHLYCHDRGRIRMSCLCTAKMVLGFLLASFGFVEFFYILLERSQEIQQHMVFL : 105
Oni_6 : MDAFCRLSGLDPLWDWNRTWYTANPDLTQCFQNTVLVWVPCIYLWLLAPFYCLHLYCHDRGHIQMSCLCSAKMVLGFLLASFGFVEFFYILLERSQDIQHHMVFL : 105
Ola_6 : IAAVCSRQSMCSFHDWNRTWYTANPDLTQCFQNTVLVWVPCIYLWLLVPFYCLHLYCHDSGRIRMSCLCMAKMVLGFLLASFGFVEFFYILLERSQDIGHHMVFL : 105
Xma_6 : MDAFCRLSGLDPLWDWNRTWYTANPDLTQCFQNTVLVWVPCVYLWLLVPFYCVHLYCHDRGRIQMSCLCTAKMMLGFLLASFGFVEFFYILLERSQDIQHHMVFL : 105
Gmo_6 : MDTFCKLSGLDPLWDWNRTWYTASPDLTHCFQNTVLVWVPCVYLWLLAPFYCLHLYCHGNGRIRISCLCTAKMVLGFLLASFGFVEFFYILLERSGEIHQHMVFL : 105
Dre_6a : MDTFCSLSGLDPLWDWNQTWYTHRPELSNCFQNTVLVWAPCIYLWVLSPFYCLHLYCHGRGRLPLSSLCNAKLLLGFFLASFGFVEFFYILLERRLEIHQHLVFL : 105
Dre_6b1 : -----------------------------------------FYLWICAPFYCLYLKFYYNGRISISSLCCAKMGLALCLASIGFLETVYLLVERSRDIEHLMVFL : 64
Dre_6b2 : --------------------------------------------------------------------------------------------------------- : -
Ame_6a : MDTFCSLNGLDPLWDWNQTWYTHTPELSDCFQHTVLVWAPCIYLWICSPFYCLHLYCHGRGRLPLSLLCSAKLLLGFFLASFGFVEFFYILLERNQEIHRHLVFL : 105
Ame_6b : KSSFVTVSVFQ---NWNLTWFTPNPDLTECFQHTVLVWFPCFYLWLCAPFYFLYLGFHDYGRISVSSLCVTKTVLGLSLAFFSLLELVYLLVSRSGELNNHMVFL : 102
Loc_6 : MDAFCSLSGLDPLWDWNRTWYTPNPDLTQCFQNTVLVWVPCVYLWVCAPFYCLYLHCYDRGYIRVSCLCCAKMVLGFLLASFGFVEFFYILLERNREIQQHLVFL : 105
Hsa_1 : LRGFCSADGSDPLWDWNVTWNTSNPDFTKCFQNTVLVWVPCFYLWACFPFYFLYLSRHDRGYIQMTPLNKTKTALGFLLWIVCWADLFYSFWERSRGIFLAPVFL : 105
Ptr_1 : --------------DWNVTWNTSNPDFTKCFQNTVLVWVPCFYLWACFPFYFLYLSRHDRGYIQMTPLNKTKTALGFLLWIVCWADLFYSFWERSRGIFLAPVFL : 91
Ggo_1 : LQASSCPLGCVLLTDWNVTWNTSNPDFTKCFQNTVLVWVPCFYLWACFPFYFLYLSRHDRGYIQMTPLNKTKTALGFLLWIVCWAGLFYSFWERSRGIFLAPVFL : 105
Mmu_1 : LRSFCSADGSDPLWDWNVTWHTSNPDFTKCFQNTVLTWVPCFYLWSCFPLYFFYLSRHDRGYIQMTHLNKTKTALGFFLWIICWADLFYSFWERSQGVLRAPVLL : 105
Cluf_1 : LRGFCRADGSDPFWEWDVSWNTSNPDFTKCFQNTVLVWVPCCYLWLCFPFYFLYLSRHDRGYIQMTYLNKTKTALGFVLWIVCWADLFYSFWERSWGKILAPVFL : 105
Bta_1 : --------------EWNVTWNTSNPDFTKCFQNTVLVWVPCSYLWVCFPFYFLYLSHHDRGYIQMTHLNKAKTALGFLLWIVCWADLFYSFWERSMGKLLAPVFL : 91
Dno_1 : SRGDSYGQGFFPLWDWNVTWHTDSPDFTKCFQNTVLVWVPCCYLWACFPFYFLYLSRHDRGYIQMTHLNKAKTALGFLLWIVCWADLFYSFWERNQGLFVAPVFL : 105
Mdo_1 : LPRFCSADGSDPLWDWNITWHTDNPDFTKCFQNTVLVWVPCVYLWACFPFYFLYLCRHNRGYIQMIHLNKAKTALGFLLWIVCWADLFYSFWERSQSIYRAPVFL : 105
Oan_1 : --------------------------------------------------------------------------------------------------------- : -
Gga_1 : IESLCSADASEPFWDWNLTWHTENPDFTQCFQNTVLVWVPCIYLWVCFPAYFLYLRSHDRGYIQMSILNKAKTALGLILWIVCWADLFYSFWERSQNIFRAPFFL : 105
Aca_1 : --------------DWNLTWNTPRPDFTPCFQNTVLAWTPCAFLWACFPFYAFFLRRHDKGYIQMSRLNKAKTALGFLLWIVCWADLFYSFWERSRNIYQAPVYL : 91
Xtr_1 : MESFCSYDGSERFWDSNLTWYTENPDFTKCFQNTVLIWIPCIYLWFCLPFYFAYLRKNDQGYIQMSHLNKAKTAIGFILWLACWADLFYSFWERSQSIYRAPVYV : 105
Lch_1 : IEALCAKDGSDPFWDWNQTWYTENPDFTKCFQNSVLVWIPCVYLWVASLFYYLYLRRYGRGYIRMSWLNKTKTVLAVLMWLICWVDIIIFFMERFQKINSAPILI : 105
Tni_1 : FARFCTSNESDLFWDWNRTWYTDNPDFTQCFQNTVLVWIPCLYLWICGPIYMLYLHSHSHGYICMNHLNKAKTAVCLLLWVLCWSDVFYTFWERSQKSNVPLVYL : 105
Gac_1 : LHGFCSADTSDPFWDWNRTWYTTKPDFTQCFQNTVLVWLPCLYLWICAPLYLLYLRSHDHGYIRMSHINRAKTAVGLLLWIICWADVFFSFWERSYGSNLAPVHL : 105
Dla_1 : MNT-----------DWNRTWYTANPDLTQCFQNTVLVWLPCLYLWMCAPLYLLYLRGHDRGYICMSHLNKAKTAVGLLLWIICWADVFYSFWERSHGSRPAPVYL : 94
Oni_1 : FDQFCSVDRSDPLWDWNRTWYTDNPDFTQCFQNTVLVWLPCFYLWICAPFYLVYLHTHDHGYICMNHLNKAKTAVGFLLWIICWSDVFYSFWERSHVSSPAPVRL : 105
Ola_1 : LDRFCSPNSSEPFWDWNRSWNTSNPDLTPCFQSTVLVWVPCLYLWLCAPFYLMYMRSHNRGYICMSHLNKAKTAVGFLLWIICWLDVFYSFWERSHSRNVAAVHL : 105
Xma_1 : ----------------------------------------CFYLFACLTVQYFLNSISMKEYRTRRHKNKSQNVVGFLLWVVCWSDVFYSFWERNHSSNTAPVHL : 65
Dre_1 : IDSFCSLDGSDPLWDWNRTWQTYYPDLTPCFQNTVLVWIPCLYLWLFAPLYILYLKSHDRGYICMTHLNRAKTVIGFTLWLICWADVFYSFWERSHGATVAPVYL : 105
Ame_1 : IDHFCSVDGSDPFWDWNRTWHTHNPDLTQCFQNTLLVVVPCFYLWLFAPFYFLYLKSHDRGYICMTHLNKAKTVTGFLLWIICWADVFYSFWERGHGVASAPVYL : 105
Loc_1 : IDEFCSLDGSDPFWDWNRTWYTPNPDLTQCFQNTVLVWVPCVYLWVCAPFYCLYLHCNDRGYIRMSHLNKAKTVTGFLLWIVCWADVFYSFWERSQGQAKAPVYF : 105
Cmi_1 : --------------DSNLTWHTQDPDFTICFKKTVLIWIPCIFLWLCFPFYTLFLYYRKQGYIRMSNLNKSKTLLGFLLWLSCWSQVLNIILEKSRGFGHATVLI : 91
Ler_1 : --------------------------------------------------------------------------------------------------------- : -
Rty_1 : --------------------------------------------------------------------------------------------------------- : -
Sca_1 : --------------------------------------------------------------------------------------------------------- : -


 * 120 * 140 * 160 * 180 * 200 *
Hsa_6 : IHPTVWLTTMSFAVFLIHTERKKGVQSSGVLFGYWLL---CFVLPATNAAQQASGAGFQSDPVRHLSTYLCLSLVVAQFVLSCLADQPPFFPEDPQQSNPCPETG : 207
Ptr_6 : --------------------------------------------------------GFQSDPVRHLSTYLCLSLVVAQFVLSCLADQPPFFPEDPQQSNPCPETG : 49
Ggo_6 : IHPTVWLTTMSFAVFLIHTERKKGVQSSGVLFGYWLL---CFVLPATNAAQQASGAGFQSDPVRHLSTYLCLSLVVAQFVLSCLVDQPPFFPEDPQQSNPCPETG : 207
Mmu_6 : IHPTVWLTTMSFATFLIHMERRKGVRSSGVLFGYWLL---CCILPGINTVQQASAGNFRQEPLHHLATYLCLSLVVAELVLSCLVDQPPFFSEDSQPLNPCPEAE : 207
Cluf_6 : IHPTVWLTTMSFAMFLIHTERKKGVRASGVLFGYWML---CFLLPITSTAQLTLQGDFRSDPFSHLATYLCLSLVAAQFVLSCLVDQPPFFPKDPQQSNPCPKAE : 207
Bta_6 : IHPTVWLTTMSFAVFLIHAERKKGVQASGVLFGYWLL---CFLFPATSATQQASRGDFQSDPFRHLSPYLYLSLVMAQFALSCLADQCPLFRKRPPQANPCPKAG : 207
Dno_6 : IYPTVWLTTMSFAMFVIHMERRKGVQASGVLFGYWLL---CCLLPATNTAQLVLRGDFQRDAFRHLSTLLCLSLVAVQLVLSCLADWPPFFPKAPQQPNPCPEAG : 134
Mdo_6 : INPTVWLITMILAIFLIHLERRRGIQSSGVLFIYWLLCSFSMAVTVSATVHQALQGGFPEDTFRHLITYFHSALIGAQFVLSFLADQPPFFSKIMHDSNPCPESG : 208
Oan_6 : INPAMLLITMSLTVFLIHFERLKGVQSSGVLFVYWLLSFLVTLVTLSATVQHALQGGFPRDAFRHIVSYLYSALVGAQFVISFFADQPPFFAKVPQESNPCPESG : 146
Gga_6 : ISPAVLGITMILAMFLTQVERMMGIQSSGIMLIYWLLTFLSALVMFSSKIQRGLERGFLEDFFHHVATYLYASLVLGELVLFCLVDHPPFFSKAVNSSNQCPEAS : 210
Aca_6 : ISCALQLATMILVLFLTQTERQKGVQSSGLLLLYWLLSFLSATASLISKIQEAREGGFRSAPFHHATSYIYFTLVSLELGLCCLVDQPPFFSKVDSDANPCPESR : 136
Xtr_6 : ISPLILGSSMLLATCIIQYERMQGVRSSALLLFFWLLALLCATFQLRTKITTAISE---IDKLRYTLFVLYFVFVLAQSVLCTFNDDPPFFSNLKKESNPCPVSE : 180
Lch_6 : LSPAILAFTLVLATFLLHYERVKGVQSSGVLLFYWLLSLLCAVFPFRSKIQQAPPDGSVIHSFRFGMFYSYFAMVLAQLILCCFTEPPPFFSEERKAPNPCPESN : 210
Tn1_6 : LSPIIRSMTVVLVLCIIQLERVRGCRSSVFLFLFWVMAVVCSLVPLRAKIQLAMDEGFASDIVQYFAFFSYFTIQLAQLFLCCFADQPPV-GKTILEKNPCPVKD : 209
Gac_6a : IGPLIRSLTLVLAVIILHVERMKGCRSSFLLFQFWILLVLCSLVPLKVDIEQIIDRGFSSDSSRLLLFFLCFFLQLIQLVLSCFCDLRPLCAKQSYVQNRCPEED : 210
Gac_6b : LSPIIRSMTVILALCIIQLERIRGCRSSVFLFLFWVLAVVCSLVPLRAKIQLAMDEGISSDIVRYFAFFSYFTIQLAQLFLCCFADKPGSTSKSAGDENPCPVKD : 210
Dla_6 : LSPIIRSMTVILALCIIQLERVRGCRSSVFLFLFWVLAVVCSLVPLRAKIQLAMDEGIASDIVRYLAFFSYFTIQLAQLFLCCFADQPPE-GKTILEKNPCPVKD : 209
Oni_6 : LSPIIRSLTVILAMCIIQLERIRGCRSSIFLFLFWVLAVVCALVPLRAKIQLAMDEGIASDIVRYLAFFSYFTIQLAQLFLCCFADQPPE-GKIISEKNPCPVKD : 209
Ola_6 : LSPIIRSMTVILALCIIQLERIRGCRSSIFLFLFWVLSVVCSLVPLRAKIQLAVDEGIASDIVRYLAFFSYFTIQMAQLFLCCFADQPPQ-GKPNLEKNPCPVKD : 209
Xma_6 : LSPIIRSMTVILALCIIQLERIRGCRSSVFLFLFWVLSVVCSLVPLRAKIQLAIDEGIASDIVRYLAFFSYFSIQLAQLFLCCFADRPPQ-GKPVLEKNPCPVED : 209
Gmo_6 : LSPIVRSVTVILALFIIQLERLRGCRSSVFLFLFWVLAVVCSLVPLRAKIQLAVDEGIGSDVVRFLAFFSYFTLQLAQLVLVCFADR----------RNPCPVKD : 200
Dre_6a : LSPIMRSLTVVLAVCVIHWERVRGCRSSVFLFFFWLLGVLCSIIPLHAKVQLAVEQGLSPDIVRYLAFFSYFALQLAQLFLSCFADQAPL--GKAVHKNACPVQD : 208
Dre_6b1 : LSPIIRSLTMILVMLMIHLERLRGFRSSVFLFLFWMLSVVCSLVPLRANIQANIKEGFSADPMRFAAFFTFFSLQLAQLILSCFADQRPDTLKPVYVKNPCPVED : 169
Dre_6b2 : --------------------------------------------------------------------------------------------------------- : -
Ame_6a : LSPIVRSLTVVLAVCIIQWERVRGCRSSVVLFLYWLLGVICSLVPLRAKIQLAVEQGFSPDIVHYLAFFAYFALQLAQLFLSCFADQAP--PGKVALKNACPVQD : 208
Ame_6b : LSPVIRSLTMVLTVCVIHLERMKGCRSSLFLFVFWTLAVVCSLVPLRANIQAVVGESCSRDSVRSAAFFTCFSLQLAQLILSCFADQRSDDLKWVDVKNPCPVED : 207
Loc_6 : LSPVIRSLTVVLAVLIIQFERVRGSRSSAFLFLFWLLAVVCSLVPLRAKIQLAIDEGFSADAVRYLAFFSYFTLQLAQLFLSCFSDQPPYTRRPVKVPNPCPVQD : 210
Hsa_1 : VSPTLLGITMLLATFLIQLERRKGVQSSGIMLTFWLVALVCALAILRSKIMTALKEDAQVDLFRDITFYVYFSLLLIQLVLSCFSDRSPLFSETIHDPNPCPESS : 210
Ptr_1 : VSPTLLGITMLLATFLIQLERRKGVQSSGIMLTFWLVALVCALAILRSKIMTALKE---------ITFYVYFSLLLIQLVLSCFSDRSPLFSETIHDPNPCPESS : 187
Ggo_1 : VSPTLLGITMLLATFLIQLERRKGVQSSGIMLTFWLVALVCALAILRSKIMTALKEDAQVDLFRDITFYVYFSLLLIH--------------------------- : 183
Mmu_1 : VSPTLLGITMLLATFLIQLERRKGVQSSGIMLTFWLVALLCALAILRSKIISALKKDAHVDVFRDSTFYLYFTLVLVQLVLSCFSDCSPLFSETVHDRNPCPESS : 210
Cluf_1 : VSPTLLGITMLLATFLIQLERRKGVQSSGIMLTFWLIALLCALAILRSKIMTALKEDAEIDVFRDVTFYIYFSLVLIQLVLSCFSDRPPLFSETIHDLNPCPESS : 210
Bta_1 : VSPTLLGITMLLATFLIQIERRRGVQSSGIMLTFWLIALLCALAILRSKIMTALKEDARVDVFRDVTFYIYFSLVLIQLVLSCFSDRSPLFSETINDPNPCPESS : 196
Dno_1 : VSPTLLGITMLLATLLIQLERRKGVQSSGIMLTFWLVALLCATAILRSKIMTALKKDAGVNVVRDVTFYIYFSLVLIQLVLSCFSDRSPLFSETINDPNPCPETS : 210
Mdo_1 : VSPTLLGITMLLATFLIQCERRKGVQSSGVLLTFWLLALLCAGIVFRSKVLHALRASDKIDIFRDVTFYIYFLLVLAQLILSCFSDHSPLFSETINDPNPCPESG : 210
Oan_1 : --------------------------------------------------------------------------------------------------------- : -
Gga_1 : ISPTVLGITMLLATFLIQHERLKGVQSSGVMMIFWLISLLCATVIFRSKIMLALNTDTEVDAFRYVTFCTYFILLLVQLILSCFPEKPPLFSEAVNDPKPCPEFS : 210
Aca_1 : VSPTILGITMLLATFLIQYERMKGVQSSGIMLLFWLIALLCASVTFRSKILQASDAVKGFDVFRCITFFLYFALVLTELVLSCFPERPPLFSETVHDPSNRPRIK : 196
Xtr_1 : VSPTVLGITMLLATFLIQYERIKGVQSSGVMLNFWIVALLCAIIIFRSKVLHALKPDAQIDVFRDTTFYIYFLLVLVELILSAFPDRPPLFSERVNDPNPCPESS : 210
Lch_1 : ISPTVLGATMLLAALLIQYERKKGIQSSGILMIFWLTALICATVTFRSKIIYALQKDADVDVSRYVTFYVYYMLLLVQLVLSCLTDQPPLFSEEVKDENPCPEYS : 210
Tni_1 : VSPTLLGLTMLLCAALIQSERLKGVQSSGVLFIYWLLALLSATFILRSKILHALEQSLTAFPWRHTTFYIYYGLLLAAFVLSCLTDQPPLFCAVVKNSNPCPEPG : 210
Gac_1 : ISPTMLGFTMLLATLLIQYERMKGVQSSGVMLLYWLLALLCATVTFGSKISRALDQPLTVSVWRYTTFYTYYALLLVSLCLSCLTDQLPLFSEAVKDSNPCPERG : 210
Dla_1 : VSPTLLGLTMLSA----------------------------------------------VYVWRYTTFYIYYALLLVALFLSCLTDQPPLFSQAVKDSNPCPELG : 153
Oni_1 : VSPTLLGLTMLLAVMLIHYERMKGAQSSGVMLIYWLLALLCATVTFRSKIFQALEQPQTVCVWRYTTFYIYYALLLIALFLSSLTDQPPLFSRDVKDSNPCPEPG : 210
Ola_1 : VSPTLLGLTMLLATLLVQYERMKGVQSSGIMLIFWLLALLCASVTFRSKILQAQDQPEAVSGWRYTTFYVYYALLLLALVLSCLSDQMPLFSQAVKDPNPCPEPG : 210
Xma_1 : VSPTLLGLTMLLATFIIQYERLKGVQSCGILLIFWLLALLCATVSFRSKILQARNEPETVCIWKYTTFYIYYAFLLVALILSCLTDQPPLFSQATKELNPCPEPG : 170
Dre_1 : VSPTMLGVTMLLATFLIQYERMKGVQSSGVMLNFWLITIVCATITFRSKIMHALNDPASVGVFRYTTFYIYYTLLLISLILACLSDQPPLFSEVVKDSNPCPESG : 210
Ame_1 : VSPTILGITMLLATLLIQYERIKGVQSSGIMLNFWLVATVCATVTFRSKILQAVNEPETVNVFRYSTFYIYYALLLISLILSCLSDQPPLFSQAVKDSNPCPEAG : 210
Loc_1 : VSPTLLGITMLLATFLVQYERMKGVQSSGVLLNFWVIAVICGTISFRSKILQAFSETSGVDLFRYFTFFTYFALLLIQLFLSCLSDQPPLFSQAVKDSNPCPELG : 210
Cmi_1 : LGPAFLGVTMLLAVFIIQFERLKGLRSSAVMFLFWLLTLLCSTIEFRSTVMNLLYPPAHFDLVDHIIFFFNFTMVLAEFVLCCFTDDPPFF-QLILSTNPCPESK : 195
Ler_1 : --------------------------------------------------------------------------------------------------------- : -
Rty_1 : --------------------------------------------------------------------------------------------------------- : -
Sca_1 : ---------MLLAVFLIQYERLKGVQSSAILLIFWLLALLCAIPTLRSKIMHSIDQVSYIDVFRDSTFYLYFVLVLIELVLSCLTDQPPLFSESVKDSNLCPELS : 96


 220 * 240 * 260 * 280 * 300 *
Hsa_6 : AAFPSKATFWWVSGLVWRGYRRPLRPKDLWSLGRENSSEELVSRLEKEWMRNRSAARRHN---------------KAIAFKRKGGSGMKAPETEPFLRQEGSQWR : 297
Ptr_6 : AAFPSKATFWWVSGLVWRGYRRPLRPKDLWSLGRENSSEELVSRLEKEWMRNRSAARRHN---------------KAIAFKRKGGSGMEAPETEPFLRQEGSQWR : 139
Ggo_6 : AAFPSKATFWWVSG--------KRKKKSAWALEAESWNQIPVLLLLKYKMGPRANLSL----------PLRHN--KAIAFKRKGGSGMKAPETEPFLRQEGSQWR : 292
Mmu_6 : ASFPSKAMFWWASGLLWRGYKKLLGPKDLWSLGRENSSEELVSQLEREWRRSCNGLP-----------------------GHKGHSSVGAPETEAFLQPERSQRG : 289
Cluf_6 : ASFLSRAMFWWVSGLVWRGYRRLLGPEDLWSLGRENSSEELVSQLQREWTRTRSAAQQHT---------------KARDAKRKGSRDVEAPEMEALLQQEGSQRG : 297
Bta_6 : ASFPSKAMFWWVSGLVWKGYRRPLGPKDLWSLGSKNSSEELVSQLEKEWTRNRSATQRHT---------------KATAFKRKGSHNKEAPETETLLPQQRGKRG : 297
Dno_6 : ASFPSKATFWWVSGLVWRGYRRPLGPKDLWSLGKESSSEELVSRLEREWTRNRRAAQRRHL--------------KAKASKRKGGAGPEVPETEAFLRQEGSQRG : 225
Mdo_6 : ASFPSKVTFWWFSRLVWQGYRKPLEMDDLWSLGKENSSEEIISRLESEWKRICNETQQTKEEM------------GFERGGGNRAEPALPPETETFLQGHQSPRF : 301
Oan_6 : ASFPSKVTFWWFSRLVWQGYRRPLEPDDLWSLQRENSSEELVSQLEREWKKNH------------HQTPWSPDAVALNRDGQLRDEAADPWETQPFLQSERTQSG : 239
Gga_6 : SSFLSKITYWWFSGLVWKGCRQSLGVDDLWSVRKEDSSEEIVAWAEREWKKYNNRTKQKMES-ATFKKSW-----------KIGTDTAEAEETEVLLQSEHSQSG : 303
Aca_6 : ASFLSRITFWWFAGTIWKGYWKPLQREDLWSLAKENSSEEIVAKFKDAWEKHCASAEDKFPM-NLTTSEISES--ATCKREKRKSQTARETA--LLLQPENSKSK : 236
Xtr_6 : SSFLSKVTFSWFTEIMFRGYKQPLKAEDVWSLRKSDTAEEILTLFSKGVEKECANLQNKLIV-------------AAESLGLGLPRETEKSEIELLLKNRHIQLS : 272
Lch_6 : ASFLSKVTFWWFTGQVIKGYRRPLVAEDLWSLRKENRSDEIVRHLEREWKKEYAKGKQSVETV------------QFSKKQRHSGASLKQPEETQVLMKRQGEQS : 303
Tn1_6 : ASFLSKLLFWWFTGLVVKGYRNPLAAEDLWTLREEDTSCKIIAELQQDWTAECAKIQNQGVR---------THSGQQKALASNAALGSRLPDQAQLLRKLQKEQS : 305
Gac_6a : ASFLSNFFFSWFSGLVVRGYRHPLQAADLWPLRDQDSSIRIMTDFENLWAQNCKPLQEEPDN-VELTQNWTQS-------TTSSGSLSRAWDKTQLLKKKKKGQK : 307
Gac_6b : ASFLSKILFWWFTGLVVKGYRTPLEAEDLWTLREEDTSRKIIAELEEDWTDECAKVQNN----SLFSCRQEKA------LAAGAALGSRLPDQAQLLRKLQKEQS : 305
Dla_6 : ASFLSKILFWWFTGLVVKGYRTPLAAEDLWTLREEDTSNKIISELQQDWTAECAKIQKQEKA-----------------LASGVALGSRLPDQAQLLRKLQKEQS : 297
Oni_6 : ASFLSKILFWWFTGLVVKGYRTPLEAGDLWTLREEDTSQKIISDLEQDWTAECAKLQKIMEPIFLCQPDKQSS------FKSSNCNADFLLK-ILILRSPRLYWD : 307
Ola_6 : ASFLSKILFWWFTGLVVKGYRTPLEATDLWTLREEDTSHKIISDLQQEWGAECAKLQKQEKS-----------------LESAPVLGSRLPDQAQLLRKLQKEQS : 297
Xma_6 : ASFLSKILFWWFTGLVVKGYRTPLEAEDLWTLRKEDTSHKIISELQQDWTDECAKLQKQQKA-----------------LASGAALGSRLPDQAQLLRKLQKEQS : 297
Gmo_6 : ASFLSKILFWWFTG------------------------------------------------------------------ASGTALGAKLPDQAQLLRKLQREQS : 239
Dre_6a : ASFLSKILFWWFSGLIFKGYRSPLQAEDLWSLREEDTSERIISDLEEEWTAKRTKLQQQENH-----------------MSTSAALGSRLPDQAQLLRKIQKEQS : 296
Dre_6b1 : ASFLSKLLFWWYGRLVVKGYRSPLKAEDLWSLREEDTSEKIICDLEKEWAKQWAKLQQKKSS-----------------LNEAQTLGFKLSSVKQLFRKLFKEQC : 257
Dre_6b2 : --------------------------------------------------------------------------------------------------------- : -
Ame_6a : ASFLSKLLFWWFRGLVVKGYRTPLQAEDLWSLREEDTSDKIISDLEEEWMAERTKLQQIR---------------QETYLSSSVALGSRLPDQAQLLRKLQKEQS : 298
Ame_6b : ASFLSKILFWWFSGLVVKGYRSPLKAEDLWSLRKEDTSEKIIGDLEREWTTQCAKLQQKMSY-STTQLPYHTK--QQERGDSVTALGCRITEQTQLLHKLRQEQS : 309
Loc_6 : ASFLSKILFWWFSGLVVKGYRKPLKAEDLWSLREEDRSDRIISDLEREWTAQCTKLQQQE-------------------KSLSALPGSKLTEQAQLLRKLHREQS : 296
Hsa_1 : ASFLSRITFWWITGLIVRGYRQPLEGSDLWSLNKEDTSEQVVPVLVKNWKKECAKTRKQPVK-VVYS-SKDPA--QP--KESSKVDANEEVE-ALIVKSPQKEWN : 308
Ptr_1 : ASFLSRITFWWITGLIVRGYRQPLEGSDLWSLNKEDTSEQVVPVLVKNWKKECAKTRK----------------------------------------------- : 245
Ggo_1 : ASFLSRITFWWITGLIVRGYRQPLEDSDLWSLNKEDTSEQVVPVLVKNWKKECAKTRKQPVT-VVYS-SKDPA--QP--KESSKVDANEEVE-ALIVKSPQKEWN : 281
Mmu_1 : ASFLSRITFWWITGMMVHGYRQPLESSDLWSLNKEDTSEEVVPVLVNNWKKECDKSRKQPVR-IVYAPPKDPS--KP--KGSSQLDVNEEVE-ALIVKSPHKDRE : 309
Cluf_1 : ASFLSRVTFWWITGLMVRGYRQPLESTDLWSLNKEDTSEQVVPVLVKNWKKECAKSKRQQRK-ITYS-SKDPA--KP--KGGSQVDVNEEAE-VLIVKTPQKERE : 308
Bta_1 : ASFLSRITFWWITGMMVQGYRQPLESTDLWSLNKEDTSEQVVPVLVKNWKKECAKSRKQPVK-IVYS-SKDPA--KP--KGSSKVDVNEEAE-ALIVKCPQKERD : 294
Dno_1 : ASFLSRITFWWITGLVVRGYRQPLESTHLWSLNREDTSEEVVPVLVKNWKKECARSRKQPVK-IVYP-AKDPA--KP--KGGSKVDVNEEVE-ALIVRSPQKERD : 308
Mdo_1 : ASFLSRITFWWISGLMVQGYKCPLEATDLWSLNREDTSNQVVPVLVKNWKKECAKTRKQPVK-IVYS-PKDPA--KA--EGGSKGDVNEEVE-ALILKPTQRERK : 308
Oan_1 : ---------------MVQGYKRPLEASDLWSLNREDTSDQVVPVLVKNWAKECTKSKKQSLK-IVYA-PKDPV--KL--KTGSKGDVNEEVE-ALIVKPSQRDKE : 83
Gga_1 : ASFLSRITFWWITGLMIQGHRRPLEAKDLWSLNKEDTSEEIVPGLAKNWAKEWAKTKRQPLN-MLYSSKKQQK------SSDSNGEVTEEAE-ALIIKPSQRSSE : 307
Aca_1 : TNVLKSMVLWEIKGLLLSGYQNILEMAFVFHLNSSLPSEELVVVKVKDCKWDCVKKRKRWCKWMLYSPKKAPK--GG--VSSTEAMAAEEAE-ALILRPAQRDKK : 296
Xtr_1 : ASFLSQITFWWISRMMVQGFKRPLEAKDLWSLNKEDKSLEVVPVLSKNWEKEYAKAKKVPLK-MVYS-PKKQT--K---KLNSKEDVREEAE-ALIVKPAPKELE : 307
Lch_1 : TSFLSRITFWWITGMMIQGYKQPLEAKDMWSLNKEDTSAQVVPLLDRQWQKETAKTKKGGLQ-LMYS-PKKPK--SS---KDEDGEAVEEAQ-TLIIKRAEKDKE : 307
Tni_1 : ASFLSRITFWWITNFIITHFIANILKKSFF-----------LSSVVREQKKNISKVLLHS--------------------NRKEDRMVEESE-ILIVKKQAKTKE : 283
Gac_1 : ANFLSRITFWWITRLMVTGYRRPLEEKDLWSLNSADRSHKVVPELVGRWNVECQKVKRTEQR-TLYSPREAPP---------SEGKEGRAVESEVLIVKAQKAKE : 305
Dla_1 : ASFLSRITFWWITNMMMTGYKHPLEEKDLWSLNPEDRSHRVVPQLVCRWNAECQKVKRAEEK-TLYS-PKQVP--HS---EDKDGRAVEESE-ILIVKNPQKTKE : 250
Oni_1 : ASFLSRITFWWITRMMMTGYRRPLEEKDLWSLNAEDCSHRVVPQLVKRWNTQCQKFKRSEDK-MLYS-SKRVP--HS---ENPQGQAVEESE-ILILRPRKKNKE : 307
Ola_1 : ASFLSRITFWWISGMMLSGYKRPLEEKDLWSLNPEDRSHCVVPQLLRRWTAECHKVKRTEEK-MLYS-PKQPP--HG---ENKEVRAVEESE-ILIVKSPKKSRE : 307
Xma_1 : ASFLSRITFWWITGMMMVGYRRPLEEKDLWSLNPDDCSHRVVPQLVHRWNSECQKVKRTEQQ-MVYS-PKRAL--HG---ENREGQPVEESE-ILLLTSPRKTKE : 267
Dre_1 : ASFLSKITFWWITGLMVKGYKRPLEEKDLWSLNNEDKSERVVPQLVRRWDQECVKVKRPVDK-TLYS-PKRST--RG---EKKDGQPVEESE-ILLAKALQKTGE : 307
Ame_1 : ASFLSRISFWWITGLMVQGYKRPLEEKDLWSLNAEDRSHTVVPQLVRRWDHECSKGKRTDDK-TMYS-PKRPS--MA---EKKDGRPVEESE-ILLIKAPQKSGD : 307
Loc_1 : ASFLSRITFWWITGLMVQGYRRPLEERDLWSLNEEDKSQKVVPQLVHRWNSQRAKVKRPAAK-TMYS-PKRSA--RAAAEEKKDGAPAEESE-VLIVQEPRKAQE : 310
Cmi_1 : ASFLARITFWWFTELTILGYKRPLEAKDLWSLNENDTSEKIVPELVYEWKKECSKMRKPE---TVHTQQKEKT--GA--NKEHENENTEQSE-VLLIQK-KQNME : 291
Ler_1 : --------------------------------------------------------------------------------------------------------- : -
Rty_1 : --------------------------------------------------------------------------------------------------------- : -
Sca_1 : ASFLSRITFWWFTGMTVLGYKRPLEPGDLWSLNRKDKSKTIVPQLVEEWEKECAKNTRQGE--MVYSPKKNRT-------KKQHEDTGEPDETHVFILKKKKNLQ : 192


 320 * 340 * 360 * 380 * 400 * 420
Hsa_6 : P---LLKAIWQVFHSTFLLGTLSLIISDVFRFTVPKLLSLFLEFIGDPKPPAWKGYLLAVLMFLSACLQTLFEQQNMYRLKVLQMRLRSAITGLVYRKVLALSSG : 399
Ptr_6 : P---LLKAIWQVFHSTFLLGTLSLIISDVFRFTVPKLLSLFLEFIGDPKPPAWKGYLLAVLMFLSACLQTLFEQQNMYRLKVLQMRLRSAITGLVYRKVLALSSG : 241
Ggo_6 : P---LLKAIWQVFHSTFLLGTLSLVISDVFRFTVPKLLSLFLEFIGDPKPPAWKGYLLAVLMFLSACLQTLFEQQNMYRLKVLQMRLRSAITGLVYRKVLALSSG : 394
Mmu_6 : P---LLRAIWRVFRSTFLLGTLSLVISDAFRFAVPKLLSLFLEFMGDRNSSAWTGWLLAVLMFAAACLQTLFEQQHMYRAKVLQMRLRTAITGLVYRKVLVLSSG : 391
Cluf_6 : P---LLRAIWQVSRSTFLLATFNLVICTVFRFAVPKLFSLFLEFIGNPTIPAWKGYVLAVLLFLSASLQSLLEQHYMYKLKVLQMRLRTAITGLVYRKVLVLSSA : 399
Bta_6 : P---LLRAIWQVGRSAFLLGTLSLIVSDVFRFTVPKLLSLFLEFIGDPNTPAWKGYLLAVLMFLSACLQTLFEQQHMYRLKVLQLRLRTAIIGLVYRKVLALSSS : 399
Dno_6 : P---LLRAIWQVSRVTFLLGTLSLVIGDAFRFTVPKLLSLFLEFIGDPKAPAWKGYFLAALMFLSACLQTLFEQQHVYRMKVLQIRLRTAITGLVYRKVLALSSS : 327
Mdo_6 : P---LLKAIWKVFNGTFLLGTLSLIVCDVFRFAVPKILSFFLEFISDPEAPAWKGYFYAVLLFLSACLQTLFEQRHMYVCMVLEIRLRTAVMGLVYRKVLALSNA : 403
Oan_6 : P---LLKAIWRVFGLSFLLGSLSLVACDIFTFSIPKILSLFLEFISDLAAPGWKGYFCAVLLFLINSLKILFEQRYMYVCFVLGMRLKTALVGLVYRKVLALSSA : 341
Gga_6 : P---LLQAFWSMFGIYFLLSTLCLVICDVFLFSIPKILSLFLEFIEDQEAPSWHGYFYAFILVLLACLQTLFEQRYMYMCLVLGLRLKTAVTGLVYRKILTVSNA : 405
Aca_6 : L---LLKSFWSVFGTYFILGTLCLVAGDVFLFLIPKTLSVFLDFISAPEAPSWKGYFYAAAMFLLACLQTLFEQQYMYMCLVLGVRLKTAITGLVYRKLLVMSNA : 338
Xtr_6 : QKT-LLKVIMRSFGLYFLLSALLMTFYTAFLFISPLLVRLLLQLLKDPSAPSWQGFLVAVFLFICPCCQSLFLHQHDYICYVIGMRLRAAIVGTVYKKALMISSA : 376
Lch_6 : NGTALLKALWRIYGNHFLLGTLCLVLSDVLLFSIPQILDSLLGFMSDPEAPVWRGYFYAALMFLFACLQSLLVHQYMYMACIIGMRLKTALTGLIYRKILVMTSG : 408
Tn1_6 : SGFFLLRTLTRKFGPYFLSGTLCIIFHDAFMFAIPQVLSLLLGFMRDEDAPLWKGYFYATLMFLLSCLQSLFNHQYMYTCFTVGMRVKTAVMGLVYRKSLVINSA : 410
Gac_6a : GQKGLLHTVACSFGPYFLCGTLWLLLHEVFMFAVPQVLSLLLAFISDEDAAMWKGFLFCSLLFLLSCLQSLLHHQYMFHCFSVGMRLKTALIGLVYRKCLLLSSA : 412
Gac_6b : SGFFLLRTMARKFGPYFLTGTLYIIFHDAFMFAIPQVLSLLLDFMRDEDAPLWKGYFYATLMFLLSCLQSLFNHQYMYTCFTVGMRVKTAVMGLVYRKSLVINSS : 410
Dla_6 : SGFFLLRTLARKFGPYFLTGTLCIIFHDAFMFAIPQVLSLLLGFMRDEDAPLWKGYFYATLMFLLSCLQSLFNHQYMYTCFTVGMRVKTAVMGLVYRKSLVINSA : 402
Oni_6 : HQ--LYLTLARKFGPYFLTGTLCIIFHDAFMFAIPQVLSLLLDFMRDEDAPLWKGYFYATLMFLLSCLQSLFNHQYMYTCFTVGMRVKTAVMGLVYRKSLVINSS : 410
Ola_6 : SGFFLLRTLARKFGPYFLTGTLCIIFHDAFMFAIPQVLSLLLGFIRDPEAPQWKGYFYATLMFLLSCLQSLFNHQYMYTCFTVGMRVKTAVMGLVYRKSLVINSA : 402
Xma_6 : SGFFLLRTLARKFGPYFLTGTLCIIFHDAFMFAIPQVLSLLLGFMRDEDAPLWKGYFYATLMFLLSCLQSLFNHQYMYTCFTVGMRVKTAVMGLVYRKSLVINSS : 402
Gmo_6 : SGFFLLRTLTRKFGPYFLTGTLCIVFHDAFMFAIPQVLSLLLGFMRDDEAPLWKGYFYATLIFLLSCLQSLFNHQYMYTCFTVGMRVKTAVMGLVYRKSLVINSS : 344
Dre_6a : SGFCLLRTLARSFGPYFLTGTLCLIVHDVFMFSVPQVLSLLLGFMKDEDTPLWKGYFYATLMFLLSCLQSVFNHQYTYTCFTVGMRVKTAVMGLVYRKSLVMNSS : 401
Dre_6b1 : TGFVLFRTLAKIFSPYFLTGTLFLVIQDALMFSIPQVLSLLLGYVRDEDAPLWKGYLFAFSLFLLSCLQSLFNHQYMYTCLTVGMRVKTAVMGLVYRKSLVINSA : 362
Dre_6b2 : --------------------------------------------------------------------------------------------------------- : -
Ame_6a : SGFCLLRTLARNFGPCFLSGTLCLIVHDAFMFAIPQVLSLLLGFMREEDAPLWKGYFYASLMFLLSCLQSLFNHQYMYSCFTVGMRVKTAVMGLVYRKSLVINSA : 403
Ame_6b : SGFLLLRTLAKTFGPFFLTGTLCLIIHDAFMFSIPQVLSLLLSFVGDKDVPLWKGYLFAFTMFLLSCLQSLFNHQYMYSCFTVGMRVKTAVMGLVYRKALVISSA : 414
Loc_6 : SGFSLLRTLARSFGPYFLTGTLCLIVHDAFMFSIPQVLSFLLGFMRDKDAPLWKGYFYAALMFLLSCLQSLFNHQYMYSCFTVGMRVKTAVMGLVYRKSLVINSA : 401
Hsa_1 : PS--LFKVLYKTFGPYFLMSFFFKAIHDLMMFSGPQILKLLIKFVNDTKAPDWQGYFYTVLLFVTACLQTLVLHQYFHICFVSGMRIKTAVIGAVYRKALVITNS : 411
Ptr_1 : ---------------------------------------LLIKFVNDTKAPDWQGYFYTVLLFVTACLQTLVLHQYFHICFVSGMRIKTAVIGAVYRKALVITNS : 311
Ggo_1 : PS--LFKVLYKTFGPYFLMSFFFKAVHDLMMFSGPQILKLLIKFVNDTKAPDWQGYFYSILLFVTACLQTLVLHQYFHICFVSGMRIKTAVIGVVYRKALVITSS : 384
Mmu_1 : PS--LFKVLYKTFGPYFLMSFLYKALHDLMMFAGPKILELIINFVNDREAPDWQGYFYTALLFVSACLQTLALHQYFHICFVSGMRIKTAVVGAVYRKALLITNA : 412
Cluf_1 : PS--LFKVLYKTFGPYFLMSFLFKALHDLMMFAGPEILKLLINFVNDKKAPDWQGYLYTALLFICACLQTLVLHQYFHICFVSGMRIKTAVIGAVYRKALVITNS : 411
Bta_1 : PS--LFKVLYKTFGPYFLMSFLFKAVHDLMMFAGPEILKLLINFVNDKKAPEWQGYFYTALLFISACLQTLVLHQYFHICFVSGMRIKTAVIGAVYRKALVITNA : 397
Dno_1 : PS--LFKVLYKTFGPYFLMSFLFKALHDLMMFAGPELLKLLISFVNDKQAPSWQGYFYTALLFISACLQTLVLHQYFHICFVSGMRFRTCSIGVTWEPALVISNA : 411
Mdo_1 : PS--LFKVLYKTFGPYFLMSFLFKALHDLMMFAGPEILKLLINFVNDNQAPDWQGYFYTALLFVSACLQTLVLHQYFHICFVSGMRIKTAVIGAIYRKALVITNS : 411
Oan_1 : PS--LFKVLYKTFGPYFLMSFLFKALHDLMMFAGPEILKLLINFVNDKDAPDWQGYLYTGLLFVSACLQTLVLHQYFHICFVSGMRIKTAVIGAIYRKALVITNS : 186
Gga_1 : AS--LSKVLYKTFGPYFLMSFLFKAAHDLLMFTGPEILKLLINFVNNKSAPNWQGYFYTGLLFVCACLQTLILHQYFHICFVTGMRLKTAIVGVIYRKALVITNS : 410
Aca_1 : PS--LSKVLYKTFGPYFLMSFLFKAFHDLMMFAGPEILKRLIIFVSDQSAPNWQGYFYTALLFLSAGLQTLVLHQYFHICFVTGMRLKTAVIGAIYRKALVITHS : 399
Xtr_1 : PS--LLKALYKTFGPYFFISCFFKFFHDVLMFSGPQLLQLLIKFVGDKDAPDWHGYLYTFLLFFCACLQTLILHKYFHICFVTGMRLKTAVVGLVYRKALVINNS : 410
Lch_1 : PS--LFKALCRTFGPYFLMSFVYKAIHDLLMFVGPEILRLLIQFVNNKDAPNWHGYIYTALLFVSALMQTVVLHQYFHICFVTGMRLKTSVIGTVYRKALVITHA : 410
Tni_1 : PS--LFWALCLTFGPYFLISCLYKLIQDVLMFIGPEILRLLINFVNNPEAPSWQGYFYTSLLFVCTTVQSLVLQKYFHVCFVSGMRLRTAIIGAVYRKALVIGST : 386
Gac_1 : PS--LFWALCLTFGPYFLISCLYKIIQDILMFVGPEILRLLIRFVNNSSAPSWHGYFYTALLFICTCVQSLILQKYFHVCFVSGMRLRTAIIGAVYRKALVISSE : 408
Dla_1 : PS--LLWALCLTFGPYFLISCLYKIIQDILMFVGPEILRLLIRFVNNSSAPSWQGYFYTALLFICTCVQSLILQKYFHVCFVSGMRLRTAIIGAVYRKALVISSA : 353
Oni_1 : PS--LLWALCLTFGPYFFISCIYKLIQDILMFVGPEILRLLIQFVNDSSAPSWQGYFYAALLFICTSVQSLILQKYFHVCFVSGMRLRTAIIGAVYRKALVISSA : 410
Ola_1 : PS--LLWALCLTFAPHFLVSCLYKLIQDILMFVGPEILRLLILFVNDPDAPSWQGYFYAALLFVCTCVQSLILQRYFHVCFVSGMRLRTAVIGAIYRKALVISSA : 410
Xma_1 : PS--LLWALCLTFGPYFLISCLYKIIQDILMFVGPEILRLLIHFVNDTSALSWQGYFYTALLFICTCVQSLILQRYFHVCFVSGMRLRTAIIGAVYRKALVISNG : 370
Dre_1 : PS--LFFALCRTFGPYFLVSSLYKIIHDVLMFVGPEILRLLILFVNDSSAPTWHGYFYTALLFVCTCLQTLILQKYFHVCFVTGMRLRTAIVGAVYRKALVITNA : 410
Ame_1 : PS--LFLALCRTFGPYFLVSSIYKIIHDVLMFVGPEILRLLIQFVNDSDAPSWHGYFYTALLFVCTCFQTLILQKYFHVCFVTGMRLRTAIVGAVYRKALVITNA : 410
Loc_1 : PS--LAMALCLAFGPHFLVSFVYKIIHDVLMFVGPEILKLLILFVNDPSAPSWQGYSYTALLFVCACLQTLILQQYFHVCFVTGMRLRTAIVGAVYRKALVITNA : 413
Cmi_1 : PS--LLKALCRAFGPYLMISFFFKIFHDVLVFASPEIMRLLLGFVNNHFAPVWQGYFYAILLFICAFVQTLFLHQYFHICFVTGMRLKTAIIGAVYRKALVITNE : 394
Ler_1 : --------------------------------------------------------------------------------------------------------- : -
Rty_1 : --------------------------------------------------------------------------------------------------------- : -
Sca_1 : PS--LLKALCRVFGPYFMMSFLYKIIHDLLMFASPEILKLLLQFINNENAPAWQGYFYVMLLFLCALIQTLFLHQYFQICFVTGMRLKTAIIGAVYRKALVISNA : 295


 * 440 * 460 * 480 * 500 * 520
Hsa_6 : SRKASAVGDVVNLVSVDVQRLTESVLYLNGLWLPLVWIVVCFVYLWQLLGPSALTAIAVFLSLLPLNFFISKKRNHHQEEQMRQKDSRARLTSSILRNSKTIKFH : 504
Ptr_6 : SRKASAVGDVVNLVSVDVQRLTESVLYLNGLWLPLVWIVVCFVYLWQLLGPSALTAIAVFLSLLPLNFFITKKRNHHQEEQMRQKDSRARLTSSILRNSKTIKFH : 346
Ggo_6 : SRKASAVGDVVNLVSVDVQRLTESVLYLNGLWLPLVWIVVCFVYLWQLLGPSALTAIAVFLSLLPLNFFITKKRNHHQEEQMRQKDSRARLTSSILRNSKTIKFH : 499
Mmu_6 : SRKSSAAGDVVNLVSVDIQRLAESIIYLNGLWLLFLWIFVCFVYLWQLLGPSALTAVAVFLSLLPLNFFITKKRGFHQEEQMRQKASRARLTSSMLRTVRTIKSH : 496
Cluf_6 : SRKASAVGDVVNLVSVDVQRLTECIIYLNGLWLPVIWMIICFVYLWQLLGPSALTAIAVFMSLLPLNFFITKKRKQHQEEHMRQKDSRVRLTSCIIRNMKMVKSH : 504
Bta_6 : SRKSSAVGDVVNLVSVDVQRLTESVTYLNGLWLPLIWIVVCFVYLWQLLGPSALTAIAVFVSLLPLNFFITKKRNHHQQEQMRQKDCRARLTSCILRNVRTVKYH : 504
Dno_6 : SRKASAVGDVVNLVSVDVQRLMDSIFFLNGLWLPLVWIIICFVFLWQLLGPSALTAVAVFLSLLPLNFFITKKRKHHQEEQMRQKDARARLTSSILRHAKLIKFH : 432
Mdo_6 : MRKTAAVGEIINLVSVDVQRLMDAVLYLNGLWLPVIWIIICFTFLWQLLGPSALTAIAVFLILLPLNFIITKKRSCFQEEQMQHKDRRARLTDSILRNMKIIKFH : 508
Oan_6 : ARKATAVGEIVNLVSVDVQRLVDAVVYFNGIWICPIWIVICFIFLWQLLGPSALTALAVFLFLLPLNFVITKKRSRFQEEQMRQKDHRGTLTSSILSNVRIIKFH : 446
Gga_6 : SRKAVTVGEIVNLVSVDVQKLMDLIIYFNGTWLAPIRIIICFVFLWQLLGPSALASIAVFLFL-PLNFMITKKRSHFQEAQMKHKDERATLTNAILSDIKVIKLY : 509
Aca_6 : AKKEATVGEIVNLVSVDVQKLMDLIIYFNGTWLAPIRIVICFVFLWQLLGPSALMAVVVFLFLLPLNFVIAKKRTQFQEAQMAHKDSRAKLTSAILSDIKTLKLH : 443
Xtr_6 : GRKESSAGEIVNLISTDVQKLMDLATCVNYMWSAPVTIIVAMYFLWQTLGIAVLAGVAVFILNLPFMTVFAVIIKRVQEQQMKQKDGRIKIISEILQGIKVLKLY : 481
Lch_6 : AKKTSTVGEVVNLVSVDIQKLMDLIIYFNGVWLAPLEIALCFYFLWQYLGPSSLAGVVAIFVVFSLNGVIAKKRSKFQEEQMRCKDERVKHTYQLLGGIKVLKFH : 513
Tn1_6 : SRRTCTVGEIVNLVSADTQKLMDFVVYFNAVWLAPIEIALCLFFLWQQLGPSALAGIATVIFIFPLNGFIAKKRSKLQEIQMKFMDGRIRLMNEILNGIKILKFY : 515
Gac_6a : ARRRGDVGEIINLVSADTQKLMDFVVYFNSLWVTPIEITLCFYFLWQLLGPSALAGIVPVVLIVPLNGLIAKMRSKLQEIQMKFTDGRLKLMNEILSGVKILKFY : 517
Gac_6b : ARRTCTVGEIVNLVSADTQKLMDFVVYFNAVWLAPIEISLCLFFLWQHLGPSALAGIATVILIFPLNGLIAKKRSKLQEVQMKFMDGRIRLMNEILSGIKILKFY : 515
Dla_6 : ARRTCTVGEIVNLVSADTQKLMDFVVYFNAVWLAPIEIALCLFFLWQHLGPSALAGIATVILIFPLNGFIAKKRSKLQEIQMKFMDGRIRLMNEILNGIKILKFY : 507
Oni_6 : ARRTCTVGEIVNLVSADTQKLMDFVVYFNAVWLAPIEIALCLFFLWQHLGPSALAGIATVILIFPLNGFIAKKRSKLQEIQMKFMDGRIRLMNEILNGIKILKFY : 515
Ola_6 : ARRTCTVGEIVNLVSADTQKLMDFVVYFNAVWLAPIEIGLCLFFLWQHLGPSALAGIATVILIFPLNGFIAKKRSKLQEIQMKFMDGRVRLMNEILNGIKILKFY : 507
Xma_6 : ARRTCTVGEIVNLVSADTQKLMDFVVYFNAVWLAPIEIALCLFFLWQHLGPSALAGITTVILIFPLNGFIAKKRSKLQEIQMKFMDGRIRLMNEILNGIKILKFY : 507
Gmo_6 : ARRTCTVGEIVNLVSADTQKLMDFVVYFNAVWLAPIEIALCLFFLWQHLGPSALAGIATVILIFPLNGYIAKKRSKLQEVQMKFMDGRIRLMNEILSGIKILKFY : 449
Dre_6a : ARRTCTVGEIVNLVSADTQKLMDFVVYFNAVWLAPIEVTLCLFFLWQHLGPSALAGIATVIFIFPLNGFIARKRSKLQEIQMKYMDGRVKLMNEILNGIKILKFY : 506
Dre_6b1 : ARKTCTVGEIVNLVSADTQKLVDFVMYFNALWLAPIEIALCLFFLWQHLGPSTLAGITTVILIFPLNGFIAKMRSKLQEVQMKHKDERIKLMNEILSGIKILKFY : 467
Dre_6b2 : --------------------------------------------------------------------------------------------------------- : -
Ame_6a : ARRTCTVGEIVNLVSADTQKLMDFVVYFNAVWLAPIEIALCLFFLWQHLGPSALAGIATVILIFPLNGFIAKKRSKLQEIQMKFMDGRIKLMNEILNGIKILKFY : 508
Ame_6b : ARRTCTVGEIVNLVSADTQKLMDFVVYFNAVWLAPIEIGLCLFFLWQRLGPSALAGITTVILIFPLNGLIAKMRSKLQEVQMKYMDGRIKLMTEILSGIKILKFY : 519
Loc_6 : ARRTCTVGEIVNLVSADTQKLMDFVVYFNAVWLAPIEIGLCLFFLWQHLGPSALAGIATVILIFPLNGFIAKQRSKLQEVQMKHMDGRIKLMNEILNGIKIIKFY : 506
Hsa_1 : ARKSSTVGEIVNLMSVDAQRFMDLATYINMIWSAPLQVILALYLLWLNLGPSVLAGVAVMVLMVPVNAVMAMKTKTYQVAHMKSKDNRIKLMNEILNGIKVLKLY : 516
Ptr_1 : ARKSSTVGEIVNLMSVDAQRFMDLATYINMIWSAPLQVILALYLLWLNLGPSVLAGVAVMVLMVPVNAVMAMKTKTYQVAHMKSKDNRIKLMNEILSGIKVLKLY : 416
Ggo_1 : ARKSSTVGEIVNLMSVDAQRFVDLATYINMIWSAPLQVIVALYLLWLNLGPSVLAGVAVMVLMVPVNAVMAMKTKTYQVRHVSLRAPKPGM-------------- : 475
Mmu_1 : ARKSSTVGEIVNLMSVDAQRFMDLATYINMIWSAPLQVILALYFLWLSLGPSVLAGVAVMILMVPLNAVMAMKTKTYQVAHMKSKDNRIKLMNEILNGIKVLKLY : 517
Cluf_1 : ARKSSTVGEIVNLMSVDAQRFMDLATYINMIWSAPLQVILALYLLWLNLGPSVLAGVAVMILMVPLNAVMAMKTKTYQVAHMKSKDNRIKLMNEILNGIKVLKLY : 516
Bta_1 : ARKSSTVGEIVNLMSVDAQRFMDLATYINMIWSAPLQVILALYLLWLNLGPSVLAGVAVMVLMVPLNAVMAMKTKTYQVAHMKSKDNRIKLMNEILNGIKVLKLY : 502
Dno_1 : ARKTSTVGEIVNLMSVDAQRFMDLATYINMIWSAPLQVVLALYLLWLNLGPSILAGVAVMILMVPINAVMAMKTKTYQVAHMKSKDNRIKLMNEILNGIKVLKLY : 516
Mdo_1 : ARKSSTVGEIVNLMSVDAQRFMDLATYINMIWSAPLQVILALYLLWLNLGPSVFAGVAVMILMVPLNAVMAMKTKTYQVAHMKSKDNRIKLMNEILNGIKVLKLY : 516
Oan_1 : ARKSSTVGEIVNLMSVDAQRFMDLATYINMIWSAPLQVILALYLLWLNLGPSVLAGVAVMILMVPINAVMAMKTKTYQVAHMKSKDNRIKLMHEILNGIKVLKLY : 291
Gga_1 : ARKTSTVGEIVNLMSVDAQRFMDLATYINMIWSAPLQVILALYLLWRNLGPSVLAGVAVMILLVPINAVMAMKTKTYQVAQMKSKDNRIKLMNEILNGIKVLKLY : 515
Aca_1 : ARKSSTVGEIVNLMSVDAQRFMDLATYINMVWSAPLQVILALYLLWQNLGPSVLAGVAVMLLLVPVNAVIAMKTKTYQVAHMKSKDNRIKLMNEILNGIKVLKLY : 504
Xtr_1 : ARRTSTIGEIVNLMSVDAQRFMDLATYINMIWSAPLQVILAFYLLWQNVGPSVLAGIAVMVVMLMLVAPCIFSAAVTSVVQMKCKDNRIKLMNEILNGIKVLKLY : 515
Lch_1 : ARKMSTVGEIVNLMSVDAQRFMDLVTYINMIWSAPLQVILALFFLWQNLGPSVLAGVAVMVLMVPVNAFIAMKSKTYQVTQMKSKDNRIKLMNEILNGIKVLKLY : 515
Tni_1 : ARRTSTVGEIVNLMSVDAQRFMDLITYINMIWSAPLQVVLALYFLWQTLGPSVLAGVGVMVLMVPVNAVIAMKTKTYQVAQMKNKDSRIKLMNEMLNSIKVLKLY : 491
Gac_1 : ARRTSTVGEIVNLMSVDAQRFMDLITYINMIWSAPLQVVLALYFLWQNLGPSVLAGVAVMVLMVPINAVIAMKTKTYQVAQMKSKDSRIKLMNEMLNGIKVLKLY : 513
Dla_1 : ARRTSTVGEIVNLMSVDAQRFMDLITYINMIWSAPLQVVLALYFLWQNLGPSVLAGVAVMVLMVPVNAVIAMKTKTYQVAQMKSKDNRIKLMNEMLNGIKVLKLY : 458
Oni_1 : ARRTSTVGEIVNLMSVDAQRFMDLITYINMIWSAPLQVVLALYFLWQNLGPSVLAGVAVMVLMVPVNAVIAMKTKAYQVAQMKSKDNRIKLMNEMLNGIKVLKLY : 515
Ola_1 : ARRSSTVGEIVNLMSVDAQRFMDLVTYINMIWSAPLQVVLALYFLWENLGPSVLAGVAVMILMVPINAVIAMKTKTYQVAQMKNKDSRIKLMNEMLNGIKVLKLY : 515
Xma_1 : ARRTSTVGEIVNLMSVDAQRFMDLVTYINMVWSAPLQVVLALYFLWQNLGPSVLAGVAVMVLMVPINAVIAMKTKTYQVAQMKSKDSRIKLMNEMLNGIKVLKLY : 475
Dre_1 : ARRTSTVGEIVNLMSVDAQRFMDLITYINMIWSAPLQVILALYFLWQNLGASVLAGVAVMVLMVPLNAVIAMKTKTYQVAQMKSKDNRIKLMNEVLNGIKVLKLY : 515
Ame_1 : ARRTSTVGEIVNLMSVDAQRFMDLITYINMIWSAPLQVILALYFLWQNLGPSVLAGVAVMVLMVPVNAVIAMKTKTYQVAQMKSKDNRIKLMNEVLNGIKVLKLY : 515
Loc_1 : ARCTSMVGEIVNLMSVDAQRFMDLVTYINMIWSAPLQVILALYFLWQNLGPSVLAGVAVMVFMVPVNAVIAMKSKTYQVAQMKSKDSRIKLMNEVLNGIKVLKLY : 518
Cmi_1 : ARKTSTIGEIVNLMSVDAQRLMDLITYINMMWSAPLQVTLAMYFLWQNLGPSVLAGVAVMILLVPINSMIAMKTKDLQVTQMKEKDNRIKLMNEILNGIKVIKLY : 499
Ler_1 : -----------------------------------------------------------------------MKTKTLQVTQMKEKDNRIKLMNEVLNGIKVIKLY : 34
Rty_1 : --------------------------------------------------------------------------------------------------------- : -
Sca_1 : ARKTSTVGEIVNLMSVDAQKFMDLITYLNMIWSAPLQVILAMYFLWQNLGPSVLAGVAVMVLLVPINGVIAVKTKNLQVTQMKEKDNRIKLMNEVLNGMRVLKLY : 400


 * 540 * 560 * 580 * 600 * 620 *
Hsa_6 : GWEGAFLDRVLGIRGQELGALRTSGLLFSVSLVSFQVSTFLVALVVFAVHTLVAE-NAMNAEKAFVTLTVLNILNKAQAFLPFSIHSLVQARVSFDRLVTFLCLE : 608
Ptr_6 : GWEGAFLERVLGIRGQELGALRTSGLLFSVSLVSFQVSTFLVALVVFAVHTLVAE-NAMDAEKAFVTLTVLNILNKAQAFLPFSIHSLVQARVSFDRLVTFLCLE : 450
Ggo_6 : GWEGAFLDRVLGIRGQELGALRTSGLLFSVSLVSFQVSTFLVALVVFAVHTLVAE-NAMDAEKAFVTLTVLNILNKAQAFLPFSIHSLVQARVSFDRLVAFLCLE : 603
Mmu_6 : GWEHAFLERLLHIRGQELSALKTSTLLFSVSLVSFQVSTFLVALVVFAVHTLVAEDNAMDAEKAFVTLTVLSILNKAQAFLPFSVHCIVQARVSFDRLAAFLCLE : 601
Cluf_6 : GWEEAFLERVLHIRGQELGAMRTSSLLFSVSLVSFQVSTFLVALVVFAVHTLVAEENAMDAEKAFVTLTVLSILNKAQVFMPFSINSVVQARVSFDRLAAFLCLE : 609
Bta_6 : GWEGAFLDRVLHIRAQELGALKTSSLLFSVSLVSFQVSTFLVALVVFAVHTLVAEENAMDAEKAFVTLTVLNILNKAQAFLPFSIHSIVQARVSFDRLAAFLSLE : 609
Dno_6 : GWEEAFLDRVLQSRRRELGALRTSGLLFSVSLVSFQASTFLVALVVFAVHTLVAEENAMDAEKAFVTLTVLNILNKAQAFLPFSIHSVVQARVSFDRLAAFLCLE : 537
Mdo_6 : GWEEAFMEKILTIRKGELQALKNSGFLFAVSLVSFHLSTFLVALVMFAVHALTDEKHVLDAEKAFVALTIINILNRAQAFLPFSINTIFQAWVSLARLAAFLHLE : 613
Oan_6 : GWEKAFMEKVLHIRKEELQALKKSGLLFSVSLVSFHLSTFLVALVMFAVYTLSDENNVLDAQKAFVALMLINILNKAQGFLPLSLHTTIQAKVSLARLAAFLSLE : 551
Gga_6 : GWEKTFMEKVHAIRKQELQALKRSQILFSASLASFHSSTFLIAFVMFAVYTLVDNTHVLDAQKAFVSLTLINILNTAHSFLPFSINAAVQAKVSLKRLAAFLNLE : 614
Aca_6 : GWEEAFVGRVMGVRTRELQALRRSQFLFSASLVSFQSSTFLISFIMFAVYTLADERNIFSAQKAFVSLALVNILNTAHSFLPFSINSVVQAKVSLNRLAAFLSLE : 548
Xtr_6 : AWENAFMKKVTEFRLMELKAVKTGALLLSGALAVFVASPFWVSLTMFGVFLALDEKNILDAEKAFVTIILLNILRIPLRMFPMAITLFAQSSVSLKRMVKFFSAE : 586
Lch_6 : AWETALMEKVLGIRQKELKVLKKSQLLFAASLASFHSSAFLISFAVFAVYMLVDRSNVLDAQKAFVSMALVTIMKIPLSFLPFSISTTVQAGVSLKRLSTFLSHE : 618
Tn1_6 : AWEKAFLEQVLGHREKELRALKKSQILYSISIASFNSSSFLIAFAMFGVYVMLDNRNVLDAQKVFVSMALINILKTPLSQLPFAISTTMQAMVSLRRLGKYLCSG : 620
Gac_6a : AWEDAFLRRIGVLRDGELETLKMSQVLHSVSLASFNSSSFLIALSVFAVYVTIDDRNLLDAQKIFVSVALINILKTPLSQLPFAMSATMQAVVSLRRLGNFLSQD : 622
Gac_6b : AWEKAFLEQVLGLREKELKALKKSQILYSISIASFNSSSFLIAFSMFGVYVMLDDRNVLDAQKVFVSMALINILKTPLSQLPFAISTTMQAMVSLRRLGKYLCSE : 620
Dla_6 : AWEKAFLEQVLGHREKELKALKKSQILYSISIASFNSSSFLIAFAMFGVYVTLDDRNVLDAQKVFVSMALINILKTPLSQLPFAISTTMQAMVSLRRLGKYLCSE : 612
Oni_6 : AWEKAFLEQVLGYREKELKALKKSQILYSISIASFNSSSFLIAFAMFGVYVMLDDKNVLDAQKVFVSMALINILKTPLSQLPFAISTTLQAVVSLKRLGKYLCSE : 620
Ola_6 : AWEKAFLEQVLGYREKELKALKKSQVLYSISIASFNSSSFLIAFAMFGVYVMLDERNVLDAQKVFVSMALINILKTPLSQLPFAISTTMQALVSLRRLGKYLCSE : 612
Xma_6 : AWEKAFLEQVLGYREKELKALKKSQILYSISIASFNSSSFLIAFAMFGVYVMLDDRNVLDAQKVFVSMALINILKTPLSQLPFAISTTMQALVSLRRLGKYLCSE : 612
Gmo_6 : AWEKAFLEQVLGYREKELQALKKSQVLYSISFASFNCSSFLIAFAMFGVYVTIDARNVLDAQKVFVSMALINILKTPLSQLPFVMSTTMQ-------LGKYLCSE : 547
Dre_6a : AWEKAFLEQVLGYREKELKTLKKSQILYSVSLASFNSSSFLIAFAMFGVYVLIDDKNVLDAQKIFVSMALINILKTPLSQLPFAMSTTMQALVSLKRLGKFLCQD : 611
Dre_6b1 : AWEKAFRERVLGYREKELNALKKSQILYSVSIASFNSSTLLIAFAMFGVYVLIDDKHVLDAQKIFVSMALINILKAPLSQLPIAMSTTMQVVVSLKRLGTFLDQD : 572
Dre_6b2 : --------------------------------------------------------------------------------------------------------- : -
Ame_6a : AWEKAYLEKVLGYREKELGALRKSQILYSISIASFNSSTFLIAFAMFGVYVLIDDKNVLDAQKIFVSMALINILKTPLSQLPFAMSTTMQAVVSLKRLGKFLCQE : 613
Ame_6b : AWEKAFEERVLGYREKELKALKKSQILYSISIASFNSSTFLIAFAMFGVYVLIDEKNVLDAQKVFVSMALINILKTPLSQLPFAMSTTMQAIVSLKRLGKFLCQD : 624
Loc_6 : AWEKAFLERVLGYRQKELKALKKSQILYSISIASFNSSTFLIAFAMFGVYVLIDEKNVLDAQKVFVSMALINILKTPLSQLPFAMSTTMQAFVSLKRLGKFLCQE : 611
Hsa_1 : AWELAFKDKVLAIRQEELKVLKKSAYLSAVGTFTWVCTPFLVALCTFAVYVTIDENNILDAQTAFVSLALFNILRFPLNILPMVISSIVQASVSLKRLRIFLSHE : 621
Ptr_1 : AWELAFKDKVLAIRQEELKVLKKSAYLSAVGTFTWVCTPFLVALCTFAVYVTIDENNILDAQTAFVSLALFNILRFPLNILPMVISSIVQASVSLKRLRIFLSHE : 521
Ggo_1 : -------------------------CVCDIITISHWAFCLQVALCTFAVYVTIDENNILDAQTAFVSLALFNILRFPLNILPMVISSIV---VSLKRLRIFLSHE : 552
Mmu_1 : AWELAFQDKVMSIRQEELKVLKKSAYLAAVGTFTWVCTPFLVALSTFAVFVTVDERNILDAKKAFVSLALFNILRFPLNILPMVISSIVQASVSLKRLRIFLSHE : 622
Cluf_1 : AWELAFKDKVLAIRQEELKVLKKSAYLAAVGTFTWVCTPFLVALSTFAVYVTVDKNNILDAQKAFVSLALFNILRFPLNILPMVISSIVQASVSLKRLRIFLSHE : 621
Bta_1 : AWELAFKDKVLAIRQEELKVLKKSAYLAAVGTFTWVCTPFLVALSTFAVYVTVDENNILDAQKAFVSLALFNILRFPLNILPMVISSIVQASVSLKRLRVFLSHE : 607
Dno_1 : AWELAFQEKVSAIRKEELKVLKKSAYLAAVGTFTWVCTPFLVALSTFAVYVTVDENNILDAQKAFVSLALFNILRFPLNILPMVISSIVQASVSLKRLRIFLSHE : 621
Mdo_1 : AWELAFKEKVLEIRQEELKVLKKSAYLAAVGTFTWVCTPFLVALSTFAVYVTVDKNNVLDAQKAFVSLALFNILRFPLNILPMVISSIVQASVSLKRLRIFLSHE : 621
Oan_1 : AWELAFKKKVLEIRQEELKVLKKSAYLAAVGTFTWVCTPFLVALSTFAVYMTIDENNILDAQKAFVSLALFNILRFPLNILPMVISSIVQASVSLKRLRIFLSHE : 396
Gga_1 : AWELAFREKVLEIRQKELKVLKKSAYLAAMGTFTWVCAPFLVALSTFAVYVKVNKNNILDAQKAFVSLALFNILRFPLNILPMVISSIVEASVSLKRLRVFLSHE : 620
Aca_1 : AWELAFKEKVLGIRKEELRVLKKSAYLAAIGTFTWVCAPFLLAKCANCVFIYIDYKNVLDNPERWISETLLYSLK-SISHISLLKFKRQHASTSTESVSVYLSYD : 608
Xtr_1 : AWELAFKEKVLGIRKDELKVLKKSAYLAAVGTFTWVCAPFLVALSTFAVYVLIDKQNVLDAEKAFVSLALFNILRFPLNMLPMVISSMVQASVSLKRLRVFLSHE : 620
Lch_1 : AWELAFKDKVLEIRQQELQVLKKAAYLAVVATFTWVCTPFLVALSTFTVYVLVDENNVLDAQKAFVSLALFNILRFPLNMLPMVISSIVQASVSLKRLRVFLSHE : 620
Tni_1 : AWELAFKDKVSEIREHELHVLKKAGYLGAVTTFTWICAPFLVALSTFTVYVLMDENNVLDAQKAFVSLALFNILRFPLTMLPMVIK-ICRINWKLKTAMTLCSLQ : 595
Gac_1 : AWELAFKDKVSKIRESELQVLKKAAYLGAVSTFTWICAPFLVALSTFAVYVLIDEHNVLDAQKAFVSLALFNILRFPLNMLPMVISSMVQASVSLKRLRVFLSHA : 618
Dla_1 : AWELAFKDKVSEIRESELRVLKKAAYLGAMSTFTWVCAPFLVALSTFTVYVLIDEHNVLDAQKAFVSLALFNILRFPLNMLPMVISSMVQASVSLKRLRVFLSHE : 563
Oni_1 : AWELAFKGKVSEIRESELRVLKKAAYLGAVSTFTWVCAPFLVALSTFAVYVLIDEQNVLDAQKAFVSLALFNILRFPLNMLPMVISSMVQASVSLKRLRVFLSHE : 620
Ola_1 : AWEMAFKEKVSQIRENELKVLKKAAYLGAVSTFTWVCAPFLVALSTFSVYVLIDDQNVLDAEKAFVSLALFNILRFPLNMLPMVISSMVQASVSLKRLRVFLSHE : 620
Xma_1 : AWELAFKEKVSKIRESELRVLKKTAYLGAISTFTWVCAPFLVALSTFAVYVLIDDQNVLDAQKAFVSLALFNILRFPLNMLPMVISSIVQV-------------- : 566
Dre_1 : AWELAFKGKVSAIRESELRVLKKMAYLGAISTFTWVCAPFLVALSTFAVYVLVDENNILDAQKAFVSLALFNILRFPLNMLPMVISSMVQASVSMQRLRVFLSHE : 620
Ame_1 : AWELAFKDKVSTIRESELRVLKKAAYLGAISTFTWVCAPFLVALSTFAVYVLVDEHNVLDAQKAFVSLALFNILRFPLNMLPMVISSMVQASVSMKRLRVFLSHE : 620
Loc_1 : AWELAFQGKVLGIRETELRVLKKSAYLAAVSTFTWVCTPFLVALSTFAVYVLVDERNVLDAQKAFVSLALFNILRFPLNMLPMLISSMVQASVSLKRLRVFLSHE : 623
Cmi_1 : AWELAFKEKVMQIRRKELKVLKNAAYFSAVSTFTWICAPFLVALSSFAVYVLVNEHNVLDAQKAFVSLALFNILRFPLNMLPVVISSLVQASVSLKRLRLFLSHE : 604
Ler_1 : AWELAFKEKVLDIRENELKVLKRAAYLAAVSTFTWVCAPFLVALCTFGVYVLVDEKNVLDAQKAFVSLALFNILRFPLNMLPMVISSLVQASVSLDRLETFLSHD : 139
Rty_1 : -------------------------------------------------------------------------------------PNFLQANVSLKRLGTFLSHE : 20
Sca_1 : AWELAFKEKVLQIRQKELQVLKSAAYLSAVSTFTWVCAPFLVALSTFAVYVLVDENNVLDAQKAFVSLALFNILRFPLNMLPMVISSLVQAGVSLKRLDTFLSHE : 505


 640 * 660 * 680 * 700 * 720 *
Hsa_6 : EVDPGVVDSSSSGSAAGKDCITIHSATFAWSQESPPCLHRINLTVPQGCLLAVVGPVGAGKSSLLSALLGELSKVEGFVSIEGAVAYVPQEAWVQNTSVVENVCF : 713
Ptr_6 : EVDPGAVDSSSSGSTAGKDCITIHSATFAWSQESPPCLHRINLTVPQGCLLAVVGPVGAGKSSLLSALLGELSKVEGFVSIEGAVAYVPQEAWVQNTSVVENVCF : 555
Ggo_6 : EVDPGAVDSSSSGSAAGKDCITIHSATFAWSQESPACLHRINLTVPQGCLLAVVGPVGAGKSSLLSALHGELSKVEGFVSIEGAVAYVPQEAWVQNTSVVENVCF : 708
Mmu_6 : EVDPNGMIASNSR-RSSKDRISVHNGTFAWSQESPPCLHGINLTVPQGCLLAVVGPVGAGKSSLLSALLGELLKVEGSVSIEGSVAYVPQEAWVQNTSVVENVCF : 705
Cluf_6 : ELDLRAVDLSPSRCSAGETCIRVHDGTFAWSREGTPCLRRINLTVPQGRLLAVVGAVGSGKSSLLSALLGELSKVEGSVSIKGSVAYVPQEAWVQNTSVVENVCF : 714
Bta_6 : ETDPGAVDSSPSRCAAGEDCISIQEGTFTWSQESAPCLRRINLTVPQGCLLAVVGPVGAGKSSLLSALLGELSKVEGSVSIKGPVAYVPQEAWVQNMSVVDNVCF : 714
Dno_6 : EVDPGAVVSMPFRCPAGKAGITVRNGTFAWSRESPPCLQRITLTVPQGCLLAVVGPVGAGKSSLLSALLGELSKVEGSVSIKGPVAYVPQEAWVQNASVLDNVCF : 642
Mdo_6 : EVEPRAISTTPV----GEESISVQDGTFAWSQENSPCLQRINLVVPRGSFFAVTGPVGSGKSSLLSAILGELTKLEGNVNIKGSVAYVPQEAWIQNASVEENVCF : 714
Oan_6 : EIEPNAVDTSPK--GSSGECITIENGTFAWSRESSPCLRRISLAVPRGHLLAVIGSVGAGKSSLLAALLGELSKLDGHVNVEGSVAYVPQEAWVQNASVEENVCF : 654
Gga_6 : ELNPESSNRHTS--DCGELFIIIRNGTFCWSKDTSPCLRRIDLTVPQGSLLAVVGQVGAGKSSLLSALLGDLEKMDGCVTMKGTAAYVPQQAWIQNASVEDNILF : 717
Aca_6 : DLDQTNAEPGSL--DGTQDCITIRNGTFTWSRESPPCLKRINLSIARGSLCAVIGQVGSGKSSLLSALLGELQKTEGSLALKGTVAFVPQESWIQNASVEENITF : 651
Xtr_6 : ELEPESVDINDSLSSNLEHAITIRHGTFTWSSSEPPCLQSINIGIPQGTLVAVVGQVGCGKTSLLSALLGEMEKVEGQVSLMGSVAYVPQQTWIPNATFKENVLF : 691
Lch_6 : ELNPNNTDKSTT--HSLGKQIVVENGTFSWSKDNPPCLTRINLTIPEGALVAVVGHVGCGKSSLLSALLGELEKQEGYVAIKGSVPYVPQQAWIQNATLKDNILF : 721
Tn1_6 : ELKADNVSKAPR--TSDGENVVIENGTFSWSAAGPPCLKRINVHVPRGSLVAVVGPVGSGKSSLLSAMLGETEKRSGQVTVKGSVAYVPQQAWIQNATVQDNIVF : 723
Gac_6a : ELKGDSVERLPR--SSDGDAVRIEDGWFSWTCDGPPCLQGISVKVKPGSLVAVVGHVGSGKSSLLSAMLGEMERRSGSISIKGSVAYVPQQAWIQNASLKDNILF : 725
Gac_6b : ELREDNVSKAPF--CSDGEDVVIENGNFSWSAEGPPCLKRISIHVPRGSLVAVVGHVGSGKSSLLSAMLGETEKRSGCVTVKGSVAYVPQQAWIQNATVQDNILF : 723
Dla_6 : ELRVDNVSKAPL--SSDGEDVAIENGTFSWSAEGPPCLKRINIHVPRGSLVAVVGHVGSGKSSLLSAMLGETEKRTGRVSVKGSVAYVPQQAWIQNATVQDNIIF : 715
Oni_6 : ELKMENVSKAPL--SSDGEDVVIENGTFSWSAEGPPCLKRISVSVPRGSLVAVVGPVGSGKSSLLSAMLGETEKRSGQVTVKGSVAYVPQQAWIQNATVQDNIIF : 723
Ola_6 : ELKVDGVSKALS--SSDGEDLVIENGTFSWSKEGPPCLKRISVRVPRGSLVAVVGHVGSGKSSLLSAMLGETEKRSGQVTVKGSVAYVPQQAWIQNATVQDNILF : 715
Xma_6 : ELRVDNVSKTLL--SPDGEDVMIESGTFSWTPEGPPCLKRINVRVPRGSLVAVVGHVGSGKSSLLSAMLGETEKRSGQVTVKGSVAYVPQQAWIQNATVQDNILF : 715
Gmo_6 : ELKGDNVTKAAY--SLDGEDVSIDNGTFSWSGEGPPCLKRINVHVPRGSLVAVVGHVGSGKSSLLSAMLGETERRTGHVTVKGSLAYVPQQAWIQNATVQDNVVF : 650
Dre_6a : ELKPDNVARESF--KSDVDGVVFDNGTFSWSKDGPPCLKRISVKVPCGSLVAVVGHVGSGKSSLLSAMLGETEKRSGTVSVKGSIAYVPQQAWIQNASLQDNILF : 714
Dre_6b1 : ELKLDSVQRVPY--NPNIESVVINNGTFSWSKDSTPCLRRINVKVQRGSLVAVVGHVGSGKSSLLSAMLGEMEKKSGHITITGSVGYVPQQAWIQNATLKDNILF : 675
Dre_6b2 : ------------------ESVVINNGTFSWSKDSTPCLRRINVKVQRGSLVAVVGHVGSGKSSLLSAMLGEMEKKSGHIKITGSVAYVPQQAWIQNATLKDNILF : 87
Ame_6a : EIKPDNVSRDPY--KQDEDSVVVENGTFSWSKDGPPCLKRISVRVPCGGLVAVVGHVGSGKSSLLSAMLGETERRSGNVSIKGSVAYVPQQAWIQNATLQDNILF : 716
Ame_6b : ELKLDSVVRAPF--SPDGDSVTIEDGTFSWSRDGPPCLRRISVKVPRGCLVAVVGHVGSGKSSLLSAMLGETEKKSGNVTVKGSVAYVPQQAWIQNATLRENIVF : 727
Loc_6 : ELKADNVERAPL--SPEGGSVVVENGTFSWTREGSPCLRRINVRVPQGALLAVVGHVGSGKSSLLSAILGETEKRSGRVLVKGSVAYVPQQAWIQNATLRENVIF : 714
Hsa_1 : ELEPDSIERRPVKDGGGTNSITVRNATFTWARSDPPTLNGITFSIPEGALVAVVGQVGCGKSSLLSALLAEMDKVEGHVAIKGSVAYVPQQAWIQNDSLRENILF : 726
Ptr_1 : ELEPDSIERRPVKDGGGTNSITVRNATFTWARSDPPTLNGITFSIPEGALVAVVGQVGCGKSSLLSALLAEMDKVEGHVAIKGSVAYVPQQAWIQNDSLRENILF : 626
Ggo_1 : ELEPDSIERRPVKDGGGTNSITVRNATFTWARSDPPTLNGITFSIPEGALVAVVGQVGCGKSSLLSALLAEMDKVEGHVAIKGSVAYVPQQAWIQNDSLRENILF : 657
Mmu_1 : ELEPDSIERRSIK-SGEGNSITVKNATFTWARGEPPTLNGITFSIPEGALVAVVGQVGCGKSSLLSALLAEMDKVEGHVTLKGSVAYVPQQAWIQNDSLRENILF : 726
Cluf_1 : ELEPDSIERRPVKDGGGANSITVKNATFTWARSDPPTLSGITFSIPEGSLVAVVGQVGCGKSSLLSALLAEMDKVEGHVAIKGSVAYVPQQAWIQNDSLRENILF : 726
Bta_1 : DLDPDSIQRRPIKDAGATNSITVKNATFTWARNDPPTLHGITFSVPEGSLVAVVGQVGCGKSSLLSALLAEMDKVEGHVTVKGSVAYVPQQAWIQNISLRENILF : 712
Dno_1 : ELEPDSIERRPVKDGGGTNSITVRNATFTWARDDSPTLSGITFSVPEGALVAVVGQVGCGKSSLLSALLAEMDKVEGHAALKGSVAYVPQQAWIQNDSLRENILF : 726
Mdo_1 : ELEPESIVRKPIK-DGGGDSIIVKNATFTWSRSDPPTLNGITFTVPQGALVAVLGQVGCGKSSLLSALLAEMDKIEGHVSIKGSVAYVPQQAWIQNASLRENVLF : 725
Oan_1 : ELEPDSVVRCSVKNAGG-NSISVTNATFTWSRNDPPTLTGITFAVPEGSLIAVVGQVGCGKSSLLSALLAEMDKVEGHVAIKGSIAYVPQQAWIQNASLRENILF : 500
Gga_1 : ELDPDSIIRGPI--TNAEGSIVVKNATFSWSKTDPPSLNSINFTVPEGSLIAVVGQVGCGKSSLLSALLGEMDKKEGYVVVKGSIAYVPQQAWIQNATLEDNIIF : 723
Aca_1 : FFEPLFLANG--------NSITVRNATFSWSRSDLPCLNNINFAVPEHRLVAVVGQVGCGKSSLLSALLGEMEKREGLVSLKGSVAYVPQQAWIQNATLKENILF : 705
Xtr_1 : ELEPESIIREPQKSSGMHNGDSIKVDVFLRA----STYHIINISIPEGSLVAVVGQVGCGKSSLLSALLGEMEKQDGYVAMKGSVGYVSQQAWIQNASLKDNVLF : 721
Lch_1 : ELDPDSVDRSSS--KGSDESITMRSGTFSWSNSDPPCLKGINLTIPEGALVAIVGHVGCGKSSMLSALLGEMEKQEGHVAVKGSVAYVPQQAWIQNATLKDNILF : 723
Tni_1 : VICFVLIERLCTPCNG--------------------CLDKNHLETNRGKFV---GLIMC-------------------VIVQGLVAYVPQQAWIQNSTLKENIVF : 658
Gac_1 : ELQEDGVDHKAA--VGTSHSVSIVDGVFSWSRAESPTLKRLNVCIPEGSLVAVVGHVGSGKSSLLSALLGEMDKLEGSVAVKGSVAYVPQQAWIQNATLRENIMF : 721
Dla_1 : ELQEDSVEHKAV--AGSPHSISIVDGVFSWSRTESPILKKLTVHIPEGSLVAVVGHVGSGKSSLLSALLGEMDKLEGTVTVKGWVAYVPQQAWIQNSTLKENIMF : 666
Oni_1 : ELQVDSVEHKAA--EGSQYSISVTDGVFTWSRTESLSLFRLNINIPEGSLVAVVGHVGSGKSSLLSALLGEMDKLEGSVTVKGSVAYVPQQAWIQNSSLKDNIIF : 723
Ola_1 : ELQEDSVERPAA--GGSPYSISIEDGVFSWSRSESPTLKRLNVRIPEGSLVAVVGHVGSGKSSLLSALLGEMDKMEGSVSVKGSVAYVPQQAWILNATLKNNIVF : 723
Xma_1 : -----------------------------------------------------------GKSSLLSALLGEMDKVEGSVVVKGSVAYVPQQAWIQNSTLKENIVF : 612
Dre_1 : ELDDDNVERPAI--SGTPDSIRIADGAFSWSKDDPPTLKRINVSIPEGALVAVVGHVGSGKSSLLSALLGEMHKQEGSVSIKGSVAYVPQQAWIQNATLKDNILF : 723
Ame_1 : ELDENSVDRRAI--TGSADSIRIGDGAFSWSKEDRPALKRINVRIPEGALVAVVGHVGSGKSSLLSALLGEMHKQEGDVSIKGSVAYVPQQAWIQNATLRENIMF : 723
Loc_1 : ELDEDGVNRKIT--AGSSDSISIVDGVFSWSKKDTPTLKRISVRIPEGALVAVVGHVGSGKSSLLSALLGEMEKQEGQVSVKGSVAYVPQQAWIQNATLRENVIF : 726
Cmi_1 : ELDPGNVNRNDL--KGSAYSISMKNATLSWSKDDSPCLKDINLAILEGSLVAVVGHVGCGKSSLISALLGEMEKQEGYVAVKGTVAYVSQQAWIQNTTLKDNIIF : 707
Ler_1 : QLDLDAVDRQFN--PGSLNSIIVNNGTFSWSREDPPCLNNISVEIPDGTLIAVVGHVGCGKSSLLSALLGEMEKMEGYVAVKESVAYVSQQAWIQNASLKRNILF : 242
Rty_1 : ELDLDTVDKHSV--HTSWNSIIVNDGTFRWSNEDPPCLNNIDVEIPEGSLVAIVGHVGCGKSSLLSALLGEMQK-QGYVAVKGSVAYVPQQAWIQNASLRANIIF : 122
Sca_1 : ELDLDTVDRHLV--HSSLNSIIVKNGTFSWSKDDAPCLNKIDVEIPEGSLVAVVGHVGCGKSSLLSALLGEMQKIEGYIAVKGSVAYVPQQSWIQNASLRANIIF : 608


 740 * 760 * 780 * 800 * 820 * 840
Hsa_6 : GQELDPPWLERVLEACALQPDVDSFPEGIHTSIGEQGMNLSGGQKQRLSLARAVYRKAAVYLLDDPLAALDAHVGQHVFNQVIGPGGLLQGTTRILVTHALHILP : 818
Ptr_6 : GQELDPPWLERVLEACALQPDVDSFPEGVHTSIGEQGMNLSGGQKQRLSLARAVYRKAAVYLLDDPLAALDAHVGQHVFNQVIGPGGLLQGTTRILVTHALHILP : 660
Ggo_6 : GQELDPPWLERVLEACALQPDVDSFPEGVHTSIGEQGMNLSGGQKQRLSLARAVYRKAAVYLLDDPLAALDAHVGQHVFNQVIGPGGLLRGTTRILVTHALHILP : 813
Mmu_6 : RQELDLPWLQKVLDACALGSDVASFPAGVHTPIGEQGMNLSGGQKQRLSLARAVYKKAAIYLLDDPLAALDAHVSQQVFKQVIGPSGLLQGTTRILVTHTLHVLP : 810
Cluf_6 : RQKLDPLWLETVLEACALWPDVSGFPAGVHTKIGEQGMNLSGGQKQRLSLARAVYSKAAVYLLDDPLVALDAHVGQSVFNQVIGPGGLLHGTTRILVTHALHVLP : 819
Bta_6 : GQELDAPWLETVLEACALWPDVDGFPAGVHTRTGEQGMNLSGGQKQRLSLARAVYRKAAVYLLDDPLAALDAQVGQHVFNRVIGPDGLLQGTTRILVTHALHILP : 819
Dno_6 : GKELDWPWLQRVLQACALWPDVGGLPAGVHTQIGEQGMNLSGGQKQRLSLARAVYRKAAVYLLDDPLAALDAHVGQQVFSRVIGPDGLLQGTTRILVTHARHVLP : 747
Mdo_6 : GQELNMPWLDRVLEACALPPDLASFPAGIHTEIGEQGINLSGGQKQRLSLARAVYKKAAIYLLDDPLAALDAHVGQHIFDRVIGPGGLLHGTTRILVTHAVHILP : 819
Oan_6 : GQELEEPWFSRVLEACALQPDLASLPAGVHTEIGEQGINISGGQKQRVSLARAVYRRASVYLLDDPLSAVDAHVGQHIFDHIIGPDGLLKDTTRILVTHAVSVLP : 759
Gga_6 : GKEMDETWFNRVVDACALQPDLESFPAGQKSEIGEKGINISGGQKQRVSLARAVYQRSSIYLLDDPLSAVDAHVGQHIFEHVLGPNGLLKDKTRVLVTHMISVLH : 822
Aca_6 : GQKLDRNWFDRVVDACALQPDLDSFPHGSQAEIGEKGVNLSGGQKQRVSLARAVYTKAEVYLLDDPLSAVDAQVGQHIFKHVLGPTGLLKNKTRLLVTNAVHLLP : 756
Xtr_6 : GRKMEKCWYDQVVQACALLPDLKILSGGENTEIGEKGVNLSGGQKQRISIARAVYRNCDVYLLDDPLSAVDAHVGQHLFEQVIGPSGLLKDKTRVLVTHGVSFLP : 796
Lch_6 : GQEMNESWYHRVIEACALLPDLEILPAGDGTEIGEKGVNLSGGQKQRVSLARAVCRRSAVYLLDDPLSAVDARVGQSIFEKVIGPNGLLKHKTRVLVTHAVSVLP : 826
Tn1_6 : GREKSKAWYQRVLEACALLPDLDILPAGDATEIGEKGLNLSGGQKQRVSLARAVYRKADVYLLDDPLSAVDAHVGQHIFDKVIGPKGVLRDKTRILVTHGMSFLP : 828
Gac_6a : GREKKESWYLRVLEACALLPDLEMLPAGDGTEIGEKGLNLSGGQRHRVSLARSVYRRSDVYLLDDPLSAVDAHVGQHIFDRVIGPRGLLKEKTRVLVTHGLSFLS : 830
Gac_6b : GREKLKTWYHRVLEGCALLPDLEILPAGDATEIGEKGLNLSGGQKQRVSLARAVYRKADVYLMDDPLSAVDAHVGQHIFDKVIGPKGVLRDKTRILVTHGMSFLP : 828
Dla_6 : GREKMKTWYHRVLEACALLPDLDILPAGDATEIGEKGLNLSGGQKQRVSLARAVYRKADVYLLDDPLSAVDAHVGQHIFDKVIGPKGVLRDKTRILVTHGMSFLP : 820
Oni_6 : GREKLKTWYHRVLEACALLPDLDILPAGDATEIGEKGLNLSGGQKQRVSLARAVYRKADVYLLDDPLSAVDAHVGQHIFDKVIGPKGVLRDKTRILVTHGMSFLP : 828
Ola_6 : GREKLKTWYQRVLEACALLPDLDILPAGDATEIGEKGLNLSGGQKQRVSLARAVYRKADLYLLDDPLSAVDAHVGQHIFDKVIGPKGVLKDRTRILVTHGMSFLP : 820
Xma_6 : GRDKLKTWYQRVLEACALLPDLDILPAGDATEIGEKGLNLSGGQKQRVSLARAVYRKADVYLLDDPLSAVDAHVGQHIFDRVIGPKGVLRDKTRILVTHGMSFLP : 820
Gmo_6 : GREKLKTWYQCVLEACALLPDLDILPAGDATEIGEKGLNLSGGQKQRVSLARAVYRRADVYLLDDPLSAVDAHVGQHIFDKVIGPKGVLRDKTRVLVTHGMSFLP : 755
Dre_6a : GREKKESWYQRVLEACALLPDLDNLPAGDATEIGEKGLNLSGGQKQRVSLARAVYRKGDVYLLDDPLSAVDAHVGQHIFNKVIGPKGILRDKTRVLVTHGMSFLP : 819
Dre_6b1 : GCEKKDSLYQKVLEACALLPDLEILPARDATEIGEKGLNLSGGQKQRVSLARAVYRNSDIYLLDDPLSAVDAHVGQHIFEKVIGPNGSLKNKTRVLVTHGLSFLP : 780
Dre_6b2 : GCEKKDSLYQKVLEACALLPDLEILPARDATEIGEKGLNLSGGQKQRVSLARAVYRKADIYLLDDPLSAVDAHVGQHIFEKVIGPNGILKNKTRVLVTHGLSFLP : 192
Ame_6a : GREKKKTWYQRVLEACALLPDLEILPAGDATEIGEKGLNLSGGQKQRVSLARAVYRKADIYLLDDPLSAVDAHVGQHIFDKVIGPKGVLRDKTRVLVTHGTSFLP : 821
Ame_6b : GQEKKESWYQTVLEACALVRDLNILPARDATEIGEKGLNLSGGQKQRVSLARAVYRNADIYLLDDPLSAVDAQVGQHIFDRVIGPKGILKNKTRVLVTHGLNFLP : 832
Loc_6 : GREKKEAWYQRVVEACALLPDLEILPAGDATEIGEKGLNLSGGQKQRVSLARAVYRKADVYLLDDPLSAVDAHVGQHIFDKVIGPKGLLRDKTRVLVTHGLSFLP : 819
Hsa_1 : GCQLEEPYYRSVIQACALLPDLEILPSGDRTEIGEKGVNLSGGQKQRVSLARAVYSNADIYLFDDPLSAVDAHVGKHIFENVIGPKGMLKNKTRILVTHSMSYLP : 831
Ptr_1 : GCQLEEPYYRSVIQACALLPDLEILPSGDRTEIGEKGVNLSGGQKQRVSLARAVYSNADIYLFDDPLSAVDAHVGKHIFENVIGPKGMLKNKTRILVTHGMSYLP : 731
Ggo_1 : GCQLEEPYYRSVIQACALLPDLEILPSGDRTEIGEKGVNLSGGQKQRVSLARAVYSNADIYLFDDPLSAVDAHVGKHIFENVIGPKGMLKNKTRILVTQSMSYLP : 762
Mmu_1 : GHPLQENYYKAVMEACALLPDLEILPSGDRTEIGEKGVNLSGGQKQRVSLARAVYSNSDIYLFDDPLSAVDAHVGKHIFEKVVGPMGLLKNKTRILVTHGISYLP : 831
Cluf_1 : GRQLQERYYKAVIEACALLPDLEILPSGDRTEIGEKGVNLSGGQKQRVSLARAVYCDSDIYLFDDPLSAVDAHVGKHIFENVIGPKGMLKNKTRLLVTHSISYLP : 831
Bta_1 : GRQLQERYYKAVVEACALLPDLEILPSGDRTEIGEKGVNLSGGQKQRVSLARAVYCDSDVYLLDDPLSAVDAHVGKHIFENVIGPKGLLKNKTRLLVTHAISYLP : 817
Dno_1 : GRQLQERCYKAVIKACALLPDLEILPTGDRTEIGEKGVNLSGGQKQRVSLARAVYCNADIYLLDDPLSAVDAHVGKHIFENVVGPKGLLKNKTRILVTHSVNYLP : 831
Mdo_1 : GRQPQERFYKTVIESCALLPDLEILPSGDRTEIGEKGVNLSGGQKQRVSLARAVYCDSDVYLFDDPLSAVDAHVGKHIFEKVIGPQGILKNKTRILVTHSISYLS : 830
Oan_1 : GRQPEERHYKQVIEACALLPDLEILPSGDWTEIGEKGVNLSGGQKQRVSLARSVYCDADVYLFDDPLSAVDAHVGKHIFEKVIGPKGLLRNKTRILVTHGISYLP : 605
Gga_1 : GREMNESRYKRVIEACALLPDLEILPMGDRTEIGEKGVNLSGGQKQRVSLARAVYCNADTYLFDDPLSAVDAHVGKHIFEKVIGPKGILKNKTRVLVTHAVNYLP : 828
Aca_1 : GREARERQYNCVVEACALLPDLEVLPSGDQTEIGEKGVNLSGGQKQRVSLARAVYSDADIYLMDDPLSAVDAHVGRHIFEKVIGPKGILKKKTRILVTHGVSYLP : 810
Xtr_1 : GRESNESMYKKVIEACALLPDLEILPTGDRTEIGEKGVNLSGGQKQRVSLARAVYCNTDIYLLDDPLSAVDAHVGKHIFDKVIGPKGMLKNKTRILVTHGVSYLP : 826
Lch_1 : GQEMNENWYRCVVEACALLPDLEILPAGDSTEIGEKGVNLSGGQKQRVSLARAVYCNRAVYLMDDPLSAVDAHVGKHIFEKVIGPKGLLKNKTRVLVTHGVSYLP : 828
Tni_1 : GQEFRESWYHSVIKVCNLI--LKVIFDKFNMQCHSQGVNLSGGQKQRVSLARAVYCDRAVYLLDDPLSAVDAHVGRHIFDQVVGPQGLLKAKTRLLVTHGLSFLS : 761
Gac_1 : GQESREAWYQRVVEACALQPDLEILPAGDETEIGEKGVNLSGGQKQRVSLARAVYCERDVYLLDDPLSAVDAHVGKHIFEQVVGPQGLLKDKTRVLVTHGLSYLP : 826
Dla_1 : GQERRDSWYQCVVDACALRPDLEILPAGDETEIGEKGVNLSGGQKQRVSLARAVYCDRAVYLLDDPLSAVDAHVGKHIFDQVIGPQGLLKDKTRVLVTHGLSYLP : 771
Oni_1 : GHERRQSWYQHVVEACALQPDLEILPAGDDTEIGEKGVNLSGGQKQRVSLARAVYCDRAVYLLDDPLSAVDAHVGKHIFDQVIGPQGLLKDKTRVLVTHGLSYLP : 828
Ola_1 : GQKRKEAWYHRVVEACALHQDLEILPAGDETEIGEKGVNLSGGQKQRVSLARAVYCDRSVYLLDDPLSAVDAHVGKHIFDHVIGPQGLLKDKTRILVTHGLSYLP : 828
Xma_1 : GQKRREDWYNHVVEVCALQPDLEILPAGDETEIGEKGVNLSGGQKQRVSLARAVYCDRAVYLLDDPLSAVDAHVGKHIFDYVIGPQGILREKTRVLVTHGLSFLP : 717
Dre_1 : GRETKDSWYQKVVEACALLPDLEILPGGDTTEIGEKGVNLSGGQKQRVSVARAVYCNCSVYLLDDPLSAVDAHVGKHIFEKVIGPQGLLQGRTRVLVTHGLSFLP : 828
Ame_1 : GQEKKESWYQKVLEACALLPDLEILPGGDTTEIGEKGVNLSGGQKQRVSLARAVYCDCSVYLLDDPLSAVDAHVGKHIFEKVIGPQGVLQGRTRVLVTHGLSFLP : 828
Loc_1 : GREKKEAWYQRVVEACALLPDLEILPAGDATEIGEKGVNLSGGQKLRVSLARAVYCDCAVYLLDDPLSAVDAHVGKHIFEKVIGPRGVLRGRTRVLVTHGLSFLP : 831
Cmi_1 : GQDWHKGWYNRVIRSCALLPDLEMLPAGDESEIGEKGVNLSGGQKQRVNLARAVYNDCSVYFLDDPLSAVDAHVGRHIFEKVIGPKGLLKNTTRILVTHGINFLP : 812
Ler_1 : GQELVEEWYKSVIESCALLPDLEVLPAGDETEIGEKGVNLSGGQKQRVSLARAVYSNNAVYLMDDPLSAVDAHVGRHIFDKVIGPKGLLNGKTRVLVTHGISFLP : 347
Rty_1 : GQELMEEWYRKVVESCALLPDLENLPAGDETEIGEKGVNLSGGQKQRVSLARAVYSRNSVYFLDDPLSAVDAHVGRHIFDKVIGPNGLLSGKTRVLVTHGVNFLP : 227
Sca_1 : GQELMEDWYMKVVESCALQPDLESLPAGDETEIGEKGVNLSGGQKQRVSLARAVYSDNSVYFLDDPLSAVDAHVGRHIFDKVIGPKGLLKNKTRVLVTHGVSFLA : 713


 * 860 * 880 * 900 * 920 * 940
Hsa_6 : QADWIIVLANGAIAEMGSYQELLQRKGALMCLLDQARQPGDRGEG---------ET-------------EPGTSTKDPRGTSAG--------RRPELRRE----- : 888
Ptr_6 : QADWIIVLANGAIAEMGSYQELLQRKGALMCLLDQARQPGDTGEG---------ET-------------EPGTSTKDPRGTSAG--------RRPELRRE----- : 730
Ggo_6 : QADWIIVLANGAIAEMGSYQELLQRKGALMCLLDQARQPGDIGEG---------ET-------------EPGTSTKDPRGTSAG--------RRPELRRE----- : 883
Mmu_6 : QADRILVLANGTIAEMGSYQDLLQRNGALVGLLDGARQPAGTHDA---------------------------ATSDDLGGFPGG--------GRPTCRPDRP--- : 877
Cluf_6 : QADWIVVLEDGAIAEMGRYQELLHRKGALVGLLDAARQPGDRGDG---------ET-------------ELMTNAEDPRGPAGS--------EQPVGGPE----- : 889
Bta_6 : QADWIVVLEDGAIAEMGSFQELLHRKGALVGLLDGASQPGDGGEG---------DT-------------EPPAGAKDPRGSAAG--------GRPEGRSE----- : 889
Dno_6 : QADRIAVLADGAIAEMGSYQELLHRKGALADLLGRARQPGERGEG---------EA-------------EPAAGAEDPRGSAGG--------GRPEREPE----- : 817
Mdo_6 : QADYIIMMADGAVVESGSYQELLQRNGPFTDFLGQSKQEEANASQ--------------------------------EVKSSRN--------------------- : 871
Oan_6 : RVDSIVMLVDGAIAEIGSYRELVRRKGAFVDFLCQSGQTEDAAG--------------------------GETALSAAAGTSRI--------SLASKN------- : 823
Gga_6 : QVDTIVVLVDGTIAEIGSYQELSQRSGAFAEFLQSHNTAEEKAC-------------------------SGFPATGDIRDTITS--------------------- : 881
Aca_6 : RMDRIIVVMNGEISETGSWQELVARNGAFADFLRSHGTEGGKDQ-------------------------DLQDNNIHNTAQNQK--------SMQKGMTLSDFIP : 828
Xtr_6 : QMDMIIVMSDGRVSEVGTYNELLQKNGAFSEFLNTYARKSVVFEE----------------------SYEEQISAETPNSIQGA--------------------- : 858
Lch_6 : QADSIIVMSNGGISEMGSYKELLERGGAFAELLRTYTNAEQSES------------------------------TGLREKSCSV--------TIETLGSSSMTED : 893
Tn1_6 : QADQILVLVDGEITESGSYQELLSRHG-FADFIHTFARTERKES-------------------------AIQRGAAHWVGQPGR-----------DFKAV----- : 891
Gac_6a : KTDLILVMLEGHISEMGSYKDLMDRKGNFAKFIHAFNGKRRRGSSAS----------------------RGSSASRDKSKTCVG--------SRKSASRLSELSI : 905
Gac_6b : QADFILVLGDGEITESGSYQELLSRHGAFADFIHTFANAERKESAI-----------------------QRGEKHSVKRRPCKS--------NMVDFMPSS---- : 898
Dla_6 : QADHILVLVDGEITESGSYQELLSRHGAFADFIHTFASTERKESA--------------------------IQRAGSRRSNARL--------SMVDFMPFS---- : 887
Oni_6 : QADLILVLVDGEITESGSYQELLSRHGAFADFIHTFASTERKESA---------IQRGKE---------TFQTLAGSRRSNARL--------SMVDFMPFS---- : 903
Ola_6 : QADLILVLIDGEITESGSYQELLSHHGAFADFIHTFASTEKKESA---------IQ-------------RVFCVAGSRRSNARL--------SMVDFMPFSRDLS : 895
Xma_6 : QADLILVLADGEITESGSYQELLSRHGAFADFIHTFASTERKES--------------------------VIQRAGSRRSNARL--------SMVDFMPFS---- : 887
Gmo_6 : QADLILVMVDGEITESGSYQELLSRQGAFAEFIHTFANTERKES-----------------------------------AIQRG--------EMVDFMPCS---- : 813
Dre_6a : QADLILVLVDGEISERGSYQELLNRNGAFADFIHTFANSERKEC-------------------------FSEALQRGSRKSVRL--------SVTDYMPFS---- : 887
Dre_6b1 : QADLILVMADGEIKEMGSYAELLSRKNAFAE-LKAFSVSERKMH--------------------------LVLGTRKSVSFLSI--------------------- : 837
Dre_6b2 : KADLILVIVDGEITEMGSYVELLSRKNAFAEFVKAFSVSERKESA---------------------------THKGTRKSVSRL--------SMTDFS------- : 255
Ame_6a : QADLILVLVDGEITESGSYQELLNRNGAFADFIRTFASSERKESS---------------------------VQRGSRRSCARL--------SVTDYMLFS---- : 887
Ame_6b : QADLILVMGDGEITETGSYIELLNRRNAFADFVQTFAGNERKEIS------------------------TNKGKQGFPLTENKD--------SLGNLHST----- : 900
Loc_6 : QADLILVLSDGEITEMGSYLELLGRNGAFADFIRVFASSDRKESA----------------------------VHRGPRKSSSR-------LSVTDYMPVS---- : 885
Hsa_1 : QVDVIIVMSGGKISEMGSYQELLARDGAFAEFLRTYASTEQEQDA---------EE-------------NGVTGVSGPGKEAKQ---------MENGMLVTDSAG : 905
Ptr_1 : QVDVIIVMSGGKISEMGSYQELLARDGAFAEFLRTYASTEQEQDAENGSTVMDEEE-------------AGVTGVSGPGKEAKQ---------MENGMLVTDSAG : 814
Ggo_1 : QVDVIIVMSGGKISEMGSYQELLARDGAFAELLRTYASTEQEQDA---------EE-------------NGITGVSGPGKEAKQ---------MENGMLVTDSAG : 836
Mmu_1 : QVDVIIVMSGGKISEMGSYQELLDRDGAFAEFLRTYANAEQDLA-------------------------SEDDSVSGSGKESKP---------VENGMLVTDTVG : 902
Cluf_1 : QVDVIIVMTGGKISEMGSYQELLARDGAFAEFLRTYASGDQEQAE---------QD-------------DGLTGVSSPGKEVKQ---------MENGMLVTDVAG : 905
Bta_1 : QMDVIIVMSGGKISEMGSYQELLARDGAFAEFLRTYASAEQEQGQ---------PE-------------DGLAGVGGPGKEVKQ---------MENGMLVTDTAG : 891
Dno_1 : QVDVIVVMSGGKISEMGSYQELLARDGAFAEFLRTYAGAEQEQAA---------EG-------------DGPTGVSGPAKEAKQ---------MENGMLVMDAAG : 905
Mdo_1 : QVDVIIVMSDGKISEMGSHQELLDRDGAFAEFLRTYANAEQNME---------------------------DEGTNGPVVKEVK--------QMENGVLISETAG : 900
Oan_1 : QVDKIIVMSEGKISEMGSHQELLERDGAFAEFLRTYANAEQSPDDGDVKKGEGNQPL------------EEEEGSNSPAVKEVK--------PMENGVLVMEGSA : 690
Gga_1 : QMDTILVMTDGEISEMGSYQELLKQDGAFAEFLRTYANAEQSMES---------------------------SDASSPSGKEGK--------PVENGVLVNDAPG : 898
Aca_1 : VVDTIIVLSEGKVSEMGSYQELLQRDGAFAEFLRTFASAEQTRE-------------------------SDDAGANSPAAKEEK--------HLENGILANDGPG : 882
Xtr_1 : QMDSIIVMVDGKITEVGSYQDLLMQDGAFAEFLRTYANAEQNKDQEVE---------------------SGTCPIEAPSPVPSE------EKRLENGILRNE--- : 901
Lch_1 : QMDLVVVMVDGQISEIGSYQELLKQEGAFAEFLHTYANKEQNAENEHK---------------------VGLCPVNSPTFKEGP--------LLENGIVPLQ--- : 901
Tni_1 : QTNLILVMSRGKLYR---WAPTTTAGGTHSQFLWNFLVHLEPLKI------------------------SNKIVALSNVKIIVK--------VWEENVRNRSTRT : 831
Gac_1 : QADLILVLVEGEVSEMGSYQHLMATEGAFAEFQRTYAAVEHADHD------------------------ENVTRVRKSLTHFTR--------GRLLGQLDN---- : 895
Dla_1 : QADLILVMVEGQITEMGSYQQLMATEGAFSEFQRTYSAVDHTDN------------------------------------------------------------- : 815
Oni_1 : QADLILVMMKGEISEVGSYQQLMATEGAFAEFLRTYAAVDKTDNSGE----------------------DVLSSSSEPVPNSSI-------QRLENGSVSTP--- : 901
Ola_1 : QAHLVLVMVDGEITEVGSYQQLKEKEGAFAEFLRMYAANEQSEETEWTGLDFSSENWLYVYHE------KSLSSCLEPVPNSPT-------KPMENGVG------ : 914
Xma_1 : QTDLILVMVDGEITEAGSYQQLMVQEGAFAEFLRTYATVDQTDDGVQHVPVASSRSCFF----------TLQNLKTAPKSGSKA---------VENGDL------ : 797
Dre_1 : QADLILVMVDGEITEMGSYTELLGRQGAFAEFLRTYTNTEQEEG-------------------------EESLGDAVPRK------------GLENGGPAALLRQ : 896
Ame_1 : QADLILVMVDGEITETGSYAELLNRQGAFADFLRTYANAEQDGE-------------------------PDGMTDGAPRK------------TLENGGPAAVL-- : 894
Loc_1 : QADLILVMVDGEITEVGSYSELMDRQGAFSEFLRTYANADQGEEEDQPEDGDENEGKD-----------KPEDRSSSPGREQKG---------LENGGPAAL--- : 913
Cmi_1 : QMDVIFVMVDGKISEFGTYQELLEQGGAFSEYLQAYAHKETSEPE---------VESESEDIVLLEVEDAEDDGPNRPRHKKRRLSTISSASEMQNQAKSRAVLR : 908
Ler_1 : QMDRILVMVDGKISESGTYKELLEQKGAFSEFLRMHAPTENDNA-------------------------PAIQKLNDTGLQASL------------YNILT---- : 411
Rty_1 : QMDRILVMVDGKISESGTYKELLKQNGAFAEFLRTYAPRENEVTE---------KSV----------EEEEETGQSLNHISPHK--------SPQNFHQSL---- : 301
Sca_1 : QMDRILVMVDGKISESGTYKELLKQNGAFAEFLRTYAPTDNEET-------------------------EQDQKHIHPRRTSRN--------------------- : 772


 * 960 * 980 * 1000 * 1020 * 1040 *
Hsa_6 : --------------RSIKSVPEKDRTT------SEAQTEVPLDDPDRAG-WPAGKDSIQYGRVKATVHLAYLRAVGTPLCLYALFLFLCQQVASFCRGYWLSLWA : 972
Ptr_6 : --------------RSIESVPEKDRTT------SEAQTEFPLDDPDRAG-WPAGKDSIQYGRVKATVHLAYLRAVGTPLCLYALFLFLCQQVASFCRGYWLSLWA : 814
Ggo_6 : --------------RSIKSVPEKDRTT------SEAQTEVPLDDPDRAG-WPAGKDSIQYGRVKATVHLAYLHAVGTPLCLYALFLFLCQQVASFCRGYWLSLWA : 967
Mmu_6 : --------------RPTEAAPVKGRSTS------EVQMEASLDDPEATG-LTAEEDSVRYGRVKTTIYLSYLRAVGTPLCTYTLFLFLCQQVASFSQGYWLSLWA : 961
Cluf_6 : --------------RSVKLVPEKDGTT------SEAQTGAPLAGPEWAG-RPAGEDGTQNGRVKATMYLSYFQAVGVPLCVYALFLFLCQQVASFCHGYWLSLWA : 973
Bta_6 : --------------RFMKLVPEKDSAA------SEAQTGLPLDDPEGPG-QPKGKDGTQYGRVKATMYLTYLRAVGTPLCLYALFLFLCQQVASFCRGYWLSLWA : 973
Dno_6 : --------------RSVQHGVERDSTT------SKTQSGATLEDPEGTA--PTGGDSMPYGRVKASMYLSYLRAVGAPLCLYALFLFLAQQVASFCRGYWLSLWA : 900
Mdo_6 : --------------ISESEASTNKTDSFIKDSPEKGRTTLQRAEGTKMG-QLTEGDRVHYGRVNATLYLAYLRAVGMPICLSVVFLFLCQQMISSSRGYWLSLWA : 961
Oan_6 : --------------RKFPGEVDRGRTAPTLMGKDATASKHQSEVPDVSG-RLTEEDRVQTGRVNLALYLTYMRAAGTPGCLITLLFFLCQQVASFSSSYWLSLWV : 913
Gga_6 : --------------RNNPPEDNLFSDNS-VKSPAMGRETIPSQDAEVTG-RLTRGENTQQGRVNAPVYAAYLRATGLPLCAYIILLFTCQQGVSFFRGYWLSVWT : 970
Aca_6 : KQQQ----------HGSPHSYSWNPEVSAEHSSRSEKRSVYASDLETA--ELAEEDKGPTGRAKTSIYLSYLRVAGSLAWAYIVLLFTCQQVASFCRGYWLSLWA : 921
Xtr_6 : --------------MKMKGKHSARCRND-----TDNETDDVNEIEADAG-KLTEADVALTGRVKLSVYLEYCKIMGKWYLLISALFFIVQQAASLSYNYWIGLWA : 943
Lch_6 : I-------------PAQKHSPRIPGEKTWRSAQQNGTPELG---AKDAG-KLMEADTAQSERVKLAVYQEYFKKLGSFLFLYVIWLHICQQAASFSASYWLSLWA : 981
Tn1_6 : --------------ESVLGIPAFRGDTT-----NSNLQNMEVPEPEDLG-KLTVVDKARTGRVRLEMYKKYFNTIGLAIIVPIIFLYAFQQGVSLAYNYWLSMWA : 976
Gac_6a : DLPQ----------KQLISTTEIQNKIN---PAHVSMAPGFWGSRRDAG-AVVESGGQRHGQVKLQMYREYFRTVGPTIIAAIVFLCAFQQAASLAYSYWLSLWA : 996
Gac_6b : --------------RDLSQEQLIGGDTT-----NTNLQNMEVSEPEDLG-KLTEADKARSGRVRLATYNKYFKTIGLAIIVPIVFLYAFQQGASLAYNYWLSVWA : 983
Dla_6 : --------------RDLSQEQLIGGDTT-----NTNLQNMEVSEPEDLG-KLTEADKARTGRVRLEMYQKYFKTIGLAIIIPIVFLYAFQQGASLAYNYWLSMWA : 972
Oni_6 : --------------RDLSQEQLIGGDTT-----NTNLQNMEVSEPEDLG-KLTEADKARTGRVRLDMYKKYFKTIGLAIIIPIVFLYAFQQGASLAYNYWLSKWA : 988
Ola_6 : QEQLIGYINSR---SELESNALVTGDTT-----NTNLQNMEVSEPEDLG-KLTEADKAHTGRVKLDMYKKYFKTIGLAIIIPIVFLYAFQQGASLAYSYWLSMWA : 991
Xma_6 : --------------RDLSQEQLIGGDTT-----NTNLQNMEVSEPEDLG-KLTEVDKARTGRVRLTLYKKYFKTIGLAIIILIVFLYAFQQGASLAYNYWLSMWA : 972
Gmo_6 : --------------RDLSQEQLIGGDST-----NTNLQNMEVPEPEDLG-RLTEVDKARTGRVRLDMYMEYFKTIGVALVVPIVLMYAFQQGASLTYNYWLSLWA : 898
Dre_6a : --------------RDLSQEQLIGGDTN-----SIAIEPLPSDEPEDLG-KLTKVDKARIGRVKLEMYIEYFRTIGLPLIISIVFLYAFQQAASLSNNYWLSLWA : 972
Dre_6b1 : --------------KDFSTDLIR-GDLG-----SASIQTMEISDRDEVG-RLTQADKAHTGRVKLEMYVEYFRTIGLAFIIPIIFLYAFQQVASLAYNYWLSLWA : 921
Dre_6b2 : --------------IDLSQEQLISGDMG-----SASIQTMEISDNEEVG-RLTQADKAHTGRVKLEMYVEYFRTISLALIIPIIFLYAFQQAASLAYNYWLSLWA : 340
Ame_6a : --------------RDLSQEQLIGGDTN-----SVAMQSLEDIEPEDLG-KLTEVDKARIGRVKLEMYIEYFRTIGLALIVPILFLYAFQQAASLAYNYWLSMWA : 972
Ame_6b : --------------CNESLTHLKDGDMG-----GANIQTMELSDQEELG-KLTEADKAHTGRVKLEMYVEYFRTIGLALIVPIVFLYAFQQAASLGYNYWLRLWA : 985
Loc_6 : --------------RDLSQEQLISGDTGSPSLQNMESRSDSDETPEDPG-KLTEADRARTGRVKLEMYMEYFKTIGLALILPIIFLYAFQQAASLTYNYWLSMWA : 975
Hsa_1 : KQLQ----------RQLSSSSSYSGDIS---RHHNSTAELQAEAKEETW-KLMEADKAQTGQVKLSVYWDYMKAIGLFISFLSIFLFMCNHVSALASNYWLSLWT : 996
Ptr_1 : KQLQ----------RQLSSSSSYSGDIS---RHHNSTAELQAEAKEETW-KLMEADKAQTGQVKLSVYWDYMKAIGLFISFLSIFLFMCNHVSALASNYWLSLWT : 905
Ggo_1 : KQLQ----------RQLSSSSSYSGDIS---RHHNSTAELQAEAKEETW-KLMEADKAQTGQVKLSVYWDYMKAIGLFISFLSIFLFMCNHVSALASNYWLSLWT : 927
Mmu_1 : KHLQ----------RHLSNSSSHSGDTS---QQHSSIAELQAGAKEETW-KLMEADKAQTGQVQLSVYWNYMKAIGLFITFLSIFLFLCNHVSALASNYWLSLWT : 993
Cluf_1 : KQLQ----------RQLSNSSSYSGDVS---LHHTSTAELQAGPNEDAW-KLVEADKAQTGQVKLSVYWDYMKAIGLFISFLSIFLFLCNHVASLVSNYWLSLWT : 996
Bta_1 : KQMQ----------RQLSSSSSYSGDVS---RHHTSTAELRPGPTEETW-KLVEADKAQTGQVKLSVYWDYMKAIGLFISFLSIFLFLCNHMASLVSNYWLSLWT : 982
Dno_1 : KQLQ----------RQLSSSSSYSGVVS--------TAELQAGAKEDTW-KLMEADKAQTGQVKLSVYWDYMKAIGLFISFLSIFLFLCNHVAALASNYWLSLWT : 991
Mdo_1 : KQLK----------RQLSNSSSYSTEPG----KHNSTADMQSEAQKDAW-KLMEADKAKTGQVKLSVYWDYMKAIGLFISFLSIFLFICNHVASLASNYWLSLWT : 990
Oan_1 : KQLH----------RQLSNSSTYSTDTG---KHQTSTGELHAGTDKNAW-KLMEADKAKTGQVKLSVYWEYMKAIGLFISFLSIFLFICNHVAALASNYWLSLWT : 781
Gga_1 : KLMH----------RQLSNSSTYSRETG-KSQHQSSTAELQPLAEKNSW-KLTEADTAKTGRVKATVYWEYMKAIGLYISLLSVFLFMCNHIASLASNYWLSLWT : 991
Aca_1 : NPLHSPRLKNQ---RQLSNSSTFSGEAG-KTLSQNSTTELQAPATEKSW-KLTEADTAKTGKVKPRVYWEYMKATGLWLALLSLLLFLCNHVASLASNYWLSLWT : 982
Xtr_1 : --------------RNLQRQLSASSETK-----SLNQNKTGLNSEKDDW-KLTEADKAKTGRVSYTFYYYY---------------------------------- : 952
Lch_1 : --------------KQLPRQSTTSPDTT-EPLLQKNAADTP---QQKSG-KLTEADKAQTGKVKFSVFWEYMKAIGLFLFFLSIFLFTCHHVASLASNYWLSLWT : 987
Tni_1 : AYLS----------LSLSLCSSAPGNLSIMAQPGQATKQPGIMAKSEAG-KLTEADKASTGRVKLSVFWAYLKAIGVLLSSISLLMFFTHHGVSLFSNYWLSLWT : 925
Gac_1 : --------------ASLSIGVCNSPEVS-RVSKPGQTGEEDVPGKQAEG-KLTEADKASTGRVKLSVFGSYLKAIGVLLSCVSLLMFLAQHLGSLSSNYWLSLWT : 984
Dla_1 : --------------------------------------------------------------------------------------------------------- : -
Oni_1 : --------------AGLSSSPGVCTASK---QSTKADEELSKPKNPEVG-KLTEADKASTGQVKLSVFWAYFKSIGVLLSCISLLLFLAHHLLSLFSNYWLSLWT : 988
Ola_1 : --------------PGFTGSSQSASNVS---KGSVSEQNVEKNSNAEAG-KTTDADKALTGRVKLSVFFSYLKAIGVLLSIISLLFFLSHNLLSLFANYWLSLWT : 1001
Xma_1 : --------------PALIGEPAVKTETQ-KPHKTDNARELNKTKNSEMG-KLTEADKASTGQVKLSVFWAYLKAIGVLLSCISLLLFLTHHLVSLFSNYWLSLWT : 886
Dre_1 : --------------SQISLNATGAGKTT-----QKTEANDDAATKKSAESRLTEADKANTGRVKLSVFWEYMKAIGLPLSIFSIFLFFCHHLSSLGSNYWLSLWT : 982
Ame_1 : --------------RQSQSSLNTPGAAKPQQKAESNDTQAK-KTKPDAA-KLTEADKANTGRVKLAVFWEYMKAIGVFLSFISILLFLAHHVSSLGSNYWLSLWT : 983
Loc_1 : --------------RQNSLTSSVGSDSAKALLKSGSAETPSPGAGKEAG-RLTEADKAKTGKVKLSVYWEYMKAIGIFLSFFSIFLFLSHHSASLASNYWLSLWT : 1003
Cmi_1 : RHHSPSVHRDPSTTRQQYLSPSYRQGSS-YRRQESSSADRRNNTEEKAGGKLTVADKAQTGRVKFSVYIKYIKAIGVCTSCLIVVAYICQHVAALFSNFWLSLWT : 1012
Ler_1 : --------------GSKSNIQSNDQEATQSGQENDPKVEDPKTEKKVAVVKLTEADKALTGRVKFRVFWTYVKAIGVCVCFWIILFYLCQHIASLSSNYWLSLWT : 502
Rty_1 : --------------IKINSISHDEEATP-SQQKNASNLQHAEPAKQAAGVKLTEADKALTGRVKFSVFWAYMKAIGICISFCIVLFYLCQHVASLSSNYWLSLWT : 391
Sca_1 : --------------SPLSSASYDEEAMP-SQQKNSNLQDASEKAKQTAGVKLTEADKALTGRVKFSVFWSYIKAIGTCISFWIVLFYLCQHIASLSSNYWLSLWT : 862


 1060 * 1080 * 1100 * 1120 * 1140 *
Hsa_6 : DDPAVGGQQTQAALRGGIFGLLGCLQAIGLFASMAAVLLGGARASRLLFQRLLWDVVRSPISFFERTPIGHLLNRFSKETDTVDVDIPDKLRSLLMYAFGLLEVS : 1077
Ptr_6 : DDPAVGGQQTQAALRGGIFGLLGCLQAIGLFASMAAVLLGGVRASRLLFQRLLWDVVRSPISFFERTPIGNLLNRFSKETDTVDVDIPDKLRSLLMYAFGLLEVS : 919
Ggo_6 : DDPAVGGQQTQAALRGGIFGLLGCLQAIGLFASMAAVLLGGVRASRLLFQRLLWDVVRSPISFFERTPIGNLLNRFSKETDTVDVDIPDKLRSLLMYAFGLLEVS : 1072
Mmu_6 : DDPVVDGRQMHAALRGWVFGLLGCLQAIGLFASMAAVFLGGARASGLLFRSLLWDVARSPIGFFERTPVGNLLNRFSKETDTVDVDIPDKLRSLLTYAFGLLEVG : 1066
Cluf_6 : DDPTVDGRQTQAALRGSIFGILGCLQAVGLFASMAMVLLGGIRASSLLFQRLLWDVMRSPIGFFERTPIGNLLNRFSKETDIVDVDIPDKLRSLLIYVFGLLEVS : 1078
Bta_6 : DDPIVDGQQTHVALRGWVFGLLGCLQAIGLFASMATVLLGGIRASSLLFRGLLWDVARSPIGFFERTPVGNLLNRFSKETDIVDVDIPDKLRSLLMYAFGLLEVG : 1078
Dno_6 : DDPVVGGRQAHAALRGWVFGLLGCLQAVGLFASMAVVLLGGARASSLLFRRLLWDVARSPVGFFEQTPVGNLLNRFSKETDVVDVDIPDKLQALLTYAFGLLEAS : 1005
Mdo_6 : DDPVVNGTQQHTGLRVGVFGLLGCLQAIGRFGSIAVVLLGGVQASQWLFQGLLREVSRSPMTFFEQTPIGNLLNRFSKETDAVDAVIPDKFKSFLGFLFGLLEVI : 1066
Oan_6 : DDPLTDGVQQHTRLRLGVFGALGFLQAIGKFGSIAAVLLGGVCASHRLFRELLRSVSRCPMGFFEKTPVGNLLNRFSKDMDAIDAEIPDKLKSFLGFMCGLLEVC : 1018
Gga_6 : EDPVQNGTQQYTELRVGVFGALGVIQAVVRFVSTAAVFLGGVLASHKLFLQLLWNVARSPTVFFEETPIGNLLNRFSKEMDAIDSIIPDKLKSLLGFLFNLLEIY : 1075
Aca_6 : NDPVVNGTQPHTELRVGVFFFLGFAQALGKFASMATVFLAGTVASHRLFRQLLWDVVRSPMGFFEQTPSGHLLNRFSKDMDAVDSIIPDKLKSLLGFFFVLLEIY : 1026
Xtr_6 : DDPPVNGTQQHTSLRLGVYSFLGVMQALSIFAASSTIIVGGVSVSRQLHSRLLYSILRCPLSFFERTPSGNLTNRFAKEMDIIDNTVPQVLMLFIIMMLTIAEIL : 1048
Lch_6 : DDPVVNGTQQHVDVRLGVFGLLGFIQGATKFGSTMAIFVGGVMASQRLHSDLLRSVLRSPMSFFEKTPSGNLLNRFSKEIDAIDTVIPNGIKSLLGFLFSLLEVY : 1086
Tn1_6 : DDPIVNGTQIDTDLKLTVFGALGFVQGVSIFGTTVAISICGIIASRHLHMDLLMNVLRSPMSFFECTPSGNLLNRFAKEIDAIDCMVPEGLKMMLSYAFKLLEVC : 1081
Gac_6a : DEPALNATRSH-QLRLGVFAALGLTQGAAMFGTTLAIALGGIVASRHLHADLLLSVLRSPVSFFEATPSGNLLNRFSKEVDAIDCMIPDGLKMMLGYLFKLLEVC : 1100
Gac_6b : DDPIVNGTQTDTDLKLIVFGALGFVQGIAIFGTTVAISVCGIIASSHLHADLLINVLRSPMSFFECTPSGNLLNRFSKEIDAIDCMVPDGLKMMLCYVFKLMEVC : 1088
Dla_6 : DDPVVNGTQIDTDLKLTVFGALGFVQGIAIFGTTVAISICGIIASRHLHMELLINVLRSPMSFFECTPSGNLLNRFAKEIDAIDCMVPDGLKMMLSYVFKLMEVC : 1077
Oni_6 : DDPVVNGTQIDTDLKLTVFGALGFVQGVAIFGTTVAISICGIIASRHLHMDLLNNVLHSPMSFFESTPSGNLLNRFAKEIDAIDCMVPEGLKMMLSYVFKLMEVC : 1093
Ola_6 : DDPVVNGTQTDRDLKLAVFGALGFVQGIAIFGTTVAISICGIIASRQLHMDLLVNVLRSPMAFFESTPSGNLLNRFVKEIDAIDCMVPEGLKMMLSYVFKLVEVC : 1096
Xma_6 : DDPVVNGTQIDTDLKLSVFGALGFVQGIAIFGTTVAISICGIIASRQLHMDLLVNVLRSPMSFFESTPSGNLLNRFAKEIDAIDCMVPEGLKMMLSYVFKLLEVC : 1077
Gmo_6 : DGPIVNGTQQGTDLKLAVFGALGLAQGIAIFGTTVAISLCGIVASRQLHRELLNNVLHSPMSFFETTPSGNLLNRFAKEIDGIDCMIPDGLRMMLGYVFKLLEVC : 1003
Dre_6a : DQPVINGTQLNTDLKLGVYGALGFAQGISIFGTTVAISLGCIIASRHLHLDLLNNVLHSPMSFFESTPSGNLLNRFAKEIDAIDNMIPDGLKMMLSYFFKLTEVC : 1077
Dre_6b1 : DDPVINGTQVNTDLKLGVYGALGFAQGIAIFGTTVAISLGGIIASRQLHLDLLNNVLHSPMSFFESTPSGNLLNRFSKEIDAIDCMIPHGLKIMLGYVFKLLEVC : 1026
Dre_6b2 : DDPVINGTQVNTDLKLGVYGALGFAQGIAIFGTTVAISLGGIIASRQLHLDLLNNVLHSPMSFFESTPSGNLLNRFSKEIDAIDCMIPDGLKMMLGYVFKLLEVC : 445
Ame_6a : DEPVINGTQLNTDMKLGVYGALGFAQGMAIFGTTVAISIGGIIASRQLHLDLLKNVLHSPMSFFESTPSGNLLNRFAKEIDAIDCMIPDGLKMMLSYFFKLMEVC : 1077
Ame_6b : DDPVINGTQADADVKLAVFGALGIAQGVAIFGTTVAISLGGIIASRHLHMDLLSNVLHSPMAFFESTPSGNLLNRFSKEVDAIDCMIPDGFKMMLGYVFKLMEVC : 1090
Loc_6 : DDPIVNGTQLDTDLKLGVYGVLGFAQGVAIFGTNVAISVGGIIASRHLHQELLHNVLRSTMSFFERTPSGNLLNRFSKEIDAIDCMIPDGLKMMLGYLFKLLEVC : 1080
Hsa_1 : DDPIVNGTQEHTKVRLSVYGALGISQGIAVFGYSMAVSIGGILASRCLHVDLLHSILRSPMSFFERTPSGNLVNRFSKELDTVDSMIPEVIKMFMGSLFNVIGAC : 1101
Ptr_1 : DDPIVNGTQEHTKVRLSVYGALGISQGIAVFGYSMAVSIGGILASRCLHVDLLHSILRSPMSFFERTPSGNLVNRFSKELDTVDSMIPEVIKMFMGSLFNVIGAC : 1010
Ggo_1 : DDPIVNGTQEHTKVRLSVYGALGISQGIAVFGYSMAVSIGGILASRCLHVDLLHSILRSPMSFFERTPSGNLVNRFSKELDTVDSMIPEVIKMFMGSLFNVIGAC : 1032
Mmu_1 : DDPVVNGTQANRNFRLSVYGALGILQGAAIFGYSMAVSIGGIFASRRLHLDLLYNVLRSPMSFFERTPSGNLVNRFSKELDTVDSMIPQVIKMFMGSLFSVIGAV : 1098
Cluf_1 : DDPIVNGTQEHTKIRLSVYGALGISQGITVFGYSMAVSIGGIFASRRLHVDLLQNVLRSPMSFFERTPSGNLVNRFSKELDTVDSMIPQVIKMFMGSLFNVIGAC : 1101
Bta_1 : DDPIVNGTQEHTQVRLSVYGALGISQGITVFGYSMAVSIGGIFASRRLHLDLLHNVLRSPISFFERTPSGNLVNRFSKELDTVDSMIPQVIKMFMGSLFNVIGAC : 1087
Dno_1 : DDRVVNGTQEHTQVRLSVYGALGISQGVAVFGYSMAVSIGGIFASRRLHLDLLHNVLRSPMSFFERTPSGNLVNRFSKELDTVDSMIPQVIKMFMGSLFNVIGAC : 1096
Mdo_1 : DDPVVNGTQQHTNVRLSVYGALGISQGPARSHILRLVSFTSSLCFPRLHLDLLHNVLRSPMSFFERTPSGNLVNRFSKEMDTVDSMIPQIIKMFMGSLFNVIGAC : 1095
Oan_1 : DDPVVNGTQQYTDVRLGVYGALGISQGIAVFGYSMAVSIGGICASRRLHLDLLHSVLRSPLSFFERTPSGNLVNRFAKELDTVDSMIPQIIKMFMSSLFNVVGAC : 886
Gga_1 : DDPVVNGTQQYTNVRLGVYGALGISQGIAVFGYSMAVSIGGIFASRHLHLDLLHNVLRSPMSFFERTPSGNLVNRFSKEIDTIDSTIPPIIKMFMGSTFNVIGAC : 1096
Aca_1 : DDPVINGTQQNTDLRLAVYGALGFSQGVAVFGYSMAVSVGGLLASRRLHLGLLHSVLRCPMGFFERTPSGNLVNRFSKEIDTIDSMIPQIIKMFMGSLFNVVGAC : 1087
Xtr_1 : -DPLLWMKTTFTENEQFLSGSYGLLQNIAVYGYSTAVSLGGILASRFLHTDLLHNVLRFPMSFFERTPSGNLVNRFSKEIDTIDNTIPQIIKMFMGSLFNVIGAC : 1056
Lch_1 : DDPIINGTQQHTKVRLGVYGTLGILQGIAVLGYSMAISIGGILASRHLHLNLLHQILRSPISFFERTPSGNLVNRFSKEIDTIDTLLPAIIKMFMGSTFNVISAC : 1092
Tni_1 : DDPVVNGTQPYRVMRLGVYGSLGLAQGITVFGYSLCIFIGGILASRHLHQSMLYDVLRSPMSFFEKTPSGNLVNRFAKEMDTIDSVIPNIVKMFTGSMFTVIGAC : 1030
Gac_1 : DDPVVNGTQPNRRMRLGVYGALGLFQGLAVFGYSLSGSIGGILASRCLHQSMLYDVLRSPMSFFERTPSGNLVNRFAKEIDTIDSLIPSIIKMFMGSMFNVVGSC : 1089
Dla_1 : --------------------------------------------------------------------------------------------------------- : -
Oni_1 : DDPVVNGTQPNRLMRLGVYGAFGLSQGVAVFGYSLSMSIGGVLASRYLHQSMLYDVLRSPMSFFERTPSGNLVNRFAKEMDTIDTLIPSIIKMFLGSMFNVLGSC : 1093
Ola_1 : DDPVVNGTQPNRLMRLGVYGALGVSQGVAVCGYSLSVSIGGILASRFLHQSMLFDVLRSPMSFFERTPSGNLVNRFAKEMDTIDSVIPMILKMFMGSLFNVVGSC : 1106
Xma_1 : DDLVVNGTQPYRLRRLAVYGSFGLTQ------------------------------------------------------------------------------- : 912
Dre_1 : DDPVVNNTQPKREMRLGVYGALGISQGIAVFCYSVSVSVGGILASRYLHQTMLYNVLRSPMSFFERTPSGNLVNRFAKETDTIDSVIPSIIKMFMGSMFNVLGSC : 1087
Ame_1 : DDPVINGTQPSREMRLGVYGALGLSQGIAVFCYSISVSIGGILASRYLHETMLYNVLRSPMSFFERTPSGNLVNRFAKETDTIDSVIPSIIKMFMGSMFNVLGSC : 1088
Loc_1 : DDHIINGTQQYTEVRLSVYGVLGLCQGIAVFGYSVSVSIGGILASRYLHQTMLHNVLRSPMSFFERTPSGNLVNRFAKETDTIDSVIPGIIKMFMGSLFNVLGAC : 1108
Cmi_1 : DDPIVNGTQPFNKLRLGVYSALGLGQGFFVLCSSVLMCAGGIIASKWLHADLLNDVLQSPMNFFERTPSGNLVNRFAKDIDTIDSMIPTVIKMFLGSLFNVLIAC : 1117
Ler_1 : DDPVINGTQQRTPLRLGVYGALGIIQGVFVLISSLMMSIGGIKASRRLHVNVLYDVLMSPLSFFEQTPSGNLVNRFAKDIETIDSVLPMVIKMFLGSLFNVLAAC : 607
Rty_1 : DDPVVNGTQQHTSLRLAVYGALGLAQGVFVLLSSLMMSVGGISASRWLHVRLLYDTLMSPMSFFERTPSGNLVNRFSKDIDTIDSMIPVVIKMFLGSLFNVLVSC : 496
Sca_1 : DDPVVNGTQQHTSLRLGVYGALGIIQGLFVLFASAVMSVGGISASRWLHDHVLFDILMSPMSFFERTPSGNLVNRFSKDIDTIDSVIPVVIRMFLGSLFHVLASC : 967


 1160 * 1180 * 1200 * 1220 * 1240 * 1260
Hsa_6 : LVVAVATPLATVAILPLFLLYAGFQSLYVVSSCQLRRLESASYSSVCSHMAETFQGSTVVRAFRTQAPFVAQNNARVDESQRISFPRLVADRWLAANVELLGNGL : 1182
Ptr_6 : LVVAVATPLAIVAILPLFLLYAGFQSLYVVSSCQLRRLESASYSSVCSHMAETFQGSTVVRAFRTQAPFVAQNNARVDESQRISFPRLVADRWLAANVELLGNGL : 1024
Ggo_6 : LVVAVATPLAIVAILPLFLLYAGFQSLYVVSSCQLRRLESASYSSVCSHMAETFQGSTVVRAFRTQAPFVAQNNARVDESQRISFPRLVADRWLAANVELLGNGL : 1177
Mmu_6 : LAVTMATPLAIVAILPLMVLYAGFQSLYVATSCQLRRLESARYSSVCSHMAETFQGSLVVRAFRAQASFTAQHDALMDENQRVSFPKLVADRWLATNLELLGNGL : 1171
Cluf_6 : LVVTVTTPLAMMAILPLLVFYAGFQSLYVASICQLRRLESARHSFVCSHVAETFQGSVVVRAFQAQCRFVAQNDTHVDESQRVNFPRLVADRWLAANLELLGNML : 1183
Bta_6 : LVVTVTTPLAVVAILPLLLLYAGFQSLYVASSCQLRRLESARYSYVCSHVAETFQGGPVVRAFRVQGPFTAQNDAHVDESQRVSFPRLVADRWLAANLELVGNGL : 1183
Dno_6 : LVVAVATPLAIAVILPLLLLYAGFQSLYVASSCQLRRLESASHSPVCSHVAETFQGSPVVRAFRAQGPFVAQSNAHVDKSQRVSFSRLVADSWVTSAVGLRGKGG : 1110
Mdo_6 : VVVVVATPLAAVMVLPLMALYVGLQSLYVASSCQLRRLESASRSPIYSHISETFQGNAVIRAFQAQDQFIAQNDSRIDEHQRASFPRLVADRWLATNMELLGNVL : 1171
Oan_6 : LVVVVATPMATLVILPLLLFYGVFQSFYVASSCQLRRLESASQSPIYSHISMTFQGSGVIRAFRAQSRFVSRSDGHVDENQRVSFPRLVADRWLATNLELLGNGV : 1123
Gga_6 : LVIVVVTPKAAMAIVPLTAFYAVFQHFYVITSCQLRRMEAASRSPIYSHISETFQGSSVIRAYKDQERFILKINCLVDENLRICFPGAVADRWLATNLEFLGNGI : 1180
Aca_6 : IVIIVATPIVVVAIVPLTVLYAVSQNFFIATSCQLKRLEAASRSPIYSNISETFEGSNSIRAYKAQQRFVLQNDFNVDENQRASYPAVVADRWLATNIEFLGNGI : 1131
Xtr_6 : LVISIATPLAAVAFIPLGLLYFFLQRFYVASSRQLKRLDAVSKSPLYTHFNESLQGVYVIRAFREQERFIQDNNMRLNMNQRFYFCSFVANRWLSVRCDFLSNFI : 1153
Lch_6 : LVVLVATPIAAVIIVPLTVMYCVIQSFYVATSCQLRRLESVSRSPIFSHVNETYQGASVIRAFGEQMRFLSQNDSKVDENQKAYYPSVVANRWLAVNLEFLANVI : 1191
Tn1_6 : IIVMMATPFAAVIILPLAFLYACVQSFYVATSCQLRRLEAVSRSPIYTHFNETVQGASVIRAFGEQPRFILQANKRVDFNQTSYFPRFVATRWLAVNLEFIGNGV : 1186
Gac_6a : VVLLLATPLTGLVLLPLACIYIFIQSFYVASSCQLRRLEAVSRSPVYSHLNETVQGAAVVRAFGEQGRFVLEADRRVDRNQEAYFPRFVATRWLAVNLEFLGNLL : 1205
Gac_6b : IIVLLAMPFAAVVILPLALLYAFVQSFYVATSCQLRRLEAVSRSPIYTHFNETVQGASVIRAFGEQSRFILQTNKRVDFNQTSYFPRFVATRWLAVNLEFVGNGV : 1193
Dla_6 : IIVLMATPFAAVIILPLAFLYAFVQSFYVATSCQLRRLEAVSRSPIYTHFNETVQGASVIRAFGEQPRFILQANQRVDFNQTSYFPRFVATRWLAVNLEFVGNGV : 1182
Oni_6 : IIVLMATPFAAVIILPLSFLYAFVQSFYVATSCQLRRLEAVSRSPIYTHFNETVQGASVIRAFGEQSRFILQANDRVDFNQTSYFPRFVATRWLAVNLEFVGNGV : 1198
Ola_6 : IIVLIATPIAAVIILPLAFLYAFVQSFYVATSCQLRRLEAVSRSPIYTHFNETVQGASVIRAFGEQSRFIMQANERVDFNQTSYFPRFVATRWLAVNLEFVGNGV : 1201
Xma_6 : IIVLMATPFAAVIILPLAFLYAFVQSFYVATSCQLRRLEAVSRSPIYTHFNETVQGASVIRAFGEQPRFILQANERVDFNQTSYFPRFVATRWLAVNLEFVGNVV : 1182
Gmo_6 : VIVMMATPFAAVFILPLAFLYAFVQSFYVATSCQLRRLEAVSRSPIYTHFNETVQGASVIRAFREERRFILQANRRVDLNQTAYFP-FVATRWLAVNLEFVGNGV : 1107
Dre_6a : IIVLMATPFAAVIILPMVFLYGFIQSFYVATSCQLRRLESVSRSPIYTHLNETVQGASVIRAFNEQSRFIMGANHKVDHNQTAYFPRFIATRWLGVNLEFLGNGI : 1182
Dre_6b1 : IIVLMATPFAGVIILPLTLLYAFIQSFYVATSCQLRRLESVSRSPIYTHFNETVQGASVIRAFGEQPRFILQANCRVDLNQTSYFPRFVASRWLAVNLEFLGNLL : 1131
Dre_6b2 : IIVLMATPFAGVIILPLALLYAFIQSFYVATSCQLRRLESVSRSPIYTHFNETVQGASVIRAFGEQPRFILQANCRVDLNQTSYFPRFVATRWLAVNLEFLGNLL : 550
Ame_6a : IIVLLATPFAAAIIFPLALLYAFVQSFYVATSCQLRRLESVSRSPIYTHFNETVQGASVIRAFSEQSRFILQANRRVDVNQTSYFPRFVATRWLAVNLEFLGNGV : 1182
Ame_6b : IIVLIATPFAGLVILPLTLFYVFIQSFYVATSCQLRRLESVSRSPIYTHFNETVQGASVIRAFGEQPRFILQANGRVDHNQTSYFPRFVATRWLAVNLEFLGNLL : 1195
Loc_6 : IIVLLAMPFAAVVILPLALFYCFIQSFYVATSCQLRRLESVSRSPIYTHFNETVQGVSVIRAFREQPRFILQANHRVDYNQTSYFPRFVATRWLAVNLEFLGNAL : 1185
Hsa_1 : IVILLATPIAAIIIPPLGLIYFFVQRFYVASSRQLKRLESVSRSPVYSHFNETLLGVSVIRAFEEQERFIHQSDLKVDENQKAYYPSIVANRWLAVRLECVGNCI : 1206
Ptr_1 : IVILLATPIAAIIIPPLGLIYFFVQRFYVASSRQLKRLESVSRSPVYSHFNETLLGVSVIRAFEEQERFIHQSDLKVDENQKAYYPSIVANRWLAVRLECVGNCI : 1115
Ggo_1 : IVILLATPIAAIIIPPLGLIYFFVQRFYVASSRQLKRLESVSRSPVYSHFSETLLGVSVIRAFEEQERFIHQSDLKVDENQKAYYPSIVANRWLAVRLECVGNCI : 1137
Mmu_1 : IIILLATPIAAVIIPPLGLVYFFVQRFYVASSRQLKRLESVSRSPVYSHFNETLLGVSVIRAFEEQERFIHQSDLKVDENQKAYYPSIVANRWLAVRLECVGNCI : 1203
Cluf_1 : IIILLATPIASIIIPPLGLIYFFVQRFYVASSRQLKRLESVSRSPVYSHFNETLLGVSVIRAFEEQERFIRQSDLKVDENQKAYYPSIVANRWLAVRLECVGNCI : 1206
Bta_1 : IIILLATPMAAVIIPPLGLIYFFVQRFYVASSRQLKRLESVSRSPVYSHFNETLLGVSVIRAFEEQERFIRQSDLKVDENQKAYYPSIVANRWLAVRLECVGNCI : 1192
Dno_1 : VIILLATPMAAVIIPPLGLIYFLVQRFYVASSRQLKRLESVSRSPVYSHFNETLLGVSVIRAFEEQERFIRQSDLKVDENQKAYYPSIVANRWLAVRLECVGNCI : 1201
Mdo_1 : IIILLATPIAAIIIPPLGLIYFFVQRFYVASSRQLKRLESVSRSPVYSHFNETLLGVSVIRAFEEQQRFIRQSDLKVDENQKAYYPSIVANRWLAVRLECVGNCI : 1200
Oan_1 : IIILLATPIAAVVIPPLGLIYFFVQRFYVTSSRQLKRLESVSRSPVYSHFNETLLGVSVIRAFEEQKRFIQQSDMKVDENQKAYYPSIVANRWLAVRLECVGNCI : 991
Gga_1 : IIILLATPIAAVVIPPLGLVYLLVQRFYVATSRQLKRLESVSRSPVYSHFNETLLGVSVIRAFEEQKRFIKQNDMKVDENQKAYYPSIVANRWLAVRLEFVGNCI : 1201
Aca_1 : VVILLATPLAAVAIPPLALVYFFVQRFYVATSRQLKRLESVSRSPVYSHFNETLLGVSVIRAFAEQQRFVRQSDLKVDQNQKAYYPSIVANRWLAVRLESVGNCI : 1192
Xtr_1 : VIILTATPIVAVIIPPLGLVYFFVQRFYVATSRQLKRLESVSRSPVYSHFNETLLGSSVIRAFGEQKRFIQISDFKVDENQRAYYPSIVSNRWLAIRLEFVGNCI : 1161
Lch_1 : IVILFATPIVAVIIPPLGLLYFFVQS------------------------------------------------------------------------------- : 1118
Tni_1 : IIILISTPLVAAIIPFLGLLYFFVQ---------------------------------------------------------------------------VGNII : 1060
Gac_1 : IIILIATPLVAIIIPFLGVLYFFVQRFYVASSRQLKRLESVSRSPIYTHFNETLLGTSVIRAFGEQERFICESDQRVDLNQKAYYPGIVANRWLAVRLEFVGNCI : 1194
Dla_1 : --------------------------------------------------------------------------------------------------------- : -
Oni_1 : VIILIATPLVSIIIPFLGLLYFFVQRFYVASSRQLKRLESVSRSPIYTHFNETLLGTSVIRAFGEQERFIHESDQRVDHNQKAYYPSIVANRWLAIRLEFVGNCI : 1198
Ola_1 : IIILVATPMVALIIPFLGVLYFFVQRFYVASSRQLKRLESVSRSPIYTHFSETLLGTSVIRAFGEQERFIHESDQRVDHNQKAYYPGIVANRWLAVRLEFVGNCI : 1211
Xma_1 : --------------------------------------------------------------------------------------------------------- : -
Dre_1 : AVILIATPLVAIIIPPLGLLYFFVQRFYVASSRQMKRLESVSRSPVYTHFNETLLGTSVIRAFGEQQRFIKESDGRVDHNQKAYFPSIVANRWLAVRLEFVGNCI : 1192
Ame_1 : AVILIATPLVAIIIPPLGLLYFFVQRFYVASSRQLKRLESVSRSPVYTHFNETLLGTSVIRAFGEQQRFIGESDRRVDHNQKAYFPSIVANRWLAVRLEFVGNCI : 1193
Loc_1 : IVILIATPMIAVIIPPLGLLYFFVQRFYVATSRQLKRLESVSRSPVYSHFNETLLGTSVIRAFQDQERFIKESDSRVDYNQKAYYPSIVANRWLAVRLEYVGNCI : 1213
Cmi_1 : IVILIATPIVAVTFLPLGFVYFFVQKFYVATSRQLKRLESVSRSPIYSHFNETLLGVSVIRAFGEQDRFLQENDLRVDENQKAYYPSIVANRWLAVRLEFVGNCI : 1222
Ler_1 : VVILIATPLTAVIIPPLGFLYIFVQRYYVATSRQLKRLESASRSPIFSNFNETLLGVSVIRAFGKEDRFIDRNDSRVDENQKAYYPSVVANRWLAVRLELVGNFI : 712
Rty_1 : TVILIATPIATVIIPPLGFLYIFIQRYYVATSRQLKRLESASRSPIFSNFSETLLGVSVIRAFGEQERFIHQNDARVDENQKAYYPSIVANRWLAVRLEFVGNCI : 601
Sca_1 : TVILVATPMATVIIPPLGFLYIFVQRYYVATSRQLKRLESASRSPIFSNFNETLLGVSTIRAFGEEDRFIYQNDSRVDENQKAYYPSIVANRWLAVRLEFVGNCI : 1072


 * 1280 * 1300 * 1320 * 1340 * 1360
Hsa_6 : VFAAATCAVLSKAHLSAGLVGFSVSAALQVTQTLQWVVRNWTDLENSIVSVERMQDYAWTPKEAPWRLPTCAAQPPWPQGGQIEFRDFGLRYRPELPLAVQGVSF : 1287
Ptr_6 : VFAAATCAVLSKAHLSAGLVGFSVSAALQVTQTLQWVVRNWTDLENSIVSVERMQDYAWTPKEASWRLPTCAAQPPWPHGGQIEFRDFGLRYRPELPLAVQGVSF : 1129
Ggo_6 : VFAAATCAVLSKAHLSAGLVGFSVSAALQVTQTLQWVVRNWTDLENSIVSVERMQDYAWTPKEAPWRLPTCAAQPPWPHGGQIEFRDFGLRYRPELPLAVQGVSF : 1282
Mmu_6 : VFVAATCAVLSKAHLSAGLVGFSVSAALQVTQTLQWVVRSWTDLENSMVAVERVQDYARIPKEAPWRLPTCAAQPLWPCGGQIEFRDFGLRHRPELPLAVQGVSL : 1276
Cluf_6 : VLAAAMCAVLSKAHLSAGLVGFSVSAALQVTQTLQWAVRSWTDLASSVVSVERMKDYVQTPKEAPWRLPACAARSPWPHGGQVEFRDFGLRHHPELPLAVRGVSF : 1288
Bta_6 : VFVAALCAVLSKAHLSPGLVGFSVSAALQVTQMLQWAVRSWTDLESSIVSVERLKDYAQTPKEAPWKPLTCAAHPPWPRRGQIEFRDLGLRYRPELPLAVRGVSF : 1288
Dno_6 : TGSQQACVLLGVLGQAAHPLWSSFPTSIEVTQTLQWAVRSWTDLESSIVAVERVQEYARTPKEAPWRLPSCAARPPWPRQGQIEFRCFGLRYRPELPLAVRGVSF : 1215
Mdo_6 : IFAAAFFAVLSKPYLRPGIVGFSVSVALQVTEILHWAVRSWTDLENNIVSVERMRDYTRTPKEAPWTLSSNGVSHTWPVMGQIEFRGYSLRYRPELALALQNLTL : 1276
Oan_6 : VLSAAIFAVMGRAHLSPGIVAFSVTTSLQVTEILHWVVRSWTSLENNIVSVERVMEYSRTPKEAPWTVASSPLPKTWPDAGRIEFRNYGLRYRAGLALALRDVTV : 1228
Gga_6 : VLFAALFATIGRTHLSPGTAGFSISYALQITGVLNWMVRSWTEIENNIVSVERVSEYSRTPKEAPWTLNDKLQGQVWLTEGRIEFRNYSLRYRPNLELALKHINL : 1285
Aca_6 : VLFAALLAVKSKPYLSPGLVGFSISYALQITGILNWMVRALAEIDNNIVSVERVRDYSGTPKEAPWTSDNKFFHENWPTEGQIAFRGYSLRYRPGLELALKNVNI : 1236
Xtr_6 : VFTVAIVGVLFRDNITPGLVGLAVVNSLRLTGVLKEAVHVATDMETNSVSVERVKEYCDAEPEAPWTSDNASDPSNWPSKGKIEFQNYGLRYRPDLDLALKNVTA : 1258
Lch_6 : VLFAAILAVNGKGRLSPGVVGLSVSHALQVTGILSWIVRSWTDIENNIVSVERVKEYSETPKEDPWILNTNFLPEPWPSEGRVEFRNYGLRYRQDLDLAVKNINV : 1296
Tn1_6 : VLAAAILSVMGRNTLSPGIVGLAVSHSLQVTAILSWIVRSWTDVENNIVSVERVNEYADTAKEASWTVEGSSLPMDWPLKGTLEFQEYGLQYRKGLELALKGITL : 1291
Gac_6a : VLAAAVLSVRGRDHLSPGIVGLAVTHSLQVTGILSWIVRSWTDVENNIVSVERVKEYDSTDKEGGWVPGGNKLPADWPATGNLQFEGYGLRYRKDLDWALNNICV : 1310
Gac_6b : VLAAAILSVMGKGTLSPGIVGLAVSHSLQVTGILSWIVRSWTDVENNIVSVERVNEYADTPKEASWSIEGSSLPPDWPQRGTIEFQDYGLQYRKGLELALKGITL : 1298
Dla_6 : VLAAAILSVMGKNTLSPGIVGLAVSHSLQVTGILSWIVRSWTDVENNIVSVERVNEYADTAKEASWSVEGSSLPLAWPQRGTLEFQDYGLQYRKGLELALKGITL : 1287
Oni_6 : VLAAAILSVMGKSTLSPGIVGLAVSHSLQVTGILSWIVRSWTDVENNIVSVERVNEYADTPKEASWSIESSSLPQAWPQNGTIEFQDYGLQYRKGLELALKGITL : 1303
Ola_6 : VLAAAVLSVIGKSTVSPGIVGLAVSHSLQVTGILSWIVRSWTDVENNIVSVERVNEYADTPKEASWNTEGSALPLAWPQSGTIEFQDYGLQYRKGLELALKGITL : 1306
Xma_6 : VLAAAILSVMGRSTLSPGIVGLAVSHSLQVTGILSWIVRSWTDVENNIVSVERVNEYADTPKEASWSTEGSSLPVAWPQSGTIELQDYGLQYRKGLELALKDITL : 1287
Gmo_6 : VLAAAILSVMGKHTLSPGIVGLAVSHSLQVTGILSWIVRSWTDVENNIVSVERVKEYADTAKEAPWTVEGSSLPPDWPQRGTIEFQDYGLQYRKGLDLALKGITL : 1212
Dre_6a : VLAASILSVMAKGTLSPGMVGLAVSHSLQVTGFLSWIVRSWTDVENNIVSVERVKEYADTPKEAAWSIEGSSLPPSWPQTGTIEFQDYGLQYRKGLELALKGISV : 1287
Dre_6b1 : VLAAAILSVMGRATLSPGTVGLAVSHSLQVTGILSWIVRSWTDVENNIVSVERVKEYAETAKEAPWTFEDSPLPSDWPRSGSIGFQAYGLQYRKGLDWALKEISL : 1236
Dre_6b2 : VLAAAILSVMGRATLSPGIVGLAVSHSLQVTGILSWIVRSWTDVENNIVSVERVKEYAETAKEAPWTIEDSPLPSDWPRCGSIGFQAYGLQYRKGLDWALKEISL : 655
Ame_6a : VLAAAILSVMGKETLSPGIVGLAVSHSLQVTAILSWIVRSWTDVENNIVSVERVKEYEETPKEAAWTSEGNSLPPTWPQVGTIEFQDYGLQYRKGLEFALKGISL : 1287
Ame_6b : VLAAAILSVTGKDTLSPGIVGLAVSHSLQVTGILSWIVRAWTDVENNIVSVERVKEYAETPKEAPWTIENRSLPSAWPQTGSIEFQQYGLQYRRGLDWALKEITL : 1300
Loc_6 : VLAAAILSVIGKGTLSPGIVGLAVSHSLQVTGILSWIVRSWTDVENNIVSVERVKEYVETAKEAAWTVESSPVPPAWPQTGTIELRGYGLQYRKGLDWALKGITV : 1290
Hsa_1 : VLFAALFAVISRHSLSAGLVGLSVSYSLQVTTYLNWLVRMSSEMETNIVAVERLKEYSETEKEAPWQIQETAPPSSWPQVGRVEFRNYCLRYREDLDFVLRHINV : 1311
Ptr_1 : VLFAALFAVISRHSLSAGLVGLSVSYSLQVTTYLNWLVRMSSEMETNIVAVERLKEYSETEKEAPWQIQETAPPSSWPQVGRVEFRNYCLRYREDLDFVLRHINV : 1220
Ggo_1 : VLFAALFAVISRHSLSAGLVGLSVSYSLQVTTYLNWLVRMSSEMETNIVAVERLKEYSETEKEAPWQIQETAPPSSWPQVGRVEFRNYCLRYREDLDFVLRHISV : 1242
Mmu_1 : VLFAALFAVISRHSLSAGLVGLSVSYSLQITAYLNWLVRMSSEMETNIVAVERLKEYSETEKEAPWQIQETAPPSTWPHSGRVEFRDYCLRYREDLDLVLKHINV : 1308
Cluf_1 : VLFAALFSVISRHSLSAGLVGLSVSYSLQVTTYLNWLVRMSSEMETNIVAVERLKEYSETEKEAPWQIQEMAPPSTWPQVGRVEFRDYGLRYRENLDLVLKHINI : 1311
Bta_1 : VLFASLFAVISRHSLSAGLVGLSVSYSLQVTTYLNWLVRMSSEMETNIVAVERLKEYSETEKEAPWQIQDMAPPKDWPQVGRVEFRDYGLRYREDLDLVLKHINV : 1297
Dno_1 : VLFAALFAVVSRHSLSAGLVGLSVSYSLQVTAYLNWLVRMSSEMETNIVAVERLKEYSETEKEVSFKVKQKKVPGPEPRTSYMGSIEYILMYKYTLCVFVEHRLY : 1306
Mdo_1 : VLFAALFSVISRHSLSPGLVGLSVSYSLQVTTYLNWLVRMSSEMETNIVAVERLKEYSETEKEAPWCIEEAAPPTNWPQQGRVEFRDFSLRYREDLDLVLKHVNV : 1305
Oan_1 : VLFAALFAVISRHSLSPGLVGLSVSYSLQVTAYLNWLVRMSSEMETNVVAVERLKEYSETEKEAPWQIEETAPAPDWPQEGKVEFRDFGLRYREDLDLVLKNINV : 1096
Gga_1 : VLFAALFAVIARNKLSPGLIGLSVSYSLQITAYLNWLVRMTSDLETNIVAVERVKEYAEMEKEAEWSIDETAPASTWPQEGKVEFRGFGLRYREDLDLVLKNINI : 1306
Aca_1 : VLFAALFAVIARHVLSPGLVGLSISYSLQITTYLNWLVRMSAEMETNIVAVERVKEYSEKEQEAEWRLPGAPIPEGWPQEGRVEFRGYSLRYRDDMDLVLRNITI : 1297
Xtr_1 : VLFASLFAVISRTTLSPGLVGLSVSYALQVTTYLNWLVRMSSELETNIVAVERVKEYADLKQEAAWTVQETAPEATWPHEGKIEFRGYGLRYREDLDLALKNINV : 1266
Lch_1 : --------------------------------------------------------------------------------------------------------- : -
Tni_1 : VSFAALCAVIARQNLSPGIMGLSISYALQLTASLTWLVRMSSDLETNIVAVEKVKEYSETQKEAEWTHKPTSLPSNWPNKGCIDIRGFSLRYRDDLDLAIRNITI : 1165
Gac_1 : VSFAALFAVVARESLSPGIMGLSISYALQLTASLTWLVRMSSEVETNIVAVERVKEYSDTEKEAEWKQEPSRLPPGWPTDGCIDIRGLGLRYRPDLDLAIRNITL : 1299
Dla_1 : --------------------------------------------------------------------------------------------------------- : -
Oni_1 : VSFAALFAVVARQSLSPGIMGLSISYALQLTTSLTWLVRMSSDVETNIVAVEKVKEYSDTEKEAAWEHEPSTLSPGWPTNGCIEMRSFGLRYRQDLDLAIRNVTI : 1303
Ola_1 : VSFAALFAVLARESLSPGIMGLSISYALQLTASLTWLVRMSSDVETNIVAVERVKEYSDTEKEAEWRHEPPTVPPDWPTEGCIRITNFGLRYRSDLDLAIRNINV : 1316
Xma_1 : --------------------------------------------------------------------------------------------------------- : -
Dre_1 : VTFAALFAVMARNNLSPGIMGLSISYALQVTASLNWLVRMSSELETNIVAVERVKEYGDTEKEAEWKLENSNLPPGWPTAGHIEIHKFGLRYREDLELAICDISV : 1297
Ame_1 : VTFAALFAVMARANLSPGIMGLSISYALQVTASLNWLVRMSSELETNIVAVERVKEYEDTEKEAEWKLEQSSVPAGWPTAGHIEVRNFGLRYREDLELAIHDITV : 1298
Loc_1 : VLFASLFAVMARDRLSPGSMGLSISYALQITASLNWLVRMSSEMETNIVAVERVKEYGDTEKEAPWQLEKSAPPRGWPTAGRIEIRDFGLRYREDLELALRDIAV : 1318
Cmi_1 : VLFAALFAVAYRLKLSAGLVGLSISYALQVTATLNWLVRMSSEVETNIVAVERVKEYSEMEKEAPWFSNNNSPTSNWIQRGTIQFIGYSARYRADLDLVLKNITL : 1327
Ler_1 : VFFAALFAVVSRSDVSPGIVGLSISYALQITATLNWLVRMTSEVETNIVAVERVQQYYDTPKEARWVIKSNRPPQTWPSEGNIEFINYGLRYRTDLDLALKDITI : 817
Rty_1 : VLFAALFAVVYRDTVSPGIVGLSVSYALQVTSTLNWMVRMTSELETNIVAVERVQEYTGTPKEAHWINRSNRPPQTWPLEGKIEFVNYGLRYREDLDLALKDITV : 706
Sca_1 : VLFAALFAVAYRDAVSPGIVGLSISYALQITSTLNWLVRMTSEVETNIVAVERVQEYSVTSKEARWVIHNNRPPKTWPSEGKVEFVNYALRYREDLDFALKDITV : 1177


 * 1380 * 1400 * 1420 * 1440 * 1460 *
Hsa_6 : KIHAGEKVGIVGRTGAGKSSLASGLLRLQEAAEGGIWIDGVPIAHVGLHTLRSRISIIPQDPILFPGSLRMNLDLLQEHSDEAIWAALETVQLKALVASLPGQLQ : 1392
Ptr_6 : KIHAGEKVGIVGRTGAGKSSLASGLLRLQEAAEGGIWIDGVPIAHVGLHTLRSRISIIPQDPILFPGSLRMNLDLLQEHSDEAIWAALETVQLKALVASLPGQLQ : 1234
Ggo_6 : KIHAGEKVGIVGRTGAGKSSLASGLLRLQEAAEGGIWIDGVPIAHVGLHTLRSRISIIPQDPILFPGSLRMNLDLLQEHSDEAIWAALETVQLKALVASLPGQLQ : 1387
Mmu_6 : KIHAGEKVGIVGRTGAGKSSLAWGLLRLQEAAEGNIWIDGVPITHVGLHTLRSRITIIPQDPVLFPGSLRMNLDLLQEHTDEGIWAALETVQLKAFVTSLPGQLQ : 1381
Cluf_6 : KIHAGEKVGIVGRTGAGKSSLAGGLLRLLEAAEGGIWIDGVPIAHVGLHTLRSRITIIPQDPTLFPGSLRMNLDMLDEHTDEAIWEALEMVQLRPLVASLPGQLQ : 1393
Bta_6 : KINAGEKVGIVGRTGAGKSSLAGGLLRLVEAAEGGIWIDGVPIAQVGLHTLRSRVTIIPQDPILFPGSLRMNLDMLQEHTDEAIWEVLETVQLRATVASLPGQLH : 1393
Dno_6 : KIQAGEKVGIVGRTGAGKSSLARGLLRLLEAAEGGIWIDGVPIAHVGLHTLRSRITIIPQDPILFPGSLRMNLDLLHEHADKAIWAALETVQLHALVTSLPGQLQ : 1320
Mdo_6 : KILPQEKVGIVGRTWAGKSSLSIGLLRLIEATEGG-------SDRWGEYQSKWQITIIPQDPILFPGSVRMNLDLLDEHSDDEIWGALEMVQLKTFILGLPGQLQ : 1374
Oan_6 : TILPQEKVGVVGRTGAGKSSLAVGLLRLFEAAEGHIRIDGVNVARIGLHHLRSKITILPQDPILFPGSLRMNLDLLHEHPDGDIWTALEMVQLKAFVADLPGHLD : 1333
Gga_6 : TINGKEKIGITGRTGAGKSTLAAGLLRLVEAAEGVILIDGQDIAQLGLHDLRMKITVIPQDPVLFSGTLRMNLDPLNQYTDADIWTALELTQLKNFVADLPEQLE : 1390
Aca_6 : QIKGKEKVGIAGRTGAGKSSLAMGLLRLVEAAEGEILIDGIDVAQIGLHDLRSKITVIPQDPVLFSGPLRMNFDPLDEHTDEDIWAALELMLLKNFVSDLPGQLA : 1341
Xtr_6 : SIQQGEKVGIVGRTGAGKSSLTLGLFRILEPATGRICIDEKDISELGLHELRSKITIIPQDPVLFSGTLRMNLDPFDNYSDNDIWVALQLAHLKVFASGLPEGLS : 1363
Lch_6 : KIDKEEKVGIVGRTGAGKSSLTMGLFRIMEASTGEVFIDGVNTATLGLHDLRSRLSIIPQDPVLFCGSLRMNLDPFDNYSDKDVWRALELAHLKNFVSSLPDRLS : 1401
Tn1_6 : NIHEREKVGIVGRTGAGKSSLALGIFRILEAAKGKIFIDGVNIADIGLHDLRSRITIIPQDPVLFSGSLRMNLDPFDTYTDEDVWRSLELAHLKTFVANLPDKLN : 1396
Gac_6a : NIQDREKVGIVGRTGAGKSSLALGIFRILEAAKGRIFIDGINIAEIGLHDLRSRITIIPQDPVLFSGSLRMNLDPFDVCSDEDLWKALELAHLSSFVSALPQKLN : 1415
Gac_6b : QVHERERVGIVGRTGAGKSSLALGIFRILEAAKGKILIDGVDIADVGLHDLRSRITIIPQDPVLFSGSLRMNLDPFDTYTDEEVWRSLELAHLQNFVSNLPDKIN : 1403
Dla_6 : NIHERERVGIVGRTGAGKSSLALGIFRILEAAKGKIFIDGVNIADIGLHDLRSRITIIPQDPVLFSGSLRMNLDPFDTYTDEEVWRSLELAHLKNFVSNLPDKLN : 1392
Oni_6 : HIHEREKVGIVGRTGAGKSSLALGIFRILEAAKGKIFIDGVDIADIGLHDLRSRITIIPQDPVLFSGSLRMNLDPFDTYTDEEVWSSLELAHLKNFVSNLPDKLN : 1408
Ola_6 : QIQKREKIGIVGRTGAGKSSLALGIFRILEAAKGRIFIDGVNIAEIGLHDLRSRITIIPQDPVLFSGSLRMNLDPFDIYTDEEIWSSLELAHLKDFVSNLPDKLN : 1411
Xma_6 : HINPKEKVGIVGRTGAGKSSLALGIFRILEAAKGKIFIDGVNIADIGLHDLRSRITIIPQDPVLFSGSLRMNLDPFDTYTDEEIWSSLELAHLKTFVSNLPDKLN : 1392
Gmo_6 : SIQEREKVGIVGRTGAGKSSLALGIFRILEAAKGKIFVDGVDLADIGLHDLRSRITIIPQDPVLFSGSLRMNLDPFDNYTDQEVWSSLELAHLKNFVSNLPDKLS : 1317
Dre_6a : HIHEREKIGIVGRTGAGKSSLALGIFRILEAAKGEIYIDGINIAEIGLHDLRSRITIIPQDPVLFSGSLRMNLDPFNAYSDEEVWNALELAHLKNFVSELPDKLN : 1392
Dre_6b1 : SVNEREKVGIVGRTGAGKSSLALGIFRILEAAKGKIFIDGINIAEIGLHELRSRITIIPQDPVLFSGSLRINLDPFDRYTDEEVWRSLELAHLKTFVSDLPDKLN : 1341
Dre_6b2 : SVNEREKVGIVGRTGAGKSSLALGIFRILEAAKGKIFIDGINIAEIGLHELRSRITIIPQDPVLFSGSLRINLDPFDRYTDEEVWRSLELAHLKTFVSDLPDKLN : 760
Ame_6a : HIQRREKIGIVGRTGAGKSSLALGIFRILEAAKGKIYIDGFDIAQLGLHDLRSRITIIPQDPVLFSGSLRMNLDPFDAYSDEEVWGALELSHLRNFVSGLPEKLN : 1392
Ame_6b : NVQEREKVGIVGRTGAGKSSLALGIFRILEAAKGEIYIDGVNIAQIGLQDLRSRITIIPQDPILFSGSLRMNLDPFDGYSDEDVWRALELAHLKNFVSGLPDKLN : 1405
Loc_6 : QIREQEKVGIVGRTGAGKSSLALGIFRILEAAKGEIYIDGVNIAEIGLHDLRSRITIIPQDPVLFSGSLRMNLDPFDSYSDEEVWNALELAHLKTFVSGLPDKLN : 1395
Hsa_1 : TINGGEKVGIVGRTGAGKSSLTLGLFRINESAEGEIIIDGINIAKIGLHDLRFKITIIPQDPVLFSGSLRMNLDPFSQYSDEEVWTSLELAHLKDFVSALPDKLD : 1416
Ptr_1 : TINGGEKVGIVGRTGAGKSSLTLGLFRINESAEGEIIIDGINIAKIGLHDLRFKITIIPQDPVLFSGSLRMNLDPFSQYSDEEVWTSLELAHLKDFVSALPDKLD : 1325
Ggo_1 : TINGGEKVGIVGRTGAGKSSLTLGLFRINESAEGEIIIDGINIAKIGLHDLRFKITIIPQDPVLFSGSLRMNLDPFSQYSDEEVWTSLELAHLKDFVSALPNKLD : 1347
Mmu_1 : TIEGGEKVGIVGRTGAGKSSLTLGLFRINESAEGEIIIDGVNIAKIGLHNLRFKITIIPQDPVLFSGSLRMNLDPFSQYSDEEVWMALELAHLKGFVSALPDKLN : 1413
Cluf_1 : TINGGEKVGIVGRTGAGKSSLTLGLFRINESAEGEIIIDDINIAKIGLHDLRVKITIIPQDPVLFSGSLRMNLDPFSQYSDEEVWTSLELAHLKDFVSGLPDKLN : 1416
Bta_1 : TIDGGEKVGIVGRTGAGKSSLTLGLFRIKESAEGEIIIDDINIAKIGLHDLRFKITIIPQDPVLFSGSLRMNLDPFSQYSDEEVWTSLELAHLKGFVSALPDKLN : 1402
Dno_1 : TLGPGDTVGIVGRTGAGKSSLTLGLFRINESAEGEIVIDGVNIAHIGLHDLRFRITIIPQDPVLFSGSLRMNLDPFSQYSDDDVWTSLELAHLKSFVSALPDKLD : 1411
Mdo_1 : TIEGGEKVGIVGRTGAGKSSLTLGLFRINESAGGEIIIDGINIAKIGLHHLRFKITIIPQDPVLFSGSLRMNLDPFDQYSDEDIWTSLELAHLKNFVSSLPDKLN : 1410
Oan_1 : TIDGGEKVGIVGRTGAGKSSLTLGLFRINESAEGEIIIDGVNIAKIGLHHLRFKITIIPQDPVLFSGSLRMNLDPFDQYSDEDIWRSLELAHLKNFVSLLPDKLN : 1201
Gga_1 : TINGGEKVGIVGRTGAGKSSLTLGLFRINEAAEGEIIIDGINIAKIGLHDLRFKITIIPQDPILFSGSLRMNLDPFDQHSDEDIWRSLELAHLKNFVSSLPDKLN : 1411
Aca_1 : TISGGEKVGIVGRTGAGKSSLTLGLFRINEAAEGQILIDGVDIASIGLHDLRFKVTIIPQDPVLFSGSLRMNLDPFEQYSDEEVWRSLELAHLKAFVSALPDKLL : 1402
Xtr_1 : TIQGGEKVGIVGRTGAGKSSLTLGLFRINEAAAGEIVIDGCNLAKIGLHDLRFRVTIIPQDPVLFSGTLRMNLDPFDKYTDDDIWTSLELAHLKRFVANLPDRLN : 1371
Lch_1 : --------------------------------------------------------------------------------------------------------- : -
Tni_1 : SINGGEKVGIVGRTGAGKSSLTLGLFRIIEAAEGHIFIDGVDIRELGLHDLRSRITIIPQDPVLFSGSLRMNLDPFDKYSDEEIWKSLEYSHLKTFVSGLPNKLN : 1270
Gac_1 : VINRGELVGIVGRTGAGKSSLTLALFRIIEASEGHIFIDGVDIALLGLHELRSRITIIPQDPVLFSGSLRMNLDPFDCYSDEEVWRALELSHLQSFVSGLPNKLS : 1404
Dla_1 : --------------------------------------------------------------------------------------------------------- : -
Oni_1 : SINGGEKVGIVGRTGAGKSSLTLGLFRIIEAAEGHIFIDGVDIAKLGLHELRSRITIIPQDPVLFSGSLRMNLDPFDSYTDEEVWRALEFSHLKTFVSSLPNKLN : 1408
Ola_1 : DISGGEKVGIVGRTGAGKSSLTLGLFRIIEPAEGNIFIDGVDIAKLGLHELRSRITIIPQDPVLFSGTLRMNLDPFDSYSDEDIWKALEFSHLKSFVSGLPDKLG : 1421
Xma_1 : --------------------------------------------------------------------------------------------------------- : -
Dre_1 : NIAGGEKVGIVGRTGAGKSSLTLGLFRIIEAAEGEIRIDGVNIADLGLHELRSRITIIPQDPVLFSGSLRMNLDPFDGYTDEEVWRSLELAHLKTFVSGLPDKLN : 1402
Ame_1 : IIEGGEKVGIVGRTGAGKSSLTLGLFRIIEAAQGEICIDGVNIANLGLHDLRSRITIIPQDPVLFSGSLRMNLDPFDGYSDEDVWRALELAHLKNFVSGLPDKLN : 1403
Loc_1 : TIEGGEKVGIVGRTGAGKSSLTLGLFRIIEPAQGQICIDGVDVSTLGLHDLRSRITIIPQDPVLFSGSLRMNLDPFDSYSDEEVWNALELAHLKTFVSGLPDKLN : 1423
Cmi_1 : TIKGGEKVGIVGRTGAGKSSIALGLFRIIEPAEGLIYLDGINISEIGLHDLRSEITIIPQEPVVFSGSLRMNLDPFEHHSDNDLWNALELAHLKTFVSDLPNTLN : 1432
Ler_1 : SINGGEKIGIVGRTGAGKSSFALGLFRIIEPAEGKILIDGIDVTKIGLHDLRFQITIIPQ--------------------------------------------- : 877
Rty_1 : SINGGEKVGIVGRTGAGKSSFALGLFRIIEAAEGQIYIDGIDIAKIGLHDLRFQITIIPQDPVLFAGSLRMNLDPFDQYPDADLWTALELAHLKTFVSGLPDTLN : 811
Sca_1 : SINGGEKIGIVGRTGAGKSSFALGLFRIIEPAEGQIYIDGIDIAKIGLHDLRFQITIIPQDPVLFSGSLRMNLDPFDQYSDAGLWKALELAHLKTFVSGLPHKLN : 1282


 1480 * 1500 * 1520 * 1540 * 1560 *
Hsa_6 : YKCADRGEDLSVGQKQLLCLARALLRKTQILILDEATAAVDPGTELQMQAMLGSWFAQCTVLLIAHRLRSVMDCARVLVMDKGQVAESGSPAQLLAQKGLFYRLA : 1497
Ptr_6 : YKCADRGEDLSVGQKQLLCLARALLRKTQILILDEATAAVDPGTELQMQAMLGSWFAQCTVLLIAHRLRSVMDCARVLVMDKGQVAESGSPAQLLAQKGLFYRLA : 1339
Ggo_6 : YKCADRGEDLSVGQKQLLCLARALLRKTQILILDEATAAVDPGTELQMQAMLGSWFAQCTVLLIAHRLRSVMDCARVLVMDKGQVAESGSPAQLLAQKGLFYRLA : 1492
Mmu_6 : YECAGQGDDLSVGQKQLLCLARALLRKTQILILDEATASVDPGTEMQMQAALERWFTQCTVLLIAHRLRSVMDCARVLVMDEGQVAESGSPAQLLAQKGLFYRLA : 1486
Cluf_6 : YECTDQGSDLSVGQKQLLCLARALLRKTQILILDEATAAVDPGTELQMQAALGSWLAQCTVLLIAHRLRSVLDCARVLVMDKGQVAESGSPAQLLAQKGLFYRLA : 1498
Bta_6 : YECTDQGDNLSVGQKQLLCLARALLRKTQILILDEATAAVDPGTERQMQAALGSWFAQCTVLLIAHRLRSVLDCARVLVMDEGQVAESGSPAQLLAQKGLFYRLA : 1498
Dno_6 : YECAEQGDDLSVGQKQLLCLARALLRKTQILILDEATAAVDPGTELQMQAALGSWLAGCTVLLIAHRLPSVMDCAR----------------------------- : 1396
Mdo_6 : YECLDQGDNL----------------------------------------------------------------------------------------------- : 1384
Oan_6 : HVCSDQGENVSVGQKQLLCLARALLRKTKILVLDEATAAVDPQTDLQIQATLRTQFANCTVLTIAHRLNTVMDCNRVLVMDDGQVVEFDSPARLLTRKGLFYRLA : 1438
Gga_6 : YKCTDQGENLSTGQKQLVCLARALLQKAKVLILDEATAAIDIETDLQIQTALRTQFKESTVLTIAHRINTIMDCDRDLVLENGQIAEFDTPKQLTAQKGLFYKLM : 1495
Aca_6 : YECSERGGNLSVGQRQLICLTRALLRRGNVVFLDEATAAVDMETDLQIQSAIRSQFRDCTVLTIAHRVSTLMDCDRIIVMESGQVSECDTPQNLIARKGMFYTMA : 1446
Xtr_6 : YICTEGGENLSVGQRQLVCLARALLRKTKILVLDEATAAVDLETDDLIQNTIRKEFEDCTIITIAHRLNTIMDYTRL---------------------------- : 1440
Lch_6 : YECSEGGENLSVGQRQLVCLARALLRKSKILVLDEATAAVDLETDDLIQSTIRTQFEDCTVLTIAHRLNTIMDCTRVMVLDRGQIVEFDAPAKLLLQKGLFYRLA : 1506
Tn1_6 : HECSEGGENLSLGQRQLVCLARALLRKTKILVLDEATAAVDLETDTLIQSTIRTQFEDCTVLTIAHRLNTIMDYTRVIVMDKGHISEMDSPGNLIAQRGQFYRMC : 1501
Gac_6a : HQCCEGGENLSLGQRQLLCLARALLRKTRILVLDEATAAVDLKTDQLIQSTIRTQFDDCTVLTIAHRLNTIMDYNRVIVMDRGYIAEIDSPSELIRLQGFFYQMC : 1520
Gac_6b : HECSEGGENLSLGQRQLVCLARALLRKTKILVLDEATAAVDLETDALIQSTIRTQFEDCTVLTVAHRLNTIMDYTRVIVMDRGHISEMDTPANLIAQRGQFYRMC : 1508
Dla_6 : HECSEGGENLSLGQRQLVCLARALLRKTKILVLDEATAAVDLETDTLIQSTIRTQFEDCTVLTIAHRLNTIMDYTRVIVMDRGHISEMDSPANLIAQRGQFYRMC : 1497
Oni_6 : HECTEGGENLSLGQRQLVCLARALLRKTKILVLDEATAAVDLETDTLIQSTIRTQFEDCTVLTIAHRLNTIMDYTRVIVMDRGHVSEMDSPANLISQRGQFYRMC : 1513
Ola_6 : HECSEGGENLSLGQRQLVCLARALLRKTKILVLDEATAAVDLETDTLIQSTIRTQFEDCTVLTIAHRLNTIMDYTRVIVMDRGYISEMDSPANLISQRGQFYRMC : 1516
Xma_6 : YECSEGGENLSLGQRQLVCLARALLRKTKILVLDEATAAVDLETDTLIQSTIRTQFEHCTVLTIAHRLNTIMDYTRVIVMDRGHISEMDSPANLISQRGQFYRMC : 1497
Gmo_6 : HECSEGGENLSLGQRQLVCLARALLRKTKILVLDEATAAVDLETDTLIQSTIRTQFEDCTVLTIAHRLNTIMDYTRVIVMDKGLISETDSPANLITQRGQFYRMC : 1422
Dre_6a : HECSEGGENLSLGQRQLVCLARALLRKTKVLVLDEATAALDLETDTLIQSTIRSQFEDCAVLTIAHRLNTIMDYTKVIVMDKGHVVEMDSPSNLIAKRGQFYYMC : 1497
Dre_6b1 : HECSEGGENLSLGQRQLICLARALLRKTKILVLDEATAAVDLKTDNLIQSTIRTQFEDCTVLTIAHRLNTIMDYTRVIVMDRGNITEIDSPSNLISQHGQFYRMC : 1446
Dre_6b2 : HECSEGGENLSLGQRQLVCLARALLRKTKILVLDEATAAVDLETDNLIQSTIRTQFEDCTVLTIAHRLNTIMDYTRVIVMDRGKITEVDSPSNLISQHGQFYRMC : 865
Ame_6a : YECSEGGENLSLGQRQLVCLARALLRKTKILVLDEATAAVDLETDTLIQSTIRSQFEDCTVLTIAHRLNTIMDYTKVIVMDKGHIAEMDTPANLIAQRGQFYRMC : 1497
Ame_6b : HECSEGGENLSLGQRQLVCLARALLRKTKILVLDEATAAVDLETDNLIQSTIRTQFEDCTVLTIAHRLNTIMDYTRVIVMDNGYITEMDSPTNLIAQRGQFYCMC : 1510
Loc_6 : HECSEGGENLSLGQRQLVCLARALLRKTKVLVLDEATAAVDLETDNLIQSTIRSQFEECTVLTIAHRLNTIMDYTRVIVMDRGQITEMDTPSNLIASRGQFYRMC : 1500
Hsa_1 : HECAEGGENLSVGQRQLVCLARALLRKTKILVLDEATAAVDLETDDLIQSTIRTQFEDCTVLTIAHRLNTIMDYTRVIVLDKGEIQEYGAPSDLLQQRGLFYSMA : 1521
Ptr_1 : HECAEGGENLSVGQRQLVCLARALLRKTKILVLDEATAAVDLETDDLIQSTIRTQFEDCTVLTIAHRLNTIMDYTRVIVLDKGEIQEYGAPSDLLQQRGLFYSMA : 1430
Ggo_1 : HECAEGGENLSVGQRQLVCLARALLRKTKILVLDEATAAVDLETDDLIQSTIRTQFEDCTVLTIAHRLNTIMDYTRVIVLDKGEIQEYGSPSDLLQQRGLFYSMA : 1452
Mmu_1 : HECAEGGENLSVGQRQLVCLARALLRKTKILVLDEATAAVDLETDNLIQSTIRTQFEDCTVLTIAHRLNTIMDYTRVIVLDKGEVRECGAPSELLQQRGIFYSMA : 1518
Cluf_1 : QECAEGGENLSVGQRQLVCLARALLRKTKILVLDEATAAVDLETDDLIQSTIRTQFDDCTVLTIAHRLNTIMDYTRVIVLDKGEIRECGQPSDLLQQRGLFYSMA : 1521
Bta_1 : HECAEGGENLSVGQRQLVCLARALLRKTKILVLDEATAAVDLETDDLIQSTIRTQFDDCTVLTIAHRLNTIMDYTRVIVLDKGEIQEWGSPSDLLQQRGLFYSMA : 1507
Dno_1 : HECAEGGENLSVGQRQLVCLARALLRKTKILVLDEATAAVDLETDDLIQSTIRTQFDDCTVLTIAHRLNTIMDYTRVIVLDKGEVREFGSPSELLQQRGLFYSMA : 1516
Mdo_1 : HECTEGGENLSVGQRQLVCLARALLRKTKILVLDEATAAVDLETDNLIQSTIRTQFDDCTVLTIAHRLNTILDYTRVIVLDKGEIVECDSPPVLLQKKGIFYSMA : 1515
Oan_1 : HECTEGGENLSVGQRQLVCLARALLRKTKILVLDEATAAVDLETDDLIQSTIRTQFDDCTVLTIAHRLNTIMDYTRILVLDKGEVVECGSPSDLLQKKGIFYSMA : 1306
Gga_1 : HECSEGGENLSVGQRQLVCLARALLRKSKILVLDEATAAVDLETDNLIQSTIKSQFEECTVLTIAHRLNTIMDYTRVLVLDRGEVVECDSPDNLLQAKGLFYSMA : 1516
Aca_1 : HECAEGGENLSVGQRQLVCLSRALLRRSKILVLDEATAAVDLETDCLIQATIRRQFEGCTVLTIAHRLNTIMDYTR----------------------------- : 1478
Xtr_1 : HECAEGGENLSIGQRQLVCLARALLRKTKILVLDEATAAVDLETDGLIQSTIRKEFQDCTVITIAHRLNTIMDYTKVIVLDKGQVVEFDSPSNLLQQQGIFFNMA : 1476
Lch_1 : --------------------------------------------------------------------------------------------------------- : -
Tni_1 : HECSEGGENLSVGQRQLLCLARALLRKSKVLVLDEATAAVDMETDHLIQATIRSQFEDCTVLTIAHRLNTIMDYSRVLVLDKGELVEFASPSNLLAEKGSFYQMA : 1375
Gac_1 : HECSEGGENLSVGQRQLLCLARALLRKTKILVLDEATAAVDMETDNLIQSTIRSQFEDCTVLTIAHRLNTVMDYTRILVLDKGEMAEFDAPHNLLAQRGAFYKMA : 1509
Dla_1 : --------------------------------------------------------------------------------------------------------- : -
Oni_1 : HDCSEGGENLSVGQRQLLCLARALLRKTRILVLDEATAAVDMETDNLIQSTIRSQFEDCTVLTIAHRLNTIMDYTRVLVLENGAMAEFDSPSNLISQRGAFYKMA : 1513
Ola_1 : HECSEGGENLSLGQRQLLCLARALLRKTKVLVLDEATAAIDMETDDLIQTTIRSQFEGCTVLTIAHRLNTIMDYTRVLVLDKGQMAEFDSPSNLIAQKGAFYRMA : 1526
Xma_1 : --------------------------------------------------------------------------------------------------------- : -
Dre_1 : HECSEGGENLSLGQRQLVCLARALLRKTKILVLDEATAAVDLETDNLIQSTIRTQFEDCTVLTIAHRLNTIMDYTRVLVLDKGQMAEFDSPSNLIAKKGIFYKMA : 1507
Ame_1 : HECSEGGENLSLGQRQLVCLARALLRKTKILVLDEATAAVDLETDNLIQSTIRTQFEDCTVLTIAHRLNTIMDYTRVLVLDKGKMAEFDSPASLIAKKGIFYKMA : 1508
Loc_1 : HECSEGGENLSLGQRQLVCLARALLRKTKVLVLDEATAAVDLETDNLIQSTIRSQFEECTVLTIAHRLNTIMDYTRVLVLDKGQIVEFDSPSNLLAKKGIFYKMA : 1528
Cmi_1 : HECSEGGENLSVGQRQLICLARALLRKSKVLVLDEATAAVDLVTDKLIQSTIKSHFDQSTVLTIAHRLHTIMDYTRVLVLDKGEIIEFDTPANLLAKKGVFYHMA : 1537
Ler_1 : --------------------------------------------------------------------------------------------------------- : -
Rty_1 : HECSEGGDNLSVGQRQLLCLARALLRKSKILVLDEATAAVDLETDELIQSTIRNQFADCTVLTIAHRLKTIMDYTRIMVLDKGQIVEFDAIPALLHRKGAFYKMA : 916
Sca_1 : HECSEGGENLSVGQRQLLCLARALLRKSKILVLDEATAAVDLETDELIQSTIRNQFAECTVLTIAHRLKTIIDYTRIMVLDRGRIVEFDTAPQLLHRKGAFHKMA : 1387


 1580
Hsa_6 : QESGLV : 1503
Ptr_6 : QESGLV : 1345
Ggo_6 : RESGLV : 1498
Mmu_6 : HESGLA : 1492
Cluf_6 : QESGLV : 1504
Bta_6 : QESGLV : 1504
Dno_6 : ------ : -
Mdo_6 : ------ : -
Oan_6 : EESGLV : 1444
Gga_6 : EESGLA : 1501
Aca_6 : KESGLA : 1452
Xtr_6 : ------ : -
Lch_6 : SDAGLT : 1512
Tn1_6 : REAGLV : 1507
Gac_6a : AEAGLV : 1526
Gac_6b : REAGLM : 1514
Dla_6 : REAGLV : 1503
Oni_6 : REAGLV : 1519
Ola_6 : REAGLV : 1522
Xma_6 : LEAGLV : 1503
Gmo_6 : REAGLV : 1428
Dre_6a : REAGLL : 1503
Dre_6b1 : REAGLV : 1452
Dre_6b2 : REAGLV : 871
Ame_6a : REAGLA : 1503
Ame_6b : REAGLA : 1516
Loc_6 : REAGLV : 1506
Hsa_1 : KDAGLV : 1527
Ptr_1 : KDAGLV : 1436
Ggo_1 : KDAGLV : 1458
Mmu_1 : KDAGLV : 1524
Cluf_1 : KDAGLV : 1527
Bta_1 : KDSGLV : 1513
Dno_1 : RDANLA : 1522
Mdo_1 : KDAGLV : 1521
Oan_1 : RDASLI : 1312
Gga_1 : KDSGLA : 1522
Aca_1 : ------ : -
Xtr_1 : KDSGLV : 1482
Lch_1 : ------ : -
Tni_1 : KDAGLV : 1381
Gac_1 : KDAGLV : 1515
Dla_1 : ------ : -
Oni_1 : KDSGLV : 1519
Ola_1 : KDSGLI : 1532
Xma_1 : ------ : -
Dre_1 : KDSGLV : 1513
Ame_1 : KDSGLV : 1514
Loc_1 : KDSGLL : 1534
Cmi_1 : ADSGLL : 1543
Ler_1 : ------ : -
Rty_1 : KDSGLL : 922
Sca_1 : VDSGI- : 1392

**Supplementary Figure 2**

**Supplementary Figure 3**

**Supplementary Figure 4**

**Supplementary Table 1**

| **Species** | **Common name** | **Abbreviation** | **ABCC6** | **ABCC1** | **ABCC3** | |
| --- | --- | --- | --- | --- | --- | --- |
| ***TETRAPODS*** |  |  |  |  |  | |
| *Homo sapiens* | Human | Hsa | ENSG00000091262 | ENSG00000103222 | ENSG00000108846 | |
| *Pan troglodytes* | Chimpanzee | Ptr | ENSPTRG00000007815 | ENSPTRG00000007812 | ENSPTRG00000009406 | |
| *Gorilla gorila* | Gorilla | Ggo | ENSGGOG00000009623 | [ENSGGOG00000004324](http://www.ensembl.org/Gorilla_gorilla/Gene/Summary?db=core;g=ENSGGOG00000004324;r=16:16408295-16606832;t=ENSGGOT00000057955) | ENSGGOP00000003354 | |
| *Mus musculus* | Mouse | Mmus | ENSMUSG00000030834 | ENSMUSG00000023088 | ENSMUSG00000020865 | |
| *Canis lupus familiaris* | Dog | Cluf | ENSCAFG00000018197 | ENSCAFG00000018208 | ENSCAFG00000017201 | |
| *Bos taurus* | Cow | Bta | ENSBTAG00000015191 | ENSBTAG00000021090 | ENSBTAG00000020070 | |
| *Dasypus novemcinctus* | Armadillo | Dno | ENSDNOG00000024923 | ENSDNOG00000014990 | ENSDNOG00000046300 | |
| *Monodelphis domestica* | Opossum | Mdo | ENSMODG00000005815 | ENSMODG00000004194 | ENSMODG00000020910 | |
| *Ornithorhynchus anatinus* | Platypus | Oan | ENSOANG00000005123 | ENSOANG00000005124 | ENSOANG00000013379 | |
| *Gallus gallus* | Chicken | Gga | ENSGALG00000038152 | ENSGALG00000006646 | ENSGALG00000007522 | |
| *Anolis carolinensis* | Anole Lizard | Aca | ENSACAG00000003478 | ENSACAG00000005349 | ENSACAG00000001396 | |
| *Xenopus tropicalis* | Xenopus | Xtr | ENSXETG00000026360 | ENSXETG00000019661 | ENSXETG00000012239 | |
| *Latimeria chalumnae* | Coelacanth | Lch | ENSLACG00000022117 | ENSLACG00000001471 | ENSLACG00000007209* | |
|  |  |  |  |  | ENSLACG00000006619* | |
| ***RAY-FINNED FISH*** |  |  |  |  |  | |
| *Gasterosteus aculeatus* | Stickleback | Gac | a ENSGACG00000019172 | ENSGACG00000000434 | ENSGACG00000005901 | |
|  |  |  | b ENSGACG00000003037 |  |  | |
| *Dicentrarchus labrax* | Sea bass | Dla | 177430 | 195360 | 00098120 | |
| *Oryzias latipes* | Medaka | Ola | ENSORLG00000013429 | ENSORLG00000017141 | ENSORLG00000020741 | |
| *Tetraodon nigroviridis* | Tetraodon | Tni | ENSTNIG00000012067 | ENSTNIG00000005013 | ENSTNIG00000004171 | |
| *Oreochromis niloticus* | Tilapia | Oni | ENSONIG00000018866 | ENSONIG00000007824 | ENSONIG00000019586 | |
| *Xiphophorus maculatus* | Platyfish | Xma | ENSXMAG00000004906 | ENSXMAG00000017319* | ENSXMAG00000012203 | |
|  |  |  |  | ENSXMAG00000007738* |  | |
| *Gadus morhua* | Cod | Gmo | ENSGMOG00000005748 | ni | ENSGMO00000010029 | |
| *Danio rerio* | Zebrafish | Dre | a ENSDARG00000016750 | ENSDARG00000104719 | ENSDARG00000096662 | |
|  |  |  | b1 ENSDARG00000105403 |  |  | |
|  |  |  | b2 ENSDARG00000094901 |  |  | |
| *Astyanax mexicanus* | Blind cave fish | Ame | ENSAMXG00000004837 | ENSAMXG00000002943 | ENSAMXG00000016253 | |
|  |  |  | ENSAMXG00000003085 |  |  | |
| *Lepisosteus oculatus* | Spotted gar | Loc | ENSLOCG00000007152 | ENSLOCG00000007196 | ENSLOCG00000010918 | |
| ***CARTILAGINOUS FISHES*** |  |  |  |  |  | |
| *Callorhinchus milii* | Elephant shark | Cmi | ni | SINCAMG00000015787 | [SINCAMG00000013336](http://imcbgenomeprojects.imcb.a-star.edu.sg/Callorhinchus_milii/Gene/Summary?db=core;g=SINCAMG00000013336;r=scaffold_242:425102-456970;t=SINCAMT00000020937) | |
| *Rhincodon typus#* | Whale shark | Rty | ni | XP_020377569.1 | XP_020375725.1 | |
| *Scyliorhinus canicula+* | Spotted catshark | Sca | ni | transcript.ctg67278 | transcript.ctg14163 |  |
| *Leucoraja erinácea+* | Little skate | Ler | ni | transcriptB2.ctg13956 | transcriptB2.ctg12190 |  |
| Squalus acanthias+ | Spiny dogfish | Sac | ni | ni | EU250283 |  |
| ***AGNATHA*** |  |  |  |  |  |  |
| *Petromyzon marinus+* | *Sea lamprey* | Pma | *ni* | AKC42143.1 | KM232930.1/ ENSPMAG00000000892  KM232931.1 |  |
| *Lethenteron camtschaticum* | *Arctic lamprey* | Lca | *ni* | KE994284.1 | KE993868.1  KE993933.1$ |  |

ni- not identified, * concatenated sequences, + transcriptome data, # predicted proteins, $ not used in the phylogenetic analysis

**Supplementary Table 2**

| **Species** | **Common name** | **Accession number** |
| --- | --- | --- |
| *Caenorhabditis elegans* | Roundworm | WBGene00003407 |
|  |  | WBGene00003408 |
|  |  | WBGene00003410 |
|  |  | WBGene00003413 |
|  |  | WBGene00003409 |
|  |  | WBGene00003414 |
| *Daphnia pulex* | Water flea | DAPPUDRAFT_347281 |
| *Anopheles gambiae* | Mosquito | AGAP009835 |
|  |  | AGAP008437 |
|  |  | AGAP027980 |
|  |  | AGAP028128 |
| *Tribolium castaneum* | Flour beetle | TC012253 |
| *Apis mellifera* | Honey bee | GB53134 |
| *Drosophila melanogaster* | Fruit fly | FBgn0032456 |
| *Helobdella robusta* | Leech | HelroG163344 |
|  |  | HelroG157076 |
| *Lottia gigantea* | Owl limpet | LotgiG107213 |
|  |  | LotgiG105097 |
|  |  | LotgiG110718 |
|  |  | LotgiG153611 |
| *Strongylocentrotus purpuratus* | Sea urchin | 26395 |
| *Ciona savigny* | Ciona | ENSCSAVG00000003792 |
|  |  | ENSCSAVG00000008135 |
| *Ciona intestinallis* | Ciona | ENSCING00000020698 |
| *Branchiostoma floridae* | Amphioxus | 118638 |
|  |  | 232174 |
|  |  | 118636 |
|  |  | 128060 |
|  |  | 90918 |
|  |  | 230771 |

**Supplementary Table 3**

|  | **MRP6** | **MRP1** |
| --- | --- | --- |
| Chimpanzee | 88/89 | 92/93 |
| Gorilla | 96/97 | 91/93 |
| Mouse | 78/86 | 87/95 |
| Dog | 80/89 | 91/96 |
| Cow | 83/90 | 90/95 |
| Armadillo | 73/81 | 86/93 |
| Opossum | 57/70 | 83/91 |
| Platypus | 59/74 | 71/78 |
| Chicken | 52/69 | 77/89 |
| Anole Lizard | 50/68 | 68/80 |
| Xenopus | 40/60 | 69/82 |
| Coelacanth | 48/67 | 49/59 |
| Stickleback | 44/64 a | 66/80 |
|  | 43/60 b |  |
| Sea bass | 42/60 | 36/44 |
| Medaka | 45/63 | 64/80 |
| Tetraodon | 45/64 | 52/66 |
| Tilapia | 45/63 | 65/81 |
| Cod | 43/62 | ni |
| Zebrafish | 45/64 a | 68/81 |
|  | 44/62 b1 |  |
|  | 44/62 b2 |  |
| Blind cavefish | 46/64 a | 68/81 |
|  | 44/62 b |  |
| Spotted gar | 47/65 | 68/81 |
| Elephant shark | ni | 61/78 |
| Whale shark | ni | 40/49 |
| Little skate | ni | 37/47 |
| Spotted catshark |  | 61/75 |
| Sea Lamprey1* | 10/15 | 15/19 |
| Sea Lamprey3a | 31/44 | 49/68 |
| Sea Lamprey3b | 40/57 | 38/52 |

** Incomplete transcript*

**Supplementary Table 4**

|  | **Human** | | | **Spotted gar** |  | **Human** | | | **Spotted gar** |
| --- | --- | --- | --- | --- | --- | --- | --- | --- | --- |
|  | ***ABCC6**** | ***ABCC6***  ***P1*** | ***ABCC6P2*** | ***abcc6**** |  | ***ABCC6**** | ***ABCC6P1*** | ***ABCC6P2*** | ***abcc6**** |
| E1 | **36**  *(+30 utr)* | **124** | **36** | **42** | E18 | **168** | -- | -- | **168** |
| *I1* | 1567 | 1565 | 1565 | 4807 | *I18* | 1171 | -- | -- | 423 |
| E2 | **183** | **184** | **183** | **177** | E19 | 175 | -- | -- | 184 |
| *I2* | 1701 | 1259 | 1259 | 354 | *I19* | 1465 | -- | -- | 268 |
| E2a | -- | ***116*** | ***116*** | **--** | E20 | 76 | -- | -- | 82 |
| I2a | -- | 325 | 325 | **--** | *I20* | 2506 | -- | -- | 108 |
| E3 | **126** | **126** | **126** | **126** | E21 | 121 | -- | -- | 118 |
| *I3* | 139 | 139 | 139 | 2569 | *I21* | 3430 | -- | -- | 237 |
| E4 | **129** | **129** | **129** | **138** | E22 | **208** | -- | -- | **208** |
| *I4* | 5104 | 5055 | -- | 3025 | *I22* | 3712 | -- | -- | 572 |
| E5 | **126** | **126** | -- | **126** | E23 | **311** | -- | -- | **311** |
| *I5* | 2077 | 2070 | -- | 1531 | *I23* | 2430 | -- | -- | 438 |
| E6 | **62** | **62** | -- | **62** | E24 | **200** | -- | -- | **200** |
| *I6* | 3325 | 3338 | -- | 802 | *I24* | 1428 | -- | -- | 223 |
| E7 | **132** | **132** | -- | **132** | E25 | **127** | -- | -- | **127** |
| *I7* | 5114 | 5166 | -- | 897 | *I25* | 1854 | -- | -- | 282 |
| E8 | **204** | **204** | -- | 201 | E26 | **102** | -- | -- | **102** |
| *I8* | 1231 | 1231 | -- | 1105 | *I26* | 1672 | -- | -- | 340 |
| E9 | **178** | **178** | -- | **178** | E27 | **147** | -- | -- | **147** |
| *I9* | 3818 | 4050 | -- | 126 | *I27* | 2631 | -- | -- | 349 |
| E10 | **162** | **1562** | -- | **162** | E28 | **159** | -- | -- | **159** |
| *I10* | 5098 | -- | -- | 335 | *I28* | 78 | -- | -- | 403 |
| E11 | **93** | -- | -- | **93** | E29 | **167** | -- | -- | **167** |
| *I11* | 2462 | -- | -- | 989 | *I29* | 3855 | -- | -- | 174 |
| E12 | **204** | -- | -- | **204** | E30 | **195** | -- | -- | **195** |
| *I12* | 1189 | -- | -- | 256 | *I30* | 336 | -- | -- | 691 |
| E13 | 114 | -- | -- | 147 | E31 | **109** | -- | -- | **109** |
| *I13* | 1619 | -- | -- | 304 |  |  |  |  |  |
| E14 | **88** | -- | -- | **88** |  |  |  |  |  |
| *I14* | 2089 | -- | -- | 865 |  |  |  |  |  |
| E15 | 127 | -- | -- | 70 |  |  |  |  |  |
| *I15* | 215 | -- | -- | 114 |  |  |  |  |  |
| E16 | 177 | -- | -- | 127 |  |  |  |  |  |
| *I16* | 3446 | -- | -- | 308 |  |  |  |  |  |
| E17 | **177** | -- | -- | **177** |  |  |  |  |  |
| *I17* | 3446 | -- | -- | 609 |  |  |  |  |  |

**Supplementary Table 5**

| **Tissue** | ***ABCC6*** | | | | ***ABCC1*** | | | | ***ABCC3*** | | | |
| --- | --- | --- | --- | --- | --- | --- | --- | --- | --- | --- | --- | --- |
|  | **Hsa** | **Gga** | **Aca** | **Dre** | **Hsa** | **Gga** | **Xtr** | **Dre** | **Hsa** | **Gga** | **Aca** | **Xtr** |
| Adenocarcinoma cell line |  |  |  |  |  |  |  |  | X |  |  |  |
| Adrenal gland |  |  |  |  | X |  |  |  | X |  |  |  |
| Aorta |  |  |  |  | X |  |  |  |  |  |  |  |
| Ascites |  |  |  |  | X |  |  |  |  |  |  |  |
| Blood |  |  |  |  |  | X |  |  |  |  |  |  |
| Brain |  |  |  |  | X | X |  |  |  |  |  |  |
| Bursa of fabricius |  |  |  |  |  | X |  |  |  |  |  |  |
| Bursal lymphocyte |  |  |  |  |  | X |  |  |  |  |  |  |
| Carcinoma, cell line |  |  |  |  |  |  |  |  | X |  |  |  |
| Cerebrum | X |  |  |  |  |  |  |  |  |  |  |  |
| Cervix, tumor tissue |  |  |  |  |  |  |  |  | X |  |  |  |
| Chondrocytes isolated from growth plate cartilage |  |  |  |  |  | X |  |  |  |  |  |  |
| Chondrosarcoma lung metastasis cell lines |  |  |  |  | X |  |  |  | X |  |  |  |
| Colon |  |  |  |  |  |  |  |  | X |  |  |  |
| Connective tissue |  |  |  |  |  | X |  |  |  |  |  |  |
| Cortex | X |  |  |  |  |  |  |  |  |  |  |  |
| Craniofacial bone elements |  |  |  | X |  |  |  |  |  |  |  |  |
| Day 1 embryo |  |  |  |  |  |  |  | X |  |  |  |  |
| Ductal carcinoma, cell line |  |  |  |  |  |  |  |  | X |  |  |  |
| Dura mater |  |  |  |  | X |  |  |  |  |  |  |  |
| EBV-transformed |  |  |  |  | X |  |  |  |  |  |  |  |
| Embryonic stem cells, cell lines |  |  |  |  | X |  |  |  |  |  |  |  |
| Embryonic tissue |  |  |  |  |  | X |  |  |  |  |  |  |
| Epididymis |  |  |  |  | X |  |  |  |  |  |  |  |
| Epiphyseal growth plate |  | X |  |  |  | X |  |  |  |  |  |  |
| Epithelioid carcinoma |  |  |  |  | X |  |  |  |  |  |  |  |
| Esophageal, tumor tissue |  |  |  |  |  |  |  |  | X |  |  |  |
| Esophagus muscle |  |  |  |  | X |  |  |  |  |  |  |  |
| Fins |  |  |  | X |  |  |  |  |  |  |  |  |
| Gallbladder | X |  |  |  |  |  |  |  |  |  |  |  |
| Gastroesophage |  |  |  |  | X |  |  |  |  |  |  |  |
| Gonad |  | X |  |  |  | X |  |  |  |  |  |  |
| Gonadal PGC |  |  |  |  | X | X |  |  |  |  |  |  |
| Head (Geisha) |  | X |  |  |  | X |  |  |  |  |  |  |
| Heart |  |  |  |  |  | X | X |  |  |  | X | X |
| Hepatocelular carcinoma, cell line | X |  |  |  |  |  |  |  |  |  |  |  |
| Human embryonic stem cells |  |  |  |  | X |  |  |  |  |  |  |  |
| Intestine |  |  |  |  |  |  | X |  |  |  |  |  |
| Invertebral disc |  |  |  | X |  |  |  |  |  |  |  |  |
| Kidney | X | X | X |  |  |  | X |  |  | X | X | X |
| Large cell carcinoma |  |  |  |  |  |  |  |  | X |  |  |  |
| Large intestine | X |  |  |  |  |  |  |  |  |  |  |  |
| Leukemia cell |  |  |  |  | X |  |  |  |  |  |  |  |
| Leukocyte |  |  |  |  |  |  |  |  | X |  |  |  |
| Liver | X | X |  |  |  | X |  |  |  |  |  |  |
| Liver and Spleen |  |  |  |  |  |  |  |  | X |  |  |  |
| Lung |  |  |  |  | X |  |  |  |  |  |  |  |
| Lymph node |  |  |  |  | X |  |  |  |  |  |  |  |
| Mitral valve |  |  |  |  | X |  |  |  |  |  |  |  |
| Neuroblastoma |  |  |  |  | X |  |  |  | X |  |  |  |
| Ovary |  | X |  |  | X | X |  |  | X |  |  |  |
| Pancreas | X |  |  |  |  |  |  |  | X |  |  |  |
| Parathyroid gland |  |  |  |  | X |  |  |  |  |  |  |  |
| Peripheral Nervous system |  |  |  |  |  |  |  |  | X |  |  |  |
| Pooled colon, kidney, stomach |  |  |  |  |  |  |  |  | X |  |  |  |
| Pterygium |  |  |  |  |  |  |  |  | X |  |  |  |
| Renal cell adenocarcinoma |  |  |  |  |  |  |  |  | X |  |  |  |
| Retinoblastoma |  |  |  |  | X |  |  |  |  |  |  |  |
| Skeletal muscle |  |  |  |  |  | X | X |  |  |  |  |  |
| Skin |  |  |  |  | X |  |  | X |  |  |  |  |
| Small intestine |  | X |  |  |  |  |  |  |  |  |  |  |
| Smooth muscle |  |  |  |  | X |  |  |  |  |  |  |  |
| Spinal cord | X |  |  |  |  |  |  |  |  |  |  |  |
| Spleen |  |  |  |  | X | X |  |  | X |  |  |  |
| Splenocytes |  |  |  |  |  | X |  |  |  |  |  |  |
| Stomach |  |  |  |  | X |  |  |  | X |  |  |  |
| Testis |  |  |  |  | X |  |  | X |  |  |  |  |
| Thalamus |  |  |  |  |  |  |  |  | X |  |  |  |
| Thymus |  |  |  |  | X |  |  |  | X |  |  |  |
| Thyroid |  |  |  |  | X |  |  |  |  |  |  |  |
| Tongue |  |  |  |  | X |  |  |  |  |  |  |  |
| Tongue, tumor tissue |  |  |  |  | X |  |  |  | X |  |  |  |
| Trunk |  | X |  |  |  |  |  |  |  |  |  |  |
| Uterus |  |  |  |  | X |  |  |  |  |  |  |  |
| Vas deferens |  |  |  |  | X |  |  |  |  |  |  |  |
| Whole embryo |  |  |  |  |  | X | X | X |  |  |  |  |
| Whole larva |  |  |  |  |  |  |  | X |  |  |  |  |
| Whole body |  |  |  |  |  |  |  | X |  |  |  |  |

Hsa- Human, Gga- chicken, Aca- Anole lizard, Dre- Zebrafish, Xtr- Xenopus.

**Supplementary Data 1**

Hsa.CFTR.ENSG00000001626 : --------------------------------------------------------------------------------------------------------------MQR------- : 3
Hsa.ABCC5.ENSG00000114770: MKDIDI--------------------------------------GKEYIIPSPGYRS--VRERTSTSGTHRDREDSKFRRTRPLQDALERAEGLSLDASMHSQLRILDEEHPKGKYHHG- : 79
Hsa.ABCC11.ENSG00000121270: -----------------MRKRTYWVPN-----------------SSGGLVNRGIDIGDDVSGLIYKTYTLQDGPWSQQERNPEAAVPPW------------------------GKYDAA- : 61
Hsa.ABCC12.ENSG00000140798: -----------------------------------------------------------MVG----------------------EGP-------------YLLDQRGRRR------- : 19
Hsa.ABCC8.ENSG000000060: NHSAAYVDQGVLNNGCFVDALNVVPHVFLLFITFPI--LFIGWGQSSKVHIHHSTWLHFPGHNLRWILTFMLLFVLVCEIAEGIVTESHLYMPAGMAFMAAVTSVVYYHNIETNFPKLLA : 118
Hsa.ABCC9.ENSG000000694: NNISSYINDGVLQNSCFVDALNLVPHVFLLFITFPI--LFIGWGQSSKVQIHHNTWLHFPGHNLRWILTFALLFVHVCEIAEGIRRESRLFMPAVMGFVATTTSIVYYHNIETNFPKLLA : 118
Hsa.ABCC10.ENSG00000124: AWPLPLEGDTTG--HCFTQLVLSALPHALLAVLSACY-LGTPRSPDYILPCSPGWRLRLAASFLLSVFPLLDLLPVALPPGAGPIGLEVCVAAVAWISH-SLALWVLAHSPHGHSRGPLL : 116
Hsa.ABCC4.ENSG000001252: ------------------------------------------------------------------------------------------------------------------------ : -
Cel.WBGene00003414: ---------------CLLRDAIAAIPLLAFWLLIPVF-VRIIRDNEPKLRAPKYFWAHLILQVLLLVDNAVFGALYFNQTDHFLLRFLQYANCCIWFSLL--------SKRLHSIHPS-F : 95
Cel.WBGene00003409: MEIDQLNSTGPPISIILP-SNYVIVPSIFFWILTPVIIHDCKTSRQTPLPWSTLMSLKWFVASLLIIDRLFVFLLAVWESLFEHVTADLFIIPFFHSFT-LLALLIATNEVRRGIHSSGP : 118
Hro.HelroG163344: NQSLTWDTDYPDLTDCFQSSVLVWLPCSFLLLVAPFYYFFYLKHVRLPMRLSKLHVAKMIFTSILIILSIISLVHNMVAYVNGWWYAVNLVSPAIQIFTLTVSMLLLMAEKEKRLVTSGV : 120
Hro.HelroG157076: ------------------------------------------------------------------------------------------------------------------------ : -
Bfl.230771: ----------PDVPPGFHDTVLVWLPCLYLWVVGPLYLRQLHGNSKVRKQTSRRHMAQTVLTALMLAVTLLELFNTIVEFASDMVAAVQIVRPLNLAVTVALAGILIQYEEERGVRSSGL : 110
Lgi.LotgiG105097: -------SSYPEFTECFQHTILVWVPCAFLWLTLPVFLQHLRRLNPQPLPLGKLSICKTVLCASLSILALID-FYPYINSQVSI-ADAFIVSTVVKFITFILAGILCQVSRRFGLPSPCL : 111
Cel.WBGene00003407: GWRN--NSNIPQVTNCGQHTDFSTIPTLFLVIFSPILFYELYKSRNSYLRSFSAISLRIIFCCLLVVDLTATVIYDFYLLFTKSYNAIHFYGDLVQYAGFCLALVLTIACRNRGIITSGV : 118
Cel.WBGene00003408: GWKN--RSSIPHVTKCGQHTDFSTIPTLFL-VVFSFIVIYQLLHRTAQLRCFSPISFRIILGCLLVVDLIATVIYDLYLYVSQSFEVVHFYGDLVQFGGICLALILTVACKNKGIITSGV : 117
Lgi.LotgiG153611: ------MFSDYGAGLCTM-----WVCLVGITVLPPL--LDLSPGTVADIQ-STLREDES-----FPVVCRLGS----WSFQVGRSFPRRFMS--------MLISLLMFLERGRGLITSGV : 89
Lgi.LotgiG110718: ------------------------------------------------------------------------------------------------------------------------ : -
Dno.ENSDNOG00000024923: -----------------------------------------------------------VLGFALIVLSTSNVSVALWKIHRGTQAPELLIYPTVWLTTMSFAMFVIHMERRKGVQASGV : 61
Mmu.ENSMUSG00000030834: NQTEQEPAAYHLLSLCFVRAASSWVPPMYLWVLGPIYLLYIHRHGRCYLRMSHLFKTKMVLGLALILLYTFNVAVPLWRIHQGVQAPELLIHPTVWLTTMSFATFLIHMERRKGVRSSGV : 120
Cluf.ENSCAFG00000018197: NWTEPEPTAAHLLNVCFLKTAGVWMPPMYLWVLGPIYLLYIHRRGKGYLRMSPLFKAKMVLGLALIILCTSSVSVALWRIQRGMQAPEILIHPTVWLTTMSFAMFLIHTERKKGVRASGV : 120
Bta.ENSBTAG00000015191: NQTEPEPAAARLLSLCFLKTAGVWVPPMYLWVLGPIHLLYIHRHDKGYIQMSRLFKAKMVLGFALIILCTSSVSVTLWRIQQGTQALEFLIHPTVWLTTMSFAVFLIHAERKKGVQASGV : 120
Ggo.ENSGGOG00000009623: NQTEPEPATTSLLSLCFLRTAGVWVPPMYLWVLGPIYLLFIHHHGRGYLRMSPLFKAKMVLGFALIVLCTSSVALTLWKMQQGTEAPEFLIHPTVWLTTMSFAVFLIHTERKKGVQSSGV : 120
Hsa.ENSG00000091262: NQTEPEPAATSLLSLCFLRTAGVWVPPMYLWVLGPIYLLFIHHHGRGYLRMSPLFKAKMVLGFALIVLCTSSVAVALWKIQQGTEAPEFLIHPTVWLTTMSFAVFLIHTERKKGVQSSGV : 120
Ptr.ENSPTRG00000007815: -----------------------------------------------------------------------------------------------------------------GFQS--- : 4
Oan.ENSOANG00000005123: --------------------------------------------------MSRLFKVKMVLSFALMSLCFFNLCFTLWKIKQGTQALELLINPAMLLITMSLTVFLIHFERLKGVQSSGV : 70
Mdo.ENSMODG00000005815: NWNETQSLGLQMSSLCLMNAAVAWLPSVYLWVISPFYFLYLRYNNKGYIRMSCLFKTKMVLGFTLVLLCFSNIIFTLWKIKKGIQAPEFLINPTVWLITMILAIFLIHLERRRGIQSSGV : 120
Gga.ENSGALG00000006698: DWNQTWYTDSPRFTWCFENTVLSWIPCAYLWICFPFYYLYLQHKNKGYIRMSHIFKIKMVLGFLLVILCFSNVFFVLWEISQGIRPPAFFISPAVLGITMILAMFLTQVERMMGIQSSGI : 120
Aca.ENSACAG00000003478: ------------------------------------------------------------LGFVFLAVYFASTCYILWEANHGIQDPGLAISCALQLATMILVLFLTQTERQKGVQSSGL : 60
Cel.WBGene00003413: GDGHPFSTGLPNVSICAQHTVLVWVPAAFFLLTLPFLSCHLTAQRFARLPFSAHFIIKLLLVAFLAANSLATWCYVLFSKNS--YAAAYYVYPGLWVLVW-TGTFLVHLIRLRGLVSSGI : 117
Aga.AGAP008437: DDDLTWREEDPDLTFCFQRVILQWTPCFFLFVFSMYEVLRIVTSRYRDIPWNWFNITKMIFTFALMVMSWVDLGVVVQNLD---EPEVFILVAIFNALAYIMAMALYFFYRKYGIRSTGT : 117
Aga.AGAP027980: DDEFVWDVDNPNLTFCFQRVILQWVPCLFLFVFSIYDIFKITESKYRDIPWNWYNLSKMLVIFLLMCMCWIDLGMVVGYQDEQGLYDVQILTAVFNALAYIDLLVLLFFMRKYGVRTSGT : 120
Aga.AGAP028128: DGEFVWDVDNPNLTFCFQRVILQWVPCLFLFVFSIYDIFKITESKYRDIPWNWYNLSKMLVIFLLMCMCWIDLGMVVGYQDEQGLYDVQILTAVFNALAYIDLLVLLFFMRKYGVRTSGT : 120
Spu.026395: QKDPSLNASVPDVTPCFQETVLSWIPSAFLVLITPFYLLFMKHNDRGFIRISKLHKAKLSLGLLLILLPVLDVCKGISDMMQTDLPAVYLVTPAVLAITMVVALILIHLERMKGQQSSGV : 120
Hsa.ABCC2.ENSG000000238: FWNSSFDSPEADLPLCFEQTVLVWIPLGYLWLLAPWQLLHVYKSRTKRSSTTKLYLAKQFVGFLLILAAIELALVLTEDSGQATVPAVRYTNPSLYLGT-WLLVLLIQYSRQWCVQKNSF : 119
Xtr.ENSXETG00000026360: -------------SQCFHNSVLNWIPSIFLFICSPFYVLYLRRHGRGYIRMSALSKAKTCLGALLVLVCYTELFYTVWNMTHNVQAPVFLISPLILGSSMLLATCIIQYERMQGVRSSAL : 107
Cel.WBGene00003410: DPAVWNASTVPILSQCYQHTTLVWFPTAIVFLLAPILTAQIFYRRPNPIPWTRRIQLKI----GLACILIADSLSLFTVAIYETQGFPYFVYPLTLCLAMVVLTALIVSCRNYGIVTSGG : 116
Ame.GB53134: NYNLIWNTDDPEITECFQKTVLVWVPCAFLWLFSGIEIYYFLNSKNKNIPYTWLFISKQILIITLILLNIVDLGIAIHKSTYEK---VYYCTPIIRIVTFLKTSILVTYNRKYGMRTSGL : 117
Pma.KM232931_1: ------------------------------------------------------------------------------------------------------------------------ : -
Gac.ENSGACG00000003037: DWNLTWYTSQPDLTQCFQHTVLVWSPCVYLWICSPFYLLYLWLRDRGVIPLSKLCCSKTLLGLSLASFGLVEMLFLLVTKNEEIKHSLIVIGPLIRSLTLVLAVIILHVERMKGCRSSFL : 120
Loc.ENSLOCG00000007152: DWNRTWYTPNPDLTQCFQNTVLVWVPCVYLWVCAPFYCLYLHCYDRGYIRVSCLCCAKMVLGFLLASFGFVEFFYILLERNREIQHLVFLLSPVIRSLTVVLAVLIIQFERVRGSRSSAF : 120
Gmo.ENSGMOG00000005748: DWNRTWYTASPDLTHCFQNTVLVWVPCVYLWLLAPFYCLHLYCHGNGRIRISCLCTAKMVLGFLLASFGFVEFFYILLERSGEIQHMVFLLSPIVRSVTVILALFIIQLERLRGCRSSVF : 120
Gac.ENSGACG00000019172: DWNRTWYTANPDLTQCFQNTVLVWVPCIYLWLLAPFYCLHLYCHDSGRIRMSCLCCAKTMLGFLLASFGFVEFFYILLERSQEIQHMVFLLSPIIRSMTVILALCIIQLERIRGCRSSVF : 120
Tni.ENSTNIG00000012067: DWNRTWYTTNPDLTQCFQNTVLVWVPCIYLWLLAPFYCLHLYCHDHGRIQMSGLFAAKTVLGFLLASFGCVEFFYILLERSQEIQHMVFLLSPIIRSMTVVLVLCIIQLERVRGCRSSVF : 120
Ola.ENSORLG00000013429: DWNRTWYTANPDLTQCFQNTVLVWVPCIYLWLLVPFYCLHLYCHDSGRIRMSCLCMAKMVLGFLLASFGFVEFFYILLERSQDIHHMVFLLSPIIRSMTVILALCIIQLERIRGCRSSIF : 120
Oni.ENSONIG00000018866: DWNRTWYTANPDLTQCFQNTVLVWVPCIYLWLLAPFYCLHLYCHDRGHIQMSCLCSAKMVLGFLLASFGFVEFFYILLERSQDIHHMVFLLSPIIRSLTVILAMCIIQLERIRGCRSSIF : 120
Xma.ENSXMAG00000004906: DWNRTWYTANPDLTQCFQNTVLVWVPCVYLWLLVPFYCVHLYCHDRGRIQMSCLCTAKMMLGFLLASFGFVEFFYILLERSQDIHHMVFLLSPIIRSMTVILALCIIQLERIRGCRSSVF : 120
Dla.DLAgn_00177430: DWNRTWYTANPDLTQCFQNTVLVWVPCIYLWLLAPFYCLHLYCHDRGRIRMSCLCTAKMVLGFLLASFGFVEFFYILLERSQEIQHMVFLLSPIIRSMTVILALCIIQLERVRGCRSSVF : 120
Dre.ENSDARG00000016750: DWNQTWYTHRPELSNCFQNTVLVWAPCIYLWVLSPFYCLHLYCHGRGRLPLSSLCNAKLLLGFFLASFGFVEFFYILLERRLEIQHLVFLLSPIMRSLTVVLAVCVIHWERVRGCRSSVF : 120
Ame.ENSAMXG00000004837: DWNQTWYTHTPELSDCFQHTVLVWAPCIYLWICSPFYCLHLYCHGRGRLPLSLLCSAKLLLGFFLASFGFVEFFYILLERNQEIRHLVFLLSPIVRSLTVVLAVCIIQWERVRGCRSSVV : 120
Ame.ENSAMXG00000003085: NWNLTWFTPNPDLTECFQHTVLVWFPCFYLWLCAPFYFLYLGFHDYGRISVSSLCVTKTVLGLSLAFFSLLELVYLLVSRSGELNHMVFLLSPVIRSLTMVLTVCVIHLERMKGCRSSLF : 120
Dre.ENSDARG00000094901: ---------------------------FYLWICAPFYCLYLKFYYNGRISISSLCCAKMGLALCLASIGFLETVYLLVERSRDIHLMVFLLSPIIRSLTMILVMLMIHLERLRGFRSSVF : 93
Dre.ENSDARG00000095820: DWNVTWYTAHPDLTKCFQHTILVWFPCFYLWICAPFYCLYLKFYDNGRISISSLCCAKTGLALCLASFGFLETVYLLVERRRDIHHMVFLLSPIIRSLTMILAMLMIHLERLRGFRSSMF : 120
Lch.ENSLACG00000022117: DWNQTWFTDRPDFTGCFQITILVWLPCTFLWLCSPFYCWYLQRHGNRYIRMSRLYKAKTVLGAILILLCVSEFFITVWEINQGTRALAFLLSPAILAFTLVLATFLLHYERVKGVQSSGV : 120
Dpu.347281: DLEQTWNTNNPSFTECFHQTVLYWIPCGFIWLLAPYETYQILYSNARYIPWSIFNISKMIINLLLIILSIISIIYAVIQSNNDVTYDVYYVTPAILAATFVLTLGLMLAEKRRGIRSSGP : 120
Lca.KE993868_1: ---------------CFQATVLVWVPCTFLWLAALPYFVWLRHHRQGYIACSVLSRAKT------------------------------------------------------------- : 44
Pma.KM232930_1: NWTLTLHGDPPDVPACFQATVLVWVPCTFLWLAALPYFAWLRHHRQGYIACSVLSRAKTVLCAILWLLCWAKLFFSLWERSQGQLPPIFFISPLILGATMLLAAVLGQVERWSGVHSSAL : 120
Csa.ENSCSAVG00000008135: ------NSTSMIFSSCFQQLVFDLIPCAFLWVSALPYYFYTRRSRVSHIPISALFKAKVVTTIVLWILAWTNLIRSLWEWGQHMVPSVDLVVSLIVGLSMALALLFTQFDRMKGVRSSGL : 114
Csa.ENSCSAVG00000003792: ---------------------------------------------------------------LLWILTWVDLGRGIWEWYNGT--AVLLVTPLIVGVTMAIAAFFIYYDRLKGVRTSGL : 55
Cin.ENSCING00000020698: ------------------------------------------------------------------------------------------------------------------------ : -
Tca.TC012253: NSTLTWHTNDPDLTKCFERTVLVWVPCAFLWTFSSLEVYYILHSKRRDIPWNWLNVSKLLGTAVLFVLTVTDLVTAVNSASSS-ESDVYIYTPVIKLLSFGLSAVLLVYNRKYGLCTSGL : 119
Aga.AGAP009835: DLNLTWYTDDPDLTPCFQQTVLVWAPCAFLWLFSFLELYYLRKSANKDVPWSFVNVSKLVIGSLIVLT-----IVDLAKAISTDSAPLFYYTPVIKLASFIFVAILIYLNKHYGMRTSGL : 115
Lgi.LotgiG107213: DYEVTVNTTDPDFTPCFQKTVLTWTPCVFLIFSAIVRVISLYGVRKHSITWTWLSKVRIFLGALIGIMNLMEFLYSLQEYADNILPVVYFLTPLVVFITMIIYCFMINFERRKGIRNCGF : 120
Bfl.90918: FWNDSF-QESPDLTPCFQQTVLVWVPCFFLLAVAPLYVLYLRRDNRGYIQMSRINKAKT------------------------------------------------------------- : 58
Dme.FBgn0032456: NATETWYTNDPDFTPCFEQTALVWTPCAFYWAFVIFDFYYLKASLDRNIPWNKLNVSKALVNLGLLVITALDLIMALVKKGGDS-LPLYVWGPIIKFATFLLLFIFIPLNRKYGVQTTGC : 119
Oan.ENSOANG00000013379: DWNLTVYSDSPDLTPCFQNTVLAWLPCVYLWTALPCYLLYLKRHQQGYIVLSVLSRIKTGVGVLLWCVSWLDLFYSFHGLVKGILPPVYFVTPLVVGITMLMATMLIQYERLQGVRSSGV : 120
Pma.AKC42143_1: DRNRTLDTHTPDLTACFQNTVLVWIPCAYLWAAAPLYYLHLRRHNRGYIQCTHLNRAKTVIAAVLWLVCWTELFYTMWERDQGRRPPVLLVSPLVMGATVLLAALLVQRERLRGVQSSGV : 120
Lca.KE994284_1: ------------------------------------------------------------------------------------------------------------------------ : -
Cmi.SINCAMG00000015787: DSNLTWHTQDPDFTICFKKTVLIWIPCIFLWLCFPFYTLFLYYRKQGYIRMSNLNKSKTLLGFLLWLSCWSQVLNIILEKSRGFHATVLILGPAFLGVTMLLAVFIIQFERLKGLRSSAV : 120
Ler.ctg13956: ------------------------------------------------------------------------------------------------------------------------ : -
Rty.XP_020377569_1: ------------------------------------------------------------------------------------------------------------------------ : -
Sca.ctg67278: ----------------------------------------------------------------------------------------------------LLAVFLIQYERLKGVQSSAI : 20
Lch.ENSLACG00000001471: DWNQTWYTENPDFTKCFQNSVLVWIPCVYLWVASLFYYLYLRRYGRGYIRMSWLNKTKTVLAVLMWLICWVDIIIFFMERFQKISAPILIISPTVLGATMLLAALLIQYERKKGIQSSGI : 120
Tni.ENSTNIG00000005013: DWNRTWYTDNPDFTQCFQNTVLVWIPCLYLWICGPIYMLYLHSHSHGYICMNHLNKAKTAVCLLLWVLCWSDVFYTFWERSQKSVPLVYLVSPTLLGLTMLLCAALIQSERLKGVQSSGV : 120
Loc.ENSLOCG00000007196: DWNRTWYTPNPDLTQCFQNTVLVWVPCVYLWVCAPFYCLYLHCNDRGYIRMSHLNKAKTVTGFLLWIVCWADVFYSFWERSQGQKAPVYFVSPTLLGITMLLATFLVQYERMKGVQSSGV : 120
Dre.ENSDARG00000104719: DWNRTWQTYYPDLTPCFQNTVLVWIPCLYLWLFAPLYILYLKSHDRGYICMTHLNRAKTVIGFTLWLICWADVFYSFWERSHGAVAPVYLVSPTMLGVTMLLATFLIQYERMKGVQSSGV : 120
Ame.ENSAMXG00000002943: DWNRTWHTHNPDLTQCFQNTLLVVVPCFYLWLFAPFYFLYLKSHDRGYICMTHLNKAKTVTGFLLWIICWADVFYSFWERGHGVSAPVYLVSPTILGITMLLATLLIQYERIKGVQSSGI : 120
Xma.ENSXMAG7738/17319: --------------------------CFYLFACLTVQY-FLNSIMKEYRTRRHKNKSQNVVGFLLWVVCWSDVFYSFWERNHSSTAPVHLVSPTLLGLTMLLATFIIQYERLKGVQSCGI : 93
Ola.ENSORLG00000017141: DWNRSWNTSNPDLTPCFQSTVLVWVPCLYLWLCAPFYLMYMRSHNRGYICMSHLNKAKTAVGFLLWIICWLDVFYSFWERSHSRVAAVHLVSPTLLGLTMLLATLLVQYERMKGVQSSGI : 120
Gac.ENSGACG00000000434: DWNRTWYTTKPDFTQCFQNTVLVWLPCLYLWICAPLYLLYLRSHDHGYIRMSHINRAKTAVGLLLWIICWADVFFSFWERSYGSLAPVHLISPTMLGFTMLLATLLIQYERMKGVQSSGV : 120
Oni.ENSONIG00000007824: DWNRTWYTDNPDFTQCFQNTVLVWLPCFYLWICAPFYLVYLHTHDHGYICMNHLNKAKTAVGFLLWIICWSDVFYSFWERSHVSPAPVRLVSPTLLGLTMLLAVMLIHYERMKGAQSSGV : 120
Dla.DLA_00195360: DWNRTWYTANPDLTQCFQNTVLVWLPCLYLWMCAPLYLLYLRGHDRGYICMSHLNKAKTAVGLLLWIICWADVFYSFWERSHGSPAPVYLVSPTLLGLTMLSAVYV-------------- : 106
Xtr.ENSXETG00000019661: DSNLTWYTENPDFTKCFQNTVLIWIPCIYLWFCLPFYFAYLRKNDQGYIQMSHLNKAKTAIGFILWLACWADLFYSFWERSQSIRAPVYVVSPTVLGITMLLATFLIQYERIKGVQSSGV : 120
Aca.ENSACAG00000005349: DWNLTWNTPRPDFTPCFQNTVLAWTPCAFLWACFPFYAFFLRRHDKGYIQMSRLNKAKTALGFLLWIVCWADLFYSFWERSRNIQAPVYLVSPTILGITMLLATFLIQYERMKGVQSSGI : 120
Gga.ENSGALG00000006646: DWNLTWHTENPDFTQCFQNTVLVWVPCIYLWVCFPAYFLYLRSHDRGYIQMSILNKAKTALGLILWIVCWADLFYSFWERSQNIRAPFFLISPTVLGITMLLATFLIQHERLKGVQSSGV : 120
Dno.ENSDNOG00000014990: DWNVTWHTDSPDFTKCFQNTVLVWVPCCYLWACFPFYFLYLSRHDRGYIQMTHLNKAKTALGFLLWIVCWADLFYSFWERNQGLVAPVFLVSPTLLGITMLLATLLIQLERRKGVQSSGI : 120
Mmu.ENSMUSG00000023088: DWNVTWHTSNPDFTKCFQNTVLTWVPCFYLWSCFPLYFFYLSRHDRGYIQMTHLNKTKTALGFFLWIICWADLFYSFWERSQGVRAPVLLVSPTLLGITMLLATFLIQLERRKGVQSSGI : 120
Ggo.ENSGGOG00000004324: DWNVTWNTSNPDFTKCFQNTVLVWVPCFYLWACFPFYFLYLSRHDRGYIQMTPLNKTKTALGFLLWIVCWAGLFYSFWERSRGILAPVFLVSPTLLGITMLLATFLIQLERRKGVQSSGI : 120
Hsa_ENSG00000103222: DWNVTWNTSNPDFTKCFQNTVLVWVPCFYLWACFPFYFLYLSRHDRGYIQMTPLNKTKTALGFLLWIVCWADLFYSFWERSRGILAPVFLVSPTLLGITMLLATFLIQLERRKGVQSSGI : 120
Ptr.ENSPTRG00000007812: DWNVTWNTSNPDFTKCFQNTVLVWVPCFYLWACFPFYFLYLSRHDRGYIQMTPLNKTKTALGFLLWIVCWADLFYSFWERSRGILAPVFLVSPTLLGITMLLATFLIQLERRKGVQSSGI : 120
Cluf.ENSCAFG00000018208: EWDVSWNTSNPDFTKCFQNTVLVWVPCCYLWLCFPFYFLYLSRHDRGYIQMTYLNKTKTALGFVLWIVCWADLFYSFWERSWGKLAPVFLVSPTLLGITMLLATFLIQLERRKGVQSSGI : 120
Bta.ENSBTAG00000021090: EWNVTWNTSNPDFTKCFQNTVLVWVPCSYLWVCFPFYFLYLSHHDRGYIQMTHLNKAKTALGFLLWIVCWADLFYSFWERSMGKLAPVFLVSPTLLGITMLLATFLIQIERRRGVQSSGI : 120
Mdo.ENSMODG00000004194: DWNITWHTDNPDFTKCFQNTVLVWVPCVYLWACFPFYFLYLCRHNRGYIQMIHLNKAKTALGFLLWIVCWADLFYSFWERSQSIRAPVFLVSPTLLGITMLLATFLIQCERRKGVQSSGV : 120
Oan.ENSOANG00000005124: ------------------------------------------------------------------------------------------------------------------------ : -
Bfl.232174: DYNLL-ESVDPDLTPCFQKTVLVWIPCFFLWAVAPLYYLYLRRHNRGYIQMSKMNKAKTSLAALLVLVTLLDLCYSLWEYTNGVVPAVNFVAPLILAMSMGLAGFFIQYERLKGRQSSVV : 119
Bfl.118638: FWDHNLESVDPDLTPCFQKTVLVWIPCFFLWAVAPLYYLYLRRHNRGYIQMSKMNKAKTSLAALLVLVTLLDLCYSLWEYTNGVVPAVNFVAPLILAMSMGLAGFFIQYERLKGRQSSVV : 120
Bfl.118636: FWDYPLDSADPDLTPCFQKTVLVWIPCFFLWAVAPLYYLFLHRHNRGYIQMSKMNKAKTALAALLVLVTLLDLFRALWEYTNGVVPAVNFVSPLILAMSMGLAGFFIQYERLKGRQSSGV : 120
Bfl.128060: FWDYPLDSADPDLTPCFQKTVLVWIPCFFLWAVAPLYYLFLHRHNRGYIQMSKMNKAKTALAALLVLVTLLDLFRALWEYTNGVVPAVNFVSPLILAMSMGLAGFFIQYERLKGRQSSGV : 120
Dre.ENSDARG00000096662: ELNLTLYGQQPDLPECFQMSVLSWAPCIYLWAAAPLYILHLRRNNRGYIMMSILNRIKTVLGLILWIVCWTDLFSAFHQMNQGSISPIYFVTPLIVGMTMLLATFLIQFERLKGVQSSGV : 120
Ame.ENSAMXG00000016253: KPNLTLSTEIPDLPECFQLSVLAWIPCFYLWLVSPLYLFYLKRNNRGYIMMSILNRVKTVFGFILWIVCWTDLFASFHEIKEKDVPPIYFITPLIVGMTMLLATFIIQFERLRGVQSSGV : 120
Ola.ENSORLG00000020741: DLNTTFHTDQPDLPVCFQLSVLACLPCIYLWVVSPVYLFYLKRNNRGYIMVSILNRFKTVFGLLLWIVCWTDLFYSFHEMQQGQPPPIFFITPLVLGTTMLLATVLIQYERLHGVQSSGV : 120
Gmo.ENSGMOG00000010029: ------HTDRPDLPACFQLTVLSWLPCIYLWAVTPLYIFYLKRNNKGYIMMSVLNRFKTAFGFVLWLVCWSDLFYSFHELRNGLRPPIYFVTPLVVGMTMLLVTFLIQFERLRGMQSSGV : 114
Xma.ENSXMAG00000012203: EPNQTFHTDRPDLSECFQLSVLAWLPCVFLWVASPAYICYLKKSSRGYIMMSLLNRFKTAFGLLLWIVCWADLFYTFHELQQGQPPPIYFITPLVLGITMLLATFLIQYERLHGAQSSGV : 120
Gac.ENSGACG00000005901: ------HTDQPDLPQCFQLSVLSWLPCVYLWAVCPIYLFYLKRNHSGYIMMSILNRFKTVLGLLLWIVCWTDLFYTFHELRQDDQPPIYFITPLVLGMTMLLATFLIQFERLRGVQSSGV : 114
Oni.ENSONIG00000019586: VSNRTFNSSWPDLPECFQLSVLSWVQCIYLWAVSPIYIFCLKKNKKGYIMMSLLNRFKTAFGLLLWIVCWTDLFYTLHEGK---QPPIYYVTPLVLGMTMLLATFLIQFERLRGVQSSGV : 117
Dla.DLAgn_00098120: VANQTLHTDRPDLPECFQLSVLSWLPCIYLWAVFPIYLFYLKKNNRGYIMMSIMNRFKTAFGLLLWIVCWTDLFYTFHELRQGHQPPIYFVTPLVLGMTMLLATFLIQFERLRGVQSSGV : 120
Cmi.SINCAMG00000013336: ----------------------------------------------------------------------------------------------------LLAVGLIQYERLRGVRSSGL : 20
Ler.ctg12190: ----------------------------------------------------------------------------------------------------LVATLLIQCERLRGVQSSAV : 20
Sca.ctg14163: ------------------------------------------------------------------------------------------------------------------------ : -
Rty.XP_020375725_1: ------------------------------------------------------------------------------------------------------------------------ : -
Sac_EU250283: DHNVTFNTDSPDLTPCFQKTILVWIPCVYLWVTFPLYFLYLRQSSRGYIRVTVLNRVKTVLGVLLWIVCWSDLFYSMDEMANQKRALAYFISPLLLGVTMLLATFLIQYERLRGIQSSGV : 120
Tni.ENSTNIG00000004171: -ANETLHTDRPDLPKCFQLSVLAWLPCIYLWVACPIYLFYLKRNNKGYIMMSIMNRFKTVFGLLLWIVCWMDLFYTFHMLRQGHQPPIYFVTPLVLGMTMLLATFLIQFERLHGIQSSGV : 119
Aca.ENSACAG00000001396: DPNLTLYTDNPDLTPCFQNTVLGWIPCFYLWCILPFYLFYLKCNNRGYIVLSVLSRFKTVFAFLLWCVSWANLFYSFHGITQSRPPSVYFVTPLIVGITMLLAMLLIQYERLHGVQSSGI : 120
Gga.ENSGALG00000007522: ----------------------------------------------------MLSRFKTLFGVLLWCVSWADLFYSFHELLQSRPPPVYFVTPLVIGITVLLATLLIQYERLRGVQSSGV : 68
Xtr.ENSXETG00000012239: DSNLTLYTDNPDVTPCFQNTVLAWIPCVYLWVALPFYLLYLKYNRRGYIVLSMLSKAKTVFGVLLWCVCWADLFYSFHGLVQNQPPPVYFVTPLILGITMITATVLAQYERLRGVQSSGV : 120
Loc.ENSLOCG00000010918: DSNLTLHADSPDLPDCFQKSVLSWVPCIYLWLALPAYVLYLKRNNKGYIMMSVLNRARTVFGVLLWITCWIDLFYTFHELHQGHQPPVYFVSPLLVGITMLLATFLIQYERLKGMQSSGV : 120
Lch.ENSLACG7209/6619: IYRLKKNTENPDLTTCFQNTVLAWIPCIYLWITFPIYHLYLKCNNKGYLVVSVLNRLKT---IFLIAKLCINLFYSFYEFTRALTAPAQYISPLIVGTTMLLATLLIQYERLRGVQSSGV : 117
Mdo.ENSMODG00000020910: DSNLSLYTDKPDLTPCFQNSLLAWVPSIYLWISLPCYLLYLRHQNQGYIILSGLCRLKTVIGILLWCVTWADLFYSFSGLVKGSPAPIYFVTPLIMGITMLLATLLIQYERLRGVRSSGI : 120
Dno.ENSDNOG00000046300: DSNLSVYTDNPDLTHCFQNSLLAWTPCIYLWAMLPCYLLYLQRHQCGYIVLTHLCRLKTALGVLLWCVSWADLFYSFLGLAEGSPAPIFFVTPLVLGVTMLLAALLIQLERLRGVQSSGV : 120
Mmu.ENSMUSG00000020865: DSNLSIYTNTPDLTPCFQNSLLAWVPCIYLWAALPCYLFYLRHHQLGYIVLSWLSRLKTALGVLLWCVSWVDLFYSFHGLIHGSPAPVFFVTPLVVGITMLLATLLIQYERLRGVQSSGV : 120
Ggo.ENSGGOP00000003354: DSNLSVHTENPDLTPCFQNSLLAWVPCIYLWVALPCYLLYLRHHCRGYIILSHLSKLKTVLGVLLWCVSWADLFYSFHGLVHGRPAPVFFVTPLVVGVTMLLATLLIQYERLQGVQSSGV : 120
Hsa.ENSG00000108846: DSNLSVHTENPDLTPCFQNSLLAWVPCIYLWVALPCYLLYLRHHCRGYIILSHLSKLKMVLGVLLWCVSWADLFYSFHGLVHGRPAPVFFVTPLVVGVTMLLATLLIQYERLQGVQSSGV : 120
Ptr.ENSPTRG00000009406: DSNLSVHTENPDLTPCFQNSLLAWVPCIYLWVALPCYLLYLRHHCRGYIILSHLSKLKTVLGVLLWCVSWADLFYSFHGLVHGRPAPVFFVTPLVVGVTMLLATLLIQYERLQGVQSSGV : 120
Cluf.ENSCAFG00000017201: DSNLSVHTDNPDLTPCFQNSLLAWVPCIYLWAALPCYLFYLQRHNRGYIVLSHLSRLKTALGVLLWCVSWAELFYSFHGLVHGWPAPISFVTPLLVGVTMLLATLLIQYERLRGVQSSGV : 120
Bta.ENSBTAG00000020070: DSNLSLHTDNPDLTPCFQNSLLAWLPCIYLWAALPCYLFYLRGHRQGYIVLSHLSRLKTALGVLLWCVSWADLFYSFHGLVHGWPAPVFFVTPLVVGVTMLLATLLIQYERLQGVRSSGV : 120


Hsa.CFTR.ENSG0000000162: ----------------------------------------------------------------------SPLEKASVVSKLFFSWTRPILRKGYRQRLELSDIYQIPSVDSADNLSEKL : 53
Hsa.ABCC5.ENSG000001147: ---------LSALKPIRTTSKHQ-----------------------------------------------HPVDNAGLFSCMTFSWLSSLAVAHKKGELSMEDVWSLSKHESSDVNCRRL : 143
Hsa.ABCC11.ENSG00000121: ---------LRTMIPFRPK---------------------------------------------PRFPAPQPLDNAGLFSYLTVSWLTPLMIQSLRSRLDENTIPPLSVHDASDKNVQRL : 127
Hsa.ABCC12.ENSG00000140: ----------------------------------------SFAERYDPSLKTMIPVRP------CARLAPNPVDDAGLLSFATFSWLTPVMVKGYRQRLTVDTLPPLSTYDSSDTNAKRF : 93
Hsa.ABCC8.ENSG000000060: LLVYWTLAFITKTIKFVKFLDHAIGFSQLR--FCLTGLLVILYGMLLLVEVNVIRVRRFFKTPRVKPPEDLQDLGVRFLSKGTYWWMNAFIKTAHKKPIDLRAIGKLPIAMRALTNYQRL : 236
Hsa.ABCC9.ENSG000000694: LFLYWVMAFITKTIKLVKYCQSGLDISNLR--FCITGMMVILNGLLMAVEINVIRVRRFFMNPQVKPPEDLQDLGVRFLSKATYWWMNTLIISAHKKPIDLKAIGKLPIAMRAVTNYVCL : 236
Hsa.ABCC10.ENSG00000124: ALVALLPAPALVLTVLWHCQRGTLLPPLLPGPMARLCLILQLAALLAYALGWAAPGGPPWAQEPLPEDQEVAEDGESWLSRFSYAWLAPLLARGACGELQPQDICRLPHRLQPTYLARVF : 236
Hsa.ABCC4.ENSG000001252: -----------------------------------------------------------------QEVKPNPLQDANLCSRVFFWWLNPLFKIGHKRRLEEDDMYSVLPEDRSQHLGEEL : 55
Cel.WBGene00003414: CLIFLILLVLKAVQVLVLVFESTFDAGVTA----------EFVLLMAETALLASSTKR--LGEDEQKIEQTPEEKSSFLSKIFFCWLNPLIRAGAKQPLTNESLHNLNENATSEWLYTRW : 203
Cel.WBGene00003409: LFCIWMLFAVAAVPEFYQWMTTGSQPELVADFFRYVAYLTYFPLVVAEFVLHFVSD--PFPMPRYQNLK-CPEENANFISRQLLLWFTQIISLGYERTLVADDVFEMDSQMDQEYLKARW : 235
Hro.HelroG163344: HFLFYFLCLIAVLFNMVSIVSRA-----------------------------------------VRPRKQCPEAQASFLSRMTFFWITGLIYEGYKKTLKESDLWGLHPRDVTRSNANVL : 199
Hro.HelroG157076: ---------------------------------------------------------------------------MTF-------------I---------------------------- : 4
Bfl.230771: LSTFWLLSSLCGAVTARDKLRQMMVQDAVEDKFRFMTFSLNFSMTVLQLVLSTVSHAS---STEVRPIISSPEESSSFLSRITFLWFTPLVMLGYKRPLKAADLYPLKTEDTSRHLVPKF : 227
Lgi.LotgiG105097: LYLFWLFYIVANIIPFYTLIVQKVYEK---NFVKFVLFYINYGILMLQLVLHSFSESTTVHDEK--QKEPCPMQTASFPSKIFFMWANRLIVKGYKKPIKDEDVFDLPEEFKSKHQAPPF : 226
Cel.WBGene00003407: ITLYWLLVVVCGVPELRYYITGRLYKEYEIHSCRAALYVFAYVCSALELFLSCFADTP---SNGYIGKNSCPEYTASFLNQLTFQWFSGLAYLGNKKSLEKEDLWDLNERDKAENIIPSF : 235
Cel.WBGene00003408: ITLYWLLVVVCGIPEFRFYLSGFIYNEYALEGIRATLYIIAFTFSALELFLCCFADVP---SDMYKSESSCPEYTASFINRLTFQWFTGLAYLGNKKSLENEDLWDLNEIDKAENLIPSF : 234
Lgi.LotgiG153611: VFIFWFLLSVAGIIPLYTYLILKTYNE---GPVRFSGYILYYTLVLTQLILNCFAEKR-------------TVNSTEY--RDTTW--RHLIYKGFKAPLNDHDLFDLHPRDDSKRVANEF : 189
Lgi.LotgiG110718: -----------------------------------------------------------------------PEVTASLPGQLTYSWMFPLILNGYRKGLNEGDVWQLNPRDASSRLVPQF : 49
Dno.ENSDNOG00000024923: LFGYWL---LCCLLPATNTAQLVLRGDFQRDAFRHLSTLLCLSLVAVQLVLSCLADWPPFFPKAPQQPNPCPEAGASFPSKATFWWVSGLVWRGYRRPLGPKDLWSLGKESSSEELVSRL : 178
Mmu.ENSMUSG00000030834: LFGYWL---LCCILPGINTVQQASAGNFRQEPLHHLATYLCLSLVVAELVLSCLVDQPPFFSEDSQPLNPCPEAEASFPSKAMFWWASGLLWRGYKKLLGPKDLWSLGRENSSEELVSQL : 237
Cluf.ENSCAFG00000018197: LFGYWM---LCFLLPITSTAQLTLQGDFRSDPFSHLATYLCLSLVAAQFVLSCLVDQPPFFPKDPQQSNPCPKAEASFLSRAMFWWVSGLVWRGYRRLLGPEDLWSLGRENSSEELVSQL : 237
Bta.ENSBTAG00000015191: LFGYWL---LCFLFPATSATQQASRGDFQSDPFRHLSPYLYLSLVMAQFALSCLADQCPLFRKRPPQANPCPKAGASFPSKAMFWWVSGLVWKGYRRPLGPKDLWSLGSKNSSEELVSQL : 237
Ggo.ENSGGOG00000009623: LFGYWL---LCFVLPATNAAQQASGAGFQSDPVRHLSTYLCLSLVVAQFVLSCLVDQPPFFPEDPQQSNPCPETGAAFPSKATFWWVSGKR--------KKKSAWALEAESWNQIPVLLL : 229
Hsa.ENSG00000091262: LFGYWL---LCFVLPATNAAQQASGAGFQSDPVRHLSTYLCLSLVVAQFVLSCLADQPPFFPEDPQQSNPCPETGAAFPSKATFWWVSGLVWRGYRRPLRPKDLWSLGRENSSEELVSRL : 237
Ptr.ENSPTRG00000007815: ------------------------------DPVRHLSTYLCLSLVVAQFVLSCLADQPPFFPEDPQQSNPCPETGAAFPSKATFWWVSGLVWRGYRRPLRPKDLWSLGRENSSEELVSRL : 94
Oan.ENSOANG00000005123: LFVYWLLSFLVTLVTLSATVQHALQGGFPRDAFRHIVSYLYSALVGAQFVISFFADQPPFFAKVPQESNPCPESGASFPSKVTFWWFSRLVWQGYRRPLEPDDLWSLQRENSSEELVSQL : 190
Mdo.ENSMODG00000005815: LFIYWLLCSFSMAVTVSATVHQALQGGFPEDTFRHLITYFHSALIGAQFVLSFLADQPPFFSKIMHDSNPCPESGASFPSKVTFWWFSRLVWQGYRKPLEMDDLWSLGKENSSEEIISRL : 240
Gga.ENSGALG00000006698: MLIYWLLTFLSALVMFSSKIQRGLERGFLEDFFHHVATYLYASLVLGELVLFCLVDHPPFFSKAVNSSNQCPEASSSFLSKITYWWFSGLVWKGCRQSLGVDDLWSVRKEDSSEEIVAWA : 240
Aca.ENSACAG00000003478: LLLYWLLSFLSATASLISKIQEAREGGFRSAPFHHATSYIYFTLVSLELGLCCLVDQPPFFSKVDSDANPCPESRASFLSRITFWWFAGTIWKGYWKPLQREDLWSLAKENSSEEIVAKF : 180
Cel.WBGene00003413: QHVTSLIFLLCGAPEFYQWIRMENSNSFPNAQFLSIAYLSWYSALILYTFSLCFADPRGAKTDDKASSKSSPELQSSFLNRLTLWWFNSIPWTGARRDLEIDDIFELNERSGTEFLSELW : 237
Aga.AGAP008437: MFIFWFLKAFFGIIQMRTEAMLHDVRGSGTAEFQFVSYTIQYTFVCCVLLLELFPDKEPRYSEWAKLKNPNPELRSSFFSRLFYLYFDSYAWRGFRKPLTDDDMYDLNPEDTSRALVPPF : 237
Aga.AGAP027980: MFMFWFLRMFFGIIQLRTEVMENDKRPNAIWEYQYVSYILQYSLICLMLVLELFPDKEPTFSYYPKSKNPNPELKSSFFAKLLFLYFDTFAWKGFRKPLTMEEMYDINPQDTSRELVPPF : 240
Aga.AGAP028128: MFMFWFLRMFFGIIQLRTEVMENDKRPNAIWEYQYVSYILQYSLICLMLVLELFPDKEPSFSYYPKAAKPNPELRSSFFSKLLFLHFDAFAWKGFRNPLTMNDMYDINPQDSARELVPPF : 240
Spu.026395: LFIFYLISILCAVAEFHSKAINAKDQGSE-DAFRYVTFYLYFFLLIGQLVLVTFADQMPYNSPDIAETNPCPEARASFLSVITFWWFTSMVIKGYKKSLEKADLWSLLTRDKASRVVPEF : 239
Hsa.ABCC2.ENSG000000238: LSLFWILSILCGTFQFQTLIRTLLQGDNSN--LAYSCFFISYGFQILILIFSAFSE--------NNESSNNPSSIASFLSSITYSWYDSIILKGYKRPLTLEDVWEVDEEMKTKTLVSKF : 229
Xtr.ENSXETG00000026360: LLFFWLLALLCATFQLRTKITTAISEI---DKLRYTLFVLYFVFVLAQSVLCTFNDDPPFFSNLKKESNPCPVSESSFLSKVTFSWFTEIMFRGYKQPLKAEDVWSLRKSDTAEEILTLF : 224
Cel.WBGene00003410: LFISWLVFTISAIPELLYWIQQIVNPAEAWDYPRCIAFFIWFFCCAFETYLHCYADASPEGYKYSAARNPSPETTSSFLNRITMWWFNSLCSLGVKKPLEVSDLYSLNEADTSNLLVPKW : 236
Ame.GB53134: LFLFWFLLALCGIIEYRSLLKLYINKNEIS--YSFISYMIYYPIVIFLFLLNFLVDAEPKYSKYPRAEKPCPEQKSSFPGKIFFSWFDSMAWKGFKKPLEITDLWSINPEDTAKEIVPKF : 235
Pma.KM232931_1: -------------------------------------------------------------------------------------------M---------------------------- : 1
Gac.ENSGACG00000003037: LFQFWILLVLCSLVPLKVDIEQIIDRGFSSDSSRLLLFFLCFFLQLIQLVLSCFCDLRPLCAKQSYVQNRCPEEDASFLSNFFFSWFSGLVVRGYRHPLQAADLWPLRDQDSSIRIMTDF : 240
Loc.ENSLOCG00000007152: LFLFWLLAVVCSLVPLRAKIQLAIDEGFSADAVRYLAFFSYFTLQLAQLFLSCFSDQPPYTRRPVKVPNPCPVQDASFLSKILFWWFSGLVVKGYRKPLKAEDLWSLREEDRSDRIISDL : 240
Gmo.ENSGMOG00000005748: LFLFWVLAVVCSLVPLRAKIQLAVDEGIGSDVVRFLAFFSYFTLQLAQLVLVCFADRR----------NPCPVKDASFLSKILFWWFTGPDR-------QEKVLASGTALGAKLPDQAQL : 223
Gac.ENSGACG00000019172: LFLFWVLAVVCSLVPLRAKIQLAMDEGISSDIVRYFAFFSYFTIQLAQLFLCCFADKPGSTSKSAGDENPCPVKDASFLSKILFWWFTGLVVKGYRTPLEAEDLWTLREEDTSRKIIAEL : 240
Tni.ENSTNIG00000012067: LFLFWVMAVVCSLVPLRAKIQLAMDEGFASDIVQYFAFFSYFTIQLAQLFLCCFADQPPVGKT-ILEKNPCPVKDASFLSKLLFWWFTGLVVKGYRNPLAAEDLWTLREEDTSCKIIAEL : 239
Ola.ENSORLG00000013429: LFLFWVLSVVCSLVPLRAKIQLAVDEGIASDIVRYLAFFSYFTIQMAQLFLCCFADQPPQGKP-NLEKNPCPVKDASFLSKILFWWFTGLVVKGYRTPLEATDLWTLREEDTSHKIISDL : 239
Oni.ENSONIG00000018866: LFLFWVLAVVCALVPLRAKIQLAMDEGIASDIVRYLAFFSYFTIQLAQLFLCCFADQPPEGKI-ISEKNPCPVKDASFLSKILFWWFTGLVVKGYRTPLEAGDLWTLREEDTSQKIISDL : 239
Xma.ENSXMAG00000004906: LFLFWVLSVVCSLVPLRAKIQLAIDEGIASDIVRYLAFFSYFSIQLAQLFLCCFADRPPQGKP-VLEKNPCPVEDASFLSKILFWWFTGLVVKGYRTPLEAEDLWTLRKEDTSHKIISEL : 239
Dla.DLAgn_00177430: LFLFWVLAVVCSLVPLRAKIQLAMDEGIASDIVRYLAFFSYFTIQLAQLFLCCFADQPPEGKT-ILEKNPCPVKDASFLSKILFWWFTGLVVKGYRTPLAAEDLWTLREEDTSNKIISEL : 239
Dre.ENSDARG00000016750: LFFFWLLGVLCSIIPLHAKVQLAVEQGLSPDIVRYLAFFSYFALQLAQLFLSCFADQAPLGKAV--HKNACPVQDASFLSKILFWWFSGLIFKGYRSPLQAEDLWSLREEDTSERIISDL : 238
Ame.ENSAMXG00000004837: LFLYWLLGVICSLVPLRAKIQLAVEQGFSPDIVHYLAFFAYFALQLAQLFLSCFADQA---PPGVALKNACPVQDASFLSKLLFWWFRGLVVKGYRTPLQAEDLWSLREEDTSDKIISDL : 237
Ame.ENSAMXG00000003085: LFVFWTLAVVCSLVPLRANIQAVVGESCSRDSVRSAAFFTCFSLQLAQLILSCFADQRSDDLKWVDVKNPCPVEDASFLSKILFWWFSGLVVKGYRSPLKAEDLWSLRKEDTSEKIIGDL : 240
Dre.ENSDARG00000094901: LFLFWMLSVVCSLVPLRANIQANIKEGFSADPMRFAAFFTFFSLQLAQLILSCFADQRPDTLKPVYVKNPCPVEDASFLSKLLFWWYGRLVVKGYRSPLKAEDLWSLREEDTSEKIICDL : 213
Dre.ENSDARG00000095820: LFLFWMLAVVCSLVPLRANIQAIIEEGFSADAMRFVAFFTFFSLQLAQLILSCFADQRPDTLKPVYVKNPCPVEDASFLSKLLFWWYGRLVVKGYRSPLKAEDLWSLREEDTSEKIICDL : 240
Lch.ENSLACG00000022117: LLFYWLLSLLCAVFPFRSKIQQAPPDGSVIHSFRFGMFYSYFAMVLAQLILCCFTEPPPFFSEERKAPNPCPESNASFLSKVTFWWFTGQVIKGYRRPLVAEDLWSLRKENRSDEIVRHL : 240
Dpu.347281: LFMFWFLLTLCGGFTYADRIKSIVDGMDSLETYPFVWEMVYYPFVVSMLFINCFADKEPLYMEGGKSENPCPEEGSSFLNVITYSWLDTLVWKGYRKPLETGDLWDLNSRDKSKSVVPRF : 240
Lca.KE993868_1: -------------------------------------------------------------------------------------------V---------------------------- : 45
Pma.KM232930_1: LFVFWLLVLVGAITGTRSSVLHAVRLGMLEDPFRDTVFLMYSVLVLVQFILVCLAEKRPLFSTDSDDPNLCPEVGASFISRLTFCWFTRMAVNGYRRGLKMSDLWSLRHKDSCSFVVPHL : 240
Csa.ENSCSAVG00000008135: LTCYWLLTLLTGVFIFQSKVLLLLSSDTDGQILRCVTFFLSFVALLINFVLCFFADNMPAFKPKNAE---SPEVAATFLSKISFWWFTRMVVMGYKRPIINDDLWKLNEDDESKNIAKSF : 231
Csa.ENSCSAVG00000003792: LTIYWILFLLTWALVFRTKVEQIQNGGLTTEVMRIVTFFISYACVIAHFLMSFFVDLPPAYE--PQKLNTSPQESASFISHMFFEWFSKMIMLGYKNPLVDNDLWDLIPDDKSGRISRRF : 173
Cin.ENSCING00000020698: -------------------------------------------------------------------------------------------M---------------------------- : 1
Tca.TC012253: QFLFWLSLAICGAFQYRTELRGSQEEIPE-SRYSYISYLIYYPVVLVMLFLNCFADRSPRRSEYPKLKNPCPEENSSFLSRLLFSWFDPLAWRGFRRPLVDSDLWDMKPEDSASEVVPTF : 238
Aga.AGAP009835: LFLFWFLLTVCSIPRVRTEIRAYEARVVEDAEYQFVSFLIFFSLTCIMFLLNFFVDKPPRQSKYEITDKDCPELAASFPSRIFFAWFDRLAWVGFRKPLEVDDLWKMKPEDSSKEVSPAF : 235
Lgi.LotgiG107213: MFVFWLLYTICGLVIFRSKIRHAVLLNEVRDEVRFSIFIVCYPIVVVQLILSIFVDKQPKNLPDPQIS--CPENSASFISKITFWWFTQLVIEGYKKALERKDLWSLNYVDTSSHIVQRF : 238
Bfl.90918: ------------------------------SRVLFLT------------ISSFLPQNR------------SPKETSSFLSRMTFLWFTPLVILGYKRALVMTDLHTLSDSNKAENIAPGF : 124
Dme.FBgn0032456: QFIFWFLLTVLSIPRCRTEVRLDAERQKILEEYQFVSFFIFFTFTSIMLILNCFADGMPRQTKYQRGENEIPELSASFLSRITYQWFDKMALKGYRNPLEEKDLWDLRPQDSCSEVMPIF : 239
Oan.ENSOANG00000013379: LFVFWFLCVLCALIPFRSKIIVATAQDGVRDQFRFTTFYLYFFLILVELVLSCFREQPPLFSSAKPSPNPCPEANAGFLSRLTFWWFTRMAILGYRRPLEEKDLWSLNEDDTSQVVVQRL : 240
Pma.AKC42143_1: LLVFWLLALVCGIVPFRSKILAVAREDPDVDRFRYATFYVYFVLVCVQFVLSWFSDRPPLFSRVIKDPNPCPEISASFLSRLTFWWFTSLAILGYQRPLESKDLWSLNPEDRSAVMVPRL : 240
Lca.KE994284_1: --------------------------------------------------------------------NPCPEISASFLSRLTFWWFTRLAILGYQRPLESKDLWSLNPEDRSAVMVPRL : 52
Cmi.SINCAMG00000015787: MFLFWLLTLLCSTIEFRSTVMNLLYPPAHFDLVDHIIFFFNFTMVLAEFVLCCFTDDPPFFQL-ILSTNPCPESKASFLARITFWWFTELTILGYKRPLEAKDLWSLNENDTSEKIVPEL : 239
Ler.ctg13956: ------------------------------------------------------------------------------------------------------------------------ : -
Rty.XP_020377569_1: ------------------------------------------------------------------------------------------------------------------------ : -
Sca.ctg67278: LLIFWLLALLCAIPTLRSKIMHSIDQVSYIDVFRDSTFYLYFVLVLIELVLSCLTDQPPLFSESVKDSNLCPELSASFLSRITFWWFTGMTVLGYKRPLEPGDLWSLNRKDKSKTIVPQL : 140
Lch.ENSLACG00000001471: LMIFWLTALICATVTFRSKIIYALQKDADVDVSRYVTFYVYYMLLLVQLVLSCLTDQPPLFSEEVKDENPCPEYSTSFLSRITFWWITGMMIQGYKQPLEAKDMWSLNKEDTSAQVVPLL : 240
Tni.ENSTNIG00000005013: LFIYWLLALLSATFILRSKILHALEQSLTAFPWRHTTFYIYYGLLLAAFVLSCLTDQPPLFCAVVKNSNPCPEPGASFLSRITFWWITNFIITHFIANILKKSFFSVVREQKKNIYSSKV : 240
Loc.ENSLOCG00000007196: LLNFWVIAVICGTISFRSKILQAFSETSGVDLFRYFTFFTYFALLLIQLFLSCLSDQPPLFSQAVKDSNPCPELGASFLSRITFWWITGLMVQGYRRPLEERDLWSLNEEDKSQKVVPQL : 240
Dre.ENSDARG00000104719: MLNFWLITIVCATITFRSKIMHALNDPASVGVFRYTTFYIYYTLLLISLILACLSDQPPLFSEVVKDSNPCPESGASFLSKITFWWITGLMVKGYKRPLEEKDLWSLNNEDKSERVVPQL : 240
Ame.ENSAMXG00000002943: MLNFWLVATVCATVTFRSKILQAVNEPETVNVFRYSTFYIYYALLLISLILSCLSDQPPLFSQAVKDSNPCPEAGASFLSRISFWWITGLMVQGYKRPLEEKDLWSLNAEDRSHTVVPQL : 240
Xma.ENSXMAG7738/17319: LLIFWLLALLCATVSFRSKILQARNEPETVCIWKYTTFYIYYAFLLVALILSCLTDQPPLFSQATKELNPCPEPGASFLSRITFWWITGMMMVGYRRPLEEKDLWSLNPDDCSHRVVPQL : 213
Ola.ENSORLG00000017141: MLIFWLLALLCASVTFRSKILQAQDQPEAVSGWRYTTFYVYYALLLLALVLSCLSDQMPLFSQAVKDPNPCPEPGASFLSRITFWWISGMMLSGYKRPLEEKDLWSLNPEDRSHCVVPQL : 240
Gac.ENSGACG00000000434: MLLYWLLALLCATVTFGSKISRALDQPLTVSVWRYTTFYTYYALLLVSLCLSCLTDQLPLFSEAVKDSNPCPERGANFLSRITFWWITRLMVTGYRRPLEEKDLWSLNSADRSHKVVPEL : 240
Oni.ENSONIG00000007824: MLIYWLLALLCATVTFRSKIFQALEQPQTVCVWRYTTFYIYYALLLIALFLSSLTDQPPLFSRDVKDSNPCPEPGASFLSRITFWWITRMMMTGYRRPLEEKDLWSLNAEDCSHRVVPQL : 240
Dla.DLA_00195360: --------------------------------WRYTTFYIYYALLLVALFLSCLTDQPPLFSQAVKDSNPCPELGASFLSRITFWWITNMMMTGYKHPLEEKDLWSLNPEDRSHRVVPQL : 194
Xtr.ENSXETG00000019661: MLNFWIVALLCAIIIFRSKVLHALKPDAQIDVFRDTTFYIYFLLVLVELILSAFPDRPPLFSERVNDPNPCPESSASFLSQITFWWISRMMVQGFKRPLEAKDLWSLNKEDKSLEVVPVL : 240
Aca.ENSACAG00000005349: MLLFWLIALLCASVTFRSKILQASDAVKGFDVFRCITFFLYFALVLTELVLSCFPERPPLFSETVHDPSNRPRIKTNVLKSMVLWEIKGLLFSGYQNILEMAFVFHLNSSLPSEELVVVK : 240
Gga.ENSGALG00000006646: MMIFWLISLLCATVIFRSKIMLALNTDTEVDAFRYVTFCTYFILLLVQLILSCFPEKPPLFSEAVNDPKPCPEFSASFLSRITFWWITGLMIQGHRRPLEAKDLWSLNKEDTSEEIVPGL : 240
Dno.ENSDNOG00000014990: MLTFWLVALLCATAILRSKIMTALKKDAGVNVVRDVTFYIYFSLVLIQLVLSCFSDRSPLFSETINDPNPCPETSASFLSRITFWWITGLVVRGYRQPLESTHLWSLNREDTSEEVVPVL : 240
Mmu.ENSMUSG00000023088: MLTFWLVALLCALAILRSKIISALKKDAHVDVFRDSTFYLYFTLVLVQLVLSCFSDCSPLFSETVHDRNPCPESSASFLSRITFWWITGMMVHGYRQPLESSDLWSLNKEDTSEEVVPVL : 240
Ggo.ENSGGOG00000004324: MLTFWLVALVCALAILRSKIMTALKEDAQVDLFRDITFYVYFSLLLIH---------------------------ASFLSRITFWWITGLIVRGYRQPLEDSDLWSLNKEDTSEQVVPVL : 213
Hsa_ENSG00000103222: MLTFWLVALVCALAILRSKIMTALKEDAQVDLFRDITFYVYFSLLLIQLVLSCFSDRSPLFSETIHDPNPCPESSASFLSRITFWWITGLIVRGYRQPLEGSDLWSLNKEDTSEQVVPVL : 240
Ptr.ENSPTRG00000007812: MLTFWLVALVCALAILRSKIMTALKE---------ITFYVYFSLLLIQLVLSCFSDRSPLFSETIHDPNPCPESSASFLSRITFWWITGLIVRGYRQPLEGSDLWSLNKEDTSEQVVPVL : 231
Cluf.ENSCAFG00000018208: MLTFWLIALLCALAILRSKIMTALKEDAEIDVFRDVTFYIYFSLVLIQLVLSCFSDRPPLFSETIHDLNPCPESSASFLSRVTFWWITGLMVRGYRQPLESTDLWSLNKEDTSEQVVPVL : 240
Bta.ENSBTAG00000021090: MLTFWLIALLCALAILRSKIMTALKEDARVDVFRDVTFYIYFSLVLIQLVLSCFSDRSPLFSETINDPNPCPESSASFLSRITFWWITGMMVQGYRQPLESTDLWSLNKEDTSEQVVPVL : 240
Mdo.ENSMODG00000004194: LLTFWLLALLCAGIVFRSKVLHALRASDKIDIFRDVTFYIYFLLVLAQLILSCFSDHSPLFSETINDPNPCPESGASFLSRITFWWISGLMVQGYKCPLEATDLWSLNREDTSNQVVPVL : 240
Oan.ENSOANG00000005124: -------------------------------------------------------------------------------------------VQGYKRPLEASDLWSLNREDTSDQVVPVL : 29
Bfl.232174: LFLFWLLASLCGIVTFRSNIRVALLQVRVSDMFRFVTFYLYFPLVLTELILSAFSERAPLFSEANRDPNPCPEESSSYLSKITFWWFNPLVILGYKRALERADLYSLNHVDRSDHVVPVF : 239
Bfl.118638: LFLFWLLATLCGIVTFRSNIRVALLQGNVSDTFRFVTFYLYFPLVLTQLILSAFSERAPLFSEASRDPNPCPEESSSYLSKITFWWFNPLVILGYKRALERADLYSLNHVDRSDHVVPVF : 240
Bfl.118636: LFLFWLLATLCGIVTFRSNIRVALLEGGVSDTFRFVTFYLYFPLVLTELILSAFSERAPLFSEANRDPKPSPEESCSFLSKITFWWFNPLVILGYKRALERADLYSLNHVDRSDHVVPAF : 240
Bfl.128060: LFLFWLLATLCGIVTFRSNIRVALLEGGVSDTFRFVTFYLYFPLVLTELILSAFSERAPLFSEANRDPKPSPEESCSFLSKITFWWFNPLVILGYKRALERADLYSLNHVDRSDHVVPAF : 240
Dre.ENSDARG00000096662: LFIFWTLSVVFAIVPFRSKIMHANTEVK--DKLRFTTFYIYFSFTLLELILSCFNEKPPLFSSAVMDPNVCPETTAGFLSRMTFWWFTRMAIKGYKSPLENKDLWSLNKNDSSELVVPGL : 238
Ame.ENSAMXG00000016253: LFIFWTLSVLCAIVPFRSKILHANKGENV-DKLRFTTFYMYFGLILVELIMCCFNEKPPLFSSVVTDPNPCPETTAGFLSRMTFWWFTSMAIKGYKSPLETKDLWSLNKQDSSELVVPKL : 239
Ola.ENSORLG00000020741: LFIFWFLSVLCAIVPFRSKILKASGESEVPDKLRFTTFYVYFSLVVCELILCCFNERPPLFSDTVTDPNPCPETTAGFLSSMTFWWFTSLALKGYKMPLEAKDLWSLKKRDSSETMVPRL : 240
Gmo.ENSGMOG00000010029: LFIFWFLSLLCAIVPFRSKILQASST----DKLRFTTFYLYFSLVLLELILSCFNEKPPLFSTVVTDPNPCPESTAGFLSTITFWWFTRMAVKGYKTPLEAKDLWSLNVQDSSQTVVPRF : 230
Xma.ENSXMAG00000012203: LFFFWFLSLLCAIVPFRSKILKASSSGGVPDKLRFTSFYFYFGLILLELVLCCLNEKPPLFSNVVTDPNPCPEATAGFLSTVTFWWFTSLAIKGYKMPLEAKDLWSLNQRDSSKKIVPKL : 240
Gac.ENSGACG00000005901: LLIFWFLSVLCAIVPFRSKILQAFSQNEVTDKLRFTTFYFYFGLVVCELILCCFNEKPPLFSDVVTDPNPCPETTAGFLSTVTFWWFTSMAIKGYKKPLEDKDLWSLNKRDTSKVAVMKL : 234
Oni.ENSONIG00000019586: LFIFWLLCLLCATVPFRSKILQASSQGEVTDKLRFTTFYFYFSMVVCEFILCCFNEKPPLFSNVVTDPNPCPEATAGFLSKITFWWFTSMAVKGYKMPLEAKDLWSLNKRDSSKVMVPRL : 237
Dla.DLAgn_00098120: LFIFWFLSVLCAIVPFRSKILQASSQSEVTDKLRFTTFYFYFSMLVCELILCCFNERPPLFSNVVTDPNPCPETTAGFLSTMTFWWFTSMAIKGYKIPLEAKDLWTLNQRDSSKVAVPKL : 240
Cmi.SINCAMG00000013336: LFIFWLIALLCGIVTARSRLLQALDKGGVSDRFRFATFYVYFALLLVQLILSSFSEPPPLFSKLVTDPNPCPEDSAGFLSKITFWWFTGLARLGYRRPLEDKDLWSLSTDDTSEVIVPRL : 140
Ler.ctg12190: LFFFWLLSLLCAVVPFRSKILAALREHQVSDVFRFTTFYIYFALVLVQFCLCCLREPPPYFTSVSKDVNPSPEFNAGFLSRLTFWWFTRMAIQGYKRPLVEKDLWALNNDDRSDIIVPKL : 140
Sca.ctg14163: ------------------------------DIFRFTTFYIYFVLVLIQFFLCCFSEPPPYFSTASPDENPCPEVSAGFLSRLTFWWFTGMAMQGYKHPLEDKDLWSLNKDDRSDVVVPKL : 90
Rty.XP_020375725_1: ------------------------------------------------------------------------------------------------------------------------ : -
Sac_EU250283: LFFFWLISLLCAVIPFRSKILLALREGGVSDVFRFTTFYIYFVLVLIQFFLCCFSEPPPYFCRTSPDANPCPEASAGFLSKLTFWWFTRMAIQGYKHPLEDKDLWSLNKDDRSDVIVPKL : 240
Tni.ENSTNIG00000004171: LFIFWFLSVLCAIVPFRSKILQASSQGQ--DMLHFTAFTLFFRLVSVATT--------YLFKPRISSAGSCSPAQ---PTAVSAYQAAGRQIENLRQSF----AYRAKRTGRDRKLYFIS : 222
Aca.ENSACAG00000001396: LIIFWFLCALCAVGPLRSKIIRTSAQDQMEDRFRITTFYIYFALILIELILACLKEKPPFFSPVNLDHNPCPELNSGFLSKITFWWFTSMAVQGYKRPLEDKDLWSLNAEDKSDVVVKKL : 240
Gga.ENSGALG00000007522: LIIFWFLSVLCAVAPFRSKIMTTTAQNHVNERFRSATFYIYFVLLIVELILSCFKEKPPFFSPANTDPNPSPELTSGFLSRLTFWWFTSFAILGYKKPLEEKDLWSLNEDDISKNIVQKL : 188
Xtr.ENSXETG00000012239: LIIFWFLATVCAIIPFRSKVMASARQGQVTDKFRFTTFFLYFSLLVIELLLSCFKEARPFFSPVRDEINPCPESDAGFLSRLTFWWFTKMAILGYKRPLEDKDLWSLNEDDTSNVVVTNL : 240
Loc.ENSLOCG00000010918: LFIFWFISLLCAIVPFRSKILQATSQGRITDQLRFTTFYVYFGLLVVQLILSCFNEKPPLFSSVVTDPNPCPEASAGFLSTLTFWWFTSMAVKGYKKPLENKDLWTLNKQDSSEVIVPKF : 240
Lch.ENSLACG7209/6619: LFIFWLISLLCGIVPFRSKIMTTMSQV---KTLLFATFYIYYALLLAELILCCIKESPPLFSSLNTDPNPCPESSAGFLSLMTFWWFTSMAIKGYKHPLENKDLWSLNKQDASDVVVPCL : 234
Mdo.ENSMODG00000020910: LIIFWFLSCFCAIVPFRSKILTALAEGEIKDKFRFTTFYIYFALILFSLILSCLKDRPPFFTPSSVDPNPCPEANAGFLSRLTFWWFTDLAILGYRQPLEDKDLWSLNEENSSRIVVPRL : 240
Dno.ENSDNOG00000046300: LVVFWFLCVVCAIVPFRSKILLVVAQGKISDPFRFTTFYIYFALLLLALTLSCFREKPPVFSTANAKPSPCPEASASFLSRLSFWWFTEMAILGYQRPLQEQDLWSLKEEDCSQAVVQRL : 240
Mmu.ENSMUSG00000020865: LIIFWLLCVICAIIPFRSKILSALAEGKILDPFRFTTFYIYFALVFCALILSCFKEKPPLFSPENLDTNPCPEASAGFFSRLSFWWFTRLAILGYRRPLEDRDLWSLSEEDCSHKVVQRL : 240
Ggo.ENSGGOP00000003354: LIIFWFLCVVCAIVPFRSKILLAKAEGEISDPFRFTTFYIHFALVLSALILACFREKPPLFSAKNVDPNPYPETSAGFLSRLFFWWFTKMAIYGYRHPLEEKDLWSLKEEDRSQMVVQQL : 240
Hsa.ENSG00000108846: LIIFWFLCVVCAIVPFRSKILLAKAEGEISDPFRFTTFYIHFALVLSALILACFREKPPFFSAKNVDPNPYPETSAGFLSRLFFWWFTKMAIYGYRHPLEEKDLWSLKEEDRSQMVVQQL : 240
Ptr.ENSPTRG00000009406: LIIFWFLCVVCAIVPFRSKILLAKAEGEISDPFRFTTFYIHFALVLSALILACFREKPPFFSAKNVDPNPYPETSAGFLSRLFFWWFTKMAIYGYRHPLEEKDLWSLKEEDRSQMVVQQL : 240
Cluf.ENSCAFG00000017201: LIIFWFLCVVCAIVPFRSKILAATAKGEVSDPFHFTTFYIYFALVLFALILSCFREKPPFFSPQNVDPNPCPEVSAGFLSRLSFWWFTKMAILGYRRPLEEQDLWSLKEDDCSQKVVNRL : 240
Bta.ENSBTAG00000020070: LIIFWFLCVVCGIIPFRSKILSALTQGKISDPFRFTTFYIYFALVLSALILSCFREKPPFFSPKNMDPNPCPEAGAGFLSRLSFWWFTKLAILGYRRPLEERDLWSLNKEDRSQMVMQRL : 240

Hsa.CFTR.ENSG0000000162: EREWDRELSKKPKLINALRRCFFWRFMFYGIFLYLGEVTKAVQPLLLRIIASY--DPDNERSIAIYLGIGLCLLFIVRTLLLHPAIFGLHHIGMQMRIAMFSLIYKKTLKLSSRVLDKIS : 171
Hsa.ABCC5.ENSG000001147: ERLWQEELNEV--LRRVVWIFCRTRLILSIVCLMITQLAGFSGPAFMKHLLEYTQATESNLQYSLLLVLGLLLTEIVRSWSLALTWALNYRTGVRLRGAILTMAFKKILKLKNI--KEKS : 259
Hsa.ABCC11.ENSG00000121: HRLWEEEVSRRASVLLVMLRFQRTRLIFDALLGICFCIASVLGPILIPKILEYSEEQLGNVVHGVGLCFALFLSECVKSLSFSSSWIINQRTAIRFRAAVSSFAFEK--LIQFKSVIHIT : 245
Hsa.ABCC12.ENSG00000140: RVLWDEEVARV--LSHVVWKFQRTRVLMDIVANILCIIMAAIGPVILHQILQQTERTSGKVWVGIGLCIALFATEFTKVFFWALAWAINYRTAIRLKVALSTLVFEN--LVSFKTLTHIS : 209
Hsa.ABCC8.ENSG000000060: CEAFDAQV---RAIWQALSHAFGRRLVLSSTFRILADLLGFAGPLCIFGIVDHLGKENDQPKTQYVLAVLLFLALLLQRTFLQASYYVAIETGINLRGAIQTKIYNKHLSTSNLSMGEMT : 353
Hsa.ABCC9.ENSG000000694: KDAYEEQKKKVPSIWLAMYRAFGRPILLSSTFRYLADLLGFAGPLCISGIVQRVNETQNNNTTGYVLAVLLFLALILQRTFLQASYYVTIETGINLRGALLAMIYNKRLSTSNLSMGEMT : 356
Hsa.ABCC10.ENSG00000124: QAHWQEGA-----LWRALYGAFGRCYLALGLLKLVGTMLGFSGPLLLSLLVGFLEEGQEPLSHGLLYALGLAGGAVLGAVLQNQYGYEVYKVTLQARGAVLNILYCKALQLGPS---RPP : 348
Hsa.ABCC4.ENSG000001252: QGFWDKEVLRAPSLTRAIIKCYWKSYLVLGIFTLIEESAKVIQPIFLGKIINYFEDPMDALNTAYAYATVLTFCTLILAILHHLYFYHVQCAGMRLRVAMCHMIYRKALRLSNMAMGKTT : 175
Cel.WBGene00003414: RAEFDKEK-----IVWPFIRIQRATIITLTLARLTADIVHYLNPILLKQLIDYVSLHDQPLSFGIAIACIMFSCSTTRSLLQNYQIAGMCRQAVYYQTVLSNAILHKILRLSPSARSNRT : 318
Cel.WBGene00003409: KTEWLKQTEKAPSVIVTLWQIMKWEILGGSFIKFLSDLLQFANPTFLNYLILFIETPNAPLINGIGLAVGLFLAGQIKSLFMNTYFIAMTRVGAKIQTMLSCAVYEKSLLLSNTARRERT : 355
Hro.HelroG163344: QQKWNEEVASCV-IPNVLSKVYGPRILLSHFFKIFCDLLIMVGPQLQSYLISFIESTQGHVWKGYLYACLFFFNTLLYSILFQQVFHVGMNAGMRMNASIIALVYKKALTMSSEARKTTT : 318
Hro.HelroG157076: -------------------------------------------------------ESGTHVWKGYLLAVLFFVMTILDSLLLQQVFHIGMNAGMRMKGCIIALVYKKALTMSSQSRKRTT : 69
Bfl.230771: HHLWQNELSKLASLFAALFRCFWPLFAGTAMIHAVAIILNLTWPQILKLLIGFTKDRDEPEWKGYLYIALLMAVTMANSLVDQHFVHGCRTLHLRLKTVLKSAVYKKSLTLTSEARKTFT : 347
Lgi.LotgiG105097: LNAWDKKLREEPALFIVLVKTYWFPLFKLNALRVISDLLTFINPLLLKIMITYIENRGNSKWKGYVYVAGFSLVSFTTSAIYNQSFFRSLNLAMNIKSALVAAVYRKSLTMNSDARKMFT : 346
Cel.WBGene00003407: IENLIPEVEGYPSILIPIFKTYKFTLLAGGCYKLMFDLLQFVAPELLRQLISFIEDKNQPMWIGVSIALLMFLSSLLQSMILHQYFHEMFRLGMNIRSVLTSAVYTKTLNLSNEARKGKT : 355
Cel.WBGene00003408: MQNLKPRIDEYPSFVIPIFKTYKYTLLAGFFYKLCFDMLQFLAPQLLKQLIGFIEDKNQPVWIGCSIVGIMFFSSFLQSMFLHQYYHSMFRLGMHVRSVLTSAVYSKALNLSNEARKGKT : 354
Lgi.LotgiG153611: AVEWRKELEKY--LGRVMLKLYWKDMAESYCLKFISDILVFAGPILLSVLINLIDGEAPNGWMGYVLALGLFCLPWIQSVFYHQMYHMAMTLGMRVKAALMAVIYQKALKLNNEAQSKFT : 307
Lgi.LotgiG110718: EIYWEKEVNRVPSLFKVLCKIHCIGLMVSFITKFFADISTFISPLILGEIISYMSTRDEPEWRGYILAVGLMLTSIIRAVLFQISLHQAAVIGMQMKSTLTSVIYQKALTMTNEARKIKT : 169
Dno.ENSDNOG00000024923: EREWTRNRRAAP-LLRAIWQVSRVTFLLGTLSLVIGDAFRFTVPKLLSLFLEFIGDPKAPAWKGYFLAALMFLSACLQTLFEQQHVYRMKVLQIRLRTAITGLVYRKVLALSSSSRKASA : 297
Mmu.ENSMUSG00000030834: EREWRRSCNGLP-LLRAIWRVFRSTFLLGTLSLVISDAFRFAVPKLLSLFLEFMGDRNSSAWTGWLLAVLMFAAACLQTLFEQQHMYRAKVLQMRLRTAITGLVYRKVLVLSSGSRKSSA : 356
Cluf.ENSCAFG00000018197: QREWTRTRSAAP-LLRAIWQVSRSTFLLATFNLVICTVFRFAVPKLFSLFLEFIGNPTIPAWKGYVLAVLLFLSASLQSLLEQHYMYKLKVLQMRLRTAITGLVYRKVLVLSSASRKASA : 356
Bta.ENSBTAG00000015191: EKEWTRNRSATP-LLRAIWQVGRSAFLLGTLSLIVSDVFRFTVPKLLSLFLEFIGDPNTPAWKGYLLAVLMFLSACLQTLFEQQHMYRLKVLQLRLRTAIIGLVYRKVLALSSSSRKSSA : 356
Ggo.ENSGGOG00000009623: LKYKMGPRSLPP-LLKAIWQVFHSTFLLGTLSLVISDVFRFTVPKLLSLFLEFIGDPKPPAWKGYLLAVLMFLSACLQTLFEQQNMYRLKVLQMRLRSAITGLVYRKVLALSSGSRKASA : 348
Hsa.ENSG00000091262: EKEWMRNRSAAP-LLKAIWQVFHSTFLLGTLSLIISDVFRFTVPKLLSLFLEFIGDPKPPAWKGYLLAVLMFLSACLQTLFEQQNMYRLKVLQMRLRSAITGLVYRKVLALSSGSRKASA : 356
Ptr.ENSPTRG00000007815: EKEWMRNRSAAP-LLKAIWQVFHSTFLLGTLSLIISDVFRFTVPKLLSLFLEFIGDPKPPAWKGYLLAVLMFLSACLQTLFEQQNMYRLKVLQMRLRSAITGLVYRKVLALSSGSRKASA : 213
Oan.ENSOANG00000005123: EREWKKNHHQTP-LLKAIWRVFGLSFLLGSLSLVACDIFTFSIPKILSLFLEFISDLAAPGWKGYFCAVLLFLINSLKILFEQRYMYVCFVLGMRLKTALVGLVYRKVLALSSAARKATA : 309
Mdo.ENSMODG00000005815: ESEWKRICNETPRLLKAIWKVFNGTFLLGTLSLIVCDVFRFAVPKILSFFLEFISDPEAPAWKGYFYAVLLFLSACLQTLFEQRHMYVCMVLEIRLRTAVMGLVYRKVLALSNAMRKTAA : 360
Gga.ENSGALG00000006698: EREWKKYNNRTP-LLQAFWSMFGIYFLLSTLCLVICDVFLFSIPKILSLFLEFIEDQEAPSWHGYFYAFILVLLACLQTLFEQRYMYMCLVLGLRLKTAVTGLVYRKILTVSNASRKAVT : 359
Aca.ENSACAG00000003478: KDAWEKHCASA--LLKSFWSVFGTYFILGTLCLVAGDVFLFLIPKTLSVFLDFISAPEAPSWKGYFYAAAMFLLACLQTLFEQQYMYMCLVLGVRLKTAITGLVYRKLLVMSNAAKKEAT : 298
Cel.WBGene00003413: ESFWEPKRLKY--VVSSLFMMFRWEFLLASTLKFVSDTMQFASPFLLHELLNFISAKNAPFWKGMALSILMFSVSELRSLILNGYFYIMFRMGTKIQTSLTAAVYKKTLLISNSARRDRT : 355
Aga.AGAP008437: DKYWYESVEKG--VLPAMVKAYGGPFWFAGMLQFAISGLQFASPYLMQEIMAVIA-LDGPFWKGMIITLGLFLTSLLIALFNGQYFHRTFLVGFRIRTGLISAIYRKALRISSFAKKDTT : 354
Aga.AGAP027980: DKYWDMSVANGGSSLYAMVRAYGAPFWFAGMLQLAISGLQFASPYLMQEMMAVIA-LDGPVWKGLLLTFALFAASLLLALLNGQYYYNTFLSGFRIRTGLVSAIYRKALRISSAAKKDTT : 359
Aga.AGAP028128: DKYWKISVEKGGSVLYTMIRAYGGPFWFAGMLQLAISGLQFASPYLMQELMAVIA-FDGPLWKGFLLTFGLFGASLLLGLFNGQYLFYTFLSGFRIRTGLISAIYRKALRISSAAKKDTT : 359
Spu.026395: ESSWLKEVHRVASLFRAVAMRYGEKFLVAVLLKFIHDCLLFVNPQILRLLINFTEDHTIWQWRGFFYAGVMLMISMFNSTLLHQYFHRCFIVGMHLRSAIIGVVYRKSLQLSSAARKGAT : 359
Hsa.ABCC2.ENSG000000238: ETHMKRELQKAPKLMKALFKTFYMVLLKSFLLKLVNDIFTFVSPQLLKLLISFASDRDTYLWIGYLCAILLFTAALIQSFCLQCYFQLCFKLGVKVRTAIMASVYKKALTLSNLARKEYT : 349
Xtr.ENSXETG00000026360: SKGVEKECKKAKTLLKVIMRSFGLYFLLSALLMTFYTAFLFISPLLVRLLLQLLKDPSAPSWQGFLVAVFLFICPCCQSLFLHQHDYICYVIGMRLRAAIVGTVYKKALMISSAGRKESS : 344
Cel.WBGene00003410: YNLWDKQSKKFPSIIWTLFLMFKWDVITAMFVKLLSDVLLFCNPLLLKSLIRFTEELERPMWQGVVLAFTMFFSAELSSILLSHYFYLMYRVGTRVQTCLTAAVYRKTLRLSNAARREKT : 356
Ame.GB53134: EKYWKKNSQKRSSVLPPLCKAFGATFLFGAVLKFVQDIITFVSPQILQLLIDFIKGHE-PLWKGYFYAVLLLITAIFQTLVLSQYFHRMFLVGLRIRTALIAAIYRKALRMSNAARKEST : 354
Pma.KM232931_1: ---------------------------------------------VLSLLIRLVESPETPSWQGYSLAVLLLVSACVQSLVLQQHMHVCTVTGIRTRSALTALVYNKSLVMSNAAKKSST : 76
Gac.ENSGACG00000003037: ENLWAQNCKPLYGLLHTVACSFGPYFLCGTLWLLLHEVFMFAVPQVLSLLLAFISDEDAAMWKGFLFCSLLFLLSCLQSLLHHQYMFHCFSVGMRLKTALIGLVYRKCLLLSSAARRRGD : 360
Loc.ENSLOCG00000007152: EREWTAQCTKLFSLLRTLARSFGPYFLTGTLCLIVHDAFMFSIPQVLSFLLGFMRDKDAPLWKGYFYAALMFLLSCLQSLFNHQYMYSCFTVGMRVKTAVMGLVYRKSLVINSAARRTCT : 360
Gmo.ENSGMOG00000005748: LRKLQREQ-----LLRTLTRKFGPYFLTGTLCIVFHDAFMFAIPQVLSLLLGFMRDDEAPLWKGYFYATLIFLLSCLQSLFNHQYMYTCFTVGMRVKTAVMGLVYRKSLVINSSARRTCT : 338
Gac.ENSGACG00000019172: EEDWTDECAKVSGLLRTMARKFGPYFLTGTLYIIFHDAFMFAIPQVLSLLLDFMRDEDAPLWKGYFYATLMFLLSCLQSLFNHQYMYTCFTVGMRVKTAVMGLVYRKSLVINSSARRTCT : 360
Tni.ENSTNIG00000012067: QQDWTAECAKISGLLRTLTRKFGPYFLSGTLCIIFHDAFMFAIPQVLSLLLGFMRDEDAPLWKGYFYATLMFLLSCLQSLFNHQYMYTCFTVGMRVKTAVMGLVYRKSLVINSASRRTCT : 359
Ola.ENSORLG00000013429: QQEWGAECAKLSGLLRTLARKFGPYFLTGTLCIIFHDAFMFAIPQVLSLLLGFIRDPEAPQWKGYFYATLMFLLSCLQSLFNHQYMYTCFTVGMRVKTAVMGLVYRKSLVINSAARRTCT : 359
Oni.ENSONIG00000018866: EQDWTAECAKLHQLYLTLARKFGPYFLTGTLCIIFHDAFMFAIPQVLSLLLDFMRDEDAPLWKGYFYATLMFLLSCLQSLFNHQYMYTCFTVGMRVKTAVMGLVYRKSLVINSSARRTCT : 359
Xma.ENSXMAG00000004906: QQDWTDECAKLSGLLRTLARKFGPYFLTGTLCIIFHDAFMFAIPQVLSLLLGFMRDEDAPLWKGYFYATLMFLLSCLQSLFNHQYMYTCFTVGMRVKTAVMGLVYRKSLVINSSARRTCT : 359
Dla.DLAgn_00177430: QQDWTAECAKISGLLRTLARKFGPYFLTGTLCIIFHDAFMFAIPQVLSLLLGFMRDEDAPLWKGYFYATLMFLLSCLQSLFNHQYMYTCFTVGMRVKTAVMGLVYRKSLVINSAARRTCT : 359
Dre.ENSDARG00000016750: EEEWTAKRTKLFCLLRTLARSFGPYFLTGTLCLIVHDVFMFSVPQVLSLLLGFMKDEDTPLWKGYFYATLMFLLSCLQSVFNHQYTYTCFTVGMRVKTAVMGLVYRKSLVMNSSARRTCT : 358
Ame.ENSAMXG00000004837: EEEWMAERTKLFCLLRTLARNFGPCFLSGTLCLIVHDAFMFAIPQVLSLLLGFMREEDAPLWKGYFYASLMFLLSCLQSLFNHQYMYSCFTVGMRVKTAVMGLVYRKSLVINSAARRTCT : 357
Ame.ENSAMXG00000003085: EREWTTQCAKLSGLLRTLAKTFGPFFLTGTLCLIIHDAFMFSIPQVLSLLLSFVGDKDVPLWKGYLFAFTMFLLSCLQSLFNHQYMYSCFTVGMRVKTAVMGLVYRKALVISSAARRTCT : 360
Dre.ENSDARG00000094901: EKEWAKQWAKLTGLFRTLAKIFSPYFLTGTLFLVIQDALMFSIPQVLSLLLGYVRDEDAPLWKGYLFAFSLFLLSCLQSLFNHQYMYTCLTVGMRVKTAVMGLVYRKSLVINSAARKTCT : 333
Dre.ENSDARG00000095820: EKEWAKQWAKLAGLLRTLAKNFGPYFLTGTLCLVIQDAFMFSIPQVLSLLLGYVRDEDAPLWKGYLFAFSLFLLSCLQSLFNHQYMYTCFAVGMRVKTAVMGLVYRKSLVINSAARKTCT : 360
Lch.ENSLACG00000022117: EREWKKEYAKGTALLKALWRIYGNHFLLGTLCLVLSDVLLFSIPQILDSLLGFMSDPEAPVWRGYFYAALMFLFACLQSLLVHQYMYMACIIGMRLKTALTGLIYRKILVMTSGAKKTST : 360
Dpu.347281: EKHWLKSLSKQVSILPALCKTFAPEFLLGALLKLIQDLLAFVSPQILSLLIGFVEDSTQESWKGYLYAAILTITAMTQTLILGQYFQRMFIIGMQIRTSIVSSIYRKAIKISNSARKEST : 360
Lca.KE993868_1: -----------------------------------------------SHLIRFVGDSSRPTWHGYALAGLMFLCAIATSMLAHQHFHAGFVTGIRLRSALTGLLYRK------------- : 105
Pma.KM232930_1: QAVWRKEQARIPSLIRAMALAFGPRYLVGSVYKLLQDILSFASPLLLSHLIRFVGDNSRPTWHGYALAGLMFLCAIATSMLAHQHFHAGFVTGIRLRSALTGLLYRKALMITQAARLSCA : 360
Csa.ENSCSAVG00000008135: LKNWNAEKAKVASLGLAMVKTFGPYFLFSTVLKVFHDLLSFVSPQLLKGLITFTATENAPMWQGYLLGVGMFVSAILQSVILQQYFHICFVVGMRLRSAIISSIYRKSLLLSNAARKEST : 351
Csa.ENSCSAVG00000003792: NKTWSAEKAKYPSLFKAMVRVFGPYYFLGSLLKVVSDLLTFVSPQLLSALILFTS-NNAPVWQGYMLAVLLFVTAILQSIVLQQYFHVCFTVGMRMRSAVVGAVYRKAMVLSNSARKEST : 292
Cin.ENSCING00000020698: ------------------------------------------------------------------------------------------------------------------------ : -
Tca.TC012253: EKHWKRILQKC--ILPALVKAFGPTFIFGALLKLIQDLLTFVSPHILNLLIQFVKNNE-EQWKGFLYAGALFVTATIQTIVLSQYFNRMFVVGMRIRTALVSAIYKKALRVSNKARKEST : 355
Aga.AGAP009835: LHHWNETLEKTASILPALIKTFGGTFLLGSFLKLGQDLLTFASPQILKLIIGFVGGEE-PMWKGLMYAITLFVVAGTQTLLLGQYFNRMFFVGLRIRTALISAIYRKALIISNSARKGST : 354
Lgi.LotgiG107213: DKYWQYESQCVPRLLICLIKTFGGFCLKAAIFKFIYDVLQFVSPQLLKLLINFTKDKDSYLWVGLFYAGCMFTVALIQSFILHQYFHGCFLLGMRIRTAIVSAVYRKTLVLSNTSRKNST : 358
Bfl.90918: EKELLKEIVK---YKRAMVRYFLPTFATSAVFKLCNDILMFVSPQILGWLIEFTKDKSIHSWKGYSIAVLLLVTTTLQSLFNHQFFYRVNVWGMRVKTAITAAIYKKALLLTNEARTSLT : 241
Dme.FBgn0032456: AHHWNQNVRKNASIMPPIYKSFGGVFLFGALMKLFTDTLTFAQPQVLSLIISFVEAQDAPEWKGILYAVLLFVLAAAQTFILGQYFHRMFIVGLRIRTALINAIYRKALRISNSTKKEST : 359
Oan.ENSOANG00000013379: LREWDRQKVECPSFLRALLATFGPYFLISSFYKLLQDLLSFVNPQLLSVLIDFI------------------------------------------------------------------ : 294
Pma.AKC42143_1: MREWEREC-----------------------------------------------SRPPSAANG-------------------------------------------------------- : 257
Lca.KE994284_1: VREWERECRQHPSLLRALCRAFGPAFLLGSVYKLAQDMLTFVSPQILRLLIGFVQHREAPMWQGYFLSLLLFACAILQMLILHQYFHICFLTGMHLRTAITGAVYRK------------- : 159
Cmi.SINCAMG00000015787: VYEWKKECSKMPSLLKALCRAFGPYLMISFFFKIFHDVLVFASPEIMRLLLGFVNNHFAPVWQGYFYAILLFICAFVQTLFLHQYFHICFVTGMRLKTAIIGAVYRKALVITNEARKTST : 359
Ler.ctg13956: ------------------------------------------------------------------------------------------------------------------------ : -
Rty.XP_020377569_1: ------------------------------------------------------------------------------------------------------------------------ : -
Sca.ctg67278: VEEWEKECAKNPSLLKALCRVFGPYFMMSFLYKIIHDLLMFASPEILKLLLQFINNENAPAWQGYFYVMLLFLCALIQTLFLHQYFQICFVTGMRLKTAIIGAVYRKALVISNAARKTST : 260
Lch.ENSLACG00000001471: DRQWQKETAKTPSLFKALCRTFGPYFLMSFVYKAIHDLLMFVGPEILRLLIQFVNNKDAPNWHGYIYTALLFVSALMQTVVLHQYFHICFVTGMRLKTSVIGTVYRKALVITHAARKMST : 360
Tni.ENSTNIG00000005013: LLHSNRKEDRMPSLFWALCLTFGPYFLISCLYKLIQDVLMFIGPEILRLLINFVNNPEAPSWQGYFYTSLLFVCTTVQSLVLQKYFHVCFVSGMRLRTAIIGAVYRKALVIGSTARRTST : 360
Loc.ENSLOCG00000007196: VHRWNSQRAKVPSLAMALCLAFGPHFLVSFVYKIIHDVLMFVGPEILKLLILFVNDPSAPSWQGYSYTALLFVCACLQTLILQQYFHVCFVTGMRLRTAIVGAVYRKALVITNAARCTSM : 360
Dre.ENSDARG00000104719: VRRWDQECVKVPSLFFALCRTFGPYFLVSSLYKIIHDVLMFVGPEILRLLILFVNDSSAPTWHGYFYTALLFVCTCLQTLILQKYFHVCFVTGMRLRTAIVGAVYRKALVITNAARRTST : 360
Ame.ENSAMXG00000002943: VRRWDHECSKGPSLFLALCRTFGPYFLVSSIYKIIHDVLMFVGPEILRLLIQFVNDSDAPSWHGYFYTALLFVCTCFQTLILQKYFHVCFVTGMRLRTAIVGAVYRKALVITNAARRTST : 360
Xma.ENSXMAG7738/17319: VHRWNSECQKVPSLLWALCLTFGPYFLISCLYKIIQDILMFVGPEILRLLIHFVNDTSALSWQGYFYTALLFICTCVQSLILQRYFHVCFVSGMRLRTAIIGAVYRKALVISNGARRTST : 333
Ola.ENSORLG00000017141: LRRWTAECHKVPSLLWALCLTFAPHFLVSCLYKLIQDILMFVGPEILRLLILFVNDPDAPSWQGYFYAALLFVCTCVQSLILQRYFHVCFVSGMRLRTAVIGAIYRKALVISSAARRSST : 360
Gac.ENSGACG00000000434: VGRWNVECQKVPSLFWALCLTFGPYFLISCLYKIIQDILMFVGPEILRLLIRFVNNSSAPSWHGYFYTALLFICTCVQSLILQKYFHVCFVSGMRLRTAIIGAVYRKALVISSEARRTST : 360
Oni.ENSONIG00000007824: VKRWNTQCQKFPSLLWALCLTFGPYFFISCIYKLIQDILMFVGPEILRLLIQFVNDSSAPSWQGYFYAALLFICTSVQSLILQKYFHVCFVSGMRLRTAIIGAVYRKALVISSAARRTST : 360
Dla.DLA_00195360: VCRWNAECQKVPSLLWALCLTFGPYFLISCLYKIIQDILMFVGPEILRLLIRFVNNSSAPSWQGYFYTALLFICTCVQSLILQKYFHVCFVSGMRLRTAIIGAVYRKALVISSAARRTST : 314
Xtr.ENSXETG00000019661: SKNWEKEYNKAPSLLKALYKTFGPYFFISCFFKFFHDVLMFSGPQLLQLLIKFVGDKDAPDWHGYLYTFLLFFCACLQTLILHKYFHICFVTGMRLKTAVVGLVYRKALVINNSARRTST : 360
Aca.ENSACAG00000005349: VKDCKWDCVKKPSLSKVLYKTFGPYFLMSFLFKAFHDLMMFAGPEILKRLIIFVSDQSAPNWQGYFYTALLFLSAGLQTLVLHQYFHICFVTGMRLKTAVIGAIYRKALVITHSARKSST : 360
Gga.ENSGALG00000006646: AKNWAKEWAKTASLSKVLYKTFGPYFLMSFLFKAAHDLLMFTGPEILKLLINFVNNKSAPNWQGYFYTGLLFVCACLQTLILHQYFHICFVTGMRLKTAIVGVIYRKALVITNSARKTST : 360
Dno.ENSDNOG00000014990: VKNWKKECARSPSLFKVLYKTFGPYFLMSFLFKALHDLMMFAGPELLKLLISFVNDKQAPSWQGYFYTALLFISACLQTLVLHQYFHICFVSGMRFRTCSIGVTWEPALVISNAARKTST : 360
Mmu.ENSMUSG00000023088: VNNWKKECDKSPSLFKVLYKTFGPYFLMSFLYKALHDLMMFAGPKILELIINFVNDREAPDWQGYFYTALLFVSACLQTLALHQYFHICFVSGMRIKTAVVGAVYRKALLITNAARKSST : 360
Ggo.ENSGGOG00000004324: VKNWKKECAKTPSLFKVLYKTFGPYFLMSFFFKAVHDLMMFSGPQILKLLIKFVNDTKAPDWQGYFYSILLFVTACLQTLVLHQYFHICFVSGMRIKTAVIGVVYRKALVITSSARKSST : 333
Hsa_ENSG00000103222: VKNWKKECAKTPSLFKVLYKTFGPYFLMSFFFKAIHDLMMFSGPQILKLLIKFVNDTKAPDWQGYFYTVLLFVTACLQTLVLHQYFHICFVSGMRIKTAVIGAVYRKALVITNSARKSST : 360
Ptr.ENSPTRG00000007812: VKNWKKECAKT-------------------------------------LLIKFVNDTKAPDWQGYFYTVLLFVTACLQTLVLHQYFHICFVSGMRIKTAVIGAVYRKALVITNSARKSST : 314
Cluf.ENSCAFG00000018208: VKNWKKECAKSPSLFKVLYKTFGPYFLMSFLFKALHDLMMFAGPEILKLLINFVNDKKAPDWQGYLYTALLFICACLQTLVLHQYFHICFVSGMRIKTAVIGAVYRKALVITNSARKSST : 360
Bta.ENSBTAG00000021090: VKNWKKECAKSPSLFKVLYKTFGPYFLMSFLFKAVHDLMMFAGPEILKLLINFVNDKKAPEWQGYFYTALLFISACLQTLVLHQYFHICFVSGMRIKTAVIGAVYRKALVITNAARKSST : 360
Mdo.ENSMODG00000004194: VKNWKKECAKTPSLFKVLYKTFGPYFLMSFLFKALHDLMMFAGPEILKLLINFVNDNQAPDWQGYFYTALLFVSACLQTLVLHQYFHICFVSGMRIKTAVIGAIYRKALVITNSARKSST : 360
Oan.ENSOANG00000005124: VKNWAKECTKSPSLFKVLYKTFGPYFLMSFLFKALHDLMMFAGPEILKLLINFVNDKDAPDWQGYLYTGLLFVSACLQTLVLHQYFHICFVSGMRIKTAVIGAIYRKALVITNSARKSST : 149
Bfl.232174: EKEWQKERQKCPSLVKALVRCFWHRFAVIALYKLIQDILLFVSPQLLRLLISFTKVKEVFSWKGYLYAVLLWVVAILQSLVLGQYFHGSFLMGMRLRTVIISAIYKKSVVLTNEARKSST : 359
Bfl.118638: EKEWQKERQKCPSLVKALVRCFWHRFAVIALYKLIQDILLFVSPQLLRLLISFTKVKEVFSWKGYLYAVLLWVVAILQSLVLGQYFHGSFLMGMRLRTVIISAVYKKSVVLTNEARKSST : 360
Bfl.118636: EKEWQKERQKCPSLFKALVRCFWPLFAVSAIYKLIQDILLFVSPQLLKLLIAFTKDKDIYSWKGYLYAVLLLLVAIIQSLVLHQYFHGCFVMGMRLRTVIISAVYKKSLVVTNEARKSST : 360
Bfl.128060: EKEWQKERQKCPSLFKALVRCFWPLFAVSAIYKLIQDILLFVSPQLLKLLIAFTKDKDIYSWKGYLYAVLLLLVAIIQSLVLHQYFHGCFVMGMRLRTVIISAVYKKSLVVTNEARKSST : 360
Dre.ENSDARG00000096662: LNQWEVEKSKAPSFLWALLRAFGPYFLIGSAFKLLQDLITFINPQLLRMLIAFTKQSDVPSWWGYALAFLMFGTSLLQTLILHQHFQYCFVTGMRLRTGIIGAIYRKALVITNEAKRSST : 358
Ame.ENSAMXG00000016253: LREWEGEKSKAPSFLRSLLKAFGPYFLIGSAFKLLQDLITFINPQLLRMLIEFTKETGVPSWWGFALAFMMFGCALLQTLILHQHFQYCFVTGMRLRTAIIGAIYRKSLVITNEAKRSST : 359
Ola.ENSORLG00000020741: LAEWRKEEAKAPSFLRAILKAFGPYFLIGSAYKLLQDVITFINPQLLSLLISFTKQEDVPLWWGYTLAFLMFFTAFLQTLILHRHFQYCFVTGMNVRTALIGAIYRKALVITNAAKRSST : 360
Gmo.ENSGMOG00000010029: LEEWEKEQAKA------------------------------------QLLISFTKQKGAPDWWGYALAFLMFFTALLQTLILHQHFQYCFVTGMRLRTAIIGAIYRKSLVITNAAKRKST : 314
Xma.ENSXMAG00000012203: LKEWEKEQTKVPSFLRVLIKAFGPYFLIGSCFKLLQDSIMFVNPQLLRMLISFTSQKDVPIWWGYTLAFLMFFAAILQTLILHRHFQYCFVTGMNVRTALIGAIYRKSLVITNAAKHSST : 360
Gac.ENSGACG00000005901: LQEWEKELAKAPSFLRALIKAFGPYFLIGSAFKLLQDVITFVNPQLLRMLISFTKEKEAPDWWGYSLAFLMFFTAILQTLILHCQFQYCFITGMNIRTAVIGAIYRKSLVITNAAKRSST : 354
Oni.ENSONIG00000019586: LNEWEKEEAKAPSFLRALIKAFGPYFLIGSGYKLLQDIITFVNPQLLKMLISFIKAKDVPNWWGYTLACLMFFTALLQTLILHHHFQYCFVTGMNVRSAVIGAIYRKALVITNAAKRSST : 357
Dla.DLAgn_00098120: LKEWEKEQAKAPSFLRALIRAFGPYFLIGSAFKVLQDVVTFVNPQLLRMLILFTKQKGVPDWWGYSLAFLMFFTAFLQTLILHHHFQYCFVTGMNVRTAVIGAIYRKALVITNAAKRSST : 360
Cmi.SINCAMG00000013336: LKEWEQEIANAPSLLRALCKTFGPYFLIGSAFKLCQDLLTFVNPQLLSKLLQFVKQKASPVWWGYMIAVLMFFSAVLQTLILHQHFQYCFITGMRLRSAIIGAIYRKSLVITNAAKRAST : 260
Ler.ctg12190: LKEWEKEKMKAPSFLVALCKTFAPYFLMGSALKLCQDLLSFVNPQLLNMLISFINDSTSRIWWGYAISILMFLSAALQTLLLHQHFHYCFVTGMRLRTAIIGAIYRKSLIITNSAKRSST : 260
Sca.ctg14163: LEEWEKEKLKAPSFLIALCKTFGPYFLLGSALKLCQDLLSFVNPQLLNMLISFINNPEAKTWWGYSISILMFLSAAVQTIILHQHFQYCFVTGMRLRTAIVGAIYRKSLVITNSAKRSST : 210
Rty.XP_020375725_1: ------------------------------------------------------------------------------------------------------------------------ : -
Sac_EU250283: LKEWEKQKLKAPSFFLALFKAFTPYFLMGSALKLCQDLLSFVNPQLLNMLISFIKNPEARTWWGYGISFLMFFSAALQTLILHQHFQYCFVTGMRLRTAIVGAIYRKSLVITNSAKRSST : 360
Tni.ENSTNIG00000004171: IVNFFPFYSKQ--LINLSAKGLGAFVILQNLIFFFFKNTTFLSNIILCTCSLFICNAVSSSLANACLLCFLFQSEVTDKLRFTTFYFY---FGLVLCELILCCFNEKPPLFSNVDTDPVS : 337
Aca.ENSACAG00000001396: QKEWNKQK---PSFLKALIRTFGPYFLIGSFFKLIQDLLAFVNPQLLRILIGFIKDQSAPLWWGYLIAALMFFSAVLQTIILHQHFQYCFVTGMRLRTGIIGMIYRKSLVITNSAKRSST : 357
Gga.ENSGALG00000007522: SREWDKEKAECPSFLKALMRTFGPYFLIGSFFKLIQDLLSFVNPQLLSVLISFVKDKDAPTWWGFLIAALMFACAVLQTLILHQHFQYCFVTGMRLRTGIIGVIYRKSLVITNSAKRSST : 308
Xtr.ENSXETG00000012239: IKEWEKEKSNLPSFLKVLLRTFGPYFLIGSFFKLFQDLLSFVNPQLLSILITFIKNKDAPSWWGFCIAVLMFLTSLVQTLILHQHFQYCFVTGMRLRSAITGIIYRKSLVITNSAKRSST : 360
Loc.ENSLOCG00000010918: LKEWEVEKTKVPSFLKALLKAFGPYFLIGSAFKLLQDLINFVNPQLLNLLIYFTNQKTVPSWWGFALAFLMFFCAMLQTIILHQHFQYCFVTGMRLRTAIIGAIYRKALVITNAAKRSST : 360
Lch.ENSLACG7209/6619: LKEWAKEKGKVPSFLKALLRAFGPYFLIGSVYKLAQDLLTFVNPQLLNMLIAFINSPDAPSWWGFLIAILMFLCAALQTIILHQHFQYCFVTGMRLKTAIIGSIYRKSLVITNSAKRSST : 354
Mdo.ENSMODG00000020910: LKEWEKQRIQAPSFLRALLITFGPYFLISSFYKLIQDLLSFVNPQLLSVLIRFISNPKAPAWWGFFIAGLMFGCSVLQILILHQNFQYVFVTGMRVRTGIIGVIYRKALVISNSAKRSST : 360
Dno.ENSDNOG00000046300: LEAWKKQQRQAPSFLWALLATFGPSFLVSSGFRLMQDLLSFINPQLLSILIRFISTPSAPAWWGFLVARLMSTCSMMQTLTLHQHFHCAFGTGLRLHMGIISVPYRKALVITNSVKRVST : 360
Mmu.ENSMUSG00000020865: LEAWQKQQNQAPSFLRALVRTFTSSLLMSACFNLIQNLLGFVNPQLLSILIRFISDPTAPTWWGFLLAGLMFLSSTMQTLILHQYYHCIFVMALRLRTAIIGVIYRKALVITNSVKREST : 360
Ggo.ENSGGOP00000003354: LEAWRKQEKQTPSFLKALLATFGSSFLISACFKLIQDLLSFINPQLLSILIRFISNPMAPSWWGFLVAGLMFLCSMMQSLILQHYYHYIFVIGLKFRTGIMGVIYRKALVITNSVKRAST : 360
Hsa.ENSG00000108846: LEAWRKQEKQTPSFLKALLATFGSSFLISACFKLIQDLLSFINPQLLSILIRFISNPMAPSWWGFLVAGLMFLCSMMQSLILQHYYHYIFVTGVKFRTGIMGVIYRKALVITNSVKRAST : 360
Ptr.ENSPTRG00000009406: LEAWRKQEKQTPSFLKALLATFGSSFLLSACFKLIQDLLSFINPQLLSILIRFISNPMAPSWWGFLVAGLMFLCSMMQSLILQHYYHYIFVTGLKFRTGIMGVIYRKALVITNSVKRAST : 360
Cluf.ENSCAFG00000017201: LEAWKKLQKQAPSFLWALLATFGPSILISMCFKVVQDLLSFINPQLLSILIRFISNPTAPTWWGFLVAGLMFLCSMAQTLVLHQYFHCIFEMALRLRTAITGVIYRKALVITNSAKREST : 360
Bta.ENSBTAG00000020070: LEEWKKQQDQAASFLRALMATFSSSFLLSMGFKLIQDLLSFINPQLLSILIRFISNPTAPTWWGFLVAGLMFVCSVMQTLILHQYFHCIFVMGLRFRTGIIGVIYRKALVITNSVKREST : 360

Hsa.CFTR.ENSG0000000162: IGQLVSLLSNNLNKFDEGLALAHFVWIAPLQVALLMGLIWELLQASAFCGLGFLIVLALFQAGLGRMMMKYRDQRAGKISERLVITSEMIENIQSVKAYCWEEAMEKMIENLRQTELKLT : 291
Hsa.ABCC5.ENSG000001147: LGELINICSNDGQRMFEAAAVGSLLAGGPVVAILGMIYNVIILGPTGFLGSAVFILFYPAMMFASRLTAYFRRKCVAATDERVQKMNEVLTYIKFIKMYAWVKAFSQSVQKIREEERRIL : 379
Hsa.ABCC11.ENSG00000121: SGEAISFFTGDVNYLFEGVCYGPLVLITCASLVICSISSYFIIGYTAFIAILCYLLVFPLAVFMTRMAVKAQHHTSEVSDQRIRVTSEVLTCIKLIKMYTWEKPFAKIIEDLRRKERKLL : 365
Hsa.ABCC12.ENSG00000140: VGEVLNILSSDSYSLFEAALFCPLPATIPILMVFCAAYAFFILGPTALIGISVYVIFIPVQMFMAKLNSAFRRSAILVTDKRVQTMNEFLTCIRLIKMYAWEKSFTNTIQDIRRRERKLL : 329
Hsa.ABCC8.ENSG000000060: AGQICNLVAIDTNQLMWFFFLCPNLWAMPVQIIVGVILLYYILGVSALIGAAVIILLAPVQYFVATKLSQAQRSTLEYSNERLKQTNEMLRGIKLLKLYAWENIFRTRVETTRRKEMTSL : 473
Hsa.ABCC9.ENSG000000694: LGQINNLVAIETNQLMWFLFLCPNLWAMPVQIIMGVILLYNLLGSSALVGAAVIVLLAPIQYFIATKLAEAQKSTLDYSTERLKKTNEILKGIKLLKLYAWEHIFCKSVEETRMKELSSL : 476
Hsa.ABCC10.ENSG00000124: TGEALNLLGTDSERLLNFAGSFHEAWGLPLQLAITLYLLYQQVGVAFVGGLILALLLVPVNKVIATRIMASNQEMLQHKDARVKLVTELLSGIRVIKFCGWEQALGARVEACRARELGRL : 468
Hsa.ABCC4.ENSG000001252: TGQIVNLLSNDVNKFDQVTVFLHFLWAGPLQAIAVTALLWMEIGISCLAGMAVLIILLPLQSCFGKLFSSLRSKTATFTDARIRTMNEVITGIRIIKMYAWEKSFSNLITNLRKKEISKI : 295
Cel.WBGene00003414: AGEILNHAAVDIEIIVHSVPYLQNMWSVPFQVTLAMTMLAITLGWAAMAGVCIMILFIPLNLCTSRFIKLSQQKQMKIKDERTKLSNEMLNGIKVVKLYAWEESFEDQINRLRAKEVKML : 438
Cel.WBGene00003409: VGEMVNILSIDVDRFRMITPQIQQYWSSPFQIIICMVLLSQTIGVAVWAGIVVMISIVPINICVSMITKRWQLRLMKYKDERIRLINEVLNGIKVVKLSAWETAMEETIERVRDKELKMI : 475
Hro.HelroG163344: VGEIVNLMSVDSQRLQDATGYLWMVWSSPFQIVLSMYFLYGILGPSIFAGVAVMILFIPINAWVSAVQRKMQTDQMKRKDDRIKLTNEILNGIKVIKLYAWELSFKEKVSAIRNLELVTL : 438
Hro.HelroG157076: VGEITNLISVDSQRLQDSIEYSYALWSSPLQICLCLYLLYGILGVSVFVGIGILVLFVPINWWVTSKQGSVEVTQMGIKDERIKLMNEILNGIKVIKLYAWELSFQEKVNVIRNLELKTL : 189
Bfl.230771: VGSMTNLMSVDAQRVADTCFDVQFVWSMPVTIVGTMYVLWNSLGVSALAGVTAAVLLVPLHTWMVSKMKGLQKTIMELKDTRTKLMSEMLNGMKVLKLYGWELSFKAKIDVIREKELTAI : 467
Lgi.LotgiG105097: VGAIVNLVSGDCKKLSEIVTFLWMIWSCPLQICIALYLLYQTLGVSFVAGLGVILMVIPLNSVLSTATKKIHSELLTIKDSRLKITNEVLNGIKVLKLFAWEESFEGKIMKIRQKEIRLL : 466
Cel.WBGene00003407: TGAIVNLMSVDIQRIQDMTTFIMLFWSAPLQILLSLYFLWKLLGVSVLAGFVILILLIPFNSFISVKMRNCQMEQMKFKDERIKMMSEILNGMKVLKLYSWEKSMEKMVLEVREKEIRVL : 475
Cel.WBGene00003408: IGAIVNLMSVDIQKIQDMAPTIMLFWSAPLQIFLSIYFLWKFLGVAALAGLVVLILALPVNGLIAIQMRKCQTEQMKLKDERIKMMSEILNGMKVLKLYSWERSMENMVLKIRERELHIL : 474
Lgi.LotgiG153611: VGEIVNLMAVDCQRIQDAACYTFYVWSIPFQVAIAVYLLWSTMGPSSLAGLVVLILLVPVNAIVATKQFKYQKENLQYKDKRIKLTSEVLEGIKVLKLYAWECVFKQKILEMRKQELSIL : 427
Lgi.LotgiG110718: SGEIVNLMSVDCQRIQDLLNFLALLWSTPLQIVLSLVLLYYTIGPAVFAGVVVLCLMIPINSVFAGKQQKYQQENLKIKDKRIKWMTEILNGIKVLKLYGWELSFQEKVEKIRALEIREL : 289
Dno.ENSDNOG00000024923: VGDVVNLVSVDVQRLMDSIFFLNGLWLPLVWIIICFVFLWQLLGPSALTAVAVFLSLLPLNFFITKKRKHHQEEQMRQKDARARLTSSILRHAKLIKFHGWEEAFLDRVLQSRRRELGAL : 417
Mmu.ENSMUSG00000030834: AGDVVNLVSVDIQRLAESIIYLNGLWLLFLWIFVCFVYLWQLLGPSALTAVAVFLSLLPLNFFITKKRGFHQEEQMRQKASRARLTSSMLRTVRTIKSHGWEHAFLERLLHIRGQELSAL : 476
Cluf.ENSCAFG00000018197: VGDVVNLVSVDVQRLTECIIYLNGLWLPVIWMIICFVYLWQLLGPSALTAIAVFMSLLPLNFFITKKRKQHQEEHMRQKDSRVRLTSCIIRNMKMVKSHGWEEAFLERVLHIRGQELGAM : 476
Bta.ENSBTAG00000015191: VGDVVNLVSVDVQRLTESVTYLNGLWLPLIWIVVCFVYLWQLLGPSALTAIAVFVSLLPLNFFITKKRNHHQQEQMRQKDCRARLTSCILRNVRTVKYHGWEGAFLDRVLHIRAQELGAL : 476
Ggo.ENSGGOG00000009623: VGDVVNLVSVDVQRLTESVLYLNGLWLPLVWIVVCFVYLWQLLGPSALTAIAVFLSLLPLNFFITKKRNHHQEEQMRQKDSRARLTSSILRNSKTIKFHGWEGAFLDRVLGIRGQELGAL : 468
Hsa.ENSG00000091262: VGDVVNLVSVDVQRLTESVLYLNGLWLPLVWIVVCFVYLWQLLGPSALTAIAVFLSLLPLNFFISKKRNHHQEEQMRQKDSRARLTSSILRNSKTIKFHGWEGAFLDRVLGIRGQELGAL : 476
Ptr.ENSPTRG00000007815: VGDVVNLVSVDVQRLTESVLYLNGLWLPLVWIVVCFVYLWQLLGPSALTAIAVFLSLLPLNFFITKKRNHHQEEQMRQKDSRARLTSSILRNSKTIKFHGWEGAFLERVLGIRGQELGAL : 333
Oan.ENSOANG00000005123: VGEIVNLVSVDVQRLVDAVVYFNGIWICPIWIVICFIFLWQLLGPSALTALAVFLFLLPLNFVITKKRSRFQEEQMRQKDHRGTLTSSILSNVRIIKFHGWEKAFMEKVLHIRKEELQAL : 429
Mdo.ENSMODG00000005815: VGEIINLVSVDVQRLMDAVLYLNGLWLPVIWIIICFTFLWQLLGPSALTAIAVFLILLPLNFIITKKRSCFQEEQMQHKDRRARLTDSILRNMKIIKFHGWEEAFMEKILTIRKGELQAL : 480
Gga.ENSGALG00000006698: VGEIVNLVSVDVQKLMDLIIYFNGTWLAPIRIIICFVFLWQLLGPSALASIAVF-LFLPLNFMITKKRSHFQEAQMKHKDERATLTNAILSDIKVIKLYGWEKTFMEKVHAIRKQELQAL : 478
Aca.ENSACAG00000003478: VGEIVNLVSVDVQKLMDLIIYFNGTWLAPIRIVICFVFLWQLLGPSALMAVVVFLFLLPLNFVIAKKRTQFQEAQMAHKDSRAKLTSAILSDIKTLKLHGWEEAFVGRVMGVRTRELQAL : 418
Cel.WBGene00003413: VGEIVNLMAIDVERFQMITPQIQQFWSCPYQITFALVYLFITLGYSALPGVVIMVIFVPMNIISSMIVRKWQIEQMKLKDERTKMVNEVLNGIKVVKLYAWEVPMEAYIDEIRTKELALI : 475
Aga.AGAP008437: VGEIVNLMAVDAQRFFELTSYLHVLWSAPLIIALCIYLLYELLGPAVFAGLGVMVIMIPITGFIATRMRDLQVEQMKIKDERVKKMNEILGGIKVLKLYAWEPSFQDTVVTVRNEELDVL : 474
Aga.AGAP027980: VGEIVNLMAVDAQRFFELTSYMHILWSGVLIIALCVYLLYDILGAAVFAGLGVMILITPVSGVIATKMRDAQVAQMKIKDDRVKKMNEILGGIKVLKLYAWEPSFQDNILTVRKEEIGIL : 479
Aga.AGAP028128: VGEIVNLMAVDAQKFFELTSYLHILWSALLIIGLCVFLLYDILGPAVFAGLGVMILMTPVSGVVAAKLKTHQVAQMKLKDERVKKMNEILGGIKVLKLYAWEPSFQDSILNVRDEEVGIL : 479
Spu.026395: VGEIVNLMSVDAQRFMDLCTYLNMLWSGPFQISVALYFLWQTLGPSVLAGLGVMILLIPLNALVATQARKLQVKQMQYKDARIKLMSEVLSGIKVLKLYAWEESFQSKILAIRDKELKVL : 479
Hsa.ABCC2.ENSG000000238: VGETVNLMSVDAQKLMDVTNFMHMLWSSVLQIVLSIFFLWRELGPSVLAGVGVMVLVIPINAILSTKSKTIQVKNMKNKDKRLKIMNEILSGIKILKYFAWEPSFRDQVQNLRKKELKNL : 469
Xtr.ENSXETG00000026360: AGEIVNLISTDVQKLMDLATCVNYMWSAPVTIIVAMYFLWQTLGIAVLAGVAVFILNLPFMTVFAVIIKRVQEQQMKQKDGRIKIISEILQGIKVLKLYAWENAFMKKVTEFRLMELKAV : 464
Cel.WBGene00003410: VGEIVNLMAIDVDRFQQITPQTMQYWSNPFQIGLALFLLFQQLGVSVFSGVAVMVLLFPINFVITMIIRKWQIAQMYYKDERTKMVNEVLNGIKVIKLYAWEPPMEQVIEDLREQELGLI : 476
Ame.GB53134: VGEIVNLMSVDAQRFMDLTAYINMIWSAPLQIVLALYFLWDILGPAVLAGLAVLLILIPINVLITNRVKTLQIRQMKHKDERVKLMNEVLNGIKVLKLYAWEPSFEEQILKIRTKEIKVL : 474
Pma.KM232931_1: VGEIVNLVAVDAQKFNELPIYLNMLWSSPFQIVLAMYFLWQTLGPSVMAGVAIMILLIPVNGFIAVKTRSLQTEQMKLKDRRVKLMNEMLGGIKVLKLYAWELSFQAQVLEIRKQELSTL : 196
Gac.ENSGACG00000003037: VGEIINLVSADTQKLMDFVVYFNSLWVTPIEITLCFYFLWQLLGPSALAGIVPVVLIVPLNGLIAKMRSKLQEIQMKFTDGRLKLMNEILSGVKILKFYAWEDAFLRRIGVLRDGELETL : 480
Loc.ENSLOCG00000007152: VGEIVNLVSADTQKLMDFVVYFNAVWLAPIEIGLCLFFLWQHLGPSALAGIATVILIFPLNGFIAKQRSKLQEVQMKHMDGRIKLMNEILNGIKIIKFYAWEKAFLERVLGYRQKELKAL : 480
Gmo.ENSGMOG00000005748: VGEIVNLVSADTQKLMDFVVYFNAVWLAPIEIALCLFFLWQHLGPSALAGIATVILIFPLNGYIAKKRSKLQEVQMKFMDGRIRLMNEILSGIKILKFYAWEKAFLEQVLGYREKELQAL : 458
Gac.ENSGACG00000019172: VGEIVNLVSADTQKLMDFVVYFNAVWLAPIEISLCLFFLWQHLGPSALAGIATVILIFPLNGLIAKKRSKLQEVQMKFMDGRIRLMNEILSGIKILKFYAWEKAFLEQVLGLREKELKAL : 480
Tni.ENSTNIG00000012067: VGEIVNLVSADTQKLMDFVVYFNAVWLAPIEIALCLFFLWQQLGPSALAGIATVIFIFPLNGFIAKKRSKLQEIQMKFMDGRIRLMNEILNGIKILKFYAWEKAFLEQVLGHREKELRAL : 479
Ola.ENSORLG00000013429: VGEIVNLVSADTQKLMDFVVYFNAVWLAPIEIGLCLFFLWQHLGPSALAGIATVILIFPLNGFIAKKRSKLQEIQMKFMDGRVRLMNEILNGIKILKFYAWEKAFLEQVLGYREKELKAL : 479
Oni.ENSONIG00000018866: VGEIVNLVSADTQKLMDFVVYFNAVWLAPIEIALCLFFLWQHLGPSALAGIATVILIFPLNGFIAKKRSKLQEIQMKFMDGRIRLMNEILNGIKILKFYAWEKAFLEQVLGYREKELKAL : 479
Xma.ENSXMAG00000004906: VGEIVNLVSADTQKLMDFVVYFNAVWLAPIEIALCLFFLWQHLGPSALAGITTVILIFPLNGFIAKKRSKLQEIQMKFMDGRIRLMNEILNGIKILKFYAWEKAFLEQVLGYREKELKAL : 479
Dla.DLAgn_00177430: VGEIVNLVSADTQKLMDFVVYFNAVWLAPIEIALCLFFLWQHLGPSALAGIATVILIFPLNGFIAKKRSKLQEIQMKFMDGRIRLMNEILNGIKILKFYAWEKAFLEQVLGHREKELKAL : 479
Dre.ENSDARG00000016750: VGEIVNLVSADTQKLMDFVVYFNAVWLAPIEVTLCLFFLWQHLGPSALAGIATVIFIFPLNGFIARKRSKLQEIQMKYMDGRVKLMNEILNGIKILKFYAWEKAFLEQVLGYREKELKTL : 478
Ame.ENSAMXG00000004837: VGEIVNLVSADTQKLMDFVVYFNAVWLAPIEIALCLFFLWQHLGPSALAGIATVILIFPLNGFIAKKRSKLQEIQMKFMDGRIKLMNEILNGIKILKFYAWEKAYLEKVLGYREKELGAL : 477
Ame.ENSAMXG00000003085: VGEIVNLVSADTQKLMDFVVYFNAVWLAPIEIGLCLFFLWQRLGPSALAGITTVILIFPLNGLIAKMRSKLQEVQMKYMDGRIKLMTEILSGIKILKFYAWEKAFEERVLGYREKELKAL : 480
Dre.ENSDARG00000094901: VGEIVNLVSADTQKLVDFVMYFNALWLAPIEIALCLFFLWQHLGPSTLAGITTVILIFPLNGFIAKMRSKLQEVQMKHKDERIKLMNEILSGIKILKFYAWEKAFRERVLGYREKELNAL : 453
Dre.ENSDARG00000095820: VGEIVNLVSADTQKLMDFVVYFNAVWLAPIEIALCLFFLWQHLGPSALAGIAIVILIFPLNGFIAKMRSKLQEVQMRYMDGRIKLMNEILSGIKILKFYAWENAFRERVLEYREKELNAL : 480
Lch.ENSLACG00000022117: VGEVVNLVSVDIQKLMDLIIYFNGVWLAPLEIALCFYFLWQYLGPSSLAGVVAIFVVFSLNGVIAKKRSKFQEEQMRCKDERVKHTYQLLGGIKVLKFHAWETALMEKVLGIRQKELKVL : 480
Dpu.347281: VGEIVNLMSVDAQRLMDLTTYLNMLWSAPLQIALAIYFLYQILGPSVFAGLGVMILLIPINGVLANATKKLQIQQMKYKDKRVKMMSEILSGIKVLKLYAWEPSFQAQVEDIRNKEIKVL : 480
Lca.KE993868_1: ------------------------------------------------------------------------------------------------------------------------ : -
Pma.KM232930_1: GGELTTLMSVDVQHMADITNNLNFVWSAPLHICLAMYFLWQHLGPSVLAGVAVMLLLIPFNAFIALNIRTLQAQIIKKRDERVRTLSEALSGVRIIKMYAWEGRFRDVILRVREHELAVL : 480
Csa.ENSCSAVG00000008135: VGEVVNLMSVDAQRFMDLMSYLNIVWSGPFQIILALYFLWNILGPSVLAGLAVMVLLIPVNGVIAAKARALQVKQMKYKDERIKLMNEILNGMKVLKMYAWEMSFKDKVTSIRNKELKEL : 471
Csa.ENSCSAVG00000003792: VGEVVNLMSVDAQRFMDLMTYLNTVWSGPFQIILAMYFLWQYLGAAVLAGLGVMLLLIPINALIARKTHQLQVRQMKLKDERIKLMNEILNGIKVLKMYAWEMSFKDKVKAIRDKELDVL : 412
Cin.ENSCING00000020698: --------------------------------------------------------------------------------------------------YAWEMSFKEKVQTIRDKELSVL : 23
Tca.TC012253: VGEIVNLMSVDAQKFIDLTAYINMIWSAPLQIILALYFLWNILGPAVLAGLAVMIILIPVNGYIANKVKVLQIKQMKNKDERVKLMNEVLSGIKVLKLYAWEPSFEKQILKIRTKEIQVL : 475
Aga.AGAP009835: VGEIVNLMAVDAQRFMDLTTYINMIWSAPLQIGLALYFLWQILGPSVLAGLAVMIILIPVNGVIANMIKTLQIKQMKNKDERVKLMNEVLSGIKVLKLYAWEPSFEQQILKIRDKEVKVL : 474
Lgi.LotgiG107213: VGEIVNLMSVDAQRFMDLMTYFHTIWSGPLQIAIAIYFLWQTLGPSILAGLGVMILLIPINAVMANKTKEFQFEQMLLKDKRIKLMNEILNGIKVLKLYAWEPSFEEQVLGIRKEELYVL : 478
Bfl.90918: AGNMVNLMTSDVTKIQMLCQSLHSVWAAPLQIIVAMYFLWQTLGPSTLAGLGVMIILIPINGVIAGGTRKQVTKVMKKKDSRLKLLNEVLNGIKVLKLYAWELSFRQKIEALRRKELQYN : 361
Dme.FBgn0032456: VGEIVNLMAVDAQRFMELTTYLNMIWSAPLQIGLALYFLWQQLGPSVLAGLAVMIILIPVNGVIASRIKTYQIRQMKYKDERVKLMNEVLSGIKVLKLYAWEPSFEKQVLDIRDKEIATL : 479
Oan.ENSOANG00000013379: ------------------------------------------------------------------------------------------------------------------------ : -
Pma.AKC42143_1: ------------------------------------------------------------------------------------------------------------------------ : -
Lca.KE994284_1: -----------------------------------------------------------------------QVEQMKYKDARIKLMNEILNGIKVLKLYAWEPSFQDMVLEIRNKELQVL : 208
Cmi.SINCAMG00000015787: IGEIVNLMSVDAQRLMDLITYINMMWSAPLQVTLAMYFLWQNLGPSVLAGVAVMILLVPINSMIAMKTKDLQVTQMKEKDNRIKLMNEILNGIKVIKLYAWELAFKEKVMQIRRKELKVL : 479
Ler.ctg13956: ------------------------------------------------------------------KTKTLQVTQMKEKDNRIKLMNEVLNGIKVIKLYAWELAFKEKVLDIRENELKVL : 54
Rty.XP_020377569_1: ------------------------------------------------------------------------------------------------------------------------ : -
Sca.ctg67278: VGEIVNLMSVDAQKFMDLITYLNMIWSAPLQVILAMYFLWQNLGPSVLAGVAVMVLLVPINGVIAVKTKNLQVTQMKEKDNRIKLMNEVLNGMRVLKLYAWELAFKEKVLQIRQKELQVL : 380
Lch.ENSLACG00000001471: VGEIVNLMSVDAQRFMDLVTYINMIWSAPLQVILALFFLWQNLGPSVLAGVAVMVLMVPVNAFIAMKSKTYQVTQMKSKDNRIKLMNEILNGIKVLKLYAWELAFKDKVLEIRQQELQVL : 480
Tni.ENSTNIG00000005013: VGEIVNLMSVDAQRFMDLITYINMIWSAPLQVVLALYFLWQTLGPSVLAGVGVMVLMVPVNAVIAMKTKTYQVAQMKNKDSRIKLMNEMLNSIKVLKLYAWELAFKDKVSEIREHELHVL : 480
Loc.ENSLOCG00000007196: VGEIVNLMSVDAQRFMDLVTYINMIWSAPLQVILALYFLWQNLGPSVLAGVAVMVFMVPVNAVIAMKSKTYQVAQMKSKDSRIKLMNEVLNGIKVLKLYAWELAFQGKVLGIRETELRVL : 480
Dre.ENSDARG00000104719: VGEIVNLMSVDAQRFMDLITYINMIWSAPLQVILALYFLWQNLGASVLAGVAVMVLMVPLNAVIAMKTKTYQVAQMKSKDNRIKLMNEVLNGIKVLKLYAWELAFKGKVSAIRESELRVL : 480
Ame.ENSAMXG00000002943: VGEIVNLMSVDAQRFMDLITYINMIWSAPLQVILALYFLWQNLGPSVLAGVAVMVLMVPVNAVIAMKTKTYQVAQMKSKDNRIKLMNEVLNGIKVLKLYAWELAFKDKVSTIRESELRVL : 480
Xma.ENSXMAG7738/17319: VGEIVNLMSVDAQRFMDLVTYINMVWSAPLQVVLALYFLWQNLGPSVLAGVAVMVLMVPINAVIAMKTKTYQVAQMKSKDSRIKLMNEMLNGIKVLKLYAWELAFKEKVSKIRESELRVL : 453
Ola.ENSORLG00000017141: VGEIVNLMSVDAQRFMDLVTYINMIWSAPLQVVLALYFLWENLGPSVLAGVAVMILMVPINAVIAMKTKTYQVAQMKNKDSRIKLMNEMLNGIKVLKLYAWEMAFKEKVSQIRENELKVL : 480
Gac.ENSGACG00000000434: VGEIVNLMSVDAQRFMDLITYINMIWSAPLQVVLALYFLWQNLGPSVLAGVAVMVLMVPINAVIAMKTKTYQVAQMKSKDSRIKLMNEMLNGIKVLKLYAWELAFKDKVSKIRESELQVL : 480
Oni.ENSONIG00000007824: VGEIVNLMSVDAQRFMDLITYINMIWSAPLQVVLALYFLWQNLGPSVLAGVAVMVLMVPVNAVIAMKTKAYQVAQMKSKDNRIKLMNEMLNGIKVLKLYAWELAFKGKVSEIRESELRVL : 480
Dla.DLA_00195360: VGEIVNLMSVDAQRFMDLITYINMIWSAPLQVVLALYFLWQNLGPSVLAGVAVMVLMVPVNAVIAMKTKTYQVAQMKSKDNRIKLMNEMLNGIKVLKLYAWELAFKDKVSEIRESELRVL : 434
Xtr.ENSXETG00000019661: IGEIVNLMSVDAQRFMDLATYINMIWSAPLQVILAFYLLWQNVGPSVLAGIAVMVVMLMLVAPCIFSAAVTSVVQMKCKDNRIKLMNEILNGIKVLKLYAWELAFKEKVLGIRKDELKVL : 480
Aca.ENSACAG00000005349: VGEIVNLMSVDAQRFMDLATYINMVWSAPLQVILALYLLWQNLGPSVLAGVAVMLLLVPVNAVIAMKTKTYQVAHMKSKDNRIKLMNEILNGIKVLKLYAWELAFKEKVLGIRKEELRVL : 480
Gga.ENSGALG00000006646: VGEIVNLMSVDAQRFMDLATYINMIWSAPLQVILALYLLWRNLGPSVLAGVAVMILLVPINAVMAMKTKTYQVAQMKSKDNRIKLMNEILNGIKVLKLYAWELAFREKVLEIRQKELKVL : 480
Dno.ENSDNOG00000014990: VGEIVNLMSVDAQRFMDLATYINMIWSAPLQVVLALYLLWLNLGPSILAGVAVMILMVPINAVMAMKTKTYQVAHMKSKDNRIKLMNEILNGIKVLKLYAWELAFQEKVSAIRKEELKVL : 480
Mmu.ENSMUSG00000023088: VGEIVNLMSVDAQRFMDLATYINMIWSAPLQVILALYFLWLSLGPSVLAGVAVMILMVPLNAVMAMKTKTYQVAHMKSKDNRIKLMNEILNGIKVLKLYAWELAFQDKVMSIRQEELKVL : 480
Ggo.ENSGGOG00000004324: VGEIVNLMSVDAQRFVDLATYINMIWSAPLQVIVALYLLWLNLGPSVLAGVAVMVLMVPVNAVMAMKTKTYQVRHVSLRAPKPG------------------------------------ : 417
Hsa_ENSG00000103222: VGEIVNLMSVDAQRFMDLATYINMIWSAPLQVILALYLLWLNLGPSVLAGVAVMVLMVPVNAVMAMKTKTYQVAHMKSKDNRIKLMNEILNGIKVLKLYAWELAFKDKVLAIRQEELKVL : 480
Ptr.ENSPTRG00000007812: VGEIVNLMSVDAQRFMDLATYINMIWSAPLQVILALYLLWLNLGPSVLAGVAVMVLMVPVNAVMAMKTKTYQVAHMKSKDNRIKLMNEILSGIKVLKLYAWELAFKDKVLAIRQEELKVL : 434
Cluf.ENSCAFG00000018208: VGEIVNLMSVDAQRFMDLATYINMIWSAPLQVILALYLLWLNLGPSVLAGVAVMILMVPLNAVMAMKTKTYQVAHMKSKDNRIKLMNEILNGIKVLKLYAWELAFKDKVLAIRQEELKVL : 480
Bta.ENSBTAG00000021090: VGEIVNLMSVDAQRFMDLATYINMIWSAPLQVILALYLLWLNLGPSVLAGVAVMVLMVPLNAVMAMKTKTYQVAHMKSKDNRIKLMNEILNGIKVLKLYAWELAFKDKVLAIRQEELKVL : 480
Mdo.ENSMODG00000004194: VGEIVNLMSVDAQRFMDLATYINMIWSAPLQVILALYLLWLNLGPSVFAGVAVMILMVPLNAVMAMKTKTYQVAHMKSKDNRIKLMNEILNGIKVLKLYAWELAFKEKVLEIRQEELKVL : 480
Oan.ENSOANG00000005124: VGEIVNLMSVDAQRFMDLATYINMIWSAPLQVILALYLLWLNLGPSVLAGVAVMILMVPINAVMAMKTKTYQVAHMKSKDNRIKLMHEILNGIKVLKLYAWELAFKKKVLEIRQEELKVL : 269
Bfl.232174: VGEIINLMSVDAQRFMDLCSHINMLWSAPFQIAVSLYFLWQTLGASILAGLGVMILLIPVNAVLGNIIKKLQVKQMKHKDTRMKLMNEVLNGIKVLKLYAWELSFREKIEKIRSKELQIL : 479
Bfl.118638: VGEIINLMSVDAQRFMDLCSHINMLWSAPFQIAVSLYFLWQTLGASILAGLGVMILLIPVNAILGNIIKKLQVKQMKHKDTRMKLMNEVLNGIKVLKLYAWELSFREKIEKIRSKELQIL : 480
Bfl.118636: VGEIVNLMSVDAQRFMDLSTYLHMIWSAPFQIAVSLYFLWQTLGPSILAGLGVMILLIPINIVMANKTKQLQVKQMIQKDARIKLMNEVLNGIKVLKLYAWELSFKEKIEKIRSKELQIL : 480
Bfl.128060: VGEIVNLMSVDAQRFMDLSTYLHMIWSAPFQIAVSLYFLWQTLGPSILAGLGVMILLIPINIVMANKTKQLQVKQMIQKDARIKLMNEVLNGIKVLKLYAWELSFKEKIEKIRSKELQIL : 480
Dre.ENSDARG00000096662: VGEVVNLMSVDAQRFMDLTTFLNMLWSAPLQIFLALFFLWQNLGPSVLAGVAVMVLLIPFNAFIAMKTRTYQVEQMKYKDDRIKLMNEILNGIKVLKLYAWEPSFRDKILQIRQKELHVL : 478
Ame.ENSAMXG00000016253: VGEVVNLMSVDAQRFMDLTTFLNMLWSAPLQIILALFFLWQTLGPSVLAGVAVMILLIPFNAVIAMKTRAYQVEQMQYKDARIKLMNEILNGIKVLKLYAWEVSFKEKVLQIRQKELNVL : 479
Ola.ENSORLG00000020741: VGEIVNLMSVDAQRFMDLTAFLNMLWSAPLQIMLALYFLWENLGPSVLAGVAVMVMLIPLNAFIAMKTRAYQVEQMQHKDARLKLMNEILNGIKVLKLYAWEESFKQKVLDIRQKELNVL : 480
Gmo.ENSGMOG00000010029: VGEIVNLMSVDAQRFMDLTTFLNMLWSAPLQIILALYFLWQNLGPSVLAGLAVMVLLIPLNAAIAVKTRAYQVQQMQHKDARIKLMSEILNGIKVLKLYAWEGSFQEKVLAIRQKELDVL : 434
Xma.ENSXMAG00000012203: VGEIVNLMSVDAQRFMDLTTFLNMLWSAPLQIMLALYFLWQNLGPSVLAGVAVMVMLIPFNAVIAMKTRAYQVEQMQHKDARIKLMNEILNGIKVLKLYAWENSFKEKVLAIRQKELNVL : 480
Gac.ENSGACG00000005901: VGEVVNLMSVDAQRFMDLTTFLNMLWSAPLQIMLALYFLWQNLGPSVLAGVAVMVMLIPLNAVIAMKTRAYQVEQMQHKDSRIKLMNEILNGIKVLKLYAWENSFKEKILAIRQKELNVL : 474
Oni.ENSONIG00000019586: VGEIVNLMSVDAQRFMDLTTFLNMLWSAPLQIMLALYFLWQILGPSVFAGVAVMILLIPFNAFIAMKTRAYQVEQMQHKDARIKLMNEILNGIKVLKLYAWENSFKQKVLAIRQKELIVL : 477
Dla.DLAgn_00098120: VGEVVNLMSVDAQRFMDLTTFLNMLWSAPLQIILALYFLWQNLGPSVMAGVAVMIMLIPFNAVIAMKTRAYQVEQMQYKDSRIKLMNEILNGIKVLKLYAWENSFKEKVLAIRQKELNVL : 480
Cmi.SINCAMG00000013336: VGEIVNLMSVDAQRFMDLTTFLNMLWSAPLQICLALYFLWQSLGPSVLAGVAVMILLIPINAFIAMKTRSFQVEQMGYKDSRIKLMNEILNGIKVLKLYAWEPSFQQQILSIRNKELRVL : 380
Ler.ctg12190: VGEIVNLMSVDAQRFMDLTGFLNMLWSAPLQICLALYFLWQYLGPSVLAGVAVMILLIPFNAMIAMKCRSFQVEQMKYKDSRIKMMNEILNGIKVLKLYAWEESFAQKVLAIRSKELNIL : 380
Sca.ctg14163: VGEIVNLMSVDAQRFMDLTGFLNMLWSAPLQICLALYFLWQYLGPSVLAGVAVMVLLIPFNAVIAMKSRSFQVQQMQHKDSRIKLMNEILNGIKVLKLYAWEKSFEQKVLAIRQKELNIL : 330
Rty.XP_020375725_1: ------------------------------------------------------------------------------------------------------------------------ : -
Sac_EU250283: VGEIVNLMSVDAQRFMDLTSFLNMLWSAPLQICLALYFLWQYLGPSVLAGVAVMILLIPFNAVIAMKSRSFQVQQMLHKDSRIKLMNEILNGMKVLKLYAWEPSFEQKVLAIRQKELDIL : 480
Tni.ENSTNIG00000004171: K----TIFCDNVQGLRAVTCLVKFVT-SFVSTIMPTAASFQNLGPSVLAGVAVMVMLIPLNAVIAMKTRAFQVEQMQYKDSRIKLMNEILNGIKVLKLYAWENSFRDKVLAIRQKELNVL : 452
Aca.ENSACAG00000001396: VGEIVNLMSVDAQRFMDLTTFLNMLWSAPLQICLALYFLWQSLGPSVLAGVAVMVLLIPLNAVIAMKTRAFQVEQMRYKDSRIKLMNEILSGIKVLKLYAWEPSFADKILEIRKNELRVL : 477
Gga.ENSGALG00000007522: VGEIVNLMSVDAQRFMDLVTFLNMLWSAPLQTCLALYFLWQALGPSVLAGVAVMVLLIPFNSAVAIKTRAFQVEQMRYKDSRIKLMNEILGGIKVLKLYAWEPSFSEKVLEMRKNELRVL : 428
Xtr.ENSXETG00000012239: VGEVVNLMSVDAQRFQDLTTFLNMLWSAPLQICLALYFLWQALGPSVLAGVAVMVLLIPINAFIAMKTRAFQVEQMQYKDSRIKLMNEILNGIKVLKLYAWEPSFAQKVLEIRNKELNIL : 480
Loc.ENSLOCG00000010918: VGEIVNLMSVDAQHFMDLTTFLNLLWSAPLQIILALYFLWQKLGPSVLAGVAVMVLLIPFNAVIAMKTRAFQMEQMQYKDARIKLMNEILNGIKVLKLYAWENSFKQKIMDIRQNELKVL : 480
Lch.ENSLACG7209/6619: VGEIVNLMSVDAQRFMDLTTFLNMLWSAPLQICLALYFLWQKLGPSVLAGVAVMILLIPFNAIIAMKTRAFQVELMLYKDSRIKLMNEILNGIKVLKLYAWEPSFLKKVQEVRKNELKVL : 474
Mdo.ENSMODG00000020910: VGEIANLMSVDAQRFSDLVSFLNMLWSAPLQIILAIYFLW-------------------------------------------------------------------------------- : 400
Dno.ENSDNOG00000046300: VGETVNLMSVDAQRFMDTMQSLNLLWSIPLQIILAIYFLWQHLGPSVLAGVAVMVLMIPVNAAIAMKIQAFQVEHMKLQDLRVKVMSEILGGIKVLKLYPWEPSFSEQVESVRQGELRLL : 480
Mmu.ENSMUSG00000020865: VGEMVNLMSVDAQRFMDVSPFINLLWSAPLQVILAIYFLWQILGPSALAGVAVIVLLIPLNGAVSMKMKTYQVKQMKFKDSRIKLMSEILNGIKVLKLYAWEPSFLEQVKGIRQSELQLL : 480
Ggo.ENSGGOP00000003354: VGEIVNLMSVDAQRFMDLAPFLNLLWSAPLQIILAIYFLWQNLGPSVLAGVAFMVLLIPLNGAVAVKMRAFQVKQMKLKDSRIKLMSEILNGIKVLKLYAWEPSFLKQVEGIRQGELQLL : 480
Hsa.ENSG00000108846: VGEIVNLMSVDAQRFMDLAPFLNLLWSAPLQIILAIYFLWQNLGPSVLAGVAFMVLLIPLNGAVAVKMRAFQVKQMKLKDSRIKLMSEILNGIKVLKLYAWEPSFLKQVEGIRQGELQLL : 480
Ptr.ENSPTRG00000009406: VGEIVNLMSVDAQRFMDLAPFLNLLWSAPLQIILAIYFLWQNLGPSVLAGVAFMVLLIPLNGAVAVKMRAFQVKQMKLKDSRIKLMSEILNGIKVLKLYAWEPSFLKQVEGIRQGELQLL : 480
Cluf.ENSCAFG00000017201: VGEIVNLMSVDAQRFMDLAPFLNLVWSAPLQIILAIYFLWQNLGPSILAGVAFMVLLIPLNGAVAVKMRAFQVEQMKFKDSRIKLMSEILGGIKVLKLYAWEPSFLEKVEGIREDELRLL : 480
Bta.ENSBTAG00000020070: VGEIVNLMSVDAQRFMDVVPFINLLWSAPLQIILAVYFLWQNLGPSVLAGVALMVLLIPLNGAVAVKMRALQVEQMKFKDSRIKLMSEILGGIKVLKLYAWEPSFLKQVEGIRQDELRLM : 480

Hsa.CFTR.ENSG0000000162: RKAAYVRYFNSSAFFFSGFFVVFLSVLPYALIK---GIILRKIFTTISFCIVLRMAVTQFPWAVQTWYDSLGAINKIQDFLQKQEY--KTLEYNL-TTTEVVMENVTAFWEEGGTPVLKD : 405
Hsa.ABCC5.ENSG000001147: EKAGYFQSITVGVAPIVVVIASVVTFSVHMTLG--FDLTAAQAFTVVTVFNSMTFALKVTPFSVKSLSEASVAVDRFKSLFLMEEV--HMIKNKPAPHIKIEMKNATLAWDSSPSPTLHS : 495
Hsa.ABCC11.ENSG00000121: EKCGLVQSLTSITLFIIPTVATAVWVLIHTSLK--LKLTASMAFSMLASLNLLRLSVFFVPIAVKGLTNSKSAVMRFKKFFLQESP-VFYVQTLQDPSKALVFEEATLSWQQTLGPELHK : 482
Hsa.ABCC12.ENSG00000140: EKAGFVQSGNSALAPIVSTIAIVLTLSCHILLR--RKLTAPVAFSVIAMFNVMKFSIAILPFSIKAMAEANVSLRRMKKILIDKS-PPSYITQPEDPDTVLLLANATLTWEHESPPVLHS : 446
Hsa.ABCC8.ENSG000000060: RAFAIYTSISIFMNTAIPIAAVLITFVGHVSFFKEADFSPSVAFASLSLFHILVTPLFLLSSVVRSTVKALVSVQKLSEFLSSAEIREDCRGLTGPDNCCVQIMGGYFTWTPDGIPTLSN : 593
Hsa.ABCC9.ENSG000000694: KTFALYTSLSIFMNAAIPIAAVLATFVTHAYAS-GNNLKPAEAFASLSLFHILVTPLFLLSTVVRFAVKAIISVQKLNEFLLSDEIQPGRYHLDSYEDIAIKVTNGYFSWGS-GLATLSN : 594
Hsa.ABCC10.ENSG00000124: RVIKYLDAACVYLWAALPVVISIVIFITYVLMG--HQLTATKVFTALALVRMLILPLNNFPWVINGLLEAKVSLDRIQLFLDLPNHNPQAYYSPDPPSTVLELHGALFSWDPVGT-SLET : 585
Hsa.ABCC4.ENSG000001252: LRSSCLRGMNLASFFSASKIIVFVTFTTYVLLG--SVITASRVFVAVTLYGAVRLTVTFFPSAIERVSEAIVSIRRIQTFLLLDEISQRNRQLPSDGKKMVHVQDFTAFWDKAETPTLQG : 413
Cel.WBGene00003414: RNVCILSRIVDVANAASPFLVAIGSFTCYVLWSDENGLTPSVAFVALTIFNQLRQPMRMVANLINTLVQARVSNKRLRQFLNDEEM-----ERKTELGNAIVFKNASLNWKGPNPPVLKD : 553
Cel.WBGene00003409: KQSALLKTFADCLNVGAPVFVALSSFTVFVLIDPKNVLTPNIAFVSLSLFNLLRGPLMMAAELVAQTVQLVVSNKRVRTFLCEKEVDTAAIDKEIRYTNTVEIHSGSFAWDSAEARILSD : 595
Hro.HelroG163344: KKYSYLGAVILFSWSCAPFLVTLAAFATYVLVG--GQLDANKAFTALSLLNILRVPISILPMMISYLVMAAVSVKRIEKYLQTPDLDEKSVLRLNEDPYSIIIESGTFAWGRGDANVLNN : 556
Hro.HelroG157076: KKYYYYVAVSIFSWSCAPALV-----------------RWSHAFTALSLFNILKEPMFMFPTMMSYIVMGFVSLRRIEKFLQCADVNPNNVIELEE-----------------DKDDDKD : 275
Bfl.230771: RHIGYTRAVQGFIWNFTSPLVSFAIFSAYVLTDDNNVLDAEKVFLVYSLMETMKFSFGVLPHLYINIQQAKVSLGRIEDFLRQDELHPDDVRRDM-PGPPISVREGTFTWGKEDEPILKD : 586
Lgi.LotgiG105097: TKNAFLAATTIFCWIGVPVLVTLATFAMFILMSENQHLDAETAFVALSLFNILRIPINHLPMCISALINAQVSLGRLGKYLSGSDLDEKNCGHNQNTENAVEITEGTFTWDRDMPPSLKN : 586
Cel.WBGene00003407: KKLSYLNAATTLSWACAPFLVAVLTFGLYVLWDENNVLTPQITFVALALFNILRFPLAVFAMVFSQAVQCSASNTRLKEFFAAEEMSPQTSIAYGGTDSAIKMDGGSFAWGSKEEDKLHD : 595
Cel.WBGene00003408: KKLSYFMAAIVFSWICAPFLASVISFVVYVYLDENNVLTPEITFVALSLFDILRMPLAMVAMVYGEAVQCSVSNTRLKEFFAAEEMSPQTSISHGETDSAIEVENGLFSWSSDEDPTLRE : 594
Lgi.LotgiG153611: KKIANLNTLSTFVWTCAPYLVTLATFTCYVLTSSTGHLDAQKAFVTLSLFNILQFPINFIPETISYLSQAAASVQRIEIFLREEELSRSNVDKNDFMERAIKVDRGVFAWNKSSRPVLHR : 547
Lgi.LotgiG110718: YKIAYTYIVLSMSWAVAPFLVTFATFATYILMNENNVLDAQKAFVTLSLFNLLRVPLNLLGIIINFSIQAFVSVKRINAFIIQPDLDPENSVQDQSSKFAISVRDGEFKWDEDMPSVLHN : 409
Dno.ENSDNOG00000024923: RTSGLLFSVSLVSFQASTFLVALVVFAVHTLVAEENAMDAEKAFVTLTVLNILNKAQAFLPFSIHSVVQARVSFDRLAAFLCLEEVDPGAVVSMPFGKAGITVRNGTFAWSRESPPCLQR : 537
Mmu.ENSMUSG00000030834: KTSTLLFSVSLVSFQVSTFLVALVVFAVHTLVAEDNAMDAEKAFVTLTVLSILNKAQAFLPFSVHCIVQARVSFDRLAAFLCLEEVDPNGMIASNSSKDRISVHNGTFAWSQESPPCLHG : 596
Cluf.ENSCAFG00000018197: RTSSLLFSVSLVSFQVSTFLVALVVFAVHTLVAEENAMDAEKAFVTLTVLSILNKAQVFMPFSINSVVQARVSFDRLAAFLCLEELDLRAVDLSPSGETCIRVHDGTFAWSREGTPCLRR : 596
Bta.ENSBTAG00000015191: KTSSLLFSVSLVSFQVSTFLVALVVFAVHTLVAEENAMDAEKAFVTLTVLNILNKAQAFLPFSIHSIVQARVSFDRLAAFLSLEETDPGAVDSSPSGEDCISIQEGTFTWSQESAPCLRR : 596
Ggo.ENSGGOG00000009623: RTSGLLFSVSLVSFQVSTFLVALVVFAVHTLVA-ENAMDAEKAFVTLTVLNILNKAQAFLPFSIHSLVQARVSFDRLVAFLCLEEVDPGAVDSSSSGKDCITIHSATFAWSQESPACLHR : 587
Hsa.ENSG00000091262: RTSGLLFSVSLVSFQVSTFLVALVVFAVHTLVA-ENAMNAEKAFVTLTVLNILNKAQAFLPFSIHSLVQARVSFDRLVTFLCLEEVDPGVVDSSSSGKDCITIHSATFAWSQESPPCLHR : 595
Ptr.ENSPTRG00000007815: RTSGLLFSVSLVSFQVSTFLVALVVFAVHTLVA-ENAMDAEKAFVTLTVLNILNKAQAFLPFSIHSLVQARVSFDRLVTFLCLEEVDPGAVDSSSSGKDCITIHSATFAWSQESPPCLHR : 452
Oan.ENSOANG00000005123: KKSGLLFSVSLVSFHLSTFLVALVMFAVYTLSDENNVLDAQKAFVALMLINILNKAQGFLPLSLHTTIQAKVSLARLAAFLSLEEIEPNAVDTSPKSGECITIENGTFAWSRESSPCLRR : 549
Mdo.ENSMODG00000005815: KNSGFLFAVSLVSFHLSTFLVALVMFAVHALTDEKHVLDAEKAFVALTIINILNRAQAFLPFSINTIFQAWVSLARLAAFLHLEEVEPRAISTTPVGEESISVQDGTFAWSQENSPCLQR : 600
Gga.ENSGALG00000006698: KRSQILFSASLASFHSSTFLIAFVMFAVYTLVDNTHVLDAQKAFVSLTLINILNTAHSFLPFSINAAVQAKVSLKRLAAFLNLEELNPESSNRHTSGELFIIIRNGTFCWSKDTSPCLRR : 598
Aca.ENSACAG00000003478: RRSQFLFSASLVSFQSSTFLISFIMFAVYTLADERNIFSAQKAFVSLALVNILNTAHSFLPFSINSVVQAKVSLNRLAAFLSLEDLDQTNAEPGSLTQDCITIRNGTFTWSRESPPCLKR : 538
Cel.WBGene00003413: KKSAMVRNILDSFNTASPFLVALFSFGTFVLSNPSHLLTPQIAFVSLALFNQLRSPMTMIALLINQAVQAVVSNKRLKEFLVAEELDEKCVDRSVNSHNAVRVENLTASWDPEAGETLQD : 595
Aga.AGAP008437: KSAAYYGAGTYFVWTMAPFLVTLASFAVYVMIDEENVLDPQTAFVALALFNILRFPLAMFPMMITFAMQAWVSIKRIDKFMNSEELDPNNVTHNKS-ENALEVKDGTFSWGD-DAPTLKN : 592
Aga.AGAP027980: KRMAYYGAGIYFTFTIAPFLVTLVSFAVYVLMDEENILDPQTAFVSLALFNILRFPLGMLPMMVTFSMQAWVSVKRIDKFLNSAELDPSNVSNNKS-DEALTIKDGTFSWGDE-TPTLKN : 597
Aga.AGAP028128: KKMAYYGAGIFFTFTIAPFLVTLVSFAVYVLIDENNVLDPQTAFVSLALFNIMRFPLGMFPMVVTFSMQAWVSIKRIDKFLNSAELDPNNVTHNKS-DEALTIKDGTFSWGDE-TPTLKN : 597
Spu.026395: RLAAYLNAFTSFTWTCAPVLVSVTTFAVYVISDENNILDAEKAFVSIALFNILRFPLSIMPNLISNMVQTSVSLKRLEKFLKNEQLDPQNVDHFNMPGHSITVDSGHFTWDREEKTTLTN : 599
Hsa.ABCC2.ENSG000000238: LAFSQLQCVVIFVFQLTPVLVSVVTFSVYVLVDSNNILDAQKAFTSITLFNILRFPLSMLPMMISSMLQASVSTERLEKYLGGDDLDTSAIRHDCNFDKAMQFSEASFTWEHDSEATVRD : 589
Xtr.ENSXETG00000026360: KTGALLLSGALAVFVASPFWVSLTMFGVFLALDEKNILDAEKAFVTIILLNILRIPLRMFPMAITLFAQSSVSLKRMVKFFSAEELEPESVDINDSLEHAITIRHGTFTWSSSEPPCLQS : 584
Cel.WBGene00003410: KKAAFLRTFSDMLNTASPFLVALSTFATFIYIDPKNVLTPEIAFVSLTLFNQLRSPMSQVAELITQTVQVVVSNRRLKEFLVSEELNVEAIDHRARNNDVICLKEACLSWESAQPVTLTN : 596
Ame.GB53134: KETAYLNSGTSFIWSFAPFLVSLVSFATYVLIDENNRLDSTKAFVSLSLFNILRFPLSILPMMIGNMVQAYVSVKRINKFMNTEELDPNNVQHDSSESYTLLIENGTFIWDMEDRPTLRN : 594
Pma.KM232931_1: RKMAFLSALSIFCWACAPFLVALTTFGVYVSVDARNVLSAEKAFVSLSLFNILRFPLNLLPLVISNLVQASVSLKRLQAFLSHEELDPSNVERNNVPGVSISITNGTFKWDRCDAPVLHN : 316
Gac.ENSGACG00000003037: KMSQVLHSVSLASFNSSSFLIALSVFAVYVTIDDRNLLDAQKIFVSVALINILKTPLSQLPFAMSATMQAVVSLRRLGNFLSQDELKGDSVERLPRDGDAVRIEDGWFSWTCDGPPCLQG : 600
Loc.ENSLOCG00000007152: KKSQILYSISIASFNSSTFLIAFAMFGVYVLIDEKNVLDAQKVFVSMALINILKTPLSQLPFAMSTTMQAFVSLKRLGKFLCQEELKADNVERAPLEGGSVVVENGTFSWTREGSPCLRR : 600
Gmo.ENSGMOG00000005748: KKSQVLYSISFASFNCSSFLIAFAMFGVYVTIDARNVLDAQKVFVSMALINILKTPLSQLPFVMSTTMQ-------LGKYLCSEELKGDNVTKAAYDGEDVSIDNGTFSWSGEGPPCLKR : 571
Gac.ENSGACG00000019172: KKSQILYSISIASFNSSSFLIAFSMFGVYVMLDDRNVLDAQKVFVSMALINILKTPLSQLPFAISTTMQAMVSLRRLGKYLCSEELREDNVSKAPFDGEDVVIENGNFSWSAEGPPCLKR : 600
Tni.ENSTNIG00000012067: KKSQILYSISIASFNSSSFLIAFAMFGVYVMLDNRNVLDAQKVFVSMALINILKTPLSQLPFAISTTMQAMVSLRRLGKYLCSGELKADNVSKAPRDGENVVIENGTFSWSAAGPPCLKR : 599
Ola.ENSORLG00000013429: KKSQVLYSISIASFNSSSFLIAFAMFGVYVMLDERNVLDAQKVFVSMALINILKTPLSQLPFAISTTMQALVSLRRLGKYLCSEELKVDGVSKALSDGEDLVIENGTFSWSKEGPPCLKR : 599
Oni.ENSONIG00000018866: KKSQILYSISIASFNSSSFLIAFAMFGVYVMLDDKNVLDAQKVFVSMALINILKTPLSQLPFAISTTLQAVVSLKRLGKYLCSEELKMENVSKAPLDGEDVVIENGTFSWSAEGPPCLKR : 599
Xma.ENSXMAG00000004906: KKSQILYSISIASFNSSSFLIAFAMFGVYVMLDDRNVLDAQKVFVSMALINILKTPLSQLPFAISTTMQALVSLRRLGKYLCSEELRVDNVSKTLLDGEDVMIESGTFSWTPEGPPCLKR : 599
Dla.DLAgn_00177430: KKSQILYSISIASFNSSSFLIAFAMFGVYVTLDDRNVLDAQKVFVSMALINILKTPLSQLPFAISTTMQAMVSLRRLGKYLCSEELRVDNVSKAPLDGEDVAIENGTFSWSAEGPPCLKR : 599
Dre.ENSDARG00000016750: KKSQILYSVSLASFNSSSFLIAFAMFGVYVLIDDKNVLDAQKIFVSMALINILKTPLSQLPFAMSTTMQALVSLKRLGKFLCQDELKPDNVARESFDVDGVVFDNGTFSWSKDGPPCLKR : 598
Ame.ENSAMXG00000004837: RKSQILYSISIASFNSSTFLIAFAMFGVYVLIDDKNVLDAQKIFVSMALINILKTPLSQLPFAMSTTMQAVVSLKRLGKFLCQEEIKPDNVSRDPYDEDSVVVENGTFSWSKDGPPCLKR : 597
Ame.ENSAMXG00000003085: KKSQILYSISIASFNSSTFLIAFAMFGVYVLIDEKNVLDAQKVFVSMALINILKTPLSQLPFAMSTTMQAIVSLKRLGKFLCQDELKLDSVVRAPFDGDSVTIEDGTFSWSRDGPPCLRR : 600
Dre.ENSDARG00000094901: KKSQILYSVSIASFNSSTLLIAFAMFGVYVLIDDKHVLDAQKIFVSMALINILKAPLSQLPIAMSTTMQVVVSLKRLGTFLDQDELKLDSVQRVPYNIESVVINNGTFSWSKDSTPCLRR : 573
Dre.ENSDARG00000095820: KKSQILYSISIASFNSSTFLIAFAMFGVYVLIDDKHVLDAQKIFVSMALINILKAPLSQLPFAMSTTMQAVVSLKRLGKFLCQDELKLDSVERVPYYFESVVINNGTFSWSKDSTPCLRR : 600
Lch.ENSLACG00000022117: KKSQLLFAASLASFHSSAFLISFAVFAVYMLVDRSNVLDAQKAFVSMALVTIMKIPLSFLPFSISTTVQAGVSLKRLSTFLSHEELNPNNTDKSTTLGKQIVVENGTFSWSKDNPPCLTR : 600
Dpu.347281: KQAAYLSAGTSFLWTCAPFLVTLATFAVYVTTDPSHILDAKKAFVSLTLFNLLRFPMSMFPMLVVSFVQASVSIKRLNKFMNADELDPESVSHETT-ASAINIEKGSFAWSQGEQPILKD : 599
Lca.KE993868_1: ------------------------------------------------------------------------------------------------------------------------ : -
Pma.KM232930_1: GRAAYFSMLSAFTWLCSPFLVALASFGVYVLVDEHHVLDAERAFVSLALFNLLRYPLNQLPQLMSELAMASVSLRRLRNFLSLEELDPHAVDRSPSRESCVSIEGGTFTWSWDEPPILHD : 600
Csa.ENSCSAVG00000008135: RKAAYLNAASSFTFVCAPFLVSLTTFAVYVLSDEKNVLDAQKAFVSLSLFNILRFPLMMLPMVVTSLVQASVSLQRLESFLNNEELDQFNVDHSAASENAIRVDGASFRWDQDEEDVLQN : 591
Csa.ENSCSAVG00000003792: RTAAYLNAVGSFTWVSAPFLVSLTTFAVYVLSDSSHILTAQKAFVSLSLFNILRFPLVMLPAVIAAIVQASVSMTRLSKFLAGDELDPDAVDHSPTHGEVVEVDNATFTWGKKDEPILKN : 532
Cin.ENSCING00000020698: RTAAYLNAVGSFTWVCAPFLVSLTSFAVYVLSDKTHALTAQKAFVSLSLFNILRFPLVMLPAVIAAIVQASVSLKRLSNFLGGDELDPDAVDRSPNPGEVVEVENATFTWSKDEEPILKD : 143
Tca.TC012253: KEAAYMNAGTSFIWSCAPFLVSLVSFGTYVMVDEHNILDASKAFVSVSLFNILRFPLSMLPMMISNLVQAMVSIQRINKFMNAEELDPTSVTHDSNESAPLVIENGCFNWDEEQ--VLKN : 593
Aga.AGAP009835: KSAAYLNAGTSFIWSCAPFLVSLVTFATYVLVDENNVLDASTAFVSLSLFNILRFPLSMLPMLISNMVQTSVSVNRINTFLNQEELDPDNVQHDEKESSPLLIENGVFSWG-GEETTLKN : 593
Lgi.LotgiG107213: KKAAYLNAASSFTWTCAPVMVSLTTFAVYTLSSDENILDAEKAFVSLSLFNILRFPLSMLPQVITSIVQASVSLKRLDKFLGQPELDPVAVDRHSDKGQTISIENGTFTWETDLKPTLSE : 598
Bfl.90918: RKKGYFASLFFLTWSCAPVLVAIMTFTVYVMADERNVLDAEKAFVALSLFNIVRAPLNMLPSLVISIVQARVSLRRLGEFFGGDELDPENVHKETIPGRTIGVDDGTFSWGKEEDPILKN : 481
Dme.FBgn0032456: RSTAYLNAGTSFLWSCAPFLVSLVTFATYVLTSEANQLSVEKVLVSIALFDLMKLPLTILPMLSVDIAETQVSVNRINKFLNSEELDPNSVLHDSSKPHPMSIENGEFSWG--DEITLRN : 597
Oan.ENSOANG00000013379: ------------------------------------------------------------------------------------------------------------------------ : -
Pma.AKC42143_1: ------------------------------------------------------------------------------------------------------------------------ : -
Lca.KE994284_1: KKAAYLNAVSSFTWVCAPFLVALTTFGVYVMVDENNVLDAKKAFVSLTLFNILRFPLNMLPQVISSMVQVG------------------------------------------------- : 279
Cmi.SINCAMG00000015787: KNAAYFSAVSTFTWICAPFLVALSSFAVYVLVNEHNVLDAQKAFVSLALFNILRFPLNMLPVVISSLVQASVSLKRLRLFLSHEELDPGNVNRNDLSAYSISMKNATLSWSKDDSPCLKD : 599
Ler.ctg13956: KRAAYLAAVSTFTWVCAPFLVALCTFGVYVLVDEKNVLDAQKAFVSLALFNILRFPLNMLPMVISSLVQASVSLDRLETFLSHDQLDLDAVDRQFNSLNSIIVNNGTFSWSREDPPCLNN : 174
Rty.XP_020377569_1: --------------------------------------------------------------------QANVSLKRLGTFLSHEELDLDTVDKHSVSWNSIIVNDGTFRWSNEDPPCLNN : 52
Sca.ctg67278: KSAAYLSAVSTFTWVCAPFLVALSTFAVYVLVDENNVLDAQKAFVSLALFNILRFPLNMLPMVISSLVQAGVSLKRLDTFLSHEELDLDTVDRHLVSLNSIIVKNGTFSWSKDDAPCLNK : 500
Lch.ENSLACG00000001471: KKAAYLAVVATFTWVCTPFLVALSTFTVYVLVDENNVLDAQKAFVSLALFNILRFPLNMLPMVISSIVQASVSLKRLRVFLSHEELDPDSVDRSSSSDESITMRSGTFSWSNSDPPCLKG : 600
Tni.ENSTNIG00000005013: KKAGYLGAVTTFTWICAPFLVALSTFTVYVLMDENNVLDAQKAFVSLALFNILRFPLTMLPMVIK-ICRINWKLKTAMTLCSLQVI-------------CFVLIERLCTPCNG---CLDK : 583
Loc.ENSLOCG00000007196: KKSAYLAAVSTFTWVCTPFLVALSTFAVYVLVDERNVLDAQKAFVSLALFNILRFPLNMLPMLISSMVQASVSLKRLRVFLSHEELDEDGVNRKITSSDSISIVDGVFSWSKKDTPTLKR : 600
Dre.ENSDARG00000104719: KKMAYLGAISTFTWVCAPFLVALSTFAVYVLVDENNILDAQKAFVSLALFNILRFPLNMLPMVISSMVQASVSMQRLRVFLSHEELDDDNVERPAITPDSIRIADGAFSWSKDDPPTLKR : 600
Ame.ENSAMXG00000002943: KKAAYLGAISTFTWVCAPFLVALSTFAVYVLVDEHNVLDAQKAFVSLALFNILRFPLNMLPMVISSMVQASVSMKRLRVFLSHEELDENSVDRRAISADSIRIGDGAFSWSKEDRPALKR : 600
Xma.ENSXMAG7738/17319: KKTAYLGAISTFTWVCAPFLVALSTFAVYVLIDDQNVLDAQKAFVSLALFNILRFPLNMLPMVISSIVQV-------------------------------------------------- : 523
Ola.ENSORLG00000017141: KKAAYLGAVSTFTWVCAPFLVALSTFSVYVLIDDQNVLDAEKAFVSLALFNILRFPLNMLPMVISSMVQASVSLKRLRVFLSHEELQEDSVERPAASPYSISIEDGVFSWSRSESPTLKR : 600
Gac.ENSGACG00000000434: KKAAYLGAVSTFTWICAPFLVALSTFAVYVLIDEHNVLDAQKAFVSLALFNILRFPLNMLPMVISSMVQASVSLKRLRVFLSHAELQEDGVDHKAATSHSVSIVDGVFSWSRAESPTLKR : 600
Oni.ENSONIG00000007824: KKAAYLGAVSTFTWVCAPFLVALSTFAVYVLIDEQNVLDAQKAFVSLALFNILRFPLNMLPMVISSMVQASVSLKRLRVFLSHEELQVDSVEHKAASQYSISVTDGVFTWSRTESLSLFR : 600
Dla.DLA_00195360: KKAAYLGAMSTFTWVCAPFLVALSTFTVYVLIDEHNVLDAQKAFVSLALFNILRFPLNMLPMVISSMVQASVSLKRLRVFLSHEELQEDSVEHKAVSPHSISIVDGVFSWSRTESPILKK : 554
Xtr.ENSXETG00000019661: KKSAYLAAVGTFTWVCAPFLVALSTFAVYVLIDKQNVLDAEKAFVSLALFNILRFPLNMLPMVISSMVQASVSLKRLRVFLSHEELEPESIIREPQMHNGDSIKVDVFLRAS----TYHI : 596
Aca.ENSACAG00000005349: KKSAYLAAIGTFTWVCAPFLLAKCANCVFIYIDYKNVLDNPERWISETLLYSLK-SISHISLLKFKRQHASTSTESVSVYLISYDFEPLFLA----NGNSITVRNATFSWSRSDLPCLNN : 595
Gga.ENSGALG00000006646: KKSAYLAAMGTFTWVCAPFLVALSTFAVYVKVNKNNILDAQKAFVSLALFNILRFPLNILPMVISSIVEASVSLKRLRVFLSHEELDPDSIIRGPIAEGSIVVKNATFSWSKTDPPSLNS : 600
Dno.ENSDNOG00000014990: KKSAYLAAVGTFTWVCTPFLVALSTFAVYVTVDENNILDAQKAFVSLALFNILRFPLNILPMVISSIVQASVSLKRLRIFLSHEELEPDSIERRPVGTNSITVRNATFTWARDDSPTLSG : 600
Mmu.ENSMUSG00000023088: KKSAYLAAVGTFTWVCTPFLVALSTFAVFVTVDERNILDAKKAFVSLALFNILRFPLNILPMVISSIVQASVSLKRLRIFLSHEELEPDSIERRSIEGNSITVKNATFTWARGEPPTLNG : 600
Ggo.ENSGGOG00000004324: ----CVCDIITISHWAFCLQVALCTFAVYVTIDENNILDAQTAFVSLALFNILRFPLNILPMVISSIV---VSLKRLRIFLSHEELEPDSIERRPVGTNSITVRNATFTWARSDPPTLNG : 530
Hsa_ENSG00000103222: KKSAYLSAVGTFTWVCTPFLVALCTFAVYVTIDENNILDAQTAFVSLALFNILRFPLNILPMVISSIVQASVSLKRLRIFLSHEELEPDSIERRPVGTNSITVRNATFTWARSDPPTLNG : 600
Ptr.ENSPTRG00000007812: KKSAYLSAVGTFTWVCTPFLVALCTFAVYVTIDENNILDAQTAFVSLALFNILRFPLNILPMVISSIVQASVSLKRLRIFLSHEELEPDSIERRPVGTNSITVRNATFTWARSDPPTLNG : 554
Cluf.ENSCAFG00000018208: KKSAYLAAVGTFTWVCTPFLVALSTFAVYVTVDKNNILDAQKAFVSLALFNILRFPLNILPMVISSIVQASVSLKRLRIFLSHEELEPDSIERRPVGANSITVKNATFTWARSDPPTLSG : 600
Bta.ENSBTAG00000021090: KKSAYLAAVGTFTWVCTPFLVALSTFAVYVTVDENNILDAQKAFVSLALFNILRFPLNILPMVISSIVQASVSLKRLRVFLSHEDLDPDSIQRRPIATNSITVKNATFTWARNDPPTLHG : 600
Mdo.ENSMODG00000004194: KKSAYLAAVGTFTWVCTPFLVALSTFAVYVTVDKNNVLDAQKAFVSLALFNILRFPLNILPMVISSIVQASVSLKRLRIFLSHEELEPESIVRKPIGGDSIIVKNATFTWSRSDPPTLNG : 600
Oan.ENSOANG00000005124: KKSAYLAAVGTFTWVCTPFLVALSTFAVYMTIDENNILDAQKAFVSLALFNILRFPLNILPMVISSIVQASVSLKRLRIFLSHEELEPDSVVRCSVGGNSISVTNATFTWSRNDPPTLTG : 389
Bfl.232174: RNIAFVNALTTFTWVCAPVLVSLTTFAVYVMVDEKNILDAEKAFVALALFNIIRCPLSTLPNLITNLVQARVSLQRLENFLTHDELDPNNVDRHVLRGPPITIEDGTFSWGKTEDPILKD : 599
Bfl.118638: RNIAFVNALTTFTWVCAPVLVSLTTFAVYVMVDEKNILDAEKAFVALALFNIIRCPLSTLPNLITNLV---------------------------------------------------- : 548
Bfl.118636: RNTAFLNAGASFTWVCAPFLVSLTTFAVYVLVDERNILDAEKAFVALSLFNILRFPLNMLPNLITSMVQARVSLQRLENFLGHDELDPNNVDRHVARGPPIAIEDGTFSWGKTEDPILKD : 600
Bfl.128060: RNTAFLNAGASFTWVCAPFLVSLTTFAVYVLVDERNILDAEKAFVALSLFNILRFPLNMLPNLITSMVQARVSLQRLENFLGHDELDPNNVDRHVARGPPIAIEDGTFSWGKTEDPILKD : 600
Dre.ENSDARG00000096662: RKTAYLSALSTMAWTSAPFLVALTSFAVFVNVDEKNVLDAEKAFVSLSLFNILRFPLNMLPQVISSIVQASVSLKRLQDFLNHDELDPESVDRKSNTEYAVSVVNGKFSWAKRDQVILDN : 598
Ame.ENSAMXG00000016253: RKTAYLSALSTMAWTSAPFLVALTTFAVYVSVDKNNVLDADKAFVSLSLFNILRFPLNMLPQVISSLVQASVSLKRIQAFLSHDELDPDNVDKKPASDHAVTVVNGKFSWAKKDPPALQH : 599
Ola.ENSORLG00000020741: RKTAYLGALSTMAWTSAPFLVALTSFAVFVSVDENNVLDAKRAFVSLSLFNILRFPLNMLPQVISSIAQASVSLKRIQNFLSHDELDPDSVDRKNTGDFSVTVVNGTFTWAKEDPPVLHS : 600
Gmo.ENSGMOG00000010029: RRTAYLGALSTMAWTSAPFLVALTTFAVYVKVDENNVLDAEKAFVSLSLFNILRFPLNMLPQVISSIIQASVSLKRIQDFLSHSELDPEAVQKNSASEHSVTVINGKFTWTKQDPPALHN : 554
Xma.ENSXMAG00000012203: RKTAYLGALSTMAWTSAPFLVALTTFAVFVSVDEKNVLDAEKAFVSLSLFNILRFPLNMLPQVISSLVQASVSLKRIQNFLSHDELDPNSVDRKTSSEFAVSVVNGKFTWAKEDEPVLDN : 600
Gac.ENSGACG00000005901: RKMAYLGALSTMAWTSAPFLVALTTFAVYVSVDENNVLDAERAFVSLSLFNILRFPLNMLPQVISSLVQASVSLKRIQRFLSHDELDPDSVDRKNTTEFAVTVVNGKFTWSKEDPPILHN : 594
Oni.ENSONIG00000019586: RKTAYLGALSTMAWTSAPFLVALTTFAVYVTVDKKNILDAETAFVSISLFNILRFPLNMLPQVISSLVQASVSLKRVQNFLSHDELDPDSVNRNNTTEFAVTVVNGKFTWGKDDAPVLHN : 597
Dla.DLAgn_00098120: RKTAYLGALSTMAWTSAPFLVALTTFAVYVTVDENNILDAEKAFVSLSLFNILRFPLNMLPQVISSIVQASVSLKRIQNFLSHDELDPNSVDRKNITEFSLTVVNGKFSWAKEDPPVLHN : 600
Cmi.SINCAMG00000013336: KKAAYLNALSTFAWSSAPFLVALTTFSVYVTVDENNVLDAQKAFVSLALFNVLRFPLNMLPQVISSIVQASVSLKRLQKFLSHDELDPKSVNREKSSGYCISVLGGSFCWTRGDPAVLHN : 500
Ler.ctg12190: KKAACLNALSTFTWTTAPFIVAMTTFAVYVYVDENNVLDAQRAFVSLSLFNILRFPLNMLPQVISSVVQATVSLTRLQNFLSHDELDSTSVERQKTTGHAITVLNGTFSWGKMDPVVLER : 500
Sca.ctg14163: KKAAYLNALSTFTWTTAPFLVALTSFAVYVTVDENNVLDAQRAFVSLSLFNILRFPLNMLPQVISGVVQATVSLKRLQNFLSHDELDPTSIERQKTTGYAITMLNGTFSWGKTDPVVLNG : 450
Rty.XP_020375725_1: --------------------------------------------------------------------------------------------------HAITMLSGTFSWGKSDPVVLDG : 22
Sac_EU250283: KKAAYLSALSTFTWTTAPFIVALTTFAVYVTVDENNVLDAQKAFVSLSLFNILRFPLNMLPQVISSVVQATVSLNRLQKFLSHDELDPTSVDRQKTTGHAITVLNGTFSWGKSDPVVLDG : 600
Tni.ENSTNIG00000004171: RKMAYLGALSTMAWTSAPFLVALTTFAVYVRVDENNILDAEKAFVSLSLFNILRFPLNMLPQVISSMVQANVSLKRIQAFLSHDELDPNTIDRKNTQDYSITVVNGKFTWAKEDPPALHN : 572
Aca.ENSACAG00000001396: KKSAYLNSLSTFTWVSAPFLVALTTFAVYATVDENNILDAEKAFVSLSLFNLLRFPLNMLPQVISSIAQTSVSLKRIQHFLSHDELDPSCVDTKLIPGYSVTIRNGTFSWAKDLEPALKD : 597
Gga.ENSGALG00000007522: KKSAYLNSLSNFAWISSPFLVALTTFAVYVLVDEKNTLDAEKAFVSLSLFNILKFPLTMLPQVISNIAQTSVSLKRIQQFLSHDELDPNCVERKVIPGYAISVKNATFSWGKELKPSLKD : 548
Xtr.ENSXETG00000012239: KKAAYLNALSTFAWTSAPFLVALTTFAVYVTVDEKNILDAEKAFVSLSLFNILRFPLNMLPQVISNLAQASVSIKRIQNFLANDELDLNAVTKDKTPGNAITVHNGTFSWAKNGGAILQN : 600
Loc.ENSLOCG00000010918: RKTAYLGALSTFLWTSAPFLVALTTFAVYVTVDKDNVLDAQKAFVSLSLFNILRFPLNMLPQVISSVVQASVSLKRIQDFLSHDELDPDNVDRKNIAGFAVTVVNGKFTWSKQGPATLHN : 600
Lch.ENSLACG7209/6619: KKSAYLNAVSTFAWSSAPFLVVLDCVKFYEVVRENSTFNAGLCFFSIT-WNRIRFVLIALFMLVCNNFRFQV---RSSQAMFEGNLNSDGMDNKTHLGYAITVLNGKFSWVKSNPPILHD : 590
Mdo.ENSMODG00000020910: ------------------------------------------------------------------------------------------------------------------------ : -
Dno.ENSDNOG00000046300: HKITYLHACLNLSWICTPFLVTLITLGVYVSVNGSNVLDAEKVFVSLSLFHILKVPLGLLAQLANHLVQISVSLKQIQHFLSQDELDPECVERKAIPGCAIAIHSGTFTWARDLPPTLHS : 600
Mmu.ENSMUSG00000020865: RKGAYLQAISTFIWICTPFLVTLITLGVYVYVDESNVLDAEKAFVSLSLFNILKIPLNMLPQLISGLTQASVSLKRIQDFLNQNELDPQCVERKTIPGYAITIHNGTFTWAQDLPPTLHS : 600
Ggo.ENSGGOP00000003354: RTAAYLHTTTTFTWMCSPFLVTLITLWVYVYVDPNNVLDAEKAFVSVSLFNILRLPLNMLPQLISNLTQASVSLKRIQQFLSQEELDPQSVERKTIPGYAITIHSGTFTWAQDLPPTLHS : 600
Hsa.ENSG00000108846: RTAAYLHTTTTFTWMCSPFLVTLITLWVYVYVDPNNVLDAEKAFVSVSLFNILRLPLNMLPQLISNLTQASVSLKRIQQFLSQEELDPQSVERKTIPGYAITIHSGTFTWAQDLPPTLHS : 600
Ptr.ENSPTRG00000009406: RTAAYLHTTTTFTWMCSPFLVTLITLWVYVYVDPNNVLDAEKAFVSVSLFNILRLPLNMLPQLISNLTQASVSLKRIQQFLSQEELDSQSVERKTIPGYAITIHSGTFTWAQDLPPTLHS : 600
Cluf.ENSCAFG00000017201: RKSAYLQAISTFTWVCTPFLVTLTTLGVYVSVDQNNVLDAEKAFVSVSLFNLLKIPLNMLPQLISNLIQTSVSLKRIQHFLSQDELDLQCVERKTIPGYAVTIDNGTFTWAPDLPPTLHS : 600
Bta.ENSBTAG00000020070: RKVACLHAISTFIWVCTPFLVTLTTLGVYVSVDKNNVLDAEKAFVSVSLFNILKIPLNMLPQLISNLAQTSVSLKRIQHFLSQDELDPQCVERKTIPGYAVIIHNGTFTWAQDLPPALHS : 600

Hsa.CFTR.ENSG0000000162: INFKIERGQLLAVAGSTGAGKTSLLMVIMGELEPSEGKIKHSGRISFCSQFSWIMPGTIKENIIFGVSYDEYRYRSVIKACQLEEDISKFAEKDNIVLGEGGITLSGGQRARISLARAVY : 525
Hsa.ABCC5.ENSG000001147: IDLEIQEGKLVGICGSVGSGKTSLISAILGQMTLLEGSIAISGTFAYVAQQAWILNATLRDNILFGKEYDEERYNSVLNSCCLRPDLAILPSSDLTEIGERGANLSGGQRQRISLARALY : 615
Hsa.ABCC11.ENSG00000121: INLVVSKGMMLGVCGNTGSGKSSLLSAILEEMHLLEGSVGVQGSLAYVPQQAWIVSGNIRENILMGGAYDKARYLQVLHCCSLNRDLELLPFGDMTEIGERGLNLSGGQKQRISLARAVY : 602
Hsa.ABCC12.ENSG00000140: ISFVVRKGKILGICGNVGSGKSSLLAALLGQMQLQKGVVAVNGTLAYVSQQAWIFHGNVRENILFGEKYDHQRYQHTVRVCGLQKDLSNLPYGDLTEIGERGLNLSGGQRQRISLARAVY : 566
Hsa.ABCC8.ENSG000000060: ITIRIPRGQLTMIVGQVGCGKSSLLLAALGEMQKVSGAVFWRGPVAYASQKPWLLNATVEENIIFESPFNKQRYKMVIEACSLQPDIDILPHGDQTQIGERGINLSGGQRQRISVARALY : 713
Hsa.ABCC9.ENSG000000694: IDIRIPTGQLTMIVGQVGCGKSSLLLAILGEMQTLEGKVHWRYSVAYAAQKPWLLNATVEENITFGSPFNKQRYKAVTDACSLQPDIDLLPFGDQTEIGERGINLSGGQRQRICVARALY : 714
Hsa.ABCC10.ENSG00000124: SHLEVKKGMLVGIVGKVGCGKSSLLAAIAGELHRLRGHVAVRGGFGLATQEPWIQFATIRDNILFGKTFDAQLYKEVLEACALNDDLSILPAGDQTEVGEKGVTLSGGQRARIALARAVY : 705
Hsa.ABCC4.ENSG000001252: LSFTVRPGELLAVVGPVGAGKSSLLSAVLGELAPSHGLVSVHGRIAYVSQQPWVFSGTLRSNILFGKKYEKERYEKVIKACALKKDLQLLEDGDLTVIGDRGTTLSGGQKARVNLARAVY : 533
Cel.WBGene00003414: LSATIKPGQLIAIVGSVGGGKSSLLSAVLDEMVLLDGRVKVGGSIAYVPQHSWIFNKTIKENILFGNELSNYFYDQVVGSCQLKTDFRHFQQGENTMVGENGITLSGGQKARISLARAVY : 673
Cel.WBGene00003409: IEFLAGSKELVTVVGSVGSGKSSLLLAALGEMEKVCGYVGVRGSVAYLSQQPWILNQSLKKNVLMQADLNDVLYKKVIESCALKEDLKQLPDGDDTEIGEKGINLSGGQKARIALARAVY : 715
Hro.HelroG163344: INLKIRKKSLTAIVGQVGLGKSSLLSAALGDMEKRSGRVITNGRIGYVSQQAWIENATLRDNILFGKPFDEDKYNQIIEACALKQDLIVLAAGDMTEIGEKGINLSGGQKQRVSLARSIY : 676
Hro.HelroG157076: INLKVKKKSLTAIVGQVGIGKSSLLSAMLGDMEKKGGLVTVKGRIGYVSQQAWIENATLKANILFGKEFDEERYNKVIEACALRQDLEILPGGDMTEIGERGINLSGGQKQRVSMARSIY : 395
Bfl.230771: INFCIPDGALVAVIGQIGSGKSSLLSALLGEMENRTGDVSVKGSTAYVCQQPWIQNATLQDNILFDSPMDERWYSNVLDSCALRPDLEMLSGGDLTEIGEKGINLSGGQKQRVSLARAVY : 706
Lgi.LotgiG105097: INMKVKEGELLAVVGGVGAGKSSLLSAILGEMDKLQGQVNVKGSIAYVPQQAWIQNLSLRKNILFDGEVTKDYWQ-ILKDCALDSDLKVLPGGESIEIGERGINLSGGQKQRVSLSRSVY : 705
Cel.WBGene00003407: ITFNIKRGQLVAIVGRVGSGKSSLLHALLGEMNKLSGSVQVNGSVAYVPQLAWIQNLSLRNNILFNRPYDAKLYQNVIENCALVQDLESLPAEDRTEIGEKGINLSGGQKQRVSLARAVY : 715
Cel.WBGene00003408: ISFKIQKGQLVAIVGKVGSGKSSLLHALLGEMNKLSGSVQINGNIAYVPQQAWIQNMSLRNNILFNKPYDLENYEDVVKNCALKEDLANLPAGDRTEIGEKGINLSGGQKQRVSLARAVY : 714
Lgi.LotgiG153611: INIDISEGELVAVIGTVGSGKSSLLSAFLGEMERLAGKVATKGRVAYVSQQAWIQNNTARNNILFGAEMNKKQYKRVLKACALKEDLQILPGGEFTEIGEKGVNLSGGQKQRVNLARAVY : 667
Lgi.LotgiG110718: INIEIAEGKLVTVVGQVGCGKSSLISAILGEMDKLSGDVRVKGTIAYVPQQAWMKNDTVKGNILFGKPYNENKYTATLETCALTADLAILPGGDMTEIGEKGINLSGGQKQRVSLARALY : 529
Dno.ENSDNOG00000024923: ITLTVPQGCLLAVVGPVGAGKSSLLSALLGELSKVEGSVSIKGPVAYVPQEAWVQNASVLDNVCFGKELDWPWLQRVLQACALWPDVGGLPAGVHTQIGEQGMNLSGGQKQRLSLARAVY : 657
Mmu.ENSMUSG00000030834: INLTVPQGCLLAVVGPVGAGKSSLLSALLGELLKVEGSVSIEGSVAYVPQEAWVQNTSVVENVCFRQELDLPWLQKVLDACALGSDVASFPAGVHTPIGEQGMNLSGGQKQRLSLARAVY : 716
Cluf.ENSCAFG00000018197: INLTVPQGRLLAVVGAVGSGKSSLLSALLGELSKVEGSVSIKGSVAYVPQEAWVQNTSVVENVCFRQKLDPLWLETVLEACALWPDVSGFPAGVHTKIGEQGMNLSGGQKQRLSLARAVY : 716
Bta.ENSBTAG00000015191: INLTVPQGCLLAVVGPVGAGKSSLLSALLGELSKVEGSVSIKGPVAYVPQEAWVQNMSVVDNVCFGQELDAPWLETVLEACALWPDVDGFPAGVHTRTGEQGMNLSGGQKQRLSLARAVY : 716
Ggo.ENSGGOG00000009623: INLTVPQGCLLAVVGPVGAGKSSLLSALHGELSKVEGFVSIEGAVAYVPQEAWVQNTSVVENVCFGQELDPPWLERVLEACALQPDVDSFPEGVHTSIGEQGMNLSGGQKQRLSLARAVY : 707
Hsa.ENSG00000091262: INLTVPQGCLLAVVGPVGAGKSSLLSALLGELSKVEGFVSIEGAVAYVPQEAWVQNTSVVENVCFGQELDPPWLERVLEACALQPDVDSFPEGIHTSIGEQGMNLSGGQKQRLSLARAVY : 715
Ptr.ENSPTRG00000007815: INLTVPQGCLLAVVGPVGAGKSSLLSALLGELSKVEGFVSIEGAVAYVPQEAWVQNTSVVENVCFGQELDPPWLERVLEACALQPDVDSFPEGVHTSIGEQGMNLSGGQKQRLSLARAVY : 572
Oan.ENSOANG00000005123: ISLAVPRGHLLAVIGSVGAGKSSLLAALLGELSKLDGHVNVEGSVAYVPQEAWVQNASVEENVCFGQELEEPWFSRVLEACALQPDLASLPAGVHTEIGEQGINISGGQKQRVSLARAVY : 669
Mdo.ENSMODG00000005815: INLVVPRGSFFAVTGPVGSGKSSLLSAILGELTKLEGNVNIKGSVAYVPQEAWIQNASVEENVCFGQELNMPWLDRVLEACALPPDLASFPAGIHTEIGEQGINLSGGQKQRLSLARAVY : 720
Gga.ENSGALG00000006698: IDLTVPQGSLLAVVGQVGAGKSSLLSALLGDLEKMDGCVTMKGTAAYVPQQAWIQNASVEDNILFGKEMDETWFNRVVDACALQPDLESFPAGQKSEIGEKGINISGGQKQRVSLARAVY : 718
Aca.ENSACAG00000003478: INLSIARGSLCAVIGQVGSGKSSLLSALLGELQKTEGSLALKGTVAFVPQESWIQNASVEENITFGQKLDRNWFDRVVDACALQPDLDSFPHGSQAEIGEKGVNLSGGQKQRVSLARAVY : 658
Cel.WBGene00003413: VDLTAPRNSLIAVVGKVGSGKSSLLQALLGEMGKLRGRIGVNGRVAYVPQQPWIQNMTLRDNITFGRPFDRKRYDQVLYACALKADIKILPAGDQTEIGEKGINLSGGQKARVSLARAVY : 715
Aga.AGAP008437: INLALRRGKLSAVVGGVGTGKSSLISALLGEMEKMKGSVNTDGSIAYVPQQAWIQNATLRDNILFGRPFDQAKYDKVIECCALRPDLEMLPGGDTTEIGEKGINLSGGQKQRVALARAVY : 712
Aga.AGAP027980: INLSLRKGQLSAIVGTVGTGKSSLISALLGEMEKISGHVNTDGSIAYVPQQAWIQNATLRDNILFGKAFDQRKYDNVIECCALRPDLEMLPGGDSTEIGEKGINLSGGQKQRVALARAVY : 717
Aga.AGAP028128: INLSLRKGQLSAIVGTVGTGKSSLISALLGEMEKISGHVNTDGSIAYVPQQAWIQNATLRDNILFGKAFDQRKYDNVIECCALRPDLEMLPGGDSTEIGEKGINLSGGQKQRVALARAVY : 717
Spu.026395: INLDIKQGSLVAVVGQVGCGKSSLLSALLGEMEKVDGKVFVQGSVAYVPQQAWIQNATLRSNIVFSGDLHVTKYKHVIQSCALARDLVVLPGGDMTEIGEKGINLSGGQKQRVSLARAVY : 719
Hsa.ABCC2.ENSG000000238: VNLDIMAGQLVAVIGPVGSGKSSLISAMLGEMENVHGHITIKGTTAYVPQQSWIQNGTIKDNILFGTEFNEKRYQQVLEACALLPDLEMLPGGDLAEIGEKGINLSGGQKQRISLARATY : 709
Xtr.ENSXETG00000026360: INIGIPQGTLVAVVGQVGCGKTSLLSALLGEMEKVEGQVSLMGSVAYVPQQTWIPNATFKENVLFGRKMEKCWYDQVVQACALLPDLKILSGGENTEIGEKGVNLSGGQKQRISIARAVY : 704
Cel.WBGene00003410: ISFSVNRGQLVTIVGRVGAGKSSMLQALMGEMEKLSGSISMHGRLCYVPQQPWMQNNTLRQNITFGKQFDEYFYSRVLDACALYRDLQILPLGDNTEIGEKGINLSGGQKARISLARAVY : 716
Ame.GB53134: INLQVEQGQLVAVVGTVGSGKSSLLSALLGEMEKINGRVNTKGSIAYVSQQAWIQNASLQDNVLFGKSLHKNLYNRVIEACALTPDLKVLPAGDQTEIGEKGINLSGGQKQRVSLARAVY : 714
Pma.KM232931_1: IALRVPEGSLVAVVGDVGSGKSSLISAILGEIELVEGRVSVRGSVAYVPQQAWIQSGTVRSNVLFGRDMDEARYRHVLDTCALLPDLELLAGGDETEIGEKGINLSGGQRQRVSLARAAY : 436
Gac.ENSGACG00000003037: ISVKVKPGSLVAVVGHVGSGKSSLLSAMLGEMERRSGSISIKGSVAYVPQQAWIQNASLKDNILFGREKKESWYLRVLEACALLPDLEMLPAGDGTEIGEKGLNLSGGQRHRVSLARSVY : 720
Loc.ENSLOCG00000007152: INVRVPQGALLAVVGHVGSGKSSLLSAILGETEKRSGRVLVKGSVAYVPQQAWIQNATLRENVIFGREKKEAWYQRVVEACALLPDLEILPAGDATEIGEKGLNLSGGQKQRVSLARAVY : 720
Gmo.ENSGMOG00000005748: INVHVPRGSLVAVVGHVGSGKSSLLSAMLGETERRTGHVTVKGSLAYVPQQAWIQNATVQDNVVFGREKLKTWYQCVLEACALLPDLDILPAGDATEIGEKGLNLSGGQKQRVSLARAVY : 691
Gac.ENSGACG00000019172: ISIHVPRGSLVAVVGHVGSGKSSLLSAMLGETEKRSGCVTVKGSVAYVPQQAWIQNATVQDNILFGREKLKTWYHRVLEGCALLPDLEILPAGDATEIGEKGLNLSGGQKQRVSLARAVY : 720
Tni.ENSTNIG00000012067: INVHVPRGSLVAVVGPVGSGKSSLLSAMLGETEKRSGQVTVKGSVAYVPQQAWIQNATVQDNIVFGREKSKAWYQRVLEACALLPDLDILPAGDATEIGEKGLNLSGGQKQRVSLARAVY : 719
Ola.ENSORLG00000013429: ISVRVPRGSLVAVVGHVGSGKSSLLSAMLGETEKRSGQVTVKGSVAYVPQQAWIQNATVQDNILFGREKLKTWYQRVLEACALLPDLDILPAGDATEIGEKGLNLSGGQKQRVSLARAVY : 719
Oni.ENSONIG00000018866: ISVSVPRGSLVAVVGPVGSGKSSLLSAMLGETEKRSGQVTVKGSVAYVPQQAWIQNATVQDNIIFGREKLKTWYHRVLEACALLPDLDILPAGDATEIGEKGLNLSGGQKQRVSLARAVY : 719
Xma.ENSXMAG00000004906: INVRVPRGSLVAVVGHVGSGKSSLLSAMLGETEKRSGQVTVKGSVAYVPQQAWIQNATVQDNILFGRDKLKTWYQRVLEACALLPDLDILPAGDATEIGEKGLNLSGGQKQRVSLARAVY : 719
Dla.DLAgn_00177430: INIHVPRGSLVAVVGHVGSGKSSLLSAMLGETEKRTGRVSVKGSVAYVPQQAWIQNATVQDNIIFGREKMKTWYHRVLEACALLPDLDILPAGDATEIGEKGLNLSGGQKQRVSLARAVY : 719
Dre.ENSDARG00000016750: ISVKVPCGSLVAVVGHVGSGKSSLLSAMLGETEKRSGTVSVKGSIAYVPQQAWIQNASLQDNILFGREKKESWYQRVLEACALLPDLDNLPAGDATEIGEKGLNLSGGQKQRVSLARAVY : 718
Ame.ENSAMXG00000004837: ISVRVPCGGLVAVVGHVGSGKSSLLSAMLGETERRSGNVSIKGSVAYVPQQAWIQNATLQDNILFGREKKKTWYQRVLEACALLPDLEILPAGDATEIGEKGLNLSGGQKQRVSLARAVY : 717
Ame.ENSAMXG00000003085: ISVKVPRGCLVAVVGHVGSGKSSLLSAMLGETEKKSGNVTVKGSVAYVPQQAWIQNATLRENIVFGQEKKESWYQTVLEACALVRDLNILPARDATEIGEKGLNLSGGQKQRVSLARAVY : 720
Dre.ENSDARG00000094901: INVKVQRGSLVAVVGHVGSGKSSLLSAMLGEMEKKSGHITITGSVGYVPQQAWIQNATLKDNILFGCEKKDSLYQKVLEACALLPDLEILPARDATEIGEKGLNLSGGQKQRVSLARAVY : 693
Dre.ENSDARG00000095820: INVKVQRGSLVAVVGHVGSGKSSLLSAMLGEMEKKSGHIKITGSVAYVPQQAWIQNATLKDNILFGCEKKDSLYQKVLEACALLPDLEILPARDATEIGEKGLNLSGGQKQRVSLARAVY : 720
Lch.ENSLACG00000022117: INLTIPEGALVAVVGHVGCGKSSLLSALLGELEKQEGYVAIKGSVPYVPQQAWIQNATLKDNILFGQEMNESWYHRVIEACALLPDLEILPAGDGTEIGEKGVNLSGGQKQRVSLARAVC : 720
Dpu.347281: INIEIKPGKLVAVVGQVGAGKSSLISAILGEMEKLGGKANTNGKIAYIPQQAWIQNCSLRNNIMFGKTYNESVYNKVINACALKPDLAMLPGGDSTEIGEKGINLSGGQKQRVSLARSVY : 719
Lca.KE993868_1: ----------------------------------VTGVVHVQGQVAYVAQQAWIQNASLRDNVLFGRPLDEAWYREVLQACALEQDLLALPAGDNTEIGEKGINLSGGQRQRVSLARAIY : 191
Pma.KM232930_1: ISLAVPRGCLLAVVGTVGSGKSSLMAALLGELNKLGGSVAVKGQVAYVAQQAWIQNASLRDNVLFGRPLDETWYREVLQACALEQDLLALPAGDNTEIGEKGINLSGGQRQRVSLARAIY : 720
Csa.ENSCSAVG00000008135: ISMTVPEGSLVAIVGQVGCGKSSLVSAMLGDMEKVTGSVSVKGSVAYVPQQPWIQNLTVRDNITFGKDLNICKYQDTVEACELKSDFEMLPASDQTEIGERGINLSGGQKQRVAIARAVY : 711
Csa.ENSCSAVG00000003792: INLSVPSGSLVAVVGQVGTGKSSLISALLGDMDKVEGYVSVKGSIAYVAQQAWIQNLTVQDNILFGKPFDPCLYHQTVDACELKEDFDMLPAGDQTEIGERGINLSGGQKQRVSIARAVY : 652
Cin.ENSCING00000020698: INMSVPHGSLVAIVGQVGSGKSSLISALLGDMEKKAGSVAVKGSIAYVAQQAWIQNLTVQDNILFGKPLDACLYQQTIEACEL------------------------------------- : 226
Tca.TC012253: INIRIEKKSLCAVVGSVGSGKSSLLSAFLGEMDKTSGRVNTVGTIAYVSQQAWIQNATLRDNILFGKSFDKSLYDKVVEACALNPDFAMLPAGDQTEIGEKGINLSGGQKQRVSLARAVY : 713
Aga.AGAP009835: INVRVEKNQIVAVVGTVGSGKSSLLSAFLGEMDKISGRVNTLGRIAYVSQQAWIQNATLKDNILFGKPMDQRRYARVIEACALKPDIEMLPGGDMTEIGEKGINLSGGQKQRVSLARAVY : 713
Lgi.LotgiG107213: IDLEIKKGSLVAVVGAVGAGKSSFLSALLGEMEKTSGTVAVQGSVAYVQQQAWIQNETLQNNILFGKTLDKTVYDKVIYSCALQPDIEMLPGGDQTEIGEKGINLSGGQKQRVSVARALY : 718
Bfl.90918: INLTIPGGALVAVIGQVGSGKSSLLSALLGEMEKQGGRVAVLGSTAYVPQQAWIQNATLRDNILFGSPLNQSRYNEVLEACALGPDLEMLPAGDNTEIGEKGINLSGGQKQRVSLARAVY : 601
Dme.FBgn0032456: INIEVKKGSLVALVGTVGSGKSSVVQAFLGEMEKLAGVVNTVGKLAYVPQQAWIQNATVRDNILFGQTYDRKRYNKVIDACALRADIDILSAGDLTEIGEKGINLSGGQKQRISLARAVY : 717
Oan.ENSOANG00000013379: ------------------------------------------------------------------------------------------------------------------------ : -
Pma.AKC42143_1: ----------------------------------------------------------------------------------------------------------------AALARAVY : 265
Lca.KE994284_1: VSLSVPTGSLVAIVGHVGCGKSSLVSALLGELEKIQGMVAVK-SVALVSQQAWIQNATLRDNIIFGQPFEEAWYQEVLDACALGPDLGILPAGDQTEIGEKGINLSGGQKQRVSLARAVY : 398
Cmi.SINCAMG00000015787: INLAILEGSLVAVVGHVGCGKSSLISALLGEMEKQEGYVAVKGTVAYVSQQAWIQNTTLKDNIIFGQDWHKGWYNRVIRSCALLPDLEMLPAGDESEIGEKGVNLSGGQKQRVNLARAVY : 719
Ler.ctg13956: ISVEIPDGTLIAVVGHVGCGKSSLLSALLGEMEKMEGYVAVKESVAYVSQQAWIQNASLKRNILFGQELVEEWYKSVIESCALLPDLEVLPAGDETEIGEKGVNLSGGQKQRVSLARAVY : 294
Rty.XP_020377569_1: IDVEIPEGSLVAIVGHVGCGKSSLLSALLGEMQK-QGYVAVKGSVAYVPQQAWIQNASLRANIIFGQELMEEWYRKVVESCALLPDLENLPAGDETEIGEKGVNLSGGQKQRVSLARAVY : 171
Sca.ctg67278: IDVEIPEGSLVAVVGHVGCGKSSLLSALLGEMQKIEGYIAVKGSVAYVPQQSWIQNASLRANIIFGQELMEDWYMKVVESCALQPDLESLPAGDETEIGEKGVNLSGGQKQRVSLARAVY : 620
Lch.ENSLACG00000001471: INLTIPEGALVAIVGHVGCGKSSMLSALLGEMEKQEGHVAVKGSVAYVPQQAWIQNATLKDNILFGQEMNENWYRCVVEACALLPDLEILPAGDSTEIGEKGVNLSGGQKQRVSLARAVY : 720
Tni.ENSTNIG00000005013: LESVTNRGKFVGLI----------MCVIV------------QGLVAYVPQQAWIQNSTLKENIVFGQEFRESWYHSVIKVCNLI--LKVIFDKFNMQCHSQGVNLSGGQKQRVSLARAVY : 679
Loc.ENSLOCG00000007196: ISVRIPEGALVAVVGHVGSGKSSLLSALLGEMEKQEGQVSVKGSVAYVPQQAWIQNATLRENVIFGREKKEAWYQRVVEACALLPDLEILPAGDATEIGEKGVNLSGGQKLRVSLARAVY : 720
Dre.ENSDARG00000104719: INVSIPEGALVAVVGHVGSGKSSLLSALLGEMHKQEGSVSIKGSVAYVPQQAWIQNATLKDNILFGRETKDSWYQKVVEACALLPDLEILPGGDTTEIGEKGVNLSGGQKQRVSVARAVY : 720
Ame.ENSAMXG00000002943: INVRIPEGALVAVVGHVGSGKSSLLSALLGEMHKQEGDVSIKGSVAYVPQQAWIQNATLRENIMFGQEKKESWYQKVLEACALLPDLEILPGGDTTEIGEKGVNLSGGQKQRVSLARAVY : 720
Xma.ENSXMAG7738/17319: -------GALL--------GKSSLLSALLGEMDKVEGSVVVKGSVAYVPQQAWIQNSTLKENIVFGQKRREDWYNHVVEVCALQPDLEILPAGDETEIGEKGVNLSGGQKQRVSLARAVY : 628
Ola.ENSORLG00000017141: LNVRIPEGSLVAVVGHVGSGKSSLLSALLGEMDKMEGSVSVKGSVAYVPQQAWILNATLKNNIVFGQKRKEAWYHRVVEACALHQDLEILPAGDETEIGEKGVNLSGGQKQRVSLARAVY : 720
Gac.ENSGACG00000000434: LNVCIPEGSLVAVVGHVGSGKSSLLSALLGEMDKLEGSVAVKGSVAYVPQQAWIQNATLRENIMFGQESREAWYQRVVEACALQPDLEILPAGDETEIGEKGVNLSGGQKQRVSLARAVY : 720
Oni.ENSONIG00000007824: LNINIPEGSLVAVVGHVGSGKSSLLSALLGEMDKLEGSVTVKGSVAYVPQQAWIQNSSLKDNIIFGHERRQSWYQHVVEACALQPDLEILPAGDDTEIGEKGVNLSGGQKQRVSLARAVY : 720
Dla.DLA_00195360: LTVHIPEGSLVAVVGHVGSGKSSLLSALLGEMDKLEGTVTVKGWVAYVPQQAWIQNSTLKENIMFGQERRDSWYQCVVDACALRPDLEILPAGDETEIGEKGVNLSGGQKQRVSLARAVY : 674
Xtr.ENSXETG00000019661: INISIPEGSLVAVVGQVGCGKSSLLSALLGEMEKQDGYVAMKGSVGYVSQQAWIQNASLKDNVLFGRESNESMYKKVIEACALLPDLEILPTGDRTEIGEKGVNLSGGQKQRVSLARAVY : 716
Aca.ENSACAG00000005349: INFAVPEHRLVAVVGQVGCGKSSLLSALLGEMEKREGLVSLKGSVAYVPQQAWIQNATLKENILFGREARERQYNCVVEACALLPDLEVLPSGDQTEIGEKGVNLSGGQKQRVSLARAVY : 715
Gga.ENSGALG00000006646: INFTVPEGSLIAVVGQVGCGKSSLLSALLGEMDKKEGYVVVKGSIAYVPQQAWIQNATLEDNIIFGREMNESRYKRVIEACALLPDLEILPMGDRTEIGEKGVNLSGGQKQRVSLARAVY : 720
Dno.ENSDNOG00000014990: ITFSVPEGALVAVVGQVGCGKSSLLSALLAEMDKVEGHAALKGSVAYVPQQAWIQNDSLRENILFGRQLQERCYKAVIKACALLPDLEILPTGDRTEIGEKGVNLSGGQKQRVSLARAVY : 720
Mmu.ENSMUSG00000023088: ITFSIPEGALVAVVGQVGCGKSSLLSALLAEMDKVEGHVTLKGSVAYVPQQAWIQNDSLRENILFGHPLQENYYKAVMEACALLPDLEILPSGDRTEIGEKGVNLSGGQKQRVSLARAVY : 720
Ggo.ENSGGOG00000004324: ITFSIPEGALVAVVGQVGCGKSSLLSALLAEMDKVEGHVAIKGSVAYVPQQAWIQNDSLRENILFGCQLEEPYYRSVIQACALLPDLEILPSGDRTEIGEKGVNLSGGQKQRVSLARAVY : 650
Hsa_ENSG00000103222: ITFSIPEGALVAVVGQVGCGKSSLLSALLAEMDKVEGHVAIKGSVAYVPQQAWIQNDSLRENILFGCQLEEPYYRSVIQACALLPDLEILPSGDRTEIGEKGVNLSGGQKQRVSLARAVY : 720
Ptr.ENSPTRG00000007812: ITFSIPEGALVAVVGQVGCGKSSLLSALLAEMDKVEGHVAIKGSVAYVPQQAWIQNDSLRENILFGCQLEEPYYRSVIQACALLPDLEILPSGDRTEIGEKGVNLSGGQKQRVSLARAVY : 674
Cluf.ENSCAFG00000018208: ITFSIPEGSLVAVVGQVGCGKSSLLSALLAEMDKVEGHVAIKGSVAYVPQQAWIQNDSLRENILFGRQLQERYYKAVIEACALLPDLEILPSGDRTEIGEKGVNLSGGQKQRVSLARAVY : 720
Bta.ENSBTAG00000021090: ITFSVPEGSLVAVVGQVGCGKSSLLSALLAEMDKVEGHVTVKGSVAYVPQQAWIQNISLRENILFGRQLQERYYKAVVEACALLPDLEILPSGDRTEIGEKGVNLSGGQKQRVSLARAVY : 720
Mdo.ENSMODG00000004194: ITFTVPQGALVAVLGQVGCGKSSLLSALLAEMDKIEGHVSIKGSVAYVPQQAWIQNASLRENVLFGRQPQERFYKTVIESCALLPDLEILPSGDRTEIGEKGVNLSGGQKQRVSLARAVY : 720
Oan.ENSOANG00000005124: ITFAVPEGSLIAVVGQVGCGKSSLLSALLAEMDKVEGHVAIKGSIAYVPQQAWIQNASLRENILFGRQPEERHYKQVIEACALLPDLEILPSGDWTEIGEKGVNLSGGQKQRVSLARSVY : 509
Bfl.232174: INFTVPDGSLVAVVGQVGAGKSSLLSALLGEMEKQHGYVAVRGSTAYVPQQAWIQNATLRDNILFGKHMKCCQYKEVLEACALEQDLEMLPAGDLTEIGEKGINLSGGQKQRVSLARAVY : 719
Bfl.118638: -----------------------------------------QGSTAYVPQQAWIQNATLRDNILFGKHMKCCQYKEVLEACALEQDLGMLPAGDLTEIGEKGINLSGGQKQRVSLARAVY : 627
Bfl.118636: ISFAVPDGSLVAVVGQVGAGKSSLLSALLGEMEKQHGYVAVRGSTAFVPQQAWIQNATLRDNILFGKRLNNCQYKEVLEACALGPDLEMLPGGDMTEIGEKGINLSGGQKQRVSLARAVY : 720
Bfl.128060: ISFAVPDGSLVAVVGQVGAGKSSLLSALLGEMEKQHGYVAVRGSTAFVPQQAWIQNATLRDNILFGKRLNNCQYKEVLEACALGPDLEMLPGGDMTEIGEKGINLSGGQKQRVSLARAVY : 720
Dre.ENSDARG00000096662: INVLVPQGSLLAVVGHVGCGKTSLISALLGEMEKLDGQISIRGSVAYVPQQAWIQNATLRDNILFGRPYVEQKYRCVLEACALTPDLEVLPGGDLTEIGEKGINLSGGQRQRVSLARALY : 718
Ame.ENSAMXG00000016253: INVMVPQGSLLAVVGHVGCGKSSLVAALLGEMEKLEGHISIRGSVAYVPQQAWIQNATLRDNILFGRPYVEQKYRCVLEACALTPDLEVLPGGDLTEIGEKGINLSGGQRQRVSLARALY : 719
Ola.ENSORLG00000020741: VSVMVPRGSLLAVVGPVGCGKSSLISALLGEMEKLEGEVSIQGSVAYVPQQAWIQNATLRDNILFGNAYNEQKYCSVLDACALTQDLEVLPGGDQTEIGEKGINLSGGQRQRVSLARALY : 720
Gmo.ENSGMOG00000010029: INMLVPQGSLVAVVGQVGCGKSSLISALLGEMEKIEGDVAVRGSVAYVPQQAWIQNATLRDNILFGKPFAEQKYHSVLEACALTPDLEVLPGGDMTEIGEKGINLSGGQRQRVSLARSLY : 674
Xma.ENSXMAG00000012203: INVMVPQGSLVAVVGHVGCGKSSLISALLGDMEKVEGEVCVRGSVAYVPQQAWIQNATLRDNILFGKSFNESKYRRVLDACALTPDLEVLPGGDMTEIGEKGINLSGGQRQRVSLARALY : 720
Gac.ENSGACG00000005901: INVMVPQGSLLAVVGHVGCGKSSLISALLGEVEKLEGEVSIRGSVAYVPQQAWIQNASLRDNILFGKPYNEQKYCCVLEACALTPDLEVLPGGDMTEIGEKGINLSGGQRQRVSLARALY : 714
Oni.ENSONIG00000019586: INVMVPQGSLLAVVGHVGCGKSSLISALLGDMEKVEGEVSVRGSVAYVPQQAWIQNATLRDNILFGNPYNEQKYNSVLEACALTPDLQVLPGGDMTEIGEKGINLSGGQRQRVSLARALY : 717
Dla.DLAgn_00098120: INVMVPQGSLLAVVGHVGCGKSSLISALLGEMEKLEGEVSIRGSVAYVPQQAWIQNATLRDNILFGKPYNEQKYRCVLEACALTPDLEVLPGGDMTEIGEKGINLSGGQRQRVSLARALY : 720
Cmi.SINCAMG00000013336: IDLVAPEGALVAVVGHVGCGKSSLVSALLGEMERLEGSVSVQGSVAYVPQQAWVQNATLRENIVFGQPHIEHKYQLTLQACALLPDLTVLPGGDNTEIGEKGINLSGGQKQRVSLARALY : 620
Ler.ctg12190: INLIIPQGSLVAVVGHVGCGKSSLISALLGEMEKMEGKVAIQGSVAYVPQQAWIRNATLKDNIIFGEPISEQRYLQVLLGCALVPDLSMLPGGDQTEIGEKGINLSGGQKQRVSLARAVY : 620
Sca.ctg14163: ISMMVPQGSLVEVVGHVGCGKSSLVSALLGELEKLEGRVAIQGSVAYVPQQAWIRNASLKDNIIFEEPLSQHKYQQVLDACALITDLNVLPGGDQTEIGEKGINLSGGQKQRVSLARAVY : 570
Rty.XP_020375725_1: ISLMVPQGSLVAVVGHVGCGKSSLISALLGEMEKLEGRVAIHGSVAYVPQQAWIRNASLKDNIIFGELLSEQKYQQVLEACALITDLNVLPGGEQTEIGEKGINLSGGQKQRVSLARAVY : 142
Sac_EU250283: ISLTVPQGSLLAVVGHVGCGKSSLVSALLGEMEKLEGRVAIEGTVAYVPQQAWIRNASLKDNIVFGESLNEQKYQQVLEACALITDLNVLPGGDQTEIGEKGINLSGGQKQRVSLARAVY : 720
Tni.ENSTNIG00000004171: INLMVPQGSLLAVVGHVGCGKSSLISALLGEMEKLEGEVSIRGSVAYVPQQAWIQNATLRDNILFGEPYNEQKYCCVLEACALTADLEVLPGGDMTEIGEKGINLSGGQRQRVSLARALY : 692
Aca.ENSACAG00000001396: INWLVPNGSLVAVVGHVGCGKSSLVSALLGEMEKLHGEVAVKGSVAYVPQLAWIQNATLKDNILFGQPHNEQKYQMVLEACALKQDLEMLPGGDQTEIGEKGINLSGGQRQRVSLARAVF : 717
Gga.ENSGALG00000007522: INLLVPSGALVAVVGHVGCGKSSLVSALLGEMEKLEGEVAVKGSVAYVPQQAWIQNATLKDNILFGQAPNEQKYQNVLEACALKTDLEVLPGGDHTEIGEKGINLSGGQRQRVSLARAVF : 668
Xtr.ENSXETG00000012239: INLLVPSGSLVAVVGQVGCGKSSLVSALLGEMEKEEGEVSVRGSVAYVPQQAWIQNCTLKDNILFGRAANEKNYKKVLEACALVTDLEVLPGGDQTEIGEKGINLSGGQKQRVSLARAVF : 720
Loc.ENSLOCG00000010918: INLMVPQGSLLAVVGHVGCGKTSLISALLGEMEKLEGEISVRGSVAYVPQQAWIQNATLRNNILFGLPYNEQKYRSVLEACALVTDLEVLPGGDMTEIGEKGINLSGGQRQRVSLARALY : 720
Lch.ENSLACG7209/6619: INLMVPYGSLVAVVGHVGCGKSSLVSALLGEVEKLEGKVSIRGSVAYVPQQAWIQNATLKDNILFGQPFNEEKYQKVLEACALVTDLEMLPGGDQTEIGEKGINLSGGQKQRVSLARALY : 710
Mdo.ENSMODG00000020910: ------------------------------------------------------------------------------------------------------------------------ : -
Dno.ENSDNOG00000046300: LDIQVPGGALVAVVGPVGCGKSSLVSALQMEMEKLEGKVYVKGSVAYVPQLAWIQNATLQENVLFGKALDPKRYQRTLEACALLVDLDVLPSGDQTAIGEKDINLSGGQRQRVSLARAVY : 720
Mmu.ENSMUSG00000020865: LNIQIPKGALVAVVGPVGCGKSSLVSALLGEMEKLEGVVSVKGSVAYVPQQAWIQNCTLQENVLFGQPMNPKRYQQALETCALLADLDVLPGGDQTEIGEKGINLSGGQRQRVSLARAVY : 720
Ggo.ENSGGOP00000003354: LDIQVPKGALVAVVGPVGCGKSSLVSALLGEMEKLEGKVHMKGSVAYVPQQAWIQNCTLQENVLFGQALNPKRYQQTLEACALLSDLEMLPGGDQTEIGEKGINLSGGQRQRVSLARAVY : 720
Hsa.ENSG00000108846: LDIQVPKGALVAVVGPVGCGKSSLVSALLGEMEKLEGKVHMKGSVAYVPQQAWIQNCTLQENVLFGKALNPKRYQQTLEACALLADLEMLPGGDQTEIGEKGINLSGGQRQRVSLARAVY : 720
Ptr.ENSPTRG00000009406: LDIQVPKGALVAVVGPVGCGKSSLVSALLGEMEKLEGKVHMKGSVAYVPQQAWIQNCTLQENVLFGQALNPKRYQQTLEACALLADLEMLPGGDQTEIGEKGINLSGGQRQRVSLARAVY : 720
Cluf.ENSCAFG00000017201: LDIQVPKGALVAVVGPVGCGKSSLVSALLGEMEKLEGTVCVKGSVAYVPQGAWIQNCTLQENILFGQALDPKRYQQALKTCALLADLEMLPGGDQTEIGEKGINLSGGQRQRVSLARAVY : 720
Bta.ENSBTAG00000020070: LDIQVPKGALVAVVGPVGCGKSSLLSALLGDMEKLEGKVYMKGSVAYVPQQAWIQNCTLQENVLFGQALDPKRYHKALEACALLADLEVLPGGDQTEIGEKGINLSGGQRQRVSVARAVY : 720

Hsa.CFTR.ENSG0000000162: KDADLYLLDSPFGYLDVLTEKEIFESCVC--KLMATRILVTSKMEHLKKADKILILHEGSSYFYGTFSELQNLQPDFSSKLMGCDSFD-QFSATETKKQSFKQTGEFGEKRKNSILNPIN : 642
Hsa.ABCC5.ENSG000001147: SDRSIYILDDPLSALDAHVGNHIFNSAIR--KHLKTVLFVTHQLQYLVDCDEVIFMKEGCITERGTHEELMNLNGDYATIFNNLLLGETPPVE---INSKKETSGSQKKSQDKGPK---- : 726
Hsa.ABCC11.ENSG00000121: SDRQIYLLDDPLSAVDAHVGKHIFEECIK--KTLRTVVLVTHQLQYLEFCGQIILLENGKICENGTHSELMQKKGKYAQLIQKMH------------KEATSDMLQDTAKIAEKPKVE-S : 707
Hsa.ABCC12.ENSG00000140: SDRQLYLLDDPLSAVDAHVGKHVFEECIK--KTLRTVVLVTHQLQFLESCDEVILLEDGEICEKGTHKELMEERGRYAKLIHNLRGLQFKDPEVEAFKESPAEREEDAGIIVLAPGNEKD : 684
Hsa.ABCC8.ENSG000000060: QHANVVFLDDPFSALDIHLSDHLMQAGIL--ELLRTVVLVTHKLQYLPHADWIIAMKDGTIQREGTLKDFQRSECQLFEHWKTLMNRQ-DQEL--ETVTERKATEPPQGLSRAMSSRDGL : 828
Hsa.ABCC9.ENSG000000694: QNTNIVFLDDPFSALDIHLSDHLM-----QEGILKTLVLVTHKLQYLTHADWIIAMKDGSVLREGTLKDIQTKDVELYEHWKTLMNRQDQELEMEADQTTLERKTLRRAMYSREAKA--- : 826
Hsa.ABCC10.ENSG00000124: QEKELYLLDDPLAAVDADVANHLLHRCIL--GMLSTRLLCTHRTEYLERADAVLLMEAGRLIRAGPPSEILPLVQAVPKAWAENGQESD----SATAQSVQNPEKTKEGLEEEQSTS--- : 816
Hsa.ABCC4.ENSG000001252: QDADIYLLDDPLSAVDAEVSRHLFELCIC--QILHITILVTHQLQYLKAASQILILKDGKMVQKGTYTEFLKSGIDFGSLLK-------KDNEESEQPPVPGTPTLRNRTFSESSVW--- : 641
Cel.WBGene00003414: QDKDIYLLDDPLSAVDAHVGRALFDKVIGPDGLLRTRVLVTHNLQYTKYVDTIYVIEDGQIVQHGSFEDIAYVDGPFGRLWSECENSDEDVADAESSEASVTPPVPVLENGDNGAIEKSS : 793
Cel.WBGene00003409: QSKDVYFLDDPLSAVDAHVGKHIFDNVIGPNGMLSTRILVTNCTSFLQESGKIIVMKDGRIKHCGTYNELLTDVEA-----REYLQEVDNEYAQAQESSGEESGGEENSDILPGSIASGS : 830
Hro.HelroG163344: QDCDVYLFDDPLSAVDSHVAKHIFDSVIGPHGILKTRVLVTHGITWLPSCDVVVVMAGGIISECGSYQDLLNHNLVFAQFIRDFLQEEEEEEEKSLLERKKEVKKLLRERVNSLISNKED : 796
Hro.HelroG157076: QDCDVYLLDDPLSAVDAHVARHIFTNVIGPRGMLRTRVLVTHGIAWLPSCDVVVVMAGGLISECGSYRDLLSHNLVFAKFIRDFLQKHENDDDEEDGALRKSTDGMNEKLVKQFHHR--- : 512
Bfl.230771: SGADVYYLDDPLSAVDAHVGRHIFNHIIGPNGLLKTRLLVTHGTSFLSQCDQVIVLQDGRIWLMGDYHSLMEQSQEFAQYIRTYTNIV-----EGQSDSAGDNTGYINGLRKRLQKPPCD : 821
Lgi.LotgiG105097: SNKDIYLLDDPLSAVDTHVGKHIFKHVIGKQSILKTRILVTHGVHWLPLVDRIIVLDQGQITEVGSYEELLNHSGPFAQFVKTYTISEDKEEEGSITSIHNLGELVEEEFIEEGKVGYFK : 825
Cel.WBGene00003407: QNAEIVLLDDPLSAVDSHVGKHIFENVISATGCLGTRVLLTHGLTYLKHCDQVIVLKDETISEMGTYQELMNSNGAFSEFLEEFLLEESKHKGDQVSPAIRQRIQSQMSQEIEKTDDKNA : 835
Cel.WBGene00003408: QNPDIILLDDPLSAVDSHVGKHIFENVISSTGCLATRVLVTHGLTYLKHCDQLIVLKEGTISELGTYQELLNNSGAFAEFLEEFLIEESKTRGGEVDEILRDLGQVKPGILKRLESHLSQ : 834
Lgi.LotgiG153611: SDSDIYLLDDPLSAVDSHVGKHIFKRVISDNGILKTRVLVTHAVHWLPLVDTVVLMDNGRIIDCGHYLKLMRNNGALAEFLHTHMSKS-----DKAQEATEDAEESSLDSKKKSDFR--S : 780
Lgi.LotgiG110718: SDADIYLFDDPLSAVDSHVGKHIFKKMIGPFGCLKTRLLVTHGVHWLPMVDNILVMNDGRITERGTYAELIKGNAAFAQFLKTYLIEGVGIESTSDGATSGDENPVTERKRYENSIL--T : 647
Dno.ENSDNOG00000024923: RKAAVYLLDDPLAALDAHVGQQVFSRVIGPDGLLQTRILVTHARHVLPQADRIAVLADGAIAEMGSYQELLHRKGALADLLGRARQPG-ERGEAEPAAGAEDPRGSAGGGRPEREPE--R : 774
Mmu.ENSMUSG00000030834: KKAAIYLLDDPLAALDAHVSQQVFKQVIGPSGLLQTRILVTHTLHVLPQADRILVLANGTIAEMGSYQDLLQRNGALVGLLDGARQPAGTHDA----ATSDDLGGFPGGGRPTCRPDRPR : 832
Cluf.ENSCAFG00000018197: SKAAVYLLDDPLVALDAHVGQSVFNQVIGPGGLLHTRILVTHALHVLPQADWIVVLEDGAIAEMGRYQELLHRKGALVGLLDAARQPG-DRGDTELMTNAEDPRGPAGSEQPVGGPE--R : 833
Bta.ENSBTAG00000015191: RKAAVYLLDDPLAALDAQVGQHVFNRVIGPDGLLQTRILVTHALHILPQADWIVVLEDGAIAEMGSFQELLHRKGALVGLLDGASQPGDGGEGTEPPAGAKDPRGSAAGGRPEGRSE--R : 834
Ggo.ENSGGOG00000009623: RKAAVYLLDDPLAALDAHVGQHVFNQVIGPGGLLRTRILVTHALHILPQADWIIVLANGAIAEMGSYQELLQRKGALMCLLDQARQPGDIGEGTEPGTSTKDPRGTSAGRRPELRRE--R : 825
Hsa.ENSG00000091262: RKAAVYLLDDPLAALDAHVGQHVFNQVIGPGGLLQTRILVTHALHILPQADWIIVLANGAIAEMGSYQELLQRKGALMCLLDQARQPGDRGEGTEPGTSTKDPRGTSAGRRPELRRE--R : 833
Ptr.ENSPTRG00000007815: RKAAVYLLDDPLAALDAHVGQHVFNQVIGPGGLLQTRILVTHALHILPQADWIIVLANGAIAEMGSYQELLQRKGALMCLLDQARQPGDTGEGTEPGTSTKDPRGTSAGRRPELRRE--R : 690
Oan.ENSOANG00000005123: RRASVYLLDDPLSAVDAHVGQHIFDHIIGPDGLLKTRILVTHAVSVLPRVDSIVMLVDGAIAEIGSYRELVRRKGAFVDFLCQSGQTE-DAAGTALSAAAGTSRISLASKNRKFPGEVDR : 788
Mdo.ENSMODG00000005815: KKAAIYLLDDPLAALDAHVGQHIFDRVIGPGGLLHTRILVTHAVHILPQADYIIMMADGAVVESGSYQELLQRNGPFTDFLGQSKQEEANASQ--EVKSSRNISESEASTNKTDSFI--- : 835
Gga.ENSGALG00000006698: QRSSIYLLDDPLSAVDAHVGQHIFEHVLGPNGLLKTRVLVTHMISVLHQVDTIVVLVDGTIAEIGSYQELSQRSGAFAEFLQSHNTAEEKACSFPATGDIRDTITSRNNPPEDNLFS--D : 836
Aca.ENSACAG00000003478: TKAEVYLLDDPLSAVDAQVGQHIFKHVLGPTGLLKTRLLVTNAVHLLPRMDRIIVVMNGEISETGSWQELVARNGAFADFLRSHGTEGGKDQDNNIHNTAQNQKSMQKGMTLSDFIP--K : 776
Cel.WBGene00003413: QNLDVYLLDDPLSAVDAHVGRHIFEKVIGPNGLLRTRILVTHGLTYTKMADEILVMLEGKIEESGTFEHLIKRRGLFFDFMEEYKSGS-DNSSAIGGEIQDYMNPEDVVLTVTNDLD--E : 832
Aga.AGAP008437: ADSEVYLFDDPLSAVDAHVGKHIFEKVIGPSGMLVSRLLVTHGISFLPFVEEIFVMKDGEVSESGSYQELLDQKGAFAEFLTQHIQEM-DDEDEDELKLIQEALKDGEAKKIVQRAMSTR : 831
Aga.AGAP027980: ADAEVYLFDDPLSAVDAHVGKHIFEKVIGPSGMLVSRLLVTHGISYLPFVENIFVVKDGEISESGSYQQLLDQKGAFAEFLTQHIQELDEADE-DEIKLIQETLKDETAQRIVERSLSVR : 836
Aga.AGAP028128: ADAEVYLFDDPLSAVDAHVGKHIFEKVIGPSGMLVSRLLVTHGISFLPFVENILVLKDGEISESGTYQELIDQKGAFAEFLSQHIQELDDEDE--ALSVRSNRSNGSDGSTRKKPIS--R : 833
Spu.026395: ANTDLYLLDDPLSAVDAHVAKHIFGHVIGPQGLLKTRILVTHGISFLPQVDQIIVMIDGSVSEIGSYQDLLDQNGAFAEFLRNYSQDADEKED--------------------------- : 812
Hsa.ABCC2.ENSG000000238: QNLDIYLLDDPLSAVDAHVGKHIFNKVLGPNGLLKTRLLVTHSMHFLPQVDEIVVLGNGTIVEKGSYSALLAKKGEFAKNLKTFLRHTGPEEEGSEEEDDDYGLISSVEEIPEDAASITM : 829
Xtr.ENSXETG00000026360: RNCDVYLLDDPLSAVDAHVGQHLFEQVIGPSGLLKTRVLVTHGVSFLPQMDMIIVMSDGRVSEVGTYNELLQKNGAFSEFLNTYARKSVVFEEYEEQISAETPNSIQGAMKMKGKHS--- : 821
Cel.WBGene00003410: QNHDIYLLDDPMSAVDAHVGSQLFGSVIGPEGMLRTRILVTNELSFLEKSDLIIVMNEGKIEYSGKYDDLMQQ-GAFEQLLIECEKEE-RERRPGGIMIGGDSDFEYDDDVMASPII--D : 832
Ame.GB53134: NDSDIYFLDDPLSAVDSHVGKHIFENVIGSSGLLKTRILVTHGITYLPEVDNIIVLKDGEITEVGTYKQLLEKRGAFSEFLVQHLQEVGNLHAELQQKLTRGKSRMSESQSESGSIADRK : 834
Pma.KM232931_1: QSASLLLLDDPLSAVDAHVGSSLFRRVVAHGGLLHTRVLVTHAVGVLPSVDLIVVLSGGRVTECGSHSELLARDGAFAEFLRRHGQE------PTAAPDCEHMLLMIILVMLMMMMMFTL : 550
Gac.ENSGACG00000003037: RRSDVYLLDDPLSAVDAHVGQHIFDRVIGPRGLLKTRVLVTHGLSFLSKTDLILVMLEGHISEMGSYKDLMDRKGNFAKFIHAFNGKRRRGSSSRGSSASRDKSKTCVGSRKSASRLSEL : 840
Loc.ENSLOCG00000007152: RKADVYLLDDPLSAVDAHVGQHIFDKVIGPKGLLRTRVLVTHGLSFLPQADLILVLSDGEITEMGSYLELLGRNGAFADFIRVFASSD-----RKESAVHRGPRKSSSRLSVTDYMPVSR : 835
Gmo.ENSGMOG00000005748: RRADVYLLDDPLSAVDAHVGQHIFDKVIGPKGVLRTRVLVTHGMSFLPQADLILVMVDGEITESGSYQELLSRQGAFAEFIHTFANTE-----------RKESAIQRGEMVDFMPCS--R : 798
Gac.ENSGACG00000019172: RKADVYLMDDPLSAVDAHVGQHIFDKVIGPKGVLRTRILVTHGMSFLPQADFILVLGDGEITESGSYQELLSRHGAFADFIHTFANAERKESAQRGEKHSVKRRPCKSNMVDFMPSSRDL : 840
Tni.ENSTNIG00000012067: RKADVYLLDDPLSAVDAHVGQHIFDKVIGPKGVLRTRILVTHGMSFLPQADQILVLVDGEITESGSYQELLSRHG-FADFIHTFARTERKESA-IQRGAAHWVGQPGRDFKAVESVLGIP : 837
Ola.ENSORLG00000013429: RKADLYLLDDPLSAVDAHVGQHIFDKVIGPKGVLKTRILVTHGMSFLPQADLILVLIDGEITESGSYQELLSHHGAFADFIHTFASTEKKESAIQRVFCVAGSRRSNARLSMVDFMPFSR : 839
Oni.ENSONIG00000018866: RKADVYLLDDPLSAVDAHVGQHIFDKVIGPKGVLRTRILVTHGMSFLPQADLILVLVDGEITESGSYQELLSRHGAFADFIHTFASTERKESAKETFQTLAGSRRSNARLSMVDFMPFSR : 839
Xma.ENSXMAG00000004906: RKADVYLLDDPLSAVDAHVGQHIFDRVIGPKGVLRTRILVTHGMSFLPQADLILVLADGEITESGSYQELLSRHGAFADFIHTFASTERKESV----IQRAGSRRSNARLSMVDFMPFSR : 835
Dla.DLAgn_00177430: RKADVYLLDDPLSAVDAHVGQHIFDKVIGPKGVLRTRILVTHGMSFLPQADHILVLVDGEITESGSYQELLSRHGAFADFIHTFASTERKESA----IQRAGSRRSNARLSMVDFMPFSR : 835
Dre.ENSDARG00000016750: RKGDVYLLDDPLSAVDAHVGQHIFNKVIGPKGILRTRVLVTHGMSFLPQADLILVLVDGEISERGSYQELLNRNGAFADFIHTFANSE-RKECFSEALQRGSRKSVRLSVTDYMPFS--R : 835
Ame.ENSAMXG00000004837: RKADIYLLDDPLSAVDAHVGQHIFDKVIGPKGVLRTRVLVTHGTSFLPQADLILVLVDGEITESGSYQELLNRNGAFADFIRTFASSE-----RKESSVQRGSRRSCARLSVTDYMLFSR : 832
Ame.ENSAMXG00000003085: RNADIYLLDDPLSAVDAQVGQHIFDRVIGPKGILKTRVLVTHGLNFLPQADLILVMGDGEITETGSYIELLNRRNAFADFVQTFAGNERKEISKGKQGFPLTENKDSLGNLHSTCNESLT : 840
Dre.ENSDARG00000094901: RNSDIYLLDDPLSAVDAHVGQHIFEKVIGPNGSLKTRVLVTHGLSFLPQADLILVMADGEIKEMGSYAELLSRKNAFAE-LKAFSVSERKMHLTRKSVSFLSIKDFSTDLIRGDLGS--- : 809
Dre.ENSDARG00000095820: RKADIYLLDDPLSAVDAHVGQHIFEKVIGPNGILKTRVLVTHGLSFLPKADLILVIVDGEITEMGSYVELLSRKNAFAEFVKAFSVSERKESAKGKIKFTLTTVKIHVNLGQTSLLTSLK : 840
Lch.ENSLACG00000022117: RRSAVYLLDDPLSAVDARVGQSIFEKVIGPNGLLKTRVLVTHAVSVLPQADSIIVMSNGGISEMGSYKELLERGGAFAELLRTYTNAEQSESTGLREKSCSVTIETLGSSSMTEDIPAQK : 840
Dpu.347281: SDMDVYLLDDPLSAVDSHVGKHIFDEVIGPKGLLKTRLLVTHGITFLPQVDQIIVLKNGEVSEVGSYKELLAQKGAFAEFLLQHLEEEGADEDPDELAEIKQELENTMGKEEFARQISRQ : 839
Lca.KE993868_1: SKASVFLLDDPLSAVDAHVGKHIFERVIGPSGLLATRVLVTHGLSFLPHVDRIVVLTAGRVSESGSYSELMLSGEAFSELLRNHGQSPASQDD--------------------------- : 284
Pma.KM232930_1: SKASVFLLDDPLSAVDAHVGKHIFERVIGPSGLLATRVLVTHGLSFLPHVDRIVVLTAGRVSESGSYSELMLSGEAFSELLRIHGQGDGAGEDDLRSPGPDEVLSTCSNIADMEPVMTEP : 840
Csa.ENSCSAVG00000008135: QDADVYLFDDPLSAVDSHVGKNIFDNVLGPKGCLKTRILVTHGLAFLPQVDKIFVLVNGRITEVGDYYELIEKNGAFAEFLRNYAVMMSIASDSISDQEVEDLDLNEIPAEDARKKFVRW : 831
Csa.ENSCSAVG00000003792: QDADVYLFDDPLSAVDAHVGKNIFDNVLGPRGCLKTRLLVTHGVSFLPQVDRIVVLVGGRVSEIGHYDELLEKNGAFAEFLRNYANHEDSESTLEPDVDADETASSDVIARRNRTIQFKR : 772
Cin.ENSCING00000020698: ------------------------------------------------------------------------------------------------------------------------ : -
Tca.TC012253: ANSDIYFLDDPLSAVDSHVGKHIFDKVIGPEGLLRTRVLVTHGITYLPQTDKIIVLKDGEVSEGGTYQELLDKKGAFSEFLLQHINEVEEDEEEINKKLSRHRSRVSESVSETGSDQTSL : 833
Aga.AGAP009835: NDADVYFLDDPLSAVDSHVGKHIFEQVIGPSGLLATRVLVTHGITYLPNTDKIFVLREGEISESGTYQELMDKKGAFAEFLIQHLQEV-SEEELDEIKQQLENSVGGEELLNQLKRS--N : 830
Lgi.LotgiG107213: QNTDIYLLDDPLSAVDSHVGKHIFDNVIGPNGLLNTRVLVTHGISYLPKVDKIVVLVDGRISEAGSFQELLDHNGAFSSFLKNYLLEEVGGDRSRNTFYVLIYRSYENLALKTQLSE--- : 835
Bfl.90918: SDSSVYYLDDPLSAVDTHVGKHIFNKVIGPNGLLKTRLLMTHGISFLPQCDQIVALVDGRIWLMGTYRQLMAQNEAFADFIRNYGNLEEERDD--------DMEDNSKGRQNGQPMA--- : 710
Dme.FBgn0032456: SDADLYLLDDPLSAVDAHVGKHIFEEVIGPKGILASRVLVTHGVTFLPQVDSIYVIKMGEISESGTFDQLVKNKGAFADFIIQHLQEG-NEEETADVPELLGTVEKAIKLARTESLS--D : 834
Oan.ENSOANG00000013379: ------------------------------------------------------------------------------------------------------------------------ : -
Pma.AKC42143_1: SKVSVYLLDDPLSAVDAHVGRHIFDKVIGPSGILKTRVLVTHGLSFLPQVNDIVVLVGGSVSERGSYQQLQQRNGAFAEFLRSYAQAE-------------------------------- : 353
Lca.KE994284_1: SKVSVYLLDDPLSAVDAHVGRHIFDKVIGPSGILKTRVLVTHGLSFLPQVNDIVVLVGGSVSERGSYQQLQQRNGAFAEFLRSYAQAE-------------------------------- : 486
Cmi.SINCAMG00000015787: NDCSVYFLDDPLSAVDAHVGRHIFEKVIGPKGLLKTRILVTHGINFLPQMDVIFVMVDGKISEFGTYQELLEQGGAFSEYLQAYAHKETSEPEDAEDDGPNRPRHKKRRLSTISSASEMQ : 839
Ler.ctg13956: SNNAVYLMDDPLSAVDAHVGRHIFDKVIGPKGLLNTRVLVTHGISFLPQMDRILVMVDGKISESGTYKELLEQKGAFSEFLRMHAPTENDNAPAIQKLNDTGLQASLYNILTGSKSN--I : 412
Rty.XP_020377569_1: SRNSVYFLDDPLSAVDAHVGRHIFDKVIGPNGLLSTRVLVTHGVNFLPQMDRILVMVDGKISESGTYKELLKQNGAFAEFLRTYAPRENEVTELNHISPHKSPQNFHQSLIKINSISHDE : 291
Sca.ctg67278: SDNSVYFLDDPLSAVDAHVGRHIFDKVIGPKGLLKTRVLVTHGVSFLAQMDRILVMVDGKISESGTYKELLKQNGAFAEFLRTYAPTDNEETEQKHIHPRRTSRNSPLSSASYDEEAMPS : 740
Lch.ENSLACG00000001471: CNRAVYLMDDPLSAVDAHVGKHIFEKVIGPKGLLKTRVLVTHGVSYLPQMDLVVVMVDGQISEIGSYQELLKQEGAFAEFLHTYANKEQNAENPVNSPTFKEGPLLENGIVPLQKQLPRQ : 840
Tni.ENSTNIG00000005013: CDRAVYLLDDPLSAVDAHVGRHIFDQVVGPQGLLKTRLLVTHGLSFLSQTNLILVMSRGKLY---RWAPTTTAGGTHSQFLWNFLVHLEPLKISNKIVALSNVKIIVKVWEENVRNRSTR : 796
Loc.ENSLOCG00000007196: CDCAVYLLDDPLSAVDAHVGKHIFEKVIGPRGVLRTRVLVTHGLSFLPQADLILVMVDGEITEVGSYSELMDRQGAFSEFLRTYANADQGEEEDKPEDRSSSPGREQKGLENGGPAALRQ : 840
Dre.ENSDARG00000104719: CNCSVYLLDDPLSAVDAHVGKHIFEKVIGPQGLLQTRVLVTHGLSFLPQADLILVMVDGEITEMGSYTELLGRQGAFAEFLRTYTNTEQEEGE-----ESLGDAVPRKGLENGGPAALLR : 835
Ame.ENSAMXG00000002943: CDCSVYLLDDPLSAVDAHVGKHIFEKVIGPQGVLQTRVLVTHGLSFLPQADLILVMVDGEITETGSYAELLNRQGAFADFLRTYANAEQDGEP-----DGMTDGAPRKTLENGGPAAVLR : 835
Xma.ENSXMAG7738/17319: CDRAVYLLDDPLSAVDAHVGKHIFDYVIGPQGILRTRVLVTHGLSFLPQTDLILVMVDGEITEAGSYQQLMVQEGAFAEFLRTYATVD-QTDDQHVPVASSRSCFFTLQNLKTAPKSGSK : 747
Ola.ENSORLG00000017141: CDRSVYLLDDPLSAVDAHVGKHIFDHVIGPQGLLKTRILVTHGLSYLPQAHLVLVMVDGEITEVGSYQQLKEKEGAFAEFLRMYAANE-QSEEEKSLSSCLEPVPNSPTKPMENGVG--P : 837
Gac.ENSGACG00000000434: CERDVYLLDDPLSAVDAHVGKHIFEQVVGPQGLLKTRVLVTHGLSYLPQADLILVLVEGEVSEMGSYQHLMATEGAFAEFQRTYAAVEHADHDENVTRVRKSLTHFTRGRLLGQLDNASL : 840
Oni.ENSONIG00000007824: CDRAVYLLDDPLSAVDAHVGKHIFDQVIGPQGLLKTRVLVTHGLSYLPQADLILVMMKGEISEVGSYQQLMATEGAFAEFLRTYAAVDKTDNSDVLSSSSEPVPNSSIQRLENGSVSTPA : 840
Dla.DLA_00195360: CDRAVYLLDDPLSAVDAHVGKHIFDQVIGPQGLLKTRVLVTHGLSYLPQADLILVMVEGQITEMGSYQQLMATEGAFSEFQRTYSAVDHTDNN--------------------------- : 767
Xtr.ENSXETG00000019661: CNTDIYLLDDPLSAVDAHVGKHIFDKVIGPKGMLKTRILVTHGVSYLPQMDSIIVMVDGKITEVGSYQDLLMQDGAFAEFLRTYANAEQNKDQEAPSPVPSEEKRLENGILRNERNL--- : 833
Aca.ENSACAG00000005349: SDADIYLMDDPLSAVDAHVGRHIFEKVIGPKGILKTRILVTHGVSYLPVVDTIIVLSEGKVSEMGSYQELLQRDGAFAEFLRTFASAEQTRESGANSPAAKEEKHLENGILANDGPGNPL : 835
Gga.ENSGALG00000006646: CNADTYLFDDPLSAVDAHVGKHIFEKVIGPKGILKTRVLVTHAVNYLPQMDTILVMTDGEISEMGSYQELLKQDGAFAEFLRTYANAEQSMESDASSPSGKEGKPVENGVLVNDAPG--K : 838
Dno.ENSDNOG00000014990: CNADIYLLDDPLSAVDAHVGKHIFENVVGPKGLLKTRILVTHSVNYLPQVDVIVVMSGGKISEMGSYQELLARDGAFAEFLRTYAGAEQEQAATGVSGPAKEAKQMENGMLVMDAAG--K : 838
Mmu.ENSMUSG00000023088: SNSDIYLFDDPLSAVDAHVGKHIFEKVVGPMGLLKTRILVTHGISYLPQVDVIIVMSGGKISEMGSYQELLDRDGAFAEFLRTYANAEQDLASDSVSGSGKESKPVENGMLVTDTVG--K : 838
Ggo.ENSGGOG00000004324: SNADIYLFDDPLSAVDAHVGKHIFENVIGPKGMLKTRILVTQSMSYLPQVDVIIVMSGGKISEMGSYQELLARDGAFAELLRTYASTEQEQDATGVSGPGKEAKQMENGMLVTDSAG--K : 768
Hsa_ENSG00000103222: SNADIYLFDDPLSAVDAHVGKHIFENVIGPKGMLKTRILVTHSMSYLPQVDVIIVMSGGKISEMGSYQELLARDGAFAEFLRTYASTEQEQDATGVSGPGKEAKQMENGMLVTDSAG--K : 838
Ptr.ENSPTRG00000007812: SNADIYLFDDPLSAVDAHVGKHIFENVIGPKGMLKTRILVTHGMSYLPQVDVIIVMSGGKISEMGSYQELLARDGAFAEFLRTYASTEQEQDATGVSGPGKEAKQMENGMLVTDSAG--K : 792
Cluf.ENSCAFG00000018208: CDSDIYLFDDPLSAVDAHVGKHIFENVIGPKGMLKTRLLVTHSISYLPQVDVIIVMTGGKISEMGSYQELLARDGAFAEFLRTYASGDQEQAETGVSSPGKEVKQMENGMLVTDVAG--K : 838
Bta.ENSBTAG00000021090: CDSDVYLLDDPLSAVDAHVGKHIFENVIGPKGLLKTRLLVTHAISYLPQMDVIIVMSGGKISEMGSYQELLARDGAFAEFLRTYASAEQEQGQAGVGGPGKEVKQMENGMLVTDTAG--K : 838
Mdo.ENSMODG00000004194: CDSDVYLFDDPLSAVDAHVGKHIFEKVIGPQGILKTRILVTHSISYLSQVDVIIVMSDGKISEMGSHQELLDRDGAFAEFLRTYANAEQNMEDGTNGPVVKEVKQMENGVLISETAG--K : 838
Oan.ENSOANG00000005124: CDADVYLFDDPLSAVDAHVGKHIFEKVIGPKGLLRTRILVTHGISYLPQVDKIIVMSEGKISEMGSHQELLERDGAFAEFLRTYANAEQSPDDGSNSPAVKEVKPMENGVLVMEGSA--K : 627
Bfl.232174: SDSNIYYLDDPLSAVDAHVGKHIFDHVIGPNGILKTRLMVTHGISFLPQCDQIMVLVDGRIWLLGTYTELMEQNEAFAEFIRNYGNVDENKKARGMLSSYKGSQRILRKGSPDSRIGKLD : 839
Bfl.118638: SDSNIYYLDDPLSAVDAHVGKHIFDHVIGPNGILK--------------CDQIMVLVDGRIWLLGTYTELMEQNEAFAEFIRNYGNVDENKKA---DPTDKGSQRILRKGSPDSHIGKLD : 730
Bfl.118636: SDSNIYYLDDPLSAVDAHVGKHIFDNVIGPNGILK--------------CDQIMVLVDGKIWLLGTYTELMEQNEAFAEFIHNYGNFEDEEENDIGVLVDDDPSLPAQNAEEDNPITQAR : 826
Bfl.128060: SDSNIYYLDDPLSAVDAHVGKHIFDNVIGPNGILK--------------CDQIMVLVDGKIWLLGTYTELMEQNEAFAEFIHNYGNFEDEEENDIGVLVDDDPSLPAQNAEEDNPITQAR : 826
Dre.ENSDARG00000096662: SEADVYLLDDPLSAVDAHVAKHIFDHVIGPEGALKTRILVTHGISFLPQVNNILVLVDGRVSEMGSYQDLLRQNGAFAEFLRNYSLEDIIEDDEEEEEFPEDALSNHTDMVDNEPVV--N : 836
Ame.ENSAMXG00000016253: SEADVYLMDDPLSAVDAHVAKHIFDHVIGPEGALRTRILVTHGISFLPQVDNILVLVEGIVSEMGSYQDLLKQNGAFAEFLRNYSLEDIIEEDDEEESFPDDALSNHTDMVDNEPAVNEA : 839
Ola.ENSORLG00000020741: SDADVYLLDDPLSAVDAHVAKHIFDRLIGPDGLLKTRILVTHGISFLPQVDNIMVLGAGRVSEMGSHQELLKQNGAFAEFLRNYALED-ILEELEEELGNHHCDMMENEPVMNEARK--A : 837
Gmo.ENSGMOG00000010029: SDADVYLLDDPLSAVDAHVAKHIFDNLIGPEGALKTRLLVTHGVSFLPQVDNILVMVDGRVTEMGSYQELLDQNGAFAEFLRNYAIEDVIEEEEDEELFPDDALSNHTDMADNEPVVNET : 794
Xma.ENSXMAG00000012203: SDSEVYLLDDPLSAVDAHVSKHIFDNLIGPEGVLKTRILVTHGISFLPQVDNIVVMVEGRVSEMGSYQELLNQNGAFAEFLRNYALEDIVEED--EATGTVNRSNHHTDMVDNEPTVNEA : 838
Gac.ENSGACG00000005901: SDTDIYLLDDPLSAVDAHVAKHIFDNLIGPEGVLKTRILVTHGISFLPQVDNIMVIVEGRVSEMGSYQELLKQNGAFAEFLRTYALEDIVEDEEEHQKRPLAYKGPTDGALEDKCLS--T : 832
Oni.ENSONIG00000019586: NDADVYLLDDPLSAVDAHVSKHIFDNLIGPEGALKTRILVTHGISFLPQVDNIMVMVDGRVSEMGSYQDLLKQNGAFAEFLRNYALEDIIEEDEDDELFPDDALSNHTDMVDSEPMI--N : 835
Dla.DLAgn_00098120: SDTDVYLLDDPLSAVDAHVAKHIFDNLIGPEGALKTRILVTHGISFLPQVDNIMVMVDGRVSEMGSYQELLNQNGAFAEFLRNYALEDIIEEDEDEELFPDDALSNHIDMVDNEPGINEA : 840
Cmi.SINCAMG00000013336: SDSDVYLLDDPLSAVDAHVAKHIFDQVIGPEGALKTRVLVTHGVSFLPQVDHIVVLVDGRVSEQGSYQQLLERQGAFAEFLHTYAQSEEPNEEEEEEFLPEELLGNHDDLNDEQPMSADA : 740
Ler.ctg12190: SDMDIYLLDDPLSAVDAHVAKHIFDKVIGPEGSLKTRVLVTHGISFLPQVDQIVVLVNGKVTELGSYQELQEQNGAFAEFLRTYAQWD-EVEEGEEEFLAEDSMSNHIDLSDHELSATEA : 739
Sca.ctg14163: SDTDVYLLDDPLSAVDAHVAKHIFDKVIGPDGMLKTRVLVTHGISFLPQTDQIVVLVNGKVSEMGSYQELQNQNGAFAEFLRNYAQRDEVDED------EPTVLDEEEEFQKNEPRE--- : 681
Rty.XP_020375725_1: SDMDVYLLDDPLSAVDAHVAKHIFDKVIGPEGVLKTRVLVTHGISFLPQVDQIVVLVNGRVSEMGSYQELQEQNGAFAEFLRNYAQRD-EIEEEEEEFLGEDTLSNHIDLSDNEPSA--A : 259
Sac_EU250283: SDTDVYLLDDPLSAVDAHVAKHIFDKVIGPEGALKTRVLVTHGVSFLPQVDQIVVFVNGKVSEMGSYQELQAQNGAFAEFLRNYAQRDDVEEDEDEEFLGEDALSNHVDLSDNEPSAAEA : 840
Tni.ENSTNIG00000004171: SDADVYLLDDPLSAVDAHVSKHIFDNLIGPEGVLK-----------------------GKVSEMGSYQELLNQNGAFAEFLRNYSLEDIIEEEEDEKLFLDDALSNHTDMVDNEPAINEE : 789
Aca.ENSACAG00000001396: SDTDVYLLDDPLSAVDSHVAKHIFDKVIGPEGALRTRILVTHGINFLPQVDHIVVVVDGMISEMGSYQELLQQNRSFAEFLRNYAPDE-DIEEDEEVLLAEDTLSNHIDLADSEPVT--N : 834
Gga.ENSGALG00000007522: SSSDIYLLDDPLSAVDSHVAKHIFDQVIGPDGVLKTRILVTHGIGFLPQVDHIVVLADGKISEMGSYQELLKQNKAFAEFLRNYALDE-NTEEEEEVLLAEDTLSIHTDLADNEPVT--N : 785
Xtr.ENSXETG00000012239: SNADVYLLDDPLSAVDAHVAKHIFDNVIGPDGLLRTRVLVTHGISFLPQVDHIVVLVDGRVTEMGSYQELLKQNGAFSEFLRNYAFDD-EVEEEEEVLLAEETLSTHTDLADNEPVA--N : 837
Loc.ENSLOCG00000010918: SEADLYLLDDPLSAVDAHVAKHIFDKVIGPEGALQTRILVTHGISFLPQVDNIVVIVEGKVSEMGSHQELLKQNRAFAEFLRNYTMED-FIEEEDEETFPDDALSNHIDMVDNEPVV--N : 837
Lch.ENSLACG7209/6619: SESEVFLLDDPLSAVDSHVAKHIFDNVIGPGGALNTRILVTHGISFLPQVNQIVVLVDGKVTEMGSYQDLLKQNGAFAEFLRNYAPDE-DIEE-----PTEETLSNHVDLTDNEPIA--N : 822
Mdo.ENSMODG00000020910: ------------------------------------------------------------------------------------------------------------------------ : -
Dno.ENSDNOG00000046300: SDADVFFLDDPLSTVDSHVAKHIFDQVIGPEGMLATRVLVTHSISFLPQTNLVIVLADGQVAKVGPYQDLLQCNSSLANFIHNCVPNEDEEHPLEDLEDEEDTLSRHPNLIDSAPVT--W : 838
Mmu.ENSMUSG00000020865: SDANIFLLDDPLSAVDSHVAKHIFDQVIGPEGVLATRVLVTHGISFLPQTDFIIVLAGGQVSEMGHYSALLQHDGSFANFLRNYAPDE-DQEDNEEVLLLEDTLSTHTDLTDNEPAI--Y : 837
Ggo.ENSGGOP00000003354: SDADIFLLDDPLSAVDSHVAKHIFDHVIGPEGVLATRVLVTHGISFLPQTDSIIVLADGQVSEMGPYPALLQRNGSFANFLCNYAPDEDQGHLDKEALLIEDTLSNHTDLTDNDPVTYVV : 840
Hsa.ENSG00000108846: SDADIFLLDDPLSAVDSHVAKHIFDHVIGPEGVLATRVLVTHGISFLPQTDFIIVLADGQVSEMGPYPALLQRNGSFANFLCNYAPDEDQGHLDKEALLIEDTLSNHTDLTDNDPVTYVV : 840
Ptr.ENSPTRG00000009406: SDADIFLLDDPLSAVDSHVAKHIFDHVIGPEGVLATRVLVTHGISFLPQTDFIIVLADGQVSEMGPYPALLQRNGSFANFLCNYAPDEDQGHLDKEALLIEDTLSNHTDLTDNDPVTYVV : 840
Cluf.ENSCAFG00000017201: SEADLFLLDDPLSAVDSHVAKHIFDQVIGPEGVLATRVLVTHSISFLPQMDFIIVLADGQVSEVGSYPALLQRNGSFANFLSNYAPDENEENMDQEVMLIEDTLSNHTDLTDNEPVMYEV : 840
Bta.ENSBTAG00000020070: SDADIFLLDDPLSAVDSHVAKHIFDQVIGPEGVLATRVLVTHGISFLPQTDFVIVLSDGHVSEMGTYSALLQRDGSFANFLRNYAPDEDKEHQDEEVLMIEDTLSNHTDLTDNEPVTYEV : 840


Hsa.CFTR.ENSG0000000162: SIESIPAVTTWNTYLRYITVHKSLIFVLICLVIFLAEVAASLVVLWLTPLQDKNSTRNNAVIYYVFYIYVGVADTLAMGFRGLPLVHTLITVSKILHHKMLHSVLQAPMSTLNTLKAGGI : 762
Hsa.ABCC5.ENSG000001147: --EKGQGSVPWSVYGVYIQAAGGPLAFLVMALFMLNVGSTAFSTWWLSYWIKQDSMNPHMQYYASIYALSMAV--MLILAIRGVVVKGTLRASSRLHDELFRRILRSPMKFFDTTPTGRI : 842
Hsa.ABCC11.ENSG00000121: QAEMEEGSLSWRVYHHYIQAAGGYMVSCIIFFFVVLIVFTIFSFWWLSYWLEQGNINPQLSFYQLVYGLNALL--LICVVCSSGITKVTRKASTALHNKLFNKVFRCPMSFFDTIPIGRL : 825
Hsa.ABCC12.ENSG00000140: EGSPQEGTVTWKTYHTYIKASGGYLLSLFVFLFLLMIGSAAFSNWWLGLWLDKGAVDIGQHVYQWVYTASMVF--MLVFVTKGFVTKTTLMASSSLHDTVFDKILKSPMSFFDTTPTGRL : 802
Hsa.ABCC8.ENSG000000060: LQLHQRAEIPWRACAKYLSSAGILLLSLLVFSQLLKHMVLVAIDYWLAKWTDSECTQTV---YAMVFTVLCSL-GVLCLVTSVTVEWTGLKVAKRLHRSLLNRIILAPMRFFETTPLGSI : 944
Hsa.ABCC9.ENSG000000694: QMRLRT-KMPWKTCWRYLTSGGFFLLILMIFSKLLKHSVIVAIDYWLATWTSETGKQTY---YVAGFSILCGA-GFLCLVTSLTVEWMGLTAAKNLHHNLLNKIILGPIRFFDTTPLGLI : 941
Hsa.ABCC10.ENSG00000124: --SKKEGAVALHVYQAYWKAVGQGLALAILFSLLLMQATRNAADWWLSHWISQNGS-SDIRFYLTVYATIAGVNSLCTLLRAVLFAAGTLQAAATLHRRLLHRVLMAPVTFFNATPTGRI : 933
Hsa.ABCC4.ENSG000001252: --NRSEGKVGFQAYKNYFRAGAHWIVFIFILLNTAAQVAYVLQDWWLSYWANKGGGTEKLNWYLGIYSGLTVA--TVLFARSLLVFYVLVNSSQTLHNKMFESILKAPVLFFDRNPIGRI : 757
Cel.WBGene00003414: QINVQLGRVKKSVYQLYIKTMGIFNSSAFLIFFIAHFTVMIMRSLWLSDWSNESTSDGPVETRLIVYAGFGGLE-MLLLALAFTVTIGSLRASYGLHSPLIHALLVAPISFFDTTPTGRI : 912
Cel.WBGene00003409: RMEAAIGRVNPGVYLLYFKAMGIVTYVLPYAIAVVLNVSALGRSLWLTAWSDANHP-DTVGARLGVYAGFGITEVIFLFFSLVLLLIGGVAASKNLHKPLLHNVLRNPLSYFDITPIGRI : 949
Hro.HelroG163344: EDISETGSVKLSVYWAYIKSLGVLLSSIFIFFFILFNVAALLSNFWLSKWTNDNASNGSEDYYLGGYAGFGLLQAIFILIYALVSAETNVKSSRVLHQDLLYNILRSPMTFFDTTPIGRI : 916
Hro.HelroG157076: --VAETGQVKWPVYWTYIRALGKRFIIF-LIIFIAARTLPLLTNVWLSMWTSDNVTTNQDVYYLWGYTALGLGQAVLILMYAMLSAVVNARAARVLHDGLLKNIL----RWFTHSFIADD : 625
Bfl.230771: GVEEQSTGVKLSVLGGYIRSFGIGMFTC-MFLLCCGQRANHYSIILLSDWTSGNDSQHTVHLRLGGYGALGLAEGLCSMLIHLCVVEGAYLASKRVHDKALLHLLRGALQFFDVTPLGRI : 940
Lgi.LotgiG105097: FARPWTQKIRFKVIKSYLRHFGIRISLVTLMLFLLAQCSAVGSNIWLGIWTESN--QQG---NLSLYCCMNIFSALASLGYIAVLFYNMVKVSGIMFAYLLGNILRQPMNFFDTTPSGRI : 940
Cel.WBGene00003407: EIAVETGKVKFEVYMSYFRAIGIKIALVFFLVYVASSMLGVFSNLYLARWSDDSGNSSETQIRLGIYAVLGMGQATSVCAASIIMALGMVCASRLLHATLLENIMRSPMAFFDVTPLGRI : 955
Cel.WBGene00003408: ESTVETGKVKFEVYIAYFQAISIPITLLFFFLYVGSSGLGILSNFYLAKLSDHSGNSSDAKMELGIYAVLGMGQSFVVLIASIILTIGVLRASRILHAGLLGNIMRSPMAFFDVTPIGRI : 954
Lgi.LotgiG153611: RLNAETGKVEFSVFSTYAKAVGVFSTIFIFVSYTIYQVGSVAANIWLSVWTEDKNTSQSKEYYLAIYGVFGLVQAVFILLYAGLSTTKMVKAAKTMHFNMLDKIIRAPMIFFETTPIGRI : 900
Lgi.LotgiG110718: ILSTGRGKIPFKVFLYYFKAAGSIGFAAAIFFFVLFQAASIGSNYWLTFWTDDNNAQQTPNFFLTVYGLFGVFQVVLVLVHSLLYWTRLNKAAQVMHSKLMDSIFRAPMSFLDTTPIGRI : 767
Dno.ENSDNOG00000024923: SVSMPYGRVKASMYLSYLRAVGAPLCLYALFLFLAQQVASFCRGYWLSLWADDGGRQAHAALRGWVFGLLGCLQAVGLFASMAVVLLGGARASSLLFRRLLWDVARSPVGFFEQTPVGNL : 894
Mmu.ENSMUSG00000030834: PTSVRYGRVKTTIYLSYLRAVGTPLCTYTLFLFLCQQVASFSQGYWLSLWADDDGRQMHAALRGWVFGLLGCLQAIGLFASMAAVFLGGARASGLLFRSLLWDVARSPIGFFERTPVGNL : 952
Cluf.ENSCAFG00000018197: SVGTQNGRVKATMYLSYFQAVGVPLCVYALFLFLCQQVASFCHGYWLSLWADDDGRQTQAALRGSIFGILGCLQAVGLFASMAMVLLGGIRASSLLFQRLLWDVMRSPIGFFERTPIGNL : 953
Bta.ENSBTAG00000015191: FMGTQYGRVKATMYLTYLRAVGTPLCLYALFLFLCQQVASFCRGYWLSLWADDDGQQTHVALRGWVFGLLGCLQAIGLFASMATVLLGGIRASSLLFRGLLWDVARSPIGFFERTPVGNL : 954
Ggo.ENSGGOG00000009623: SISIQYGRVKATVHLAYLHAVGTPLCLYALFLFLCQQVASFCRGYWLSLWADDGGQQTQAALRGGIFGLLGCLQAIGLFASMAAVLLGGVRASRLLFQRLLWDVVRSPISFFERTPIGNL : 945
Hsa.ENSG00000091262: SISIQYGRVKATVHLAYLRAVGTPLCLYALFLFLCQQVASFCRGYWLSLWADDGGQQTQAALRGGIFGLLGCLQAIGLFASMAAVLLGGARASRLLFQRLLWDVVRSPISFFERTPIGHL : 953
Ptr.ENSPTRG00000007815: SISIQYGRVKATVHLAYLRAVGTPLCLYALFLFLCQQVASFCRGYWLSLWADDGGQQTQAALRGGIFGLLGCLQAIGLFASMAAVLLGGVRASRLLFQRLLWDVVRSPISFFERTPIGNL : 810
Oan.ENSOANG00000005123: GRRVQTGRVNLALYLTYMRAAGTPGCLITLLFFLCQQVASFSSSYWLSLWVDDDGVQQHTRLRLGVFGALGFLQAIGKFGSIAAVLLGGVCASHRLFRELLRSVSRCPMGFFEKTPVGNL : 908
Mdo.ENSMODG00000005815: --RVHYGRVNATLYLAYLRAVGMPICLSVVFLFLCQQMISSSRGYWLSLWADDNGTQQHTGLRVGVFGLLGCLQAIGRFGSIAVVLLGGVQASQWLFQGLLREVSRSPMTFFEQTPIGNL : 953
Gga.ENSGALG00000006698: NSNTQQGRVNAPVYAAYLRATGLPLCAYIILLFTCQQGVSFFRGYWLSVWTEDNGTQQYTELRVGVFGALGVIQAVVRFVSTAAVFLGGVLASHKLFLQLLWNVARSPTVFFEETPIGNL : 956
Aca.ENSACAG00000003478: QQGPITGRAKTSIYLSYLRVAGSLAWAYIVLLFTCQQVASFCRGYWLSLWANDNGTQPHTELRVGVFFFLGFAQALGKFASMATVFLAGTVASHRLFRQLLWDVVRSPMGFFEQTPSGHL : 896
Cel.WBGene00003413: TIGIAQGKVEIATYQLYVKAAGYLLSIAFIGFFIVYMTLQILRSFWLSAWSDEPSAPMAKGWRLGVYGALGFSETACFFVALLALVFVGQRASKNLHGPLIHNLMRSPMSFYDTTPLGRI : 952
Aga.AGAP008437: SQESATGAVGYVVYIKYFKGIGLWLGFWSIFFSVINQGASIYANIWLTDWSEDAATDPSRDMYLGVYGGLGGAQSIALLIASVTLALGCIKAARELHNNLLESSMRMPMSFFDTTPLGRI : 951
Aga.AGAP027980: SGESATGAVTWAVYKKYVTAIGFQFGFWSVVFSAINQGSGIYSSMWLTDWSEDTDTRDM---YLGVYGALGGVQSIALFIGSVLLALGCLKAAEESHNKLLESSMHMPMSFFDTTPLGRI : 953
Aga.AGAP028128: QVESATGAVTWLVYKKYIQSIGFKFGFGSVLFTAINQGSGIFSNLWLTDWSEDTDPRDK---YLGVYGALGGAQSIALFVAALLISLGCLKAAKESHNKLLESCLRMPMSFFDTTPLGRI : 950
Spu.026395: ---------------------------------------------------------------------------------------------------------------RNLEPLRGK : 821
Hsa.ABCC2.ENSG000000238: RRFIETGKVKFSIYLEYLQAIGLFSIFFIILAFVMNSVAFIGSNLWLSAWTSDNSTPASRDMRVGVYGALGLAQGIFVFIAHFWSAFGFVHASNILHKQLLNNILRAPMRFFDTTPTGRI : 949
Xtr.ENSXETG00000026360: --VALTGRVKLSVYLEYCKIMGKWYLLISALFFIVQQAASLSYNYWIGLWADDNGTQQHTSLRLGVYSFLGVMQALSIFAASSTIIVGGVSVSRQLHSRLLYSILRCPLSFFERTPSGNL : 939
Cel.WBGene00003410: HVRVETGRVKMDTYYKYFGAMGMSIAVLFVLGMTTSTIFSMGRNLWLTDWSNDGSNGQPIAIRLGVYAGLGFSEIILLFIGMLSLLYGGVSASRNLHAPLMRNLFRVPMAFYDTTPFGRI : 952
Ame.GB53134: SLKTETGSVKWRVYSHYFKSIGWFLSISTIIMNAIFQGFSIGSNTWLSMWSDDNNTVDHQNMYLGVYGGLGLGVGMTVLGGALFLAKGTIRASVHLFESTLQRVLRNPMSFFDQTPTGRI : 954
Pma.KM232931_1: HVKMETGRVSLAVYQQYGRAVGAPLALCVALLYALQGAATVGQGVWLSDWTGDNGTQGGTGPRLAVYAALGFTQGLMVLLYIVLGGVGMLRASKRLHADALIRVLRSPARFFEATPTGRL : 670
Gac.ENSGACG00000003037: SIGQRHGQVKLQMYREYFRTVGPTIIAAIVFLCAFQQAASLAYSYWLSLWADENATRSH-QLRLGVFAALGLTQGAAMFGTTLAIALGGIVASRHLHADLLLSVLRSPVSFFEATPSGNL : 959
Loc.ENSLOCG00000007152: DLRARTGRVKLEMYMEYFKTIGLALILPIIFLYAFQQAASLTYNYWLSMWADDNGTQLDTDLKLGVYGVLGFAQGVAIFGTNVAISVGGIIASRHLHQELLHNVLRSTMSFFERTPSGNL : 955
Gmo.ENSGMOG00000005748: DLKARTGRVRLDMYMEYFKTIGVALVVPIVLMYAFQQGASLTYNYWLSLWADGNGTQQGTDLKLAVFGALGLAQGIAIFGTTVAISLCGIVASRQLHRELLNNVLHSPMSFFETTPSGNL : 918
Gac.ENSGACG00000019172: SQKARSGRVRLATYNKYFKTIGLAIIVPIVFLYAFQQGASLAYNYWLSVWADDNGTQTDTDLKLIVFGALGFVQGIAIFGTTVAISVCGIIASSHLHADLLINVLRSPMSFFECTPSGNL : 960
Tni.ENSTNIG00000012067: AFKARTGRVRLEMYKKYFNTIGLAIIVPIIFLYAFQQGVSLAYNYWLSMWADDNGTQIDTDLKLTVFGALGFVQGVSIFGTTVAISICGIIASRHLHMDLLMNVLRSPMSFFECTPSGNL : 957
Ola.ENSORLG00000013429: DLKAHTGRVKLDMYKKYFKTIGLAIIIPIVFLYAFQQGASLAYSYWLSMWADDNGTQTDRDLKLAVFGALGFVQGIAIFGTTVAISICGIIASRQLHMDLLVNVLRSPMAFFESTPSGNL : 959
Oni.ENSONIG00000018866: DLKARTGRVRLDMYKKYFKTIGLAIIIPIVFLYAFQQGASLAYNYWLSKWADDNGTQIDTDLKLTVFGALGFVQGVAIFGTTVAISICGIIASRHLHMDLLNNVLHSPMSFFESTPSGNL : 959
Xma.ENSXMAG00000004906: DLKARTGRVRLTLYKKYFKTIGLAIIILIVFLYAFQQGASLAYNYWLSMWADDNGTQIDTDLKLSVFGALGFVQGIAIFGTTVAISICGIIASRQLHMDLLVNVLRSPMSFFESTPSGNL : 955
Dla.DLAgn_00177430: DLKARTGRVRLEMYQKYFKTIGLAIIIPIVFLYAFQQGASLAYNYWLSMWADDNGTQIDTDLKLTVFGALGFVQGIAIFGTTVAISICGIIASRHLHMELLINVLRSPMSFFECTPSGNL : 955
Dre.ENSDARG00000016750: DLKARIGRVKLEMYIEYFRTIGLPLIISIVFLYAFQQAASLSNNYWLSLWADQNGTQLNTDLKLGVYGALGFAQGISIFGTTVAISLGCIIASRHLHLDLLNNVLHSPMSFFESTPSGNL : 955
Ame.ENSAMXG00000004837: DLKARIGRVKLEMYIEYFRTIGLALIVPILFLYAFQQAASLAYNYWLSMWADENGTQLNTDMKLGVYGALGFAQGMAIFGTTVAISIGGIIASRQLHLDLLKNVLHSPMSFFESTPSGNL : 952
Ame.ENSAMXG00000003085: HLKAHTGRVKLEMYVEYFRTIGLALIVPIVFLYAFQQAASLGYNYWLRLWADDNGTQADADVKLAVFGALGIAQGVAIFGTTVAISLGGIIASRHLHMDLLSNVLHSPMAFFESTPSGNL : 960
Dre.ENSDARG00000094901: --KAHTGRVKLEMYVEYFRTIGLAFIIPIIFLYAFQQVASLAYNYWLSLWADDNGTQVNTDLKLGVYGALGFAQGIAIFGTTVAISLGGIIASRQLHLDLLNNVLHSPMSFFESTPSGNL : 927
Dre.ENSDARG00000095820: SNKAHTGRVKLEMYVEYFRTISLALIIPIIFLYAFQQAASLAYNYWLSLWADDNGTQVNTDLKLGVYGALGFAQGIAIFGTTVAISLGGIIASRQLHLDLLNNVLHSPMSFFESTPSGNL : 960
Lch.ENSLACG00000022117: HSTAQSERVKLAVYQEYFKKLGSFLFLYVIWLHICQQAASFSASYWLSLWADDNGTQQHVDVRLGVFGLLGFIQGATKFGSTMAIFVGGVMASQRLHSDLLRSVLRSPMSFFEKTPSGNL : 960
Dpu.347281: RAKTETGKVNSQVYVHYLQSVGGWLSFITLILYMIYQGFAVYSNIWLAKWSEAGNHEQQRDIYLGVYGALGLGQSIFLLIGTITISLGCLQASAILHEGMIARTFRLPMSHFDTTPIGRI : 959
Lca.KE993868_1: --HGKTTQMKLSVVLHYLRAVGAPLSTLALLLFCANYTTMLGTNVWLSEWAGENGTQRNTGYRLGVYSALGAV--QSVF-------KGCLRAAGTLHLALLDNKLHSPISFYDVTPLGRI : 393
Pma.KM232930_1: RRSMETGHMKLSVVLHYLRAVGAPLSTLALLLFCANYTTMLGTNVWLSEWAGENGTQRNTGYRLGVYSALGAVQSVLSLVMVLMVFKGCLRAAGSLHLALLDNKLHSPISFYDVTPLGRI : 960
Csa.ENSCSAVG00000008135: DGKAETGNVKLSVFLSYMSSIGFFLCFLICMFFIVQNAAQVYANIWLSDWSNDDGTQNGTEIRLAVYGSLGFIQALFVMASSFTLTYGSVAAAAMLHYNMLDRIFHAPMSYFDTTPLGRV : 951
Csa.ENSCSAVG00000003792: QRTAETGNIKLSVALSYMRSVGYLICSTIALAYCLQNAAQVGSSIWLSQWSTDDGTQNGTATRLGVYGALGMTQALMVLGSSFSMSYGSVVASTLLHSKMLVRIFRAPMSFFDTTPLGRI : 892
Cin.ENSCING00000020698: ------------------------------------------------------------------------------------------------------------------------ : -
Tca.TC012253: SLKAETGSVKWIVYKHYLKSIGVFLTVATILLNMLFQGFSIGSNVWLGVWADDDNVTGKRDFYLGIYGALGIGQGTCAFLSALSLYIGAVNGAQLLHHLLLSNILRVPCTFFDVTPVGRI : 953
Aga.AGAP009835: SKKSETGSVKWEVYKHYLKSIGLTLSVATVILNMIFQGFSIGSNLWLSRWSTDNDT-SRRDMYLGVYGAFGAGQVLANFVATLTFALGSLYAAKTMHELLLRYVLHWPMSLFDTTPLGRV : 949
Lgi.LotgiG107213: --SVETGRVKFTVFSAYIKAVGPLLTVFIIFFFIMTNAAAIYSNIWLSEWSDDNSTDYQRDMRLGVYAALGVLQGLFVLIAVLLKAIGARDASKILHKNFLLNVMRSPMKFFDTTPLGRL : 953
Bfl.90918: --KSKTGKVKASVYMAYVRSAGIVLPVLGVLGLTAQQAALVGSNFWLSDWSDDNGTQSARNIRLGVYGALGMAQGLLATAGAFCLILGGIRSSKTLHKDALLHVLRGSLQYFDVTPLGRI : 828
Dme.FBgn0032456: SIKSQTGGVEFAVYKHYIKSVGIFLSVATLVLNFVFQAFQIGSNLWLTQWANDNDT-GLRDMYLGVYGAFGFGQGVLAYFAVVIVYLGGFQAAKTIHNELLAVIIRGSVRFFDITPIGRL : 953
Oan.ENSOANG00000013379: ------------------------------------------------------------------------------------------------------------------------ : -
Pma.AKC42143_1: ---------------------------------------------------------------------------------DLTRPAGGAVPSAFLFLS--------------------- : 371
Lca.KE994284_1: ------EQVKLSVFLEYMRSVGLPLSLAVGLLYLTQNTAAVGSNVWLSEWANDDTRRPGTELRLGVYGALGGAQGLAALLSSIAISFGGVLASRHLHDAMLRRVLRCPAAFFERTPTGAL : 600
Cmi.SINCAMG00000015787: NQKAQTGRVKFSVYIKYIKAIGVCTSCLIVVAYICQHVAALFSNFWLSLWTDDNGTQPFNKLRLGVYSALGLGQGFFVLCSSVLMCAGGIIASKWLHADLLNDVLQSPMNFFERTPSGNL : 959
Ler.ctg13956: QSKALTGRVKFRVFWTYVKAIGVCVCFWIILFYLCQHIASLSSNYWLSLWTDDNGTQQRTPLRLGVYGALGIIQGVFVLISSLMMSIGGIKASRRLHVNVLYDVLMSPLSFFEQTPSGNL : 532
Rty.XP_020377569_1: EAKALTGRVKFSVFWAYMKAIGICISFCIVLFYLCQHVASLSSNYWLSLWTDDNGTQQHTSLRLAVYGALGLAQGVFVLLSSLMMSVGGISASRWLHVRLLYDTLMSPMSFFERTPSGNL : 411
Sca.ctg67278: QQKALTGRVKFSVFWSYIKAIGTCISFWIVLFYLCQHIASLSSNYWLSLWTDDNGTQQHTSLRLGVYGALGIIQGLFVLFASAVMSVGGISASRWLHDHVLFDILMSPMSFFERTPSGNL : 860
Lch.ENSLACG00000001471: STKAQTGKVKFSVFWEYMKAIGLFLFFLSIFLFTCHHVASLASNYWLSLWTDDNGTQQHTKVRLGVYGTLGILQGIAVLGYSMAISIGGILASRHLHLNLLHQILRSPISFFERTPSGNL : 960
Tni.ENSTNIG00000005013: TAKASTGRVKLSVFWAYLKAIGVLLSSISLLMFFTHHGVSLFSNYWLSLWTDDNGTQPYRVMRLGVYGSLGLAQGITVFGYSLCIFIGGILASRHLHQSMLYDVLRSPMSFFEKTPSGNL : 916
Loc.ENSLOCG00000007196: NSKAKTGKVKLSVYWEYMKAIGIFLSFFSIFLFLSHHSASLASNYWLSLWTDDNGTQQYTEVRLSVYGVLGLCQGIAVFGYSVSVSIGGILASRYLHQTMLHNVLRSPMSFFERTPSGNL : 960
Dre.ENSDARG00000104719: QSKANTGRVKLSVFWEYMKAIGLPLSIFSIFLFFCHHLSSLGSNYWLSLWTDDNNTQPKREMRLGVYGALGISQGIAVFCYSVSVSVGGILASRYLHQTMLYNVLRSPMSFFERTPSGNL : 955
Ame.ENSAMXG00000002943: QSKANTGRVKLAVFWEYMKAIGVFLSFISILLFLAHHVSSLGSNYWLSLWTDDNGTQPSREMRLGVYGALGLSQGIAVFCYSISVSIGGILASRYLHETMLYNVLRSPMSFFERTPSGNL : 955
Xma.ENSXMAG7738/17319: AVKASTGQVKLSVFWAYLKAIGVLLSCISLLLFLTHHLVSLFSNYWLSLWTDDNGTQPYRLRRLAVYGSFGLTQGILYF----------------------------------------- : 826
Ola.ENSORLG00000017141: GFKALTGRVKLSVFFSYLKAIGVLLSIISLLFFLSHNLLSLFANYWLSLWTDDNGTQPNRLMRLGVYGALGVSQGVAVCGYSLSVSIGGILASRFLHQSMLFDVLRSPMSFFERTPSGNL : 957
Gac.ENSGACG00000000434: SIKASTGRVKLSVFGSYLKAIGVLLSCVSLLMFLAQHLGSLSSNYWLSLWTDDNGTQPNRRMRLGVYGALGLFQGLAVFGYSLSGSIGGILASRCLHQSMLYDVLRSPMSFFERTPSGNL : 960
Oni.ENSONIG00000007824: GLKASTGQVKLSVFWAYFKSIGVLLSCISLLLFLAHHLLSLFSNYWLSLWTDDNGTQPNRLMRLGVYGAFGLSQGVAVFGYSLSMSIGGVLASRYLHQSMLYDVLRSPMSFFERTPSGNL : 960
Dla.DLA_00195360: ------------------------------------------------------------------------------------------------------------------------ : -
Xtr.ENSXETG00000019661: --KAKTGRVSYTFYYYYDPLLWMKTTFTE-------NEQFLSGSYGL-LQTNTAGPDHSNEAEGNVCHFLPIV-GIAVYGYSTAVSLGGILASRFLHTDLLHNVLRFPMSFFERTPSGNL : 942
Aca.ENSACAG00000005349: HSTAKTGKVKPRVYWEYMKATGLWLALLSLLLFLCNHVASLASNYWLSLWTDDNGTQQNTDLRLAVYGALGFSQGVAVFGYSMAVSVGGLLASRRLHLGLLHSVLRCPMGFFERTPSGNL : 955
Gga.ENSGALG00000006646: LMTAKTGRVKATVYWEYMKAIGLYISLLSVFLFMCNHIASLASNYWLSLWTDDNGTQQYTNVRLGVYGALGISQGIAVFGYSMAVSIGGIFASRHLHLDLLHNVLRSPMSFFERTPSGNL : 958
Dno.ENSDNOG00000014990: QLKAQTGQVKLSVYWDYMKAIGLFISFLSIFLFLCNHVAALASNYWLSLWTDDNGTQEHTQVRLSVYGALGISQGVAVFGYSMAVSIGGIFASRRLHLDLLHNVLRSPMSFFERTPSGNL : 958
Mmu.ENSMUSG00000023088: HLKAQTGQVQLSVYWNYMKAIGLFITFLSIFLFLCNHVSALASNYWLSLWTDDNGTQANRNFRLSVYGALGILQGAAIFGYSMAVSIGGIFASRRLHLDLLYNVLRSPMSFFERTPSGNL : 958
Ggo.ENSGGOG00000004324: QLKAQTGQVKLSVYWDYMKAIGLFISFLSIFLFMCNHVSALASNYWLSLWTDDNGTQEHTKVRLSVYGALGISQGIAVFGYSMAVSIGGILASRCLHVDLLHSILRSPMSFFERTPSGNL : 888
Hsa_ENSG00000103222: QLKAQTGQVKLSVYWDYMKAIGLFISFLSIFLFMCNHVSALASNYWLSLWTDDNGTQEHTKVRLSVYGALGISQGIAVFGYSMAVSIGGILASRCLHVDLLHSILRSPMSFFERTPSGNL : 958
Ptr.ENSPTRG00000007812: QLKAQTGQVKLSVYWDYMKAIGLFISFLSIFLFMCNHVSALASNYWLSLWTDDNGTQEHTKVRLSVYGALGISQGIAVFGYSMAVSIGGILASRCLHVDLLHSILRSPMSFFERTPSGNL : 912
Cluf.ENSCAFG00000018208: QLKAQTGQVKLSVYWDYMKAIGLFISFLSIFLFLCNHVASLVSNYWLSLWTDDNGTQEHTKIRLSVYGALGISQGITVFGYSMAVSIGGIFASRRLHVDLLQNVLRSPMSFFERTPSGNL : 958
Bta.ENSBTAG00000021090: QMKAQTGQVKLSVYWDYMKAIGLFISFLSIFLFLCNHMASLVSNYWLSLWTDDNGTQEHTQVRLSVYGALGISQGITVFGYSMAVSIGGIFASRRLHLDLLHNVLRSPISFFERTPSGNL : 958
Mdo.ENSMODG00000004194: QLKAKTGQVKLSVYWDYMKAIGLFISFLSIFLFICNHVASLASNYWLSLWTDDNGTQQHTNVRLSVYGALGISQGPARSLRLVSFTSSWHLCFPRLHLDLLHNVLRSPMSFFERTPSGNL : 958
Oan.ENSOANG00000005124: QLKAKTGQVKLSVYWEYMKAIGLFISFLSIFLFICNHVAALASNYWLSLWTDDNGTQQYTDVRLGVYGALGISQGIAVFGYSMAVSIGGICASRRLHLDLLHSVLRSPLSFFERTPSGNL : 747
Bfl.232174: GIIAETGRVKSSVFVEYLRSVGITLSVIVCLLYCTQNAASIYSNIWLSEWSNDNGTQDIRDLRLGVYGALGTLPGVCVVVTAFLSAYGGIRSSLHLHNKSLLNILRSPMQFFDVTPMGRI : 959
Bfl.118638: GIIAETGRVKSSVFVEYLRSVGITLSVIVCLLYCAQNAASIYSNIWL----------------------------VCVVVTAFLSAYGGIRSSLHLHNKSLLNILRSPMQFFDVTPMGRI : 822
Bfl.118636: RLVAETGRVKASVFVEYLRSVGITLSVIICLLYCAQNAASIYSNIWLSEWSNDNGTQDIRDLRLGVYGALGVAQGLFSMFSSFALAIGALFASTTLHAGLMNNILHLPMQFFDVTPMGRI : 946
Bfl.128060: RLVAETGRVKASVFVEYLRSVGITLSVIICLLYCAQNAASIYSNIWLSEWSNDNGTQDIRDLRLGVYGALGVAQGLFSMFSSFALAIGALFASTTLHAGLMNNILHLPMQFFDVTPMGRI : 946
Dre.ENSDARG00000096662: EATTETGRVKLQVYWEYVKAIGPLLALFICFLYGCQSAAAIGANFWLSEWTNDNRTQEMLQTRVGVYAALGISQGVLILICLLCRAYSTLRAARHTHQHMLNSVLRAPQAFFEATPSGRV : 956
Ame.ENSAMXG00000016253: RRTAETGRVKFKVYWEYAKAVGPILSLFICFLYASQSAAAIGANIWLSQWTNDDSVQEKVHMRVGVYASLGITQGILVMFSSFTLAMGKIQAARKLHQGLLDNKFHTPQAFFDTTPLGRI : 959
Ola.ENSORLG00000020741: FMTAETGRVKTKVYLEYVKAVGVLLSVLILLLYGCQSAAAIGSNIWLSQWTNDAGNQENVQMRVSVYAALGIAQGILVMISSYTLAMGNISAARRLHANLLTNKLHTPQSFFDTTPIGRI : 957
Gmo.ENSGMOG00000010029: KRTSETGRVKMKVFMDYAKAVGLVLSVIICLLYGCQNAAAIGANVWLSDWTKDNQTRDEVNMRLGVYASLGIAQGLLVMVSSFTLAVGNIGAAKKLHYNLLNNKFHTPQAFFDTTPLGRL : 914
Xma.ENSXMAG00000012203: KKTTETGRVKTKVFLEYAKAVGLVLSVIICLLYGCQSAAAIGSNIWLSQWTSDNDTQNNVHKRVGVYAALGIAQGILVMASSFTLAMGNIGAAKKLHANLLNNKLHTPQSFFDTTPIGRI : 958
Gac.ENSGACG00000005901: FVTAETGRVKSKVYLEYAKAVGVLLSVFICFLYGCQSAAAIGANIWLSQWTNDNQTQENLNMRVGVYAALGIAQGILVMMSSFTLAMGNIGAARKLHANLLTNKFHTPQSFYDTTPLGRI : 952
Oni.ENSONIG00000019586: EATAETGQVKGKVYLEYVKAVGPLLSVVICFLYGCQSAAAIGTNIWLSEWTNDNSTTENVQMRVGVYAALGFAQGILIMIASFTLAMGNIGAAKKLHVNLLTNKFHTPQSFFDTTPIGRI : 955
Dla.DLAgn_00098120: KRTAETGRVKAKVYLEYAMAVGPLLSVFICFLYGCQSAASIGANIWLSEWTNDNQTQENVPMRVGVYAALGIAQGILVMMSSFTLAMGNIGAAKKLHYNLLTNKLHTPQSFFDTTPIGRI : 960
Cmi.SINCAMG00000013336: RKTTETGTVKLAVFWVYVKAVGPWVAVVICLLYCCQNAAAIGTNIWLSDWTNDQSTTRSRHRRVGVYAALGLTQGVLVMISSFTLTLGGIVAARLLHSRLLHNQLHTPQAFFDTTPTGRI : 860
Ler.ctg12190: RLTSETGRVKLAVFWQYLKAVGPCTSVMICLLYCCQNAAAIGANFWLSEWTNDSNKTQQISMRVGVYAALGLTQGVLVMISSLTLMLGGLGAARQLHARLLVNKLHTPQAFYDTTPIGRI : 859
Sca.ctg14163: --KAETGRVKLVVFWQYLKAVGPLVSVVICIMYCCQNAAAIGASFWLSAWTNDNRTQHQTHLRVGVYAALGFAQGVLVMISSFTLAIGGLGAAQQLHARLLENKLHTPQAFFDTTPIGRL : 799
Rty.XP_020375725_1: EATTETGRVKLIVFWQYLKAVGPFISVVICILYCCQNAAAIGANFWLSDWTNDNRTQQQTYMRVGVYAALGFTQGILVLISSFTLALGGLGAARQLHTRLLDNKLHTPQAFFDTTPIGRI : 379
Sac_EU250283: RKTTETGRVKLTVFWQYLKAVGPFISVVICFLYCCQNAAAIGANFWLSDWTNDNGTQHRTNMRVGVYAALGFTQGVVVMISSFTLALGGLGAARQLHARLLDNKLHTPQAFFDTTPIGRI : 960
Tni.ENSTNIG00000004171: KRATETGRVKMKVYLEYVKAVGPLLSVFICFLYGCQSAAAIGANIWLSVWTNDNQTQENVNMRVGVYAALGLAQGILIMISSFTLAMGNIGAARKLHHNLLLNKLHTPQSFFDTTPIGRI : 909
Aca.ENSACAG00000001396: EATAETGTVKFTVFWQYMKAVGPIVSLFICFFYCCQNAAAVGANVWLSDWTNENGTQHNVPMRVGVYGALGLLQGLFVLASSFTLAMGGIRAARSLHAGLLENKLHTPQSFYDTTPTGRI : 954
Gga.ENSGALG00000007522: EVTTEVGTVKLTVFWQYMKAVSPVISLIICFLYCCQNAASIGANVWLSDWTNENGTQHNTSMRIGVYAALGLLQGFIVFVSSFTLAMGGINAARKLHTALLENKFHTPQSFYDTTPTGRI : 905
Xtr.ENSXETG00000012239: EATTETGRVKMTVFWQYMKAVGLAISVFICFLYSCQNAAAIGANVWLSDWTNENQTQQNTQMRVGVYAALGILQGLLVMTSSFSLAIAGIGAARKLHSALLDNKMHTPQSFYDTTPIGRI : 957
Loc.ENSLOCG00000010918: EATAETGRVKLKVFLEYIKAVGPFFSVFICFLYACQNAAAIGANVWLSAWTNDNGTQVDTQMRVGVYAALGMTQGLLVLISSFTLTLAGIGAAKNLHSALLENKFQTPQSFFDTTPIGRI : 957
Lch.ENSLACG7209/6619: EATTETGRVKLKVFWQYIQAVGPAISMFICLLYCCQNAANIGANYWLSDWTNENGTQQNTRMRVGVYAALGLS--LLVMISSFTLALGGIGAARKLHFALLENKFHTPQSFFDTTPIGRI : 940
Mdo.ENSMODG00000020910: ------------------------------------------------------------------------------------------------------------------------ : -
Dno.ENSDNOG00000046300: EVKAEMGNVKLSVYWDYAKAMGVWTMLAAHLMFGVQGATAIGADMWLSAWTNEDGRQNNTSLRLGVYATLGVLQGLLVTLSALIMAVGSVQAARLLHRALLHNQMHSPQSFFDTTPSGRI : 958
Mmu.ENSMUSG00000020865: EVIAETGNVKLSVYWDYAKSMGLCTTLSICLLYGGQSAAAIGANVWLSAWSNDHGQQNKTSVRLGVYAALGILQGLLVMLSAFTMVVGAIQAARLLHEALLHNKIRSPQSFFDTTPSGRI : 957
Ggo.ENSGGOP00000003354: QKKAAIGTVELSVFWDYAKAVGLCTTLAICLLYVGQSAAAVGANVWLSAWTNDDSRQNNTSLRLGVYAALGILQGLLVMLAAMAMAAGGIQAARVLHQALLHNKIRSPQSFFDTTPSGRI : 960
Hsa.ENSG00000108846: QKKAAIGTVELSVFWDYAKAVGLCTTLAICLLYVGQSAAAIGANVWLSAWTNDDSRQNNTSLRLGVYAALGILQGFLVMLAAMAMAAGGIQAARVLHQALLHNKIRSPQSFFDTTPSGRI : 960
Ptr.ENSPTRG00000009406: QKKAAIGTVELSVFWDYAKAVGLCTTLAICLLYVGQSAAAIGANVWLSAWTNDDSRQNNTSLRLGVYAALGILQGLLVMLAAMAMAAGGIQAARVLHQALLHNKIRSPQSFFDTTPSGRI : 960
Cluf.ENSCAFG00000017201: QKKAEMGTVKLSVFWDYAKAMGLYSTVAICLLYPGQSAASIGANVWLSAWTNEESQQNNTSMRLGVYAALGILQGLLVMLSAITLTVGSVQAARFLHQALLHNKMRSPQSFFDTTPSGRI : 960
Bta.ENSBTAG00000020070: QKKTELGTVKLSVYLDYAKAVGLWTALVICLLYGGQSAAAIGANVWLSAWTDEDSQQNSTSYRLGVYAALGILQGLLVMLSAITMAVGGVQAARLLHQALLHNKMRSPQSFFDTTPSGRI : 960

Hsa.CFTR.ENSG0000000162: LNRFSKDIAILDDLLPLTIFDFIQLLLIVIGAIAVVAVLQPYIFVATVPVIVAFIMLRAYFLQTSQQLKQLESEGRSPIFTHLVTSLKGLWTLRAFGRQPYFETLFHKALNLHTANWFLY : 882
Hsa.ABCC5.ENSG000001147: LNRFSKDMDEVDVRLPFQAEMFIQNVILVFFCVGMIAGVFPWFLVAVGPLVILFSVLHIVSRVLIRELKRLDNITQSPFLSHITSSIQGLATIHAYNKGQEFLHRYQELLDDNQAPFFLF : 962
Hsa.ABCC11.ENSG00000121: LNCFAGDLEQLDQLLPIFSEQFLVLSLMVIAVLLIVSVLSPYILLMGAIIMVICFIYYMMFKKAIGVFKRLENYSRSPLFSHILNSLQGLSSIHVYGKTEDFISQFKRLTDAQNNYLLLF : 945
Hsa.ABCC12.ENSG00000140: MNRFSKDMDELDVRLPFHAENFLQQFFMVVFILVILAAVFPAVLLVVASLAVGFFILLRIFHRGVQELKKVENVSRSPWFTHITSSMQGLGIIHAYGKKESCI--------TYHLLYFNC : 914
Hsa.ABCC8.ENSG000000060: LNRFSSDCNTIDQHIPSTLECLSRSTLLCVSALAVISYVTPVFLVALLPLAIVCYFIQKYFRVASRDLQQLDDTTQLPLLSHFAETVEGLTTIRAFRYEARFQQKLLEYTDSNNIASLFL : 1064
Hsa.ABCC9.ENSG000000694: LNRFSADTNIIDQHIPPTLESLTRSTLLCLSAIGMISYATPVFLVALLPLGVAFYFIQKYFRVASKDLQELDDSTQLPLLCHFSETAEGLTTIRAFRHETRFKQRMLELTDTNNIAYLFL : 1061
Hsa.ABCC10.ENSG00000124: LNRFSSDVACADDSLPFILNILLANAAGLLGLLAVLGSGLPWLLLLLPPLSIMYYHVQRHYRASSRELRRLGSLTLSPLYSHLADTLAGLSVLRATGATYRFEEENLRLLELNQRCQFAT : 1053
Hsa.ABCC4.ENSG000001252: LNRFSKDIGHLDDLLPLTFLDFIQTLLQVVGVVSVAVAVIPWIAIPLVPLGIIFIFLRRYFLETSRDVKRLESTTRSPVFSHLSSSLQGLWTIRAYKAEERCQELFDAHQDLHSEAWFLF : 877
Cel.WBGene00003414: INRLSRDLDVIDK-LQDNIRMCTQTLLNACMILVLISISTPIFLVCAAPLILIYYFVMIYYIPTSRQLKRLESANRSPILSTIAESIHGASSIRAFDKTERTTTALSTNVDKFAQCRYLS : 1031
Cel.WBGene00003409: INRLAKDMEVVDLRLSSSFRFLVMALIN--MTVLIVSYTTPLFIAIIIPVFIIYFFVLKYSIKSTRQLQRIASLTRSPIFSNFSETLQGISTVRAFQWSDEFVRRNDEHLNTHVKCSYYS : 1067
Hro.HelroG163344: LNRFSKDVET------------------------------------------------RFYIPTTRQLKRLESTTRSPIYVHFSETISGASVIRAFGKQKDFIAKSEKLVDNNMVAYFAN : 988
Hro.HelroG157076: IFRHHPHWENYKQIFQGR-----------------------------------YRCKSRFYIPTNRQLKRLESTTLSPIYVHLSESIAGTSIIRAFKKQEDFIEKCYRLVDHNLQMYFAN : 710
Bfl.230771: LSRFSQDQDRVDKGIQWVICGCTYVILWTVGTLFVVIFSTPLFVVMLLPIACLYFYMQRYFNATAQQLRRLESKRGTAIHSHFSETLQGVSTIRAFSRCQQFVTQHQARVDEHQTAFYCN : 1060
Lgi.LotgiG105097: MNRFSKDVDILDTGLNQIFRLFMHSLFIVLSIIVVITLSTPWFLAPAVPIFIVYYFIQRFYIPTSRQLWRNESKARSPLYSHFTETITGASSIRAYSVVDKFREQSQDKMDRNNALHFAS : 1060
Cel.WBGene00003407: LNRFGKDIDVIDYRLPSCIMTFVGAIVQAVTIFAVPIYATPLSSFPITIVLIGYYFLLRFYVSTSRQLKRLESASRSPIYSHFQESIQGASSIRAYGVVDKFIRESQHRVDENLATYYPS : 1075
Cel.WBGene00003408: LNRIGKDIEAIDRTLPDVIRHMSMTIFNVVATLVVIMWATPWAGIAFAILSVIYFIVLRFYISTSRQLKRLESASRSPIYSHFQESIQGASSIRAFGVVDNFIKQSQQRVDDHLIAYYPS : 1074
Lgi.LotgiG153611: INRFSRDVETIDNNLPQIFSMWIVTIYSVASTFVVISVATPLFLMVIVPLILMYSFIQRYFVPTSRQLKRLESVSRSPIYSHFGESIQGATTIRAYEATYRFTEQSRKLIDKNQVYYFAG : 1020
Lgi.LotgiG110718: INRLSRDTETIDSTLPQVMKMWLSTFLIVSATILVISITTPIFIAIFIPIGAFYYFVQEFYIPTSRQLKRIESVTRTPIYTHFSETLSGASTIRAYGVTGSCLHHIKNIVDKNQVYYFAG : 887
Dno.ENSDNOG00000024923: LNRFSKETDVVDVDIPDKLQALLTYAFGLLEASLVVAVATPLAIAVILPLLLLYAGFQSLYVASSCQLRRLESASHSPVCSHVAETFQGSPVVRAFRAQGPFVAQSNAHVDKSQRVSFSR : 1014
Mmu.ENSMUSG00000030834: LNRFSKETDTVDVDIPDKLRSLLTYAFGLLEVGLAVTMATPLAIVAILPLMVLYAGFQSLYVATSCQLRRLESARYSSVCSHMAETFQGSLVVRAFRAQASFTAQHDALMDENQRVSFPK : 1072
Cluf.ENSCAFG00000018197: LNRFSKETDIVDVDIPDKLRSLLIYVFGLLEVSLVVTVTTPLAMMAILPLLVFYAGFQSLYVASICQLRRLESARHSFVCSHVAETFQGSVVVRAFQAQCRFVAQNDTHVDESQRVNFPR : 1073
Bta.ENSBTAG00000015191: LNRFSKETDIVDVDIPDKLRSLLMYAFGLLEVGLVVTVTTPLAVVAILPLLLLYAGFQSLYVASSCQLRRLESARYSYVCSHVAETFQGGPVVRAFRVQGPFTAQNDAHVDESQRVSFPR : 1074
Ggo.ENSGGOG00000009623: LNRFSKETDTVDVDIPDKLRSLLMYAFGLLEVSLVVAVATPLAIVAILPLFLLYAGFQSLYVVSSCQLRRLESASYSSVCSHMAETFQGSTVVRAFRTQAPFVAQNNARVDESQRISFPR : 1065
Hsa.ENSG00000091262: LNRFSKETDTVDVDIPDKLRSLLMYAFGLLEVSLVVAVATPLATVAILPLFLLYAGFQSLYVVSSCQLRRLESASYSSVCSHMAETFQGSTVVRAFRTQAPFVAQNNARVDESQRISFPR : 1073
Ptr.ENSPTRG00000007815: LNRFSKETDTVDVDIPDKLRSLLMYAFGLLEVSLVVAVATPLAIVAILPLFLLYAGFQSLYVVSSCQLRRLESASYSSVCSHMAETFQGSTVVRAFRTQAPFVAQNNARVDESQRISFPR : 930
Oan.ENSOANG00000005123: LNRFSKDMDAIDAEIPDKLKSFLGFMCGLLEVCLVVVVATPMATLVILPLLLFYGVFQSFYVASSCQLRRLESASQSPIYSHISMTFQGSGVIRAFRAQSRFVSRSDGHVDENQRVSFPR : 1028
Mdo.ENSMODG00000005815: LNRFSKETDAVDAVIPDKFKSFLGFLFGLLEVIVVVVVATPLAAVMVLPLMALYVGLQSLYVASSCQLRRLESASRSPIYSHISETFQGNAVIRAFQAQDQFIAQNDSRIDEHQRASFPR : 1073
Gga.ENSGALG00000006698: LNRFSKEMDAIDSIIPDKLKSLLGFLFNLLEIYLVIVVVTPKAAMAIVPLTAFYAVFQHFYVITSCQLRRMEAASRSPIYSHISETFQGSSVIRAYKDQERFILKINCLVDENLRICFPG : 1076
Aca.ENSACAG00000003478: LNRFSKDMDAVDSIIPDKLKSLLGFFFVLLEIYIVIIVATPIVVVAIVPLTVLYAVSQNFFIATSCQLKRLEAASRSPIYSNISETFEGSNSIRAYKAQQRFVLQNDFNVDENQRASYPA : 1016
Cel.WBGene00003413: LNRCAKDIETIDMMLPMNFRYLVMCVLQVAFTLIVIIISTPLFAVVILPLALIYLIFLRYYVPTSRQLKRLESVHRSPIYSHFGETIQGAASIRAFGKVDEFRQDSGRILDTFIRCRYSS : 1072
Aga.AGAP008437: MNRFSKDVDVVDNILPQSIRAWLLMFFNVIGVFVVIGISTPIFLAVVPAFLVIYYLIQKFYIATSRQLKRLESVTRSPIYSHFGESITGQSTIRAYGQQDRFMNESEQRVDYNQLTSYPS : 1071
Aga.AGAP027980: INRFSKDVDVVDNILPATIRAWLLMLFSVIGVFVVIGISTPIFLAIVPPLMIIYYFVQRFYIETSRQLKRLESVTRSPIYSHFGESIGGQSTIRAYAQQERFIRESEHRVDYNQLVTYPT : 1073
Aga.AGAP028128: INRFSKDVDVVDNVLPVTIRAWLLFLFNVFGVFIVIGTSTPIFLAVVPPLMVIYYFVQRFYIDTSRQLKRLESVTRSPIYSHFGESIGGQSTIRAYGQQDRFTQESERRVDYNQLVSYPT : 1070
Spu.026395: ARKLSKGIEAISQY----------DIFN-----------TPS--------------------------KKTDHVMKTEAY---DEVVMG------------------------------- : 860
Hsa.ABCC2.ENSG000000238: VNRFAGDISTVDDTLPQSLRSWITCFLGIISTLVMICMATPVFTIIVIPLGIIYVSVQMFYVSTSRQLRRLDSVTRSPIYSHFSETVSGLPVIRAFEHQQRFLKHNEVRIDTNQKCVFSW : 1069
Xtr.ENSXETG00000026360: TNRFAKEMDIIDNTVPQVLMLFIIMMLTIAEILLVISIATPLAAVAFIPLGLLYFFLQRFYVASSRQLKRLDAVSKSPLYTHFNESLQGVYVIRAFREQERFIQDNNMRLNMNQRFYFCS : 1059
Cel.WBGene00003410: LNRIGKDIETVDVLLPFNVQFFAQCLLQVVSTLIIIMISTPVFGIVIIPLSVMYLMVMRYYIATSRQLKRLESITRSPIYSHLSESIQGSATIRAYHLVDRFCKLSETKVDSHVQCRYLN : 1072
Ame.GB53134: LNRLSKDTDVIDNTLPSILRSWITCLFG-------------------------------------------------------------------------------------------- : 982
Pma.KM232931_1: LNRFGRDVDSLDTLVPENLDAWLRCFCYTLATLMIISIATPTFLLAATPLAIAYWFIQRYYVATSRQLKRLESVSRSPVLSHLADTVLGRAVVRAHGQQWRFASDNARHIDANQRPTFAG : 790
Gac.ENSGACG00000003037: LNRFSKEVDAIDCMIPDGLKMMLGYLFKLLEVCVVLLLATPLTGLVLLPLACIYIFIQSFYVASSCQLRRLEAVSRSPVYSHLNETVQGAAVVRAFGEQGRFVLEADRRVDRNQEAYFPR : 1079
Loc.ENSLOCG00000007152: LNRFSKEIDAIDCMIPDGLKMMLGYLFKLLEVCIIVLLAMPFAAVVILPLALFYCFIQSFYVATSCQLRRLESVSRSPIYTHFNETVQGVSVIRAFREQPRFILQANHRVDYNQTSYFPR : 1075
Gmo.ENSGMOG00000005748: LNRFAKEIDGIDCMIPDGLRMMLGYVFKLLEVCVIVMMATPFAAVFILPLAFLYAFVQSFYVATSCQLRRLEAVSRSPIYTHFNETVQGASVIRAFREERRFILQANRRVDLNQTAYFPF : 1038
Gac.ENSGACG00000019172: LNRFSKEIDAIDCMVPDGLKMMLCYVFKLMEVCIIVLLAMPFAAVVILPLALLYAFVQSFYVATSCQLRRLEAVSRSPIYTHFNETVQGASVIRAFGEQSRFILQTNKRVDFNQTSYFPR : 1080
Tni.ENSTNIG00000012067: LNRFAKEIDAIDCMVPEGLKMMLSYAFKLLEVCIIVMMATPFAAVIILPLAFLYACVQSFYVATSCQLRRLEAVSRSPIYTHFNETVQGASVIRAFGEQPRFILQANKRVDFNQTSYFPR : 1077
Ola.ENSORLG00000013429: LNRFVKEIDAIDCMVPEGLKMMLSYVFKLVEVCIIVLIATPIAAVIILPLAFLYAFVQSFYVATSCQLRRLEAVSRSPIYTHFNETVQGASVIRAFGEQSRFIMQANERVDFNQTSYFPR : 1079
Oni.ENSONIG00000018866: LNRFAKEIDAIDCMVPEGLKMMLSYVFKLMEVCIIVLMATPFAAVIILPLSFLYAFVQSFYVATSCQLRRLEAVSRSPIYTHFNETVQGASVIRAFGEQSRFILQANDRVDFNQTSYFPR : 1079
Xma.ENSXMAG00000004906: LNRFAKEIDAIDCMVPEGLKMMLSYVFKLLEVCIIVLMATPFAAVIILPLAFLYAFVQSFYVATSCQLRRLEAVSRSPIYTHFNETVQGASVIRAFGEQPRFILQANERVDFNQTSYFPR : 1075
Dla.DLAgn_00177430: LNRFAKEIDAIDCMVPDGLKMMLSYVFKLMEVCIIVLMATPFAAVIILPLAFLYAFVQSFYVATSCQLRRLEAVSRSPIYTHFNETVQGASVIRAFGEQPRFILQANQRVDFNQTSYFPR : 1075
Dre.ENSDARG00000016750: LNRFAKEIDAIDNMIPDGLKMMLSYFFKLTEVCIIVLMATPFAAVIILPMVFLYGFIQSFYVATSCQLRRLESVSRSPIYTHLNETVQGASVIRAFNEQSRFIMGANHKVDHNQTAYFPR : 1075
Ame.ENSAMXG00000004837: LNRFAKEIDAIDCMIPDGLKMMLSYFFKLMEVCIIVLLATPFAAAIIFPLALLYAFVQSFYVATSCQLRRLESVSRSPIYTHFNETVQGASVIRAFSEQSRFILQANRRVDVNQTSYFPR : 1072
Ame.ENSAMXG00000003085: LNRFSKEVDAIDCMIPDGFKMMLGYVFKLMEVCIIVLIATPFAGLVILPLTLFYVFIQSFYVATSCQLRRLESVSRSPIYTHFNETVQGASVIRAFGEQPRFILQANGRVDHNQTSYFPR : 1080
Dre.ENSDARG00000094901: LNRFSKEIDAIDCMIPHGLKIMLGYVFKLLEVCIIVLMATPFAGVIILPLTLLYAFIQSFYVATSCQLRRLESVSRSPIYTHFNETVQGASVIRAFGEQPRFILQANCRVDLNQTSYFPR : 1047
Dre.ENSDARG00000095820: LNRFSKEIDAIDCMIPDGLKMMLGYVFKLLEVCIIVLMATPFAGVIILPLALLYAFIQSFYVATSCQLRRLESVSRSPIYTHFNETVQGASVIRAFGEQPRFILQANCRVDLNQTSYFPR : 1080
Lch.ENSLACG00000022117: LNRFSKEIDAIDTVIPNGIKSLLGFLFSLLEVYLVVLVATPIAAVIIVPLTVMYCVIQSFYVATSCQLRRLESVSRSPIFSHVNETYQGASVIRAFGEQMRFLSQNDSKVDENQKAYYPS : 1080
Dpu.347281: VNRFAKDVDVVDNLIPSSIRTALLCFLSVISTILVIGLGTPIFFAVAVPIGVLYYWIQNVYVATSRQLKRLESVSRSPIYSHFGETLTGATVIRAYGQEQRFIKESESRVDLNQICYYPS : 1079
Lca.KE993868_1: INRFSK---------------------------------------------------QRFYVATSRQLKRLESTVRSPVFSHFGESVAGAATLRAFRQEERFVIAGDRAVDALQRTCYPS : 462
Pma.KM232930_1: INRFSKDVFMIDEVLPPTFLMFLGALCNSIFTILIIVISTPVFVVAAIPLVVIYFFVQRFYVATSRQLKRLESTVRSPVFSHFGESVAGAATLRAFRQEERFVIAGDRAVDALQRTSYPS : 1080
Csa.ENSCSAVG00000008135: INRFSKDVYLVDEVIPRTLNAFISCFFKVFATLFVICYATPLFVIALLPILLIYYSVQRFYVSTSRQLKRLESISRSPIYSHFSETITGASTIRAYGLQNSFIKQSENLVDVNQMAYFPN : 1071
Csa.ENSCSAVG00000003792: VNRFSKDIYLIDEVIPRCFTGLLMTGFQCLSTFVVIVYSTPIFAVVVVPLLILYYFVQRFYIRTSRQLKRLESISRSPIYSHFSETIAGVSTIRAYGLQKSFMKQNEIKLDTNQMAYYPN : 1012
Cin.ENSCING00000020698: ------------------------------------------------------------------------------------------------------------------------ : -
Tca.TC012253: LNRFSKDVDTLDNILPMTLRGWITCFFSVLGTLVVISVSTPIFIAVIVPIGILYYFIQRFYVATSRQLKRLESVSRSPIYSHFGETITGVQAIRAFREQDRFIKESEHRVDVNQVCYYPS : 1073
Aga.AGAP009835: LNRFSKDVDTVDNTLPQLIRSFLAQFFAVVATLVVISISTPIFAAVIVPIGILYYAVQRFYVATSRQLKRLESVSRSPIYSHFGETIQGVQTIRAYSVQDRFILESDEKVDGNQLCYCPS : 1069
Lgi.LotgiG107213: VNRFGKDVDVIDITIPQTIRMWLHCFLTVVSTFVVVSISTPYFMTVAIPLLILYYFIQRFYIATSRQLKRLESISRSPIYSHFGETVTGAVTIRAFGQQERFITESENKVDENQICYFPS : 1073
Bfl.90918: VNRFSQDLNTIDNSMPSIIGMLLNFILTLMATMLVISISTPIFLAVLLPVAVLYFFIRRFYIATSRQLQRLEAVSRSPIYSHFNETLQGTSVIRAYGRTDQFGRENQDKVDHSQAAAYPG : 948
Dme.FBgn0032456: LNSFSGDMDVVDEELPATMDSFMTFIFMVLATIVVISLSTPIFLAVIVPIAFLYYFAQRFYVATSRQLMRLESVSRSPIYSHFSETVTGASTIRAYNVGDRFIEESDAKVDKNQVCKYPS : 1073
Oan.ENSOANG00000013379: ------------------------------------------------------------------------------------------------------------------------ : -
Pma.AKC42143_1: ------------------------------------------------------------------------------------------------------------------------ : -
Lca.KE994284_1: VNRFSRDVHTVDETIPGVGRMFLGSLCNVLAVFAVILLATPVAALAFLPLGAIYFFVQ-------------------------------------------------------------- : 658
Cmi.SINCAMG00000015787: VNRFAKDIDTIDSMIPTVIKMFLGSLFNVLIACIVILIATPIVAVTFLPLGFVYFFVQKFYVATSRQLKRLESVSRSPIYSHFNETLLGVSVIRAFGEQDRFLQENDLRVDENQKAYYPS : 1079
Ler.ctg13956: VNRFAKDIETIDSVLPMVIKMFLGSLFNVLAACVVILIATPLTAVIIPPLGFLYIFVQRYYVATSRQLKRLESASRSPIFSNFNETLLGVSVIRAFGKEDRFIDRNDSRVDENQKAYYPS : 652
Rty.XP_020377569_1: VNRFSKDIDTIDSMIPVVIKMFLGSLFNVLVSCTVILIATPIATVIIPPLGFLYIFIQRYYVATSRQLKRLESASRSPIFSNFSETLLGVSVIRAFGEQERFIHQNDARVDENQKAYYPS : 531
Sca.ctg67278: VNRFSKDIDTIDSVIPVVIRMFLGSLFHVLASCTVILVATPMATVIIPPLGFLYIFVQRYYVATSRQLKRLESASRSPIFSNFNETLLGVSTIRAFGEEDRFIYQNDSRVDENQKAYYPS : 980
Lch.ENSLACG00000001471: VNRFSKEIDTIDTLLPAIIKMFMGSTFNVISACIVILFATPIVAVIIPPLGLLYFFVQ-------------------------------------------------------------- : 1018
Tni.ENSTNIG00000005013: VNRFAKEMDTIDSVIPNIVKMFTGSMFTVIGACIIILISTPLVAAIIPFLGLLYFFVQ-------------------------------------------------------------- : 974
Loc.ENSLOCG00000007196: VNRFAKETDTIDSVIPGIIKMFMGSLFNVLGACIVILIATPMIAVIIPPLGLLYFFVQRFYVATSRQLKRLESVSRSPVYSHFNETLLGTSVIRAFQDQERFIKESDSRVDYNQKAYYPS : 1080
Dre.ENSDARG00000104719: VNRFAKETDTIDSVIPSIIKMFMGSMFNVLGSCAVILIATPLVAIIIPPLGLLYFFVQRFYVASSRQMKRLESVSRSPVYTHFNETLLGTSVIRAFGEQQRFIKESDGRVDHNQKAYFPS : 1075
Ame.ENSAMXG00000002943: VNRFAKETDTIDSVIPSIIKMFMGSMFNVLGSCAVILIATPLVAIIIPPLGLLYFFVQRFYVASSRQLKRLESVSRSPVYTHFNETLLGTSVIRAFGEQQRFIGESDRRVDHNQKAYFPS : 1075
Xma.ENSXMAG7738/17319: ------------------------------------------------------------------------------------------------------------------------ : -
Ola.ENSORLG00000017141: VNRFAKEMDTIDSVIPMILKMFMGSLFNVVGSCIIILVATPMVALIIPFLGVLYFFVQRFYVASSRQLKRLESVSRSPIYTHFSETLLGTSVIRAFGEQERFIHESDQRVDHNQKAYYPG : 1077
Gac.ENSGACG00000000434: VNRFAKEIDTIDSLIPSIIKMFMGSMFNVVGSCIIILIATPLVAIIIPFLGVLYFFVQRFYVASSRQLKRLESVSRSPIYTHFNETLLGTSVIRAFGEQERFICESDQRVDLNQKAYYPG : 1080
Oni.ENSONIG00000007824: VNRFAKEMDTIDTLIPSIIKMFLGSMFNVLGSCVIILIATPLVSIIIPFLGLLYFFVQRFYVASSRQLKRLESVSRSPIYTHFNETLLGTSVIRAFGEQERFIHESDQRVDHNQKAYYPS : 1080
Dla.DLA_00195360: ------------------------------------------------------------------------------------------------------------------------ : -
Xtr.ENSXETG00000019661: VNRFSKEIDTIDNTIPQIIKMFMGSLFNVIGACVIILTATPIVAVIIPPLGLVYFFVQRFYVATSRQLKRLESVSRSPVYSHFNETLLGSSVIRAFGEQKRFIQISDFKVDENQRAYYPS : 1062
Aca.ENSACAG00000005349: VNRFSKEIDTIDSMIPQIIKMFMGSLFNVVGACVVILLATPLAAVAIPPLALVYFFVQRFYVATSRQLKRLESVSRSPVYSHFNETLLGVSVIRAFAEQQRFVRQSDLKVDQNQKAYYPS : 1075
Gga.ENSGALG00000006646: VNRFSKEIDTIDSTIPPIIKMFMGSTFNVIGACIIILLATPIAAVVIPPLGLVYLLVQRFYVATSRQLKRLESVSRSPVYSHFNETLLGVSVIRAFEEQKRFIKQNDMKVDENQKAYYPS : 1078
Dno.ENSDNOG00000014990: VNRFSKELDTVDSMIPQVIKMFMGSLFNVIGACVIILLATPMAAVIIPPLGLIYFLVQRFYVASSRQLKRLESVSRSPVYSHFNETLLGVSVIRAFEEQERFIRQSDLKVDENQKAYYPS : 1078
Mmu.ENSMUSG00000023088: VNRFSKELDTVDSMIPQVIKMFMGSLFSVIGAVIIILLATPIAAVIIPPLGLVYFFVQRFYVASSRQLKRLESVSRSPVYSHFNETLLGVSVIRAFEEQERFIHQSDLKVDENQKAYYPS : 1078
Ggo.ENSGGOG00000004324: VNRFSKELDTVDSMIPEVIKMFMGSLFNVIGACIVILLATPIAAIIIPPLGLIYFFVQRFYVASSRQLKRLESVSRSPVYSHFSETLLGVSVIRAFEEQERFIHQSDLKVDENQKAYYPS : 1008
Hsa_ENSG00000103222: VNRFSKELDTVDSMIPEVIKMFMGSLFNVIGACIVILLATPIAAIIIPPLGLIYFFVQRFYVASSRQLKRLESVSRSPVYSHFNETLLGVSVIRAFEEQERFIHQSDLKVDENQKAYYPS : 1078
Ptr.ENSPTRG00000007812: VNRFSKELDTVDSMIPEVIKMFMGSLFNVIGACIVILLATPIAAIIIPPLGLIYFFVQRFYVASSRQLKRLESVSRSPVYSHFNETLLGVSVIRAFEEQERFIHQSDLKVDENQKAYYPS : 1032
Cluf.ENSCAFG00000018208: VNRFSKELDTVDSMIPQVIKMFMGSLFNVIGACIIILLATPIASIIIPPLGLIYFFVQRFYVASSRQLKRLESVSRSPVYSHFNETLLGVSVIRAFEEQERFIRQSDLKVDENQKAYYPS : 1078
Bta.ENSBTAG00000021090: VNRFSKELDTVDSMIPQVIKMFMGSLFNVIGACIIILLATPMAAVIIPPLGLIYFFVQRFYVASSRQLKRLESVSRSPVYSHFNETLLGVSVIRAFEEQERFIRQSDLKVDENQKAYYPS : 1078
Mdo.ENSMODG00000004194: VNRFSKEMDTVDSMIPQIIKMFMGSLFNVIGACIIILLATPIAAIIIPPLGLIYFFVQRFYVASSRQLKRLESVSRSPVYSHFNETLLGVSVIRAFEEQQRFIRQSDLKVDENQKAYYPS : 1078
Oan.ENSOANG00000005124: VNRFAKELDTVDSMIPQIIKMFMSSLFNVVGACIIILLATPIAAVVIPPLGLIYFFVQRFYVTSSRQLKRLESVSRSPVYSHFNETLLGVSVIRAFEEQKRFIQQSDMKVDENQKAYYPS : 867
Bfl.232174: INRFSQDMNIVDNVIPRIIRMWLYRVFGVLNALIVISFSTPVFLATILPLALPYYFIQRFYLATSRQLKRIESISRSPIYSHFGETVQGTSTIRAYGRGQQFFFQNQAKVDENHMAYYPT : 1079
Bfl.118638: INRFSQDMNIVDNVIPRIIRMWLYRVFGVLNALIVISFSTPVFLATILPLALPYYFIQRFYLATSRQLKRIESISRSPIYSHFGETVQGTSTIRAYGRHRQFFFQNQAKVDENHMAYYPT : 942
Bfl.118636: INRFSQDVNIVDTVIPMIIRMWLSCLFWVLSTLFVISFSTPLFLAIIVPLALLYYFVQRFYVATSRQLKRIESISRSPIYSHFGETVQGTSTIRAYDRGEQFFFQNQAKVDENQVAYYPM : 1066
Bfl.128060: INRFSQDVNIVDTVIPMIIRMWLSCLFWVLSTLFVISFSTPLFLAIIVPLALLYYFVQRFYVATSRQLKRIESISRSPIYSHFGETVQGTSTIRAYDRGEQFFFQNQAKVDENQVAYYPM : 1066
Dre.ENSDARG00000096662: LNRFSKDVDTIDSLIPDNIDIWMRTFWYTVNVLMVCSVLTPIFLIVIVPLMLFYWWVQRFYVATSRQLKRLESVSRSPIYSHFSESITGTSVIRAYGRNAAFVLMSDNKVDENQKSYYPG : 1076
Ame.ENSAMXG00000016253: INRFSKDIYVIDEALPSTILMFLGTFFASVATMIVIVSSTPIFAVVIVPLALVYFFVQRFYVATSRQLKRLESVSRSPIYSHFSETITGTSVIRAYGRNSAFVLMSDMKVDENQKSYYPG : 1079
Ola.ENSORLG00000020741: INRFSKDVYVIDEALPSTVLMFLGTFCASLSTMIVIVCSTPYFALIIPVLALIYVFVQRFYVASSRQLKRLESVSRSPIYSHFSETVTGSSVIRAYGRLDAFVLMSDAKVDENQRSYYPG : 1077
Gmo.ENSGMOG00000010029: INRFSKDIYIIDEVLPSTVLMFLGTLFSSVSTMIVIVASTPIFAVVIVPLAFVYVFVQRFYVATSRQLKRLESVSRSPIYSHFSETITGASVIRAYGKDSAFVHMSDTRVDDNQKSYYPG : 1034
Xma.ENSXMAG00000012203: INRFSKDIYVIDEALPSTVLMFLGTFFVSLSTMIVIVSSTPIFAVVIAPLAFIYVLVQRFYVATSRQLKRLESVSRSPIYSHFSETITGASVIRAYDRHAAFVLMSDIKVDENQKSYYPG : 1078
Gac.ENSGACG00000005901: INRFSKDIYIIDEALPSTVLMFLATFFVSLSTMIVIVSSTPIFAVVIAPLAFIYIFVQRFYVATSRQLKRLESVSRSPIYSHFSETVTGSSVIRAYGRHSAFVMMSDMKVDENQKSYYPG : 1072
Oni.ENSONIG00000019586: INRFSKDIYVIDEALPSTVLMFLGTFFVSLSTILVIVSSTPIFAVVIVPLAVIYVFVQRFYVATSRQLKRLESVSRSPIYSHFSETITGCSVIRAYGRHSAFVLMSDMKVDENQKSYYPG : 1075
Dla.DLAgn_00098120: INRFSKDIYVIDEALPATVLMLLGTAFMSLSTMIVIVSSTPIFLVVIVPLALIYIFVQRFYVATSRQLKRLESVSRSPIYSHFSETVTGCSVIRAYGRHSAFVLMSDMRVDDNQKSYYPG : 1080
Cmi.SINCAMG00000013336: INRFSKDVNVIDEVIPSTFLMFLATFFTSISTMIVIVCSTPWFALVIGPLAVVYFLVQRFYVATSRQLKRLESVSRSPVYSHFSETISGSSVVRAYGRGASFIHMSDVKVDTNQKSYFPG : 980
Ler.ctg12190: INRFGKDINVIDEVIPLTFQMFLATFFISLSTMIVIMVSTPWFILVILPLAMLYVLVQ-------------------------------------------------------------- : 917
Sca.ctg14163: INRFGKDINVIDEVIPLTFQMFLMTFFVSISTMIVVTASTPWFSLLIVPLAFVYFLVQRFYVATSRQLKRLESVSRSPIYSHFSETITGCSVIRAYGRQSSFIRMN-------------- : 905
Rty.XP_020375725_1: INRFGKDVNVIDEVIPLTFQMFLATFFISLSTTIVVVSSTPWFTLLIVPLAFVYFFVQRFYVATSRQLKRLESVSRSPIYSHFSETITGSSVIRAYGRQNSFILMNDNKVDANQKSYYPG : 499
Sac_EU250283: INRFGKDVHVIDEVIPLTFQMFLSTFFNSL-TMIVIMASTPWFTLLILPLLFVYFFVQRFYVATSRQLKRLESVSRSPIYSHFSETITGSSVIRAYGKEKSFILMNDTKVDANQKSYYPG : 1079
Tni.ENSTNIG00000004171: INRFSKDIYVIDEALPATVLMLLGTVFVSLSTIIVIVSSTPIFLVVIVPLAFIYVFVQRFYVATSRQLKRLESVSRSPIYSHFSETVTGCSVIRAYGRRSAFVLMSDKKVDDNQKSYYPG : 1029
Aca.ENSACAG00000001396: INRFSKDIYVIDEVIPPTILMFLGTFFTSLSTMLVIIASTPLFAVVIIPLAILYFFAQRFYVATSRQLKRLESVSRSPIYSHFSETVTGASVIRAYRREKSFVYISDAKVNDNQKSYYPG : 1074
Gga.ENSGALG00000007522: INRFSKDIFVIDEVIPPTILMFLGTFFASLSTMIVIVASTPLFAVVVVPLAVLYYFVQRFYVATSRQLKRLESVSRSPIYSHFSETISGTSVIRAYQRERSFIDISDLKVDENQKSYYPG : 1025
Xtr.ENSXETG00000012239: INRFSKDIYVIDEVIPGTILMFLATFFTSLSTMIVIVASTPLFAVVIIPLAIAYIFVQRFYVATSRQLKRLESVSRSPIYSHFSETITGASIIRAYGRQNSFIVLSDNKVDENQKSYYPG : 1077
Loc.ENSLOCG00000010918: LNRFSKDIYVIDELIPPTILMFLATLFVSLSTMIVIMATTPIFAVVIVPLALVYVFVQRFYVATSRQLKRLESVSRSPIYSHFSETITGTSVIRAYDRHKAFVLISDEKVDENQKSYYPG : 1077
Lch.ENSLACG7209/6619: INRFSKDIFVIDEVLPSTILMFLGTFFSSLSVMIVIICSTPIFIVVIIPLAALYFFVQRFYVATSRQLKRLESVSRSPIYSHFSETITGSSVIRAYGRQTSFIGISDKKVDENQKCYYPS : 1060
Mdo.ENSMODG00000020910: ------------------------------------------------------------------------------------------------------------------------ : -
Dno.ENSDNOG00000046300: LNRFSKDIYVMDEVLMPIMLMLLNAACNSVSSLAVILISTPLFSVVTLPLVALYILVQHYYVRTSRQLKRLESVSRSPIYSHFSETVTGASVIRAYGHCQDFEAICDVKLDTNQQSCYLY : 1078
Mmu.ENSMUSG00000020865: LNRFSKDIYVIDEVLAPTILMLLNSFFTSISTIMVIVASTPLFMVVVLPLAVLYGFVQRFYVATSRQLKRLESISRSPIFSHFSETVTGTSVIRAYGRIQDFKVLSDTKVDNNQKSSYPY : 1077
Ggo.ENSGGOP00000003354: LNRFSKDIYVIDEVLAPVILMLLNSFFNAISTLVVIVASTPLFTVVILPLAVLYTLVQRFYAATSRQLKRLESVSRSPIYSHFSETVTGASVIRAYNRSRDFEVISDTKVDANQRSCYPY : 1080
Hsa.ENSG00000108846: LNCFSKDIYVVDEVLAPVILMLLNSFFNAISTLVVIMASTPLFTVVILPLAVLYTLVQRFYAATSRQLKRLESVSRSPIYSHFSETVTGASVIRAYNRSRDFEIISDTKVDANQRSCYPY : 1080
Ptr.ENSPTRG00000009406: LNRFSKDIYVIDEVLAPVILMLLNSFFNAISTLVVIVASTPLFTVVILPLAVLYTLVQRFYAATSRQLKRLESVSRSPIYSHFSETVTGASVIRAYNRSRDFEIISDTKVDANQRSCYPY : 1080
Cluf.ENSCAFG00000017201: LNRFSKDIYVIDEVLAPTILMLLNSFYNSVATLVVIVASTPLFTVVALPLAVFYVLVQRFYVATSRQLKRLESISRSPIYSHFSETVTGSSVIRAYGRSQDFKAISDAKVDANQRSCYPY : 1080
Bta.ENSBTAG00000020070: LNRFSKDIYVIDELLAPTILMLLNSFYNSISTLVVIVASTPLFAVVILPLAVLYLFVQRFYVATSRQLKRLESVSRSPIYSHFSETVTGSSVIRAYGRSQDFETINDAKVDTNQKSCYPY : 1080

Hsa.CFTR.ENSG0000000162: LSTLRWFQMRIEMIFVIFFIAVTFISILTTG--GEGRVGIILTLAMNIMSTLQWAVNSSIDVDSLMRSVSRVFKFIDMPTEGKPTK-STKPYKNWPSGGQMTVKDLTAKYTEGGNAILEN : 999
Hsa.ABCC5.ENSG000001147: TCAMRWLAVRLDLISIALITTTGLMIVLMHGQIPPAYAGLAISYAVQLTGLFQFTVRLASETEARFTSVERINHIKTLSLEAPARIKNKAPSPDWPQEGEVTFENAEMRYRENLPLVLKK : 1082
Hsa.ABCC11.ENSG00000121: LSSTRWMALRLEIMTNLVTLAVALFVAFGISSTPYSFKVMAVNIVLQLASSFQATARIGLETEAQFTAVERILQMKMCVSEAPLHMEGTSCPQGWPQHGEIIFQDYHMKYRDNTPTVLHG : 1065
Hsa.ABCC12.ENSG00000140: AL--RWFALRMDVLMNILTFTVALLVTLSFSSISTSSKGLSLSYIIQLSGLLQVCVRTGTETQAKFTSVELLREISTCVPECTHPLKVGTCPKDWPSRGEITFRDYQMRYRDNTPLVLDS : 1032
Hsa.ABCC8.ENSG000000060: TAANRWLEVRMEYIGACVVLIAAVTSISNSLELSAGLVGLGLTYALMVSNYLNWMVRNLADMELQLGAVKRIHGLLKTEAESYEGLLPSLIPKNWPDQGKIQIQNLSVRYDSSLKPVLKH : 1184
Hsa.ABCC9.ENSG000000694: SAANRWLEVRTDYLGACIVLTASIASISGSS--NSGLVGLGLLYALTITNYLNWVVRNLADLEVQMGAVKKVNSLTMESENYEGTMDPSQVPEHWPQEGEIKIHDLCVRYENNLKPVLKH : 1179
Hsa.ABCC10.ENSG00000124: SATMQWLDIRLQLMGAAVVSAIAGIALVQHQGLNPGLVGLSLSYALSLTGLLSGLVSSFTQTEAMLVSVERLEETCDLPQEPQGQ--PLQLGTGWLTQGGVEFQDVVLAYRPGLPNALDG : 1171
Hsa.ABCC4.ENSG000001252: LTTSRWFAVRLDAICAMFVIIVAFGSLILAKTLDAGQVGLALSYALTLMGMFQWCVRQSAEVENMMISVERVIEYTDLEKEAPWEYQ-KRPPPAWPHEGVIIFDNVNFMYSPGGPLVLKH : 996
Cel.WBGene00003414: HMSNRWLATRLELLGNTCVLFASLSATLSTKGLTPGMAGLSVSYALTITEVLNICVRSVSEIESNIVSVERVNEYQKLEPEAPWRIESLENEEKWPVKGKIELDGFSMRYRKNLPLVLKN : 1151
Cel.WBGene00003409: QMANRWLSIRLELLGNIVIFSAAILAIIGKESGTAGMLGLSVSYSLNITFMLNMFVRQINEVETNVVSVERIDEYSKTKSEAEWRLDNNNLPSNWPTGGAVNIEDYSCRYRDELDLVLKQ : 1187
Hro.HelroG163344: ITSNRLLTARLSWRHNCCV---CSCICCSRKRV--------------FVLFDCWTF-------NNLCAPKRGKR---------------DPPEEWPDRNNIIFRNYGLRYRPGLELVLKN : 1069
Hro.HelroG157076: ITSNRWLNLLLDCMGGVVVLSAAIFAVMDRDVLSSAFVGLSVSSALSMTVTLSWFIEMGSELESAIVSVERVKEYSETPTEADWSKGSFDLPLTWPSKHDIIFHDYGLRYRSGLELVLKN : 830
Bfl.230771: YMTEIWLTTGLELTGNAITLAATLLAVLGRDSLSPGTVGLSISSAMMINGLLNRLVHTTGHLEMNVVSLERLQQYAHTPAEADWIAEDHRPPDQWPTEGNISLSLYKTRYREGLDLVIKD : 1180
Lgi.LotgiG105097: SVISRWLRMRLEVIGNITVLSASLLAVFMPG-VTGSDAGLSITYALQVTAMLNQMVSVSSQLEVNVVSVERIVEYSEKEQEADWYIQDTKPKDTWPDSGEIEITNLLTKYRHDLPLVLKG : 1179
Cel.WBGene00003407: IVANRWLAVRLEMVGNLIVLSSAGAAVYFRDGLSAGLVGLSVSYALNITQTLNWAVRMTSELETNIVAVERINEYTITPTEGNNS--QSLAPKSWPENGEISIKNFSVRYRPGLDLVLHG : 1193
Cel.WBGene00003408: IVANRWLAVRLEMVGNLIVLSAAGAAVYFRDGLSAGLVGLSVSYALNITQTLNWAVRMTSELETNIVSVERIKEYTVTPTEGNNS--RRLAAKSWPEKGEISIKNFSVRYRPGLDLVLHG : 1192
Lgi.LotgiG153611: ISANRWLGIWIEFVSSCVVFTAALFSLLSPD-ITGASMGLSVTYALQITAALKWLVRTISDLETNIVSVERVKEYTDITTEAPLINRHNRPCLTWPDAGHIQFQLYSTRYRPELDLVLRA : 1139
Lgi.LotgiG110718: IASNRWLGLNLDLVSSLIVFSASILAVASSDT-GAGPTGLSISYALQISAAVAWMVRQMSDLESNIVSVERVLEYSKIESEAALINFDNRPSSNWPDHGSITWSNYQVRYRQGLDLVLKG : 1006
Dno.ENSDNOG00000024923: LVADSWVTSAVGLRGKGGTGSQQACVLLGVLGQAAHPLWSSFPTSIEVTQTLQWAVRSWTDLESSIVAVERVQEYARTPKEAPWRLPSCAARPPWPRQGQIEFRCFGLRYRPELPLAVRG : 1134
Mmu.ENSMUSG00000030834: LVADRWLATNLELLGNGLVFVAATCAVLSKAHLSAGLVGFSVSAALQVTQTLQWVVRSWTDLENSMVAVERVQDYARIPKEAPWRLPTCAAQPLWPCGGQIEFRDFGLRHRPELPLAVQG : 1192
Cluf.ENSCAFG00000018197: LVADRWLAANLELLGNMLVLAAAMCAVLSKAHLSAGLVGFSVSAALQVTQTLQWAVRSWTDLASSVVSVERMKDYVQTPKEAPWRLPACAARSPWPHGGQVEFRDFGLRHHPELPLAVRG : 1193
Bta.ENSBTAG00000015191: LVADRWLAANLELVGNGLVFVAALCAVLSKAHLSPGLVGFSVSAALQVTQMLQWAVRSWTDLESSIVSVERLKDYAQTPKEAPWKPLTCAAHPPWPRRGQIEFRDLGLRYRPELPLAVRG : 1194
Ggo.ENSGGOG00000009623: LVADRWLAANVELLGNGLVFAAATCAVLSKAHLSAGLVGFSVSAALQVTQTLQWVVRNWTDLENSIVSVERMQDYAWTPKEAPWRLPTCAAQPPWPHGGQIEFRDFGLRYRPELPLAVQG : 1185
Hsa.ENSG00000091262: LVADRWLAANVELLGNGLVFAAATCAVLSKAHLSAGLVGFSVSAALQVTQTLQWVVRNWTDLENSIVSVERMQDYAWTPKEAPWRLPTCAAQPPWPQGGQIEFRDFGLRYRPELPLAVQG : 1193
Ptr.ENSPTRG00000007815: LVADRWLAANVELLGNGLVFAAATCAVLSKAHLSAGLVGFSVSAALQVTQTLQWVVRNWTDLENSIVSVERMQDYAWTPKEASWRLPTCAAQPPWPHGGQIEFRDFGLRYRPELPLAVQG : 1050
Oan.ENSOANG00000005123: LVADRWLATNLELLGNGVVLSAAIFAVMGRAHLSPGIVAFSVTTSLQVTEILHWVVRSWTSLENNIVSVERVMEYSRTPKEAPWTVASSPLPKTWPDAGRIEFRNYGLRYRAGLALALRD : 1148
Mdo.ENSMODG00000005815: LVADRWLATNMELLGNVLIFAAAFFAVLSKPYLRPGIVGFSVSVALQVTEILHWAVRSWTDLENNIVSVERMRDYTRTPKEAPWTLSSNGVSHTWPVMGQIEFRGYSLRYRPELALALQN : 1193
Gga.ENSGALG00000006698: AVADRWLATNLEFLGNGIVLFAALFATIGRTHLSPGTAGFSISYALQITGVLNWMVRSWTEIENNIVSVERVSEYSRTPKEAPWTLNDKLQGQVWLTEGRIEFRNYSLRYRPNLELALKH : 1196
Aca.ENSACAG00000003478: VVADRWLATNIEFLGNGIVLFAALLAVKSKPYLSPGLVGFSISYALQITGILNWMVRALAEIDNNIVSVERVRDYSGTPKEAPWTSDNKFFHENWPTEGQIAFRGYSLRYRPGLELALKN : 1136
Cel.WBGene00003413: LVSNRWLAVRLEFVGNCIIFFAALFAVLSKEWISPGVIGVSVSYALNITEVLNFAVRQVSEIEANIVSVERVNEYTNTPNEAPWRIEGREPAPGWPSRGVVKFDGYSTRYREGLDLVLHD : 1192
Aga.AGAP008437: IIANRWLAVRLELVGALVVFFAALFAMVARDTIGQATVGLSISYALQISATLSFLVRMTAEVETNIVAIERLEEYTVLPREAEWQL--GHVDKAWPVEGKVEFKDYQIRYREGLDLVIRG : 1189
Aga.AGAP027980: ILANRWLGVRLEIIGSLVILFAALFAILARDTIGQATVGLSISYALQISNVLSFLVRMTAEVETNIVAIERLEEYTVLPREAEWQ--KGTVDKAWPVEGKVEFKDYQIRYREGLDLVIRG : 1191
Aga.AGAP028128: IVANRWLAVRLELIGSCVILFAALFAILARDTIGQATVGVSISYALQISHYLSFLVRMTSEVETNIVAVERLEEYTVLPREAEWQ--KGTVDKAWPVEGKVEFKDYQIRYREGLDLVIRG : 1188
Spu.026395: ------------------------------------------------------------------------------------------------------------------------ : -
Hsa.ABCC2.ENSG000000238: ITSNRWLAIRLELVGNLTVFFSALMMVIYRDTLSGDTVGFVLSNALNITQTLNWLVRMTSEIETNIVAVERITEYTKVENEAPWVT-DKRPPPDWPSKGKIQFNNYQVRYRPELDLVLRG : 1188
Xtr.ENSXETG00000026360: FVANRWLSVRCDFLSNFIVFTVAIVGVLFRDNITPGLVGLAVVNSLRLTGVLKEAVHVATDMETNSVSVERVKEYCDAEPEAPWTSDNASDPSNWPSKGKIEFQNYGLRYRPDLDLALKN : 1179
Cel.WBGene00003410: YVANRWLSVRLEFIGNCIVLFSALFAALTRTTTTSGVIGLSVSYALNITTVLNFAVRQITKLETNIVSVERVKEYAETETEAEWKSEGKEPPQNWPSEGRIVMNNYSARYRPGLNLVVKQ : 1192
Ame.GB53134: ------------------------------------------------------------------------------------------------------------------------ : -
Pma.KM232931_1: IVSNRWLAVRLEILANLIVFFATLFAVVSRHSLSPGIVGLSVSYALQVTLTLNWLVRMTSELETNIVAVERLKEYSDLPTEAAWEIAATRPPATWPHCGDLEFVDYSLRYRDGLELALNQ : 910
Gac.ENSGACG00000003037: FVATRWLAVNLEFLGNLLVLAAAVLSVRGRDHLSPGIVGLAVTHSLQVTGILSWIVRSWTDVENNIVSVERVKEYDSTDKEGGWVPGGNKLPADWPATGNLQFEGYGLRYRKDLDWALNN : 1199
Loc.ENSLOCG00000007152: FVATRWLAVNLEFLGNALVLAAAILSVIGKGTLSPGIVGLAVSHSLQVTGILSWIVRSWTDVENNIVSVERVKEYVETAKEAAWTVESSPVPPAWPQTGTIELRGYGLQYRKGLDWALKG : 1195
Gmo.ENSGMOG00000005748: VAT-RWLAVNLEFVGNGVVLAAAILSVMGKHTLSPGIVGLAVSHSLQVTGILSWIVRSWTDVENNIVSVERVKEYADTAKEAPWTVEGSSLPPDWPQRGTIEFQDYGLQYRKGLDLALKG : 1157
Gac.ENSGACG00000019172: FVATRWLAVNLEFVGNGVVLAAAILSVMGKGTLSPGIVGLAVSHSLQVTGILSWIVRSWTDVENNIVSVERVNEYADTPKEASWSIEGSSLPPDWPQRGTIEFQDYGLQYRKGLELALKG : 1200
Tni.ENSTNIG00000012067: FVATRWLAVNLEFIGNGVVLAAAILSVMGRNTLSPGIVGLAVSHSLQVTAILSWIVRSWTDVENNIVSVERVNEYADTAKEASWTVEGSSLPMDWPLKGTLEFQEYGLQYRKGLELALKG : 1197
Ola.ENSORLG00000013429: FVATRWLAVNLEFVGNGVVLAAAVLSVIGKSTVSPGIVGLAVSHSLQVTGILSWIVRSWTDVENNIVSVERVNEYADTPKEASWNTEGSALPLAWPQSGTIEFQDYGLQYRKGLELALKG : 1199
Oni.ENSONIG00000018866: FVATRWLAVNLEFVGNGVVLAAAILSVMGKSTLSPGIVGLAVSHSLQVTGILSWIVRSWTDVENNIVSVERVNEYADTPKEASWSIESSSLPQAWPQNGTIEFQDYGLQYRKGLELALKG : 1199
Xma.ENSXMAG00000004906: FVATRWLAVNLEFVGNVVVLAAAILSVMGRSTLSPGIVGLAVSHSLQVTGILSWIVRSWTDVENNIVSVERVNEYADTPKEASWSTEGSSLPVAWPQSGTIELQDYGLQYRKGLELALKD : 1195
Dla.DLAgn_00177430: FVATRWLAVNLEFVGNGVVLAAAILSVMGKNTLSPGIVGLAVSHSLQVTGILSWIVRSWTDVENNIVSVERVNEYADTAKEASWSVEGSSLPLAWPQRGTLEFQDYGLQYRKGLELALKG : 1195
Dre.ENSDARG00000016750: FIATRWLGVNLEFLGNGIVLAASILSVMAKGTLSPGMVGLAVSHSLQVTGFLSWIVRSWTDVENNIVSVERVKEYADTPKEAAWSIEGSSLPPSWPQTGTIEFQDYGLQYRKGLELALKG : 1195
Ame.ENSAMXG00000004837: FVATRWLAVNLEFLGNGVVLAAAILSVMGKETLSPGIVGLAVSHSLQVTAILSWIVRSWTDVENNIVSVERVKEYEETPKEAAWTSEGNSLPPTWPQVGTIEFQDYGLQYRKGLEFALKG : 1192
Ame.ENSAMXG00000003085: FVATRWLAVNLEFLGNLLVLAAAILSVTGKDTLSPGIVGLAVSHSLQVTGILSWIVRAWTDVENNIVSVERVKEYAETPKEAPWTIENRSLPSAWPQTGSIEFQQYGLQYRRGLDWALKE : 1200
Dre.ENSDARG00000094901: FVASRWLAVNLEFLGNLLVLAAAILSVMGRATLSPGTVGLAVSHSLQVTGILSWIVRSWTDVENNIVSVERVKEYAETAKEAPWTFEDSPLPSDWPRSGSIGFQAYGLQYRKGLDWALKE : 1167
Dre.ENSDARG00000095820: FVATRWLAVNLEFLGNLLVLAAAILSVMGRATLSPGIVGLAVSHSLQVTGILSWIVRSWTDVENNIVSVERVKEYAETAKEAPWTIEDSPLPSDWPRCGSIGFQAYGLQYRKGLDWALKE : 1200
Lch.ENSLACG00000022117: VVANRWLAVNLEFLANVIVLFAAILAVNGKGRLSPGVVGLSVSHALQVTGILSWIVRSWTDIENNIVSVERVKEYSETPKEDPWILNTNFLPEPWPSEGRVEFRNYGLRYRQDLDLAVKN : 1200
Dpu.347281: IVANRWLSIRLETIGNLVVLFASLFAVIERETMDPGYVGLSITYALSITQTLNWFMRMTSEVETNIVAVERIKEYSEAVQEASWDHGKREPPNSWPDKGKVSFEKYEVRYREGLDLVIKG : 1199
Lca.KE993868_1: IVSQ-------------------------------------------------------------------------------------------------------------------- : 466
Pma.KM232930_1: IVSQRWLAVWLEFLGACVVLFAAMFAVLSRDSLKAGTV-LSVSYAMQVTVVMSWMVRMSSEVESNIVAVERVKEYSETPPEADWEVESCRPPADWPSQGHVEFRDYSTRYREGLDLVLRN : 1199
Csa.ENSCSAVG00000008135: IVSNRWLALRLELVGNFIVLFAAIFAVAGKGTIDSGIVGLSVSYAMQITQTLNWMVRQSSELETNIVAVERVEEYSNVVQEAPLVIEGERPNKDWPSVGEIKFEDYSTRYRSELDLVVKN : 1191
Csa.ENSCSAVG00000003792: IVSNRWLALRLELVGNLIVLFAAIFAVVEKGKINDGIVGLSISYSLQITMMLNWLVRQASELETNIVAVERVEEYSSVVQEAELVIDGSRPPSHWPAEGSIKFEDYSTRYREGLDLVVKN : 1132
Cin.ENSCING00000020698: ------------------------------------------------------------------------------------------------------------------------ : -
Tca.TC012253: IISNRWLAVRLEMIGNLIIFFAALFSVLGRDESNAGLVGLSVTYSLQITQTLNWLVRMTSDVETNIVAVERIKEYGEAPQEAPWEIPNKAPSPQWPENGTVQFNKYAVRYRPGLDLVLKG : 1193
Aga.AGAP009835: IIANRWLAVRLEMVGNLIILFAALFAVLGRETMNAGLVGLSVSYALQITQTLNWLVRMTSDVETNIVAVERIKEYGETKQEAAWELPNSTLPRDWPEQGMVEFRDFQVRYREGLELVLRG : 1189
Lgi.LotgiG107213: ITSNRWLAIRLEFIGNLIVFFASLFAVLGRESLSPGIVGLSISYALNVTQTLNFLVRMTCELETNIVAVERVKEYSETPTEADWIVENNRPDKEWPIKGEVEFSNFQLRYREGLDLVLKG : 1193
Bfl.90918: IVATRWLSMGLDVVSNIIIFFAALFAVVGRESLTPGLVGLSISYALQVTLFLGGVVRVSSQLEASIVAVERLKEYEETNEEADWTSDGKRPPDSWPSEGRIRFNSYQTRYREGLDLVLRD : 1068
Dme.FBgn0032456: VIANRWLAIRLEMVGNLIILFASLFAVLGGQT-NPGLVGLSVSYALQVTQTLNWLVRMSSDIETNIVSVERIKEYGETKQEAPWELEDKNKPKNWPQEGRVEFQNFQVRYREGLDLVLRG : 1192
Oan.ENSOANG00000013379: ------------------------------------------------------------------------------------------------------------------------ : -
Pma.AKC42143_1: ------------------------------------------------------------------------------------------------------------------------ : -
Lca.KE994284_1: ------------------------------------------------------------------------------------------------------------------------ : -
Cmi.SINCAMG00000015787: IVANRWLAVRLEFVGNCIVLFAALFAVAYRLKLSAGLVGLSISYALQVTATLNWLVRMSSEVETNIVAVERVKEYSEMEKEAPWFSNNNSPTSNWIQRGTIQFIGYSARYRADLDLVLKN : 1199
Ler.ctg13956: VVANRWLAVRLELVGNFIVFFAALFAVVSRSDVSPGIVGLSISYALQITATLNWLVRMTSEVETNIVAVERVQQYYDTPKEARWVIKSNRPPQTWPSEGNIEFINYGLRYRTDLDLALKD : 772
Rty.XP_020377569_1: IVANRWLAVRLEFVGNCIVLFAALFAVVYRDTVSPGIVGLSVSYALQVTSTLNWMVRMTSELETNIVAVERVQEYTGTPKEAHWINRSNRPPQTWPLEGKIEFVNYGLRYREDLDLALKD : 651
Sca.ctg67278: IVANRWLAVRLEFVGNCIVLFAALFAVAYRDAVSPGIVGLSISYALQITSTLNWLVRMTSEVETNIVAVERVQEYSVTSKEARWVIHNNRPPKTWPSEGKVEFVNYALRYREDLDFALKD : 1100
Lch.ENSLACG00000001471: ------------------------------------------------------------------------------------------------------------------------ : -
Tni.ENSTNIG00000005013: ------LLYRSCVVGNIIVSFAALCAVIARQNLSPGIMGLSISYALQLTASLTWLVRMSSDLETNIVAVEKVKEYSETQKEAEWTHKPTSLPSNWPNKGCIDIRGFSLRYRDDLDLAIRN : 1088
Loc.ENSLOCG00000007196: IVANRWLAVRLEYVGNCIVLFASLFAVMARDRLSPGSMGLSISYALQITASLNWLVRMSSEMETNIVAVERVKEYGDTEKEAPWQLEKSAPPRGWPTAGRIEIRDFGLRYREDLELALRD : 1200
Dre.ENSDARG00000104719: IVANRWLAVRLEFVGNCIVTFAALFAVMARNNLSPGIMGLSISYALQVTASLNWLVRMSSELETNIVAVERVKEYGDTEKEAEWKLENSNLPPGWPTAGHIEIHKFGLRYREDLELAICD : 1195
Ame.ENSAMXG00000002943: IVANRWLAVRLEFVGNCIVTFAALFAVMARANLSPGIMGLSISYALQVTASLNWLVRMSSELETNIVAVERVKEYEDTEKEAEWKLEQSSVPAGWPTAGHIEVRNFGLRYREDLELAIHD : 1195
Xma.ENSXMAG7738/17319: ------------------------------------------------------------------------------------------------------------------------ : -
Ola.ENSORLG00000017141: IVANRWLAVRLEFVGNCIVSFAALFAVLARESLSPGIMGLSISYALQLTASLTWLVRMSSDVETNIVAVERVKEYSDTEKEAEWRHEPPTVPPDWPTEGCIRITNFGLRYRSDLDLAIRN : 1197
Gac.ENSGACG00000000434: IVANRWLAVRLEFVGNCIVSFAALFAVVARESLSPGIMGLSISYALQLTASLTWLVRMSSEVETNIVAVERVKEYSDTEKEAEWKQEPSRLPPGWPTDGCIDIRGLGLRYRPDLDLAIRN : 1200
Oni.ENSONIG00000007824: IVANRWLAIRLEFVGNCIVSFAALFAVVARQSLSPGIMGLSISYALQLTTSLTWLVRMSSDVETNIVAVEKVKEYSDTEKEAAWEHEPSTLSPGWPTNGCIEMRSFGLRYRQDLDLAIRN : 1200
Dla.DLA_00195360: ------------------------------------------------------------------------------------------------------------------------ : -
Xtr.ENSXETG00000019661: IVSNRWLAIRLEFVGNCIVLFASLFAVISRTTLSPGLVGLSVSYALQVTTYLNWLVRMSSELETNIVAVERVKEYADLKQEAAWTVQETAPEATWPHEGKIEFRGYGLRYREDLDLALKN : 1182
Aca.ENSACAG00000005349: IVANRWLAVRLESVGNCIVLFAALFAVIARHVLSPGLVGLSISYSLQITTYLNWLVRMSAEMETNIVAVERVKEYSEKEQEAEWRLPGAPIPEGWPQEGRVEFRGYSLRYRDDMDLVLRN : 1195
Gga.ENSGALG00000006646: IVANRWLAVRLEFVGNCIVLFAALFAVIARNKLSPGLIGLSVSYSLQITAYLNWLVRMTSDLETNIVAVERVKEYAEMEKEAEWSIDETAPASTWPQEGKVEFRGFGLRYREDLDLVLKN : 1198
Dno.ENSDNOG00000014990: IVANRWLAVRLECVGNCIVLFAALFAVVSRHSLSAGLVGLSVSYSLQVTAYLNWLVRMSSEMETNIVAVERLKEYSETEKEVSFKVKQKKVPGPEPRTSYMGSIEYILMYKYTLCVFVEH : 1198
Mmu.ENSMUSG00000023088: IVANRWLAVRLECVGNCIVLFAALFAVISRHSLSAGLVGLSVSYSLQITAYLNWLVRMSSEMETNIVAVERLKEYSETEKEAPWQIQETAPPSTWPHSGRVEFRDYCLRYREDLDLVLKH : 1198
Ggo.ENSGGOG00000004324: IVANRWLAVRLECVGNCIVLFAALFAVISRHSLSAGLVGLSVSYSLQVTTYLNWLVRMSSEMETNIVAVERLKEYSETEKEAPWQIQETAPPSSWPQVGRVEFRNYCLRYREDLDFVLRH : 1128
Hsa_ENSG00000103222: IVANRWLAVRLECVGNCIVLFAALFAVISRHSLSAGLVGLSVSYSLQVTTYLNWLVRMSSEMETNIVAVERLKEYSETEKEAPWQIQETAPPSSWPQVGRVEFRNYCLRYREDLDFVLRH : 1198
Ptr.ENSPTRG00000007812: IVANRWLAVRLECVGNCIVLFAALFAVISRHSLSAGLVGLSVSYSLQVTTYLNWLVRMSSEMETNIVAVERLKEYSETEKEAPWQIQETAPPSSWPQVGRVEFRNYCLRYREDLDFVLRH : 1152
Cluf.ENSCAFG00000018208: IVANRWLAVRLECVGNCIVLFAALFSVISRHSLSAGLVGLSVSYSLQVTTYLNWLVRMSSEMETNIVAVERLKEYSETEKEAPWQIQEMAPPSTWPQVGRVEFRDYGLRYRENLDLVLKH : 1198
Bta.ENSBTAG00000021090: IVANRWLAVRLECVGNCIVLFASLFAVISRHSLSAGLVGLSVSYSLQVTTYLNWLVRMSSEMETNIVAVERLKEYSETEKEAPWQIQDMAPPKDWPQVGRVEFRDYGLRYREDLDLVLKH : 1198
Mdo.ENSMODG00000004194: IVANRWLAVRLECVGNCIVLFAALFSVISRHSLSPGLVGLSVSYSLQVTTYLNWLVRMSSEMETNIVAVERLKEYSETEKEAPWCIEEAAPPTNWPQQGRVEFRDFSLRYREDLDLVLKH : 1198
Oan.ENSOANG00000005124: IVANRWLAVRLECVGNCIVLFAALFAVISRHSLSPGLVGLSVSYSLQVTAYLNWLVRMSSEMETNVVAVERLKEYSETEKEAPWQIEETAPAPDWPQEGKVEFRDFGLRYREDLDLVLKN : 987
Bfl.232174: IVSNRWLALRLEFLGNCIVLFAGLFAVIGRENLSPGIVGLSITYALQITQTLAWVVRQTSEVETKIVAVERIKEYTETPTEADWVVDDNRPPDNWPSEGKVNFNSYQTRYREGLDLVIKG : 1199
Bfl.118638: IVSNRWLALRLEFLGNCIVLFAGLFAVIGRENLSPGIVGLSITYALQITQTLAWVVRQTSEVETKIVAVERIKEYTETPTEADWVVDDNRPPDNWPSEGKVNFNSYQTRYREGLDLVIKG : 1062
Bfl.118636: IVSNRWLALRLEFVGNCIVLFAALFAVIGRETLSPGIVGLSITYALQITQTLNWMVRMTSELETNIVAVERIKEYAETPTEAEWVVDDNRPPDNWPSEGKVNFNSYQTRYREGLDLVIKG : 1186
Bfl.128060: IVSNRWLALRLEFVGNCIVLFAALFAVIGRETLSPGIVGLSITYALQITQTLNWMVRMTSELETNIVAVERIKEYAETPTEADWVVDDNRPPDNWPSEGKVNFNSYQTRYREGLDLVIKG : 1186
Dre.ENSDARG00000096662: IVSNRWLGVRIEFIGNCIVLFAALFAVTGKDKLSPGLVGLSVSYALQVTMSLNWMVRMTSDLESNIVAVERVKEYSETQTEAPWEVLEKKPPPDWPPLGNVEFVDYSVRYREGLDLVLKN : 1196
Ame.ENSAMXG00000016253: IVSNRWLGIRIEFIGNCIVLFAAVFAVLEKENLSPGLVGLSVSYALQVTMSLNWMVRMTSDLESNIVAVERVKEYSETPPEAPWEVEDKKPPTDWPSEGNVEFADYSVRYREGLDLVLRN : 1199
Ola.ENSORLG00000020741: IVSNRWLGVRIEFIGNCIVLFAALFAVIWKETLNPGLVGLSVSYALQVTMSLNWMVRMTSDLENNIVAVERVKEYSETKPEAPWEVEDKKPPPEWPTDGKVEFHGYSVRYRDGLDLVLKN : 1197
Gmo.ENSGMOG00000010029: IVANRWLGVRIEFIGNCIVLFAALFAVIGKEKLNPGLVGLSVSYALQVTMTLNWMVRMTSDLESNIVAVERVKE-------APWVVEDKRPPSDWPPQGNVEFRDYSVRYREGLDLVLKN : 1147
Xma.ENSXMAG00000012203: IVSNRWLGVRIEFIGNCIVLFAALFAVMGKDTLSPGLVGLSVSYALQVTMSLNWMVRMTSDLENNIVAVERVKEYSETKTEAPWEVEDKKPPAEWPTQGNVEFNEYSVRYREGLDLVLRN : 1198
Gac.ENSGACG00000005901: IVSNRWLGVRIEFIGNCMVLFAALFAVTGRENLNPGLVGLSVSYALQVTMSLNWMVRMTSDLENNIVAVERVKECCLLCSQAPWEVEDKKPPPDWPTEGNVEFQDYSVRYREGLDLVLKN : 1192
Oni.ENSONIG00000019586: IVSNRWLGVRIEFIGNCIVLFAGLFAVTGKDSLSPGLVGLSVSYALQVTMSLNWMVRMTSELENNIVAVERVKEYSETKTEAPWEVEDKKPPLEWPMQGNVEFNDYSVRYREGLDLVLKD : 1195
Dla.DLAgn_00098120: IVSNRWLGVRIEFIGNCIVLFAALFAVTGKENLNPGLVGLSVSYALQVTMSLNWMVRMTSDLENNIVAVERVKEYSETKTEAPWEVEDKKPPPEWPMEGNVAFQDYSVRYREGLDLVLKK : 1200
Cmi.SINCAMG00000013336: IVANRWLGIRIEFIGNCIVLFAALFAVIGRDHLKPGIVGLSVSYALQVTMSLNWMVRMTSDLESNIVAVERIKEYSETVNEAPWVIDDKRPDPDWPQTGSVEFRGYSVRYREGLDLVLRD : 1100
Ler.ctg12190: ------------------------------------------------------------------------------------------------------------------------ : -
Sca.ctg14163: ----------------------------------------------QVTMSLNWMVRMTSDLESNIVAVERVKEYSETKTEAPWVIENNRPSQSWPEAGNVEFHGYSVRYREGLDLVLKN : 979
Rty.XP_020375725_1: IVSNRWLGIRIEFIGNCIVLFAALFAVIGRAHLNPGIVGLSVSYALQVTMSLNWMVRMTSDLESNIVAVERVKEYSETETEAPWVIESNRPPESWPQAGNVEFKGYSVRYREGLDLVLKG : 619
Sac_EU250283: IVSNRWLGIRIEFIGNCIVLFAALFAVIGRHDLDPGIVGLSVSYALQVTMSLNWMVRMTSDLESNIVAVERVKEYSETETEAPWVIESNRPPKSWPETGNVEFNGYSVRYREGLDLVLKD : 1199
Tni.ENSTNIG00000004171: IVSNRWLGVRIEFIGNCVVLFAALFAVTGKDSLSPGLVGLSVSYALQVTMSLNWMVRMSSDLENNIVAVERVKEYSETKTEAPWVVEDKRPPPEWPMEGNVEFHDYSVRYREGLELVLKE : 1149
Aca.ENSACAG00000001396: IVANRWLGIRVEFVGNCVVFFAALFAVLSRNKLSAGVVGLSVSYALQVTMALNWMVRMSSDLESNIVAVERVKEYSETETEAPWIIEDKRPSENWPDQGEVQFANYSVRYRKGLDLVLKN : 1194
Gga.ENSGALG00000007522: IISNRWLGIRVEFVGNCIVLFAALFAVIGKSSLNAGLVGLSVSYALQVTMALNWMVRTTSDLETNIVAVERIKEYSETETEAPWIIEDKRPPADWPSRGELEFVGYSVRYRKGLDLVLKD : 1145
Xtr.ENSXETG00000012239: IVSNRWLGVRVEFVGNCVVLFAALFAVLGREHLSPGLVGLSVSYALQVTMSLNWMVRMTSDLETNIVAVERVKEYAENETEAPWHIEDTKPPEDWPSKGEVELSNYSVRYRAGLDLVLKN : 1197
Loc.ENSLOCG00000010918: IVSNRWLGVRIEFIGNCIVLFAALFAVIGKSTLSPGLVGLSVSYALQVTMSLNWLVRMSSDLESNIVAVERVKEYSETKTEAPWVIEDKRPPPDWPSQGTVEFHKYSVRYREGLDLVLKN : 1197
Lch.ENSLACG7209/6619: IVANRWLGVRIEFIGTCIVLFAALFAVIGRKNLNPGLVGFSLSYALQVTMSLNWMVRMTSDLETNIVAVERVKEYSETKTEAPWIIPEKRPPKDWPIEGQVDFVNYSVRYRDGLDLVLKN : 1180
Mdo.ENSMODG00000020910: ------------------------------------------------------------------------------------------------------------------------ : -
Dno.ENSDNOG00000046300: NVSNRWPGIRVEFLGSCVVLFAALFTVLGRGSLTPGLVGLSVTYALKVTSNLTWTVRMMLDLESNIVAVERVKEYTKTETEAPWLIEGSRPPERW-------FRNFSVRYRPGLELVLKD : 1191
Mmu.ENSMUSG00000020865: IASNRWLGVHVEFVGNCVVLFAALFAVIGRNSLNPGLVGLSVSYALQVTMALNWMIRMISDLESNIIAVERVKEYSKTKTEAPWVVESNRAPEGWPTRGMVEFRNYSVRYRPGLELVLKN : 1197
Ggo.ENSGGOP00000003354: IISNRWLSIGVEFVGNCVVLFAALFAVIGRSSLNPGLVGLSVSYSLQVTFALNWMIRMMSDLESNIVAVERVKEYSKTETEAPWVVEGSRPPEGWPPRGEVEFRNYSVRYRPGLDLVLRD : 1200
Hsa.ENSG00000108846: IISNRWLSIGVEFVGNCVVLFAALFAVIGRSSLNPGLVGLSVSYSLQVTFALNWMIRMMSDLESNIVAVERVKEYSKTETEAPWVVEGSRPPEGWPPRGEVEFRNYSVRYRPGLDLVLRD : 1200
Ptr.ENSPTRG00000009406: IISNRWLSIGVEFVGNCVVLFAALFAVIGRSSLNPGLVGLSVSYSLQVTFALNWMIRMMSDLESNIVAVERVKEYSKTETE--------------------------------------- : 1161
Cluf.ENSCAFG00000017201: IASNRWLGIRVEFVGNCVVLFAALFAVIGRNSLSPGLVGLSVSYALQITLTLNWMIRMMSDLESNIVAVERVKEYSKTETEAPWVVEGSRPPAGWPLQGEVEFRNYSVRYRPGLELVLKK : 1200
Bta.ENSBTAG00000020070: IASNRWLGIRVEFVGNCVVLFAALFAVTGRSSLSPGLVGLSVSYALQVTLALNWMIRTISDLESNIVAVERVKEYSKTEMEAPWVVEGSRPPAGWPLKGEVEFRNYSVRYRPGLELVLKD : 1200

Hsa.CFTR.ENSG0000000162: ISFSISPGQVGLLGRTGSGKSTLLSAFLRLLNT-EGEIQIDGVSWDSITLQQWRKAFGVIPKVFIFSGTFRKNLDPYEQWSDQEIWKVADEVGLRSVIEQFPGKLDFVLVDGGCVLSHGH : 1118
Hsa.ABCC5.ENSG000001147: VSFTIKPKEIGIVGRTGSGKSSLGMALFRLVELSGGCIKIDGVRISDIGLADLRSKLSIIPEPVLFSGTVRSNLDPFNQYTEDQIWDALERTHMKECIAQLPLKLESEVMENGDNFSVGE : 1202
Hsa.ABCC11.ENSG00000121: INLTIRGHEVGIVGRTGSGKSSLGMALFRLVEPMAGRILIDGVDICSIGLEDLRSKLSVIPDPVLLSGTIRFNLDPFDRHTDQQIWDALERTFLTKAISKFPKKLHTDVVENGGNFSVGE : 1185
Hsa.ABCC12.ENSG00000140: LNLNIQSGQVGIVGRTGSGKSSLGMALFRLVEPASGTIFIDEVDICILSLEDLRTKLTVIPDPVLFVGTVRYNLDPFESHTDEMLWQVLERTFMRDTIMKLPEKLQAEVTENGENFSVGE : 1152
Hsa.ABCC8.ENSG000000060: VNALIAPGQIGICGRTGSGKSSFSLAFFRMVDTFEGHIIIDGIDIAKLPLHTLRSRLSIILDPVLFSGTIRFNLDPERKCSDSTLWEALEIAQLKLVVKALPGGLDAIITEGGENFSQGQ : 1304
Hsa.ABCC9.ENSG000000694: VKAYIKPGQVGICGRTGSGKSSLSLAFFRMVDIFDGKIVIDGIDISKLPLHTLRSRLSIILDPILFSGSIRFNLDPECKCTDDRLWEALEIAQLKNMVKSLPGGLDAVVTEGGENFSVGQ : 1299
Hsa.ABCC10.ENSG00000124: VTFCVQPGELGIVGRTGSGKSSLLLVLFRLLEPSSGRVLLDGVDTSQLELAQLRSQLAIIPEPFLFSGTVRENLDPQGLHKDRALWQALKQCHLSEVITSM-GGLDGELGEGGRSLSLGQ : 1290
Hsa.ABCC4.ENSG000001252: LTALIKSQEVGIVGRTGAGKSSLISALFRLSEP-EGKIWIDKILTTEIGLHDLRKKMSIIPEPVLFTGTMRKNLDPFNEHTDEELWNALQEVQLKETIEDLPGKMDTELAESGSNFSVGQ : 1115
Cel.WBGene00003414: IDLKIEGGEIGVIGRTGSGKSSLTMALYRMIEGESGTIKIDDVEIDTIGLHQLRSKLIIIPEPVVFSGTLRFNLDPFNQYSDDQIWNCLEICQLKQFAQEDDKTLDRYIAEGGKNMSVGE : 1271
Cel.WBGene00003409: ISLNILPGQVGVCGRTGAGKSSLALALFRIVEAADGNISIDQTITSHIGLHDLREKLTIIPENVLFANTLRFNIDPKGQFTDQQLWLALENSNLKAHVELLPHKLESPVAEGGENFSVGQ : 1307
Hro.HelroG163344: INIDIKSREIGIVGRTGAGKSSMTLALFRLIEPSEGSIIIDGVDITSIGLHQLRSKITILPDPVIFSGSLRFNLDPTNEHSDDQIWTALENAHLKTYVASLPAALDNECGEGGQNLSVGQ : 1189
Hro.HelroG157076: INLDIKAKEIGIVGRTGAGKSSLTLALFRLIEPSEGCIRIDGVDISSIGLHQLRSKLTILPDPVIFSGSLRFNLDPTNHHTDEQIWTALDHAHLKTFVASLSGTLDNECGEGGSNLSVGQ : 950
Bfl.230771: ITVNISGGEIGIVGRTGAGKSSLVLAIFRIIEAAGGDIVIDGVRIANIGLHDLRSRITVIPDPVVFSGTLRMNLDPFEKHTDAELWRALDLAHLRDYVMGLDKQLDHDVSEGGTNLSVGQ : 1300
Lgi.LotgiG105097: LTVNFKSGEIGIVGRTGAGKSSLSMAIFRIIESAGGSITIDGADIAKLGLHDLRTNLTILPDPILFTGSLRMNLDPFEEHSDEELWTALEHAHLKTFVSGLPEGLNFECDEGGNNLSVGQ : 1299
Cel.WBGene00003407: VTAHISPCEIGIVGRTGAGKSSLTLALFRIIEADGGCIEIDGTNIADLLLEQLRSRLTIVPDPVLFSGTMRMNLDPFFAFSDDQIWEALRNAHLDSFVKSLQEGLHHHISEGGENLSVGQ : 1313
Cel.WBGene00003408: ISAHIAPSEVGIVGRTGAGKSSLTLALFRIIEADGGSIEIDGINIANLQLEQLRSCLTIVPDPVLFSGTMKMNLDPFSAYSDSQVWEALENAHLKPFVKSLQDGLEHKISEGGENLSVGQ : 1312
Lgi.LotgiG153611: ITFEVKPGEIGVVGRTGAGKSSLILALFRLVEGVAGTITLDGKNIEDIGLHDLRSKITILPDPVLFSGTLRTNLDPFNINTDEELWTALDLAHLKAYVSGLTEGLEFQCGEGGQNFSVGQ : 1259
Lgi.LotgiG110718: VSCDILGGEIGIVGRTGAGKSSMLLTLFRLVEGSGGEIIIDGVNIADIGLHDLRSRLTILPDPVLFSGTLRMNLDPFDEFTDDQIWVALGQSHLKPFVEGLPETIFYECGEGGSNFSVGQ : 1126
Dno.ENSDNOG00000024923: VSFKIQAGEVGIVGRTGAGKSSLARGLLRLLEAAEGGIWIDGVPIAHVGLHTLRSRITIIPDPILFPGSLRMNLDLLHEHADKAIWAALETVQLHALVTSLPGQLQYECAEQGDDLSVGQ : 1254
Mmu.ENSMUSG00000030834: VSLKIHAGEVGIVGRTGAGKSSLAWGLLRLQEAAEGNIWIDGVPITHVGLHTLRSRITIIPDPVLFPGSLRMNLDLLQEHTDEGIWAALETVQLKAFVTSLPGQLQYECAGQGDDLSVGQ : 1312
Cluf.ENSCAFG00000018197: VSFKIHAGEVGIVGRTGAGKSSLAGGLLRLLEAAEGGIWIDGVPIAHVGLHTLRSRITIIPDPTLFPGSLRMNLDMLDEHTDEAIWEALEMVQLRPLVASLPGQLQYECTDQGSDLSVGQ : 1313
Bta.ENSBTAG00000015191: VSFKINAGEVGIVGRTGAGKSSLAGGLLRLVEAAEGGIWIDGVPIAQVGLHTLRSRVTIIPDPILFPGSLRMNLDMLQEHTDEAIWEVLETVQLRATVASLPGQLHYECTDQGDNLSVGQ : 1314
Ggo.ENSGGOG00000009623: VSFKIHAGEVGIVGRTGAGKSSLASGLLRLQEAAEGGIWIDGVPIAHVGLHTLRSRISIIPDPILFPGSLRMNLDLLQEHSDEAIWAALETVQLKALVASLPGQLQYKCADRGEDLSVGQ : 1305
Hsa.ENSG00000091262: VSFKIHAGEVGIVGRTGAGKSSLASGLLRLQEAAEGGIWIDGVPIAHVGLHTLRSRISIIPDPILFPGSLRMNLDLLQEHSDEAIWAALETVQLKALVASLPGQLQYKCADRGEDLSVGQ : 1313
Ptr.ENSPTRG00000007815: VSFKIHAGEVGIVGRTGAGKSSLASGLLRLQEAAEGGIWIDGVPIAHVGLHTLRSRISIIPDPILFPGSLRMNLDLLQEHSDEAIWAALETVQLKALVASLPGQLQYKCADRGEDLSVGQ : 1170
Oan.ENSOANG00000005123: VTVTILPQEVGVVGRTGAGKSSLAVGLLRLFEAAEGHIRIDGVNVARIGLHHLRSKITILPDPILFPGSLRMNLDLLHEHPDGDIWTALEMVQLKAFVADLPGHLDHVCSDQGENVSVGQ : 1268
Mdo.ENSMODG00000005815: LTLKILPQEVGIVGRTWAGKSSLSIGLLRLIEATEGGSDRWGEYQSKWAPCP-EVQITIIPDPILFPGSVRMNLDLLDEHSDDEIWGALEMVQLKTFILGLPGQLQYECLDQGDNL---- : 1308
Gga.ENSGALG00000006698: INLTINGKEIGITGRTGAGKSTLAAGLLRLVEAAEGVILIDGQDIAQLGLHDLRMKITVIPDPVLFSGTLRMNLDPLNQYTDADIWTALELTQLKNFVADLPEQLEYKCTDQGENLSTGQ : 1316
Aca.ENSACAG00000003478: VNIQIKGKEVGIAGRTGAGKSSLAMGLLRLVEAAEGEILIDGIDVAQIGLHDLRSKITVIPDPVLFSGPLRMNFDPLDEHTDEDIWAALELMLLKNFVSDLPGQLAYECSERGGNLSVGQ : 1256
Cel.WBGene00003413: ISADVAAGEIGIVGRTGAGKSSFALALFRMIEAAGGRIVIDDVEVSQIGLHDLRSNITIIPDPVLFSGTLRFNLDPFFTYSDDQIWRALELAHLKHFAAGLPDGLLYKISEAGENLSVGQ : 1312
Aga.AGAP008437: ISLNVRGGEIGIVGRTGAGKSSLTLGLFRIVEAAGGQIIIDGLDISKMGLHQLRGRLTIIPDPVLFSGTLRANVDPFKSYSDDLVWKALELSHLKTFVKGLAAGLDHEIAENGENLSVGQ : 1309
Aga.AGAP027980: ISLNVRGGEIGIVGRTGAGKSSLTLGLFRIVEAAGGQIIIDGLDISKMGLHQLRSRLTIIPDPVLFSGTLRANVDPFKSYSDDQVWKALELSHLKTFVKGLTAGLDHEIAENGENLSVGQ : 1311
Aga.AGAP028128: ISLNVRGGEIGIVGRTGAGKSSLTLGLFRIVEAAGGQIIIDGLDISKMGLHQLRGRLTIIPDPVLFSGTLRANVDPFKSYSDDQVWKALELSHLKTFVKGLSAGLDHEIAENGENLSVGQ : 1308
Spu.026395: ------------------------------------------------------------------------------------------------------------------------ : -
Hsa.ABCC2.ENSG000000238: ITCDIGSMEIGVVGRTGAGKSSLTNCLFRILEAAGGQIIIDGVDIASIGLHDLREKLTIIPDPILFSGSLRMNLDPFNNYSDEEIWKALELAHLKSFVASLQLGLSHEVTEAGGNLSIGQ : 1308
Xtr.ENSXETG00000026360: VTASIQQGEVGIVGRTGAGKSSLTLGLFRILEPATGRICIDEKDISELGLHELRSKITIIPDPVLFSGTLRMNLDPFDNYSDNDIWVALQLAHLKVFASGLPEGLSYICTEGGENLSVGQ : 1299
Cel.WBGene00003410: LNVEIKPHEVGIVGRTGAGKSSVTLSLFRIIEAAEGQIVVDGINLAEIGLHDLRSNLTIIPDPVLFSGTLRFNLDPFNHYSDGDIWKTLEMANLKEFATAHNEQLNYIITEGGDNISVGQ : 1312
Ame.GB53134: ------------------------------------------------------------------------------------------------------------------------ : -
Pma.KM232931_1: LTFSVRGGEVGIVGRTGAGKSSLTLGLFRIVEPASGLIRIDGVDIAQLGLTELRSRLTIIPEPVLFAGSLRWNLDPFGRHEDAELWQVLRMAHLEDFVQQLAGGLDHTCCEGGENLSVGQ : 1030
Gac.ENSGACG00000003037: ICVNIQDREVGIVGRTGAGKSSLALGIFRILEAAKGRIFIDGINIAEIGLHDLRSRITIIPDPVLFSGSLRMNLDPFDVCSDEDLWKALELAHLSSFVSALPQKLNHQCCEGGENLSLGQ : 1319
Loc.ENSLOCG00000007152: ITVQIREQEVGIVGRTGAGKSSLALGIFRILEAAKGEIYIDGVNIAEIGLHDLRSRITIIPDPVLFSGSLRMNLDPFDSYSDEEVWNALELAHLKTFVSGLPDKLNHECSEGGENLSLGQ : 1315
Gmo.ENSGMOG00000005748: ITLSIQEREVGIVGRTGAGKSSLALGIFRILEAAKGKIFVDGVDLADIGLHDLRSRITIIPDPVLFSGSLRMNLDPFDNYTDQEVWSSLELAHLKNFVSNLPDKLSHECSEGGENLSLGQ : 1277
Gac.ENSGACG00000019172: ITLQVHEREVGIVGRTGAGKSSLALGIFRILEAAKGKILIDGVDIADVGLHDLRSRITIIPDPVLFSGSLRMNLDPFDTYTDEEVWRSLELAHLQNFVSNLPDKINHECSEGGENLSLGQ : 1320
Tni.ENSTNIG00000012067: ITLNIHEREVGIVGRTGAGKSSLALGIFRILEAAKGKIFIDGVNIADIGLHDLRSRITIIPDPVLFSGSLRMNLDPFDTYTDEDVWRSLELAHLKTFVANLPDKLNHECSEGGENLSLGQ : 1317
Ola.ENSORLG00000013429: ITLQIQKREIGIVGRTGAGKSSLALGIFRILEAAKGRIFIDGVNIAEIGLHDLRSRITIIPDPVLFSGSLRMNLDPFDIYTDEEIWSSLELAHLKDFVSNLPDKLNHECSEGGENLSLGQ : 1319
Oni.ENSONIG00000018866: ITLHIHEREVGIVGRTGAGKSSLALGIFRILEAAKGKIFIDGVDIADIGLHDLRSRITIIPDPVLFSGSLRMNLDPFDTYTDEEVWSSLELAHLKNFVSNLPDKLNHECTEGGENLSLGQ : 1319
Xma.ENSXMAG00000004906: ITLHINPKEVGIVGRTGAGKSSLALGIFRILEAAKGKIFIDGVNIADIGLHDLRSRITIIPDPVLFSGSLRMNLDPFDTYTDEEIWSSLELAHLKTFVSNLPDKLNYECSEGGENLSLGQ : 1315
Dla.DLAgn_00177430: ITLNIHEREVGIVGRTGAGKSSLALGIFRILEAAKGKIFIDGVNIADIGLHDLRSRITIIPDPVLFSGSLRMNLDPFDTYTDEEVWRSLELAHLKNFVSNLPDKLNHECSEGGENLSLGQ : 1315
Dre.ENSDARG00000016750: ISVHIHEREIGIVGRTGAGKSSLALGIFRILEAAKGEIYIDGINIAEIGLHDLRSRITIIPDPVLFSGSLRMNLDPFNAYSDEEVWNALELAHLKNFVSELPDKLNHECSEGGENLSLGQ : 1315
Ame.ENSAMXG00000004837: ISLHIQRREIGIVGRTGAGKSSLALGIFRILEAAKGKIYIDGFDIAQLGLHDLRSRITIIPDPVLFSGSLRMNLDPFDAYSDEEVWGALELSHLRNFVSGLPEKLNYECSEGGENLSLGQ : 1312
Ame.ENSAMXG00000003085: ITLNVQEREVGIVGRTGAGKSSLALGIFRILEAAKGEIYIDGVNIAQIGLQDLRSRITIIPDPILFSGSLRMNLDPFDGYSDEDVWRALELAHLKNFVSGLPDKLNHECSEGGENLSLGQ : 1320
Dre.ENSDARG00000094901: ISLSVNEREVGIVGRTGAGKSSLALGIFRILEAAKGKIFIDGINIAEIGLHELRSRITIIPDPVLFSGSLRINLDPFDRYTDEEVWRSLELAHLKTFVSDLPDKLNHECSEGGENLSLGQ : 1287
Dre.ENSDARG00000095820: ISLSVNEREVGIVGRTGAGKSSLALGIFRILEAAKGKIFIDGINIAEIGLHELRSRITIIPDPVLFSGSLRINLDPFDRYTDEEVWRSLELAHLKTFVSDLPDKLNHECSEGGENLSLGQ : 1320
Lch.ENSLACG00000022117: INVKIDKEEVGIVGRTGAGKSSLTMGLFRIMEASTGEVFIDGVNTATLGLHDLRSRLSIIPDPVLFCGSLRMNLDPFDNYSDKDVWRALELAHLKNFVSSLPDRLSYECSEGGENLSVGQ : 1320
Dpu.347281: ITCDIQGGEVGIVGRTGAGKSSLTLALFRIIEAASGKITIDGLDIADLGLHALRSRLTIIPDPVLFSGTLRMNLDPFNSYSDDDIWTALEHAHLKTFVKSLPAGLEHEASEGGENLSVGQ : 1319
Lca.KE993868_1: ---------VGIVGRTGAGKSSLTLCLLRMVEAASGAIAIDGVDISRIGLHDLRSRITIIPDPVLFGGSLRDNLDPFGHHSDAEVWEALELAHLGETARALPGGIQHECAEGGENFSVGQ : 577
Pma.KM232930_1: ISCDIKGGEVGIVGRTGAGKSSLTLCLLRMVEAASGAIAIDGVDISRIGLHDLRSRITIIPDPVLFGGSLRDNLDPFGHHSDAEVWEALEFAHLGETARALPGGIQHECAEGGENFSVGQ : 1319
Csa.ENSCSAVG00000008135: INADIKGGEIGIVGRTGAGKSSLTLALFRIIESVEGCITIDGLNISKIGLQDLRSKLSIIPDPVLFSGTLRMNLDPFDSYTDEELWNALKHSHLKDFVLGLTLKLEHEVSEGGENLSVGQ : 1311
Csa.ENSCSAVG00000003792: ITIHINGGEVGIVGRTGAGKSSMTLALFRIIESASGRISIDGLPISNMGLQDLRSKLSIIPDPVLFSGSLRMNLDPFEEYRDSELWDALEHSHLKDYVTSLSEKLEHEVTEGGENLSVGQ : 1252
Cin.ENSCING00000020698: ------------------------------------------------------------------------------------------------------------------------ : -
Tca.TC012253: VNFSINGGEVGIVGRTGAGKSSLTLALFRIIEAAEGEILIDGVNIADLGLHTLRSRLTIIPDAVLFSGTLRMNLDPFDKHSDEEVWKALEHAHLKSFVKGLTAGLHHEVTEGGENLSVGQ : 1313
Aga.AGAP009835: ISFTVNGGEVGIVGRTGAGKSSLTLALFRIIESAGGSIVIDGQDISQLGLHALRSRLTIIPDPVLFSGTLRINLDPFNAQSDDDIWKALEHAHLKTFVKGLTAGINHEVTEGGENLSVGQ : 1309
Lgi.LotgiG107213: IDCKIKSGEIGIVGRTGAGKSSLTLALFRIIESAGGKITIDNINIADIGLHDLRSKLTIIPDPVLFSGSLRMNLDPFEEFDDDKIWQALEHAHLKSFVTSLPAQLNHECSEGGENLSVGQ : 1313
Bfl.90918: ITVDIGSGEIGIVGRTGSGKSSLALALFRIIESAGGDIVIDGINISNIGLHDLRSRISIIPDPVLFSGTLRMNLDPFEQHADVDIWLALQLSHLKNFVVNLEKKLEHDVTEGGQNLSVGQ : 1188
Dme.FBgn0032456: VSFNIQGGEVGIVGRTGAGKSSLTLALFRIIEAAGGRISIDGVDIASMGLHMLRSRLTIIPDPVLFSGSLRINLDPFEIKTDDEIWKALELSHLKSFVKSLAAGLNHEIAEGGENLSVGQ : 1312
Oan.ENSOANG00000013379: ------------------------------------------------------------------------------------------------------------------------ : -
Pma.AKC42143_1: ------------------------------------------------------------------------------------------------------------------------ : -
Lca.KE994284_1: -------------------------------------------------------------DPVLFSGSLRMNLDPFGRHSDADVWSALELSHLKAFVQTLPDKLQHECSEGGENLSVGQ : 717
Cmi.SINCAMG00000015787: ITLTIKGGEVGIVGRTGAGKSSIALGLFRIIEPAEGLIYLDGINISEIGLHDLRSEITIIPEPVVFSGSLRMNLDPFEHHSDNDLWNALELAHLKTFVSDLPNTLNHECSEGGENLSVGQ : 1319
Ler.ctg13956: ITISINGGEIGIVGRTGAGKSSFALGLFRIIEPAEGKILIDGIDVTKIGLHDLRFQITIIP----------------------------------------------------------- : 833
Rty.XP_020377569_1: ITVSINGGEVGIVGRTGAGKSSFALGLFRIIEAAEGQIYIDGIDIAKIGLHDLRFQITIIPDPVLFAGSLRMNLDPFDQYPDADLWTALELAHLKTFVSGLPDTLNHECSEGGDNLSVGQ : 771
Sca.ctg67278: ITVSINGGEIGIVGRTGAGKSSFALGLFRIIEPAEGQIYIDGIDIAKIGLHDLRFQITIIPDPVLFSGSLRMNLDPFDQYSDAGLWKALELAHLKTFVSGLPHKLNHECSEGGENLSVGQ : 1220
Lch.ENSLACG00000001471: -------------------------------------------------------------SP--------------------------------------------------------- : 1020
Tni.ENSTNIG00000005013: ITISINGGEVGIVGRTGAGKSSLTLGLFRIIEAAEGHIFIDGVDIRELGLHDLRSRITIIPDPVLFSGSLRMNLDPFDKYSDEEIWKSLEYSHLKTFVSGLPNKLNHECSEGGENLSVGQ : 1208
Loc.ENSLOCG00000007196: IAVTIEGGEVGIVGRTGAGKSSLTLGLFRIIEPAQGQICIDGVDVSTLGLHDLRSRITIIPDPVLFSGSLRMNLDPFDSYSDEEVWNALELAHLKTFVSGLPDKLNHECSEGGENLSLGQ : 1320
Dre.ENSDARG00000104719: ISVNIAGGEVGIVGRTGAGKSSLTLGLFRIIEAAEGEIRIDGVNIADLGLHELRSRITIIPDPVLFSGSLRMNLDPFDGYTDEEVWRSLELAHLKTFVSGLPDKLNHECSEGGENLSLGQ : 1315
Ame.ENSAMXG00000002943: ITVIIEGGEVGIVGRTGAGKSSLTLGLFRIIEAAQGEICIDGVNIANLGLHDLRSRITIIPDPVLFSGSLRMNLDPFDGYSDEDVWRALELAHLKNFVSGLPDKLNHECSEGGENLSLGQ : 1315
Xma.ENSXMAG7738/17319: ------------------------------------------------------------------------------------------------------------------------ : -
Ola.ENSORLG00000017141: INVDISGGEVGIVGRTGAGKSSLTLGLFRIIEPAEGNIFIDGVDIAKLGLHELRSRITIIPDPVLFSGTLRMNLDPFDSYSDEDIWKALEFSHLKSFVSGLPDKLGHECSEGGENLSLGQ : 1317
Gac.ENSGACG00000000434: ITLVINRGEVGIVGRTGAGKSSLTLALFRIIEASEGHIFIDGVDIALLGLHELRSRITIIPDPVLFSGSLRMNLDPFDCYSDEEVWRALELSHLQSFVSGLPNKLSHECSEGGENLSVGQ : 1320
Oni.ENSONIG00000007824: VTISINGGEVGIVGRTGAGKSSLTLGLFRIIEAAEGHIFIDGVDIAKLGLHELRSRITIIPDPVLFSGSLRMNLDPFDSYTDEEVWRALEFSHLKTFVSSLPNKLNHDCSEGGENLSVGQ : 1320
Dla.DLA_00195360: ------------------------------------------------------------------------------------------------------------------------ : -
Xtr.ENSXETG00000019661: INVTIQGGEVGIVGRTGAGKSSLTLGLFRINEAAAGEIVIDGCNLAKIGLHDLRFRVTIIPDPVLFSGTLRMNLDPFDKYTDDDIWTSLELAHLKRFVANLPDRLNHECAEGGENLSIGQ : 1302
Aca.ENSACAG00000005349: ITITISGGEVGIVGRTGAGKSSLTLGLFRINEAAEGQILIDGVDIASIGLHDLRFKVTIIPDPVLFSGSLRMNLDPFEQYSDEEVWRSLELAHLKAFVSALPDKLLHECAEGGENLSVGQ : 1315
Gga.ENSGALG00000006646: INITINGGEVGIVGRTGAGKSSLTLGLFRINEAAEGEIIIDGINIAKIGLHDLRFKITIIPDPILFSGSLRMNLDPFDQHSDEDIWRSLELAHLKNFVSSLPDKLNHECSEGGENLSVGQ : 1318
Dno.ENSDNOG00000014990: RLYTLGPGDVGIVGRTGAGKSSLTLGLFRINESAEGEIVIDGVNIAHIGLHDLRFRITIIPDPVLFSGSLRMNLDPFSQYSDDDVWTSLELAHLKSFVSALPDKLDHECAEGGENLSVGQ : 1318
Mmu.ENSMUSG00000023088: INVTIEGGEVGIVGRTGAGKSSLTLGLFRINESAEGEIIIDGVNIAKIGLHNLRFKITIIPDPVLFSGSLRMNLDPFSQYSDEEVWMALELAHLKGFVSALPDKLNHECAEGGENLSVGQ : 1318
Ggo.ENSGGOG00000004324: ISVTINGGEVGIVGRTGAGKSSLTLGLFRINESAEGEIIIDGINIAKIGLHDLRFKITIIPDPVLFSGSLRMNLDPFSQYSDEEVWTSLELAHLKDFVSALPNKLDHECAEGGENLSVGQ : 1248
Hsa_ENSG00000103222: INVTINGGEVGIVGRTGAGKSSLTLGLFRINESAEGEIIIDGINIAKIGLHDLRFKITIIPDPVLFSGSLRMNLDPFSQYSDEEVWTSLELAHLKDFVSALPDKLDHECAEGGENLSVGQ : 1318
Ptr.ENSPTRG00000007812: INVTINGGEVGIVGRTGAGKSSLTLGLFRINESAEGEIIIDGINIAKIGLHDLRFKITIIPDPVLFSGSLRMNLDPFSQYSDEEVWTSLELAHLKDFVSALPDKLDHECAEGGENLSVGQ : 1272
Cluf.ENSCAFG00000018208: INITINGGEVGIVGRTGAGKSSLTLGLFRINESAEGEIIIDDINIAKIGLHDLRVKITIIPDPVLFSGSLRMNLDPFSQYSDEEVWTSLELAHLKDFVSGLPDKLNQECAEGGENLSVGQ : 1318
Bta.ENSBTAG00000021090: INVTIDGGEVGIVGRTGAGKSSLTLGLFRIKESAEGEIIIDDINIAKIGLHDLRFKITIIPDPVLFSGSLRMNLDPFSQYSDEEVWTSLELAHLKGFVSALPDKLNHECAEGGENLSVGQ : 1318
Mdo.ENSMODG00000004194: VNVTIEGGEVGIVGRTGAGKSSLTLGLFRINESAGGEIIIDGINIAKIGLHHLRFKITIIPDPVLFSGSLRMNLDPFDQYSDEDIWTSLELAHLKNFVSSLPDKLNHECTEGGENLSVGQ : 1318
Oan.ENSOANG00000005124: INVTIDGGEVGIVGRTGAGKSSLTLGLFRINESAEGEIIIDGVNIAKIGLHHLRFKITIIPDPVLFSGSLRMNLDPFDQYSDEDIWRSLELAHLKNFVSLLPDKLNHECTEGGENLSVGQ : 1107
Bfl.232174: IDVTIKGGEIGVVGRTGAGKSSMTLGLFRIIEAAGGDIEIDGVNISKIGLHDLRGRITIIPDPVLFSGTLRMNLDPFDSCSDQDIWVALELSHLKDFVMGLGAQLEYEVSEGGENLSVGQ : 1319
Bfl.118638: INVNIKGGEIGVVGRTGAGKSSMTLGLFRIIEAAGGDIEIDGVNISKIGLHDLRGRITIIPDPVLFSGTLRMNLDPFDSCSDRDIWVALELSHLKDFVMGLGAQLEYEVSEGGENLSVGQ : 1182
Bfl.118636: IDVTIKGGEIGIVGRTGAGKSSLTLAIFRIIEAAGGDIEIDGVNISKIGLHDLRGRITIIPDPVLFSGTLRMNLDPFDSCSDQDIWVALELSHLKDFVMGLGAQLEHEVSEGGENLSVGQ : 1306
Bfl.128060: IDVTIKGGEIGIVGRTGAGKSSLTLAIFRIIEAAGGDIEIDGVNISKIGLHDLRGRITIIPDPVLFSGTLRMNLDPFDSCSDQDIWVALELSHLKDFVMGLGAQLEHEVSEGGENLSVGQ : 1306
Dre.ENSDARG00000096662: ISLKVRGGEIGIVGRTGAGKSSMTLCLFRLLEAAAGEIVIDDVKISEIGLHDLRSKLTIIPEPVLFSGTLRMNLDPFERYSDEEVWKALELSHLQKFVTNQAAKLELECSEGGENLSVGQ : 1316
Ame.ENSAMXG00000016253: ISLRVKGGEIGIVGRTGAGKSSMTLCLFRLLEAAGGEITIDGVKIKEIGLHDLRSKLTIIPEPVLFSGTLRMNLDPFEKYSDDDMWNALTLSHLHKFVSNQPAKLALECSEGGENLSVGQ : 1319
Ola.ENSORLG00000020741: ITLDVKGGEIGIVGRTGAGKSSMTLCLFRLLEAAAGEITIDGVKIAEIGLHDLRSRLTIIP-PVLFSGTLRMNLDPFDKYSDEDVWKALEHSHLHGFVRNQPAQLQMECAEGGENLSVGQ : 1316
Gmo.ENSGMOG00000010029: LTLSVQGGEVGIVGRTGAGKSSMTLCLFRLLEAAGGEITIDGVKISEIGLHDLRSKLTIIPEPVLFSGTLRMNLDPFDSYSDEDLWKALEHSHLHKFVTNAPAKLEMECAEGGENLSVGQ : 1267
Xma.ENSXMAG00000012203: ITLSVKGGEIGIVGRTGAGKSSMTLCLFRLLEAAGGEITIDGVKISEIGLHDLRSKLTIIPEPVLFSGTLRMNLDPFEKYSDDDVWKALEHSHLRKFIGNQPAKLELECTEGGENLSVGQ : 1318
Gac.ENSGACG00000005901: LTLSVKGGEIGIVGRTGAGKSSMTLCLFRLLEAAAGEISIDGVKISELGLHDLRSRLTIIPEPVLFSGTLKMNLDPFEKYSDEELWGALQQSHLHKFVSNQPAKLELECSEGGENLSVGQ : 1312
Oni.ENSONIG00000019586: ITLKVKGGEIGIVGRTGAGKSSMTLCLFRLLEAAGGEITIDNVKISEIGLHDLRSKLTIIPEPVLFSGTLRMNLDPFEKYSDEEVWKALEHSHLHKFVSNQAAKLDLECSEGGENLSVGQ : 1315
Dla.DLAgn_00098120: LTLNVKGGEIGIVGRTGAGKSSMTLCLFRLLEAAAGDITIDEVKISEIGLHDLRSKLTIIPEPVLFSGTLRMNLDPFESYTDEEVWKALQHSHLHKFVSNQPAKLELECSEGGENLSVGQ : 1320
Cmi.SINCAMG00000013336: LHLTIDGGEIGIVGRTGAGKSSMTLCLFRILEAAEGEITVDGVRIAEMGLHDLRSKLTIIPDPVLFSGTLRMNLDPFDHHCEEDLWRVLELSHLKRFVSNQAAGLLLQCSEGGENLSVGQ : 1220
Ler.ctg12190: ------------------------------------------------------------------------------------------------------------------------ : -
Sca.ctg14163: LSLNVHGGEVGIVGRTGAGKSSMTLCLFRIIEAAQGEIIIDGVKIADIGLHDLRSKLTIIPDPVLFSGTLRMNLDPFNQYSEEEVWNVLELSHLKGYVHSLPAGLQHECSEGGENLSVGQ : 1099
Rty.XP_020375725_1: LHLNVQGGEVGIVGQTGAGKSSMTLCLFRIIEAAEGEIIIDGVKIADIGLHDLRSKLTIIPDPVLFSGSLRMNLDPFNQYSEDEVWKAVELSHLKQFVNSLPAKLEHECSEGGENLSVGQ : 739
Sac_EU250283: LQLSVHGGEVGIVGRTGAGKSSMTLCLFRIIEAAKGEITIDGVKIADIGLHDLRSKLTIIPDPVLFSGTLRMNLDPFEQYTEEEVWNALELSHLKQFVHTLPAGLEHECSEGGENLSVGQ : 1319
Tni.ENSTNIG00000004171: L-LSVKGGEIGIVGRTGAGKSSMTLCLFRLLEAAAGEISIDEVKISEIGLHDLRSKLTIIPEPVLFSGTLRMNLDPFDKYNDEEVWKALQHSHLDKFVSNNPAKLELECSEGGENLSVGQ : 1268
Aca.ENSACAG00000001396: LSLHVKGGEVGIVGRTGAGKSSMTLCLFRILEAVEGEIKIDGLRIADIGLHDLRSKLTIIPDPVLFSGTLRMNLDPFNKYSEEEIWNALELSHLKRFVSAQPAMLDYECSEGGENLSVGQ : 1314
Gga.ENSGALG00000007522: LNLRVHGGEIGIVGRTGAGKSSMTLCLFRILEAAKGEIRIDGVRISEIGLHDLRSRLTIIPDPVLFSGTLRMNLDPFNKYSDEEVWKALELSHLKRFVSSQPSMLEFECSEGGENLSVGQ : 1265
Xtr.ENSXETG00000012239: LNLKVNGGEVGIVGRTGAGKSSMTLCLFRILEPAEGIVKIDNVNISEIGLQDLRSRLTIIPDPVLFSGTLRMNLDPFNKYSDDEIWKALELSNLKKFVAGQPSQLEYECSEGGENLSVGQ : 1317
Loc.ENSLOCG00000010918: LTLSVKGGEIGIVGRTGAGKSSMTLCLFRILEAAGGEITIDGVKISEIGLHDLRSKLTIIPEPVLFSGTLRMNLDPFEQYTDEDIWRALELSHLKKFVSSQETKLNYECSEGGENLSVGQ : 1317
Lch.ENSLACG7209/6619: LNLSVKGGEVGIVGRTGAGKSSMTLCLFRILEAAAGEIAIDKEKIADIGLQDLRSKLTIIPDPVLFSGTLRMNLDPFDNYTDEDIWKALEFSHLKRFVTSQSAGLNHECTEGGENLSVGQ : 1300
Mdo.ENSMODG00000020910: ------------------------------------------------------------------------------------------------------------------------ : -
Dno.ENSDNOG00000046300: LSLRVRSGEVGIVGRSGAGKSSLALCLFRVLEAACGEMLVDGLNVARIGLHDLRSRLTIIPDPFLFSGTLRMNLDPFGNYSEEDMWRALELSHLHAFMSSQPAGLNFQCSEGGENLSVGQ : 1311
Mmu.ENSMUSG00000020865: VTVHVQGGEVGIVGRTGAGKSSMTLCLFRILEAAEGEIVIDGLNVAHIGLHDLRSQLTIIPDPILFSGTLRMNLDPFGRYSEEDIWRALELSHLNTFVSSQPAGLDFQCAEGGDNLSVGQ : 1317
Ggo.ENSGGOP00000003354: LSLHVHGGEVGIVGRTGAGKSSMTLCLFRILEAAKGEIRIDGLNVADIGLHDLRSQLTIIPDPILFSGTLRMNLDPFGSYSEEDIWRALELSHLHTFVSSQPAGLDFQCSEGGENLSVGQ : 1320
Hsa.ENSG00000108846: LSLHVHGGEVGIVGRTGAGKSSMTLCLFRILEAAKGEIRIDGLNVADIGLHDLRSQLTIIPDPILFSGTLRMNLDPFGSYSEEDIWWALELSHLHTFVSSQPAGLDFQCSEGGENLSVGQ : 1320
Ptr.ENSPTRG00000009406: ------------------------------------------------------------------------------------------------------------------------ : -
Cluf.ENSCAFG00000017201: LSLHVHGGEVGIVGRTGAGKSSMTLCLFRILEAAEGEIRIDGLNVADIGLHDLRSQLTIIPDPILFSASLRMNLDPFGYYSEEDLWRALELSHLHTFVSSQPAGLDFQCSEGGENLSVGQ : 1320
Bta.ENSBTAG00000020070: LSLRVHGGEVGIVGRTGAGKSSMTLCLFRILEAAEGEIYIDGLNVADIGLHDLRSKLTIIPDPILFSGTLRMNLDPFGCYSEEDMWQALELSHLHTFVSSQPAGLDFQCSEGGENLSVGQ : 1320

Hsa.CFTR.ENSG0000000162: KQLMCLARSVLSKAKILLLDEPSAHLDPVTYQIIRRTLKQAFADCTVILCEHRIEAMLECQQFLVIEENKVRQYDSIQKLLNERSLFRQAISPSDRV : 1215
Hsa.ABCC5.ENSG000001147: RQLLCIARALLRHCKILILDEATAAMDTETDLLIQETIREAFADCTMLTIAHRLHTVLGSDRIMVLAQGQVVEFDTPSVLLSNSSRFYAMFAAAENK : 1299
Hsa.ABCC11.ENSG00000121: RQLLCIARAVLRNSKIILIDEATASIDMETDTLIQRTIREAFQGCTVLVIAHRVTTVLNCDHILVMGNGKVVEFDRPEVLRKKPGSLFAALMATATS : 1282
Hsa.ABCC12.ENSG00000140: RQLLCVARALLRNSKIILLDEATASMDSKTDTLVQNTIKDAFKGCTVLTIAHRLNTVLNCDHVLVMENGKVIEFDKPEVLAEKDSAFAMLLAAEVRL : 1249
Hsa.ABCC8.ENSG000000060: RQLFCLARAFVRKTSIFIMDEATASIDMATENILQKVVMTAFADRTVVTIAHRVHTILSADLVIVLKRGAILEFDKPEKLLSRDSVFASFVRADK-- : 1399
Hsa.ABCC9.ENSG000000694: RQLFCLARAFVRKSSILIMDEATASIDMATENILQKVVMTAFADRTVVTIAHRVHTILTADLVIVMKRGNILEYDTPESLLAQNGVFASFVRADM-- : 1394
Hsa.ABCC10.ENSG00000124: RQLLCLARALLTDAKILCIDEATASVDQKTDQLLQQTICKRFANKTVLTIAHRLNTILNSDRVLVLQAGRVVELDSPATLRNQHSLFQQLLQSSQQG : 1387
Hsa.ABCC4.ENSG000001252: RQLVCLARAILRKNQILIIDEATANVDPRTDELIQKKIREKFAHCTVLTIAHRLNTIIDSDKIMVLDSGRLKEYDEPYVLLQNESLFYKMVQQLGKA : 1212
Cel.WBGene00003414: RQLLCLCRALLRGARIVILDEATASVDTVTDGIVQRAIRQHFPQSTTISIAHRLDTIVDSDRIVVLDAGRVAEFDTPSNLLLNDSLYSQLLNEKNRK : 1368
Cel.WBGene00003409: RQLLCLTRALLRKSKVLVLDEATAGIDNRTDTMVQATIREKFADSTIITIAHRLHTIIDYDRIIVMDAGRIVEDGIPGELLKNNSQFYGLAKSAKIV : 1404
Hro.HelroG163344: RQLVCLGRALLHKTGLLVLDEATAAVDLETDKVIQETIRTQFDDCTILTIAHRINTILNCDRVLVMDKGNVMEFDNPQTLLNNRSLFYGLAKDAKIV : 1286
Hro.HelroG157076: RQLLCLGRALLHKTGVLVLDEATAAVDLETDKIIQETIRTQFDDCTVLTIAHRINTILNCDRVLVMDGGRVAEFDSPESLLDNQSLFYALAKDANVV : 1047
Bfl.230771: RQLVCLARALLRKSKLLVLDEATASVDPETDALIQTTIRTQFSDCTVLTIAHRLNTIMDSTRILVLDGGKVAEFDTPENLINSKGLFSSMVQDAGLV : 1397
Lgi.LotgiG105097: RQLLCLARSLLRKTKILVLDEATAAVDMETDDLIQKTIRSEFKDSTVLSIAHRLNTVLDYDKILVLDNGEIIEFDSPQSLIEDDSVFYSMAKDAKLV : 1396
Cel.WBGene00003407: RQLICLARALLRKTKVLVLDEAAAAVDVETDSLLQKTIREQFKDCTVLTIAHRLNTVMDSDRLLVLDKGCVAEFDTPKKLLSNDGIFYSMAKDANVV : 1410
Cel.WBGene00003408: RQLICLARALLRKTKVLVLDEAAAAVDVETDSLIQKTIREQFKECTVLTIAHRLNTVMDSDRLLVLDKGRVAEFDSPKNLLANDGIFYSMAKDANVV : 1409
Lgi.LotgiG153611: RQLVCLARALLRKTRVLVLDEATAAVDLKTDVLIQETIRTEFQQCTLITVAHRINTIMDYDRIMVLDKGKIVEFDSPSNLLLDSTQFYSLARDSNI- : 1355
Lgi.LotgiG110718: RQLVCLARTLLRRTRILVLDEATAAVDYETDSLIQKTVRESFKDCTIITIAHRLNTILDYDRIIVMDDGRIVEFDKPNVLLEDTSVFYGMAREAGIT : 1223
Dno.ENSDNOG00000024923: KQLLCLARALLRKTQILILDEATAAVDPGTELQMQAALGSWLAGCTVLLIAHRLPSVMDCAR----------------------------------- : 1316
Mmu.ENSMUSG00000030834: KQLLCLARALLRKTQILILDEATASVDPGTEMQMQAALERWFTQCTVLLIAHRLRSVMDCARVLVMDEGQVAESGSPAQLLAQKGLFYRLAHESGLA : 1409
Cluf.ENSCAFG00000018197: KQLLCLARALLRKTQILILDEATAAVDPGTELQMQAALGSWLAQCTVLLIAHRLRSVLDCARVLVMDKGQVAESGSPAQLLAQKGLFYRLAQESGLV : 1410
Bta.ENSBTAG00000015191: KQLLCLARALLRKTQILILDEATAAVDPGTERQMQAALGSWFAQCTVLLIAHRLRSVLDCARVLVMDEGQVAESGSPAQLLAQKGLFYRLAQESGLV : 1411
Ggo.ENSGGOG00000009623: KQLLCLARALLRKTQILILDEATAAVDPGTELQMQAMLGSWFAQCTVLLIAHRLRSVMDCARVLVMDKGQVAESGSPAQLLAQKGLFYRLARESGLV : 1402
Hsa.ENSG00000091262: KQLLCLARALLRKTQILILDEATAAVDPGTELQMQAMLGSWFAQCTVLLIAHRLRSVMDCARVLVMDKGQVAESGSPAQLLAQKGLFYRLAQESGLV : 1410
Ptr.ENSPTRG00000007815: KQLLCLARALLRKTQILILDEATAAVDPGTELQMQAMLGSWFAQCTVLLIAHRLRSVMDCARVLVMDKGQVAESGSPAQLLAQKGLFYRLAQESGLV : 1267
Oan.ENSOANG00000005123: KQLLCLARALLRKTKILVLDEATAAVDPQTDLQIQATLRTQFANCTVLTIAHRLNTVMDCNRVLVMDDGQVVEFDSPARLLTRKGLFYRLAEESGLV : 1365
Mdo.ENSMODG00000005815: ------------------------------------------------------------------------------------------------- : -
Gga.ENSGALG00000006698: KQLVCLARALLQKAKVLILDEATAAIDIETDLQIQTALRTQFKESTVLTIAHRINTIMDCDRDLVLENGQIAEFDTPKQLTAQKGLFYKLMEESGLA : 1413
Aca.ENSACAG00000003478: RQLICLTRALLRRGNVVFLDEATAAVDMETDLQIQSAIRSQFRDCTVLTIAHRVSTLMDCDRIIVMESGQVSECDTPQNLIARKGMFYTMAKESGLA : 1353
Cel.WBGene00003413: RQLVALARALLRHTRVLVLDEATAAVDVATDALIQETIREEFKECTVFTIAHRLNTIMDYDRIMVLDKGSILEFDTPDALMADNSAFAKMVADAAEQ : 1409
Aga.AGAP008437: RQLICLARAVLRKTKVLILDEATAAVDLETDDLIQKTIRTEFADCTILTIAHRLNTILDSDRVLVLDKGLVAECDSPQNLLANESIFFGMAKNAGIV : 1406
Aga.AGAP027980: RQLVCLARAILRKTKVLILDEATAAVDLETDDLIQKTIRTEFADCTILTIAHRLNTILDSDRVLVLDKGLVAECDSPQNLLANDSIFHSMAKNAGIV : 1408
Aga.AGAP028128: RQLICLARAVLRKTKVLILDEATAAVDLETDDLIQKTIRTEFADCTILTIAHRLNTILDSDRVLVLDKGLVAECDSPQNLLANESIFYGMAKNAGIV : 1405
Spu.026395: ------------------------------------------------------------------------------------------------- : -
Hsa.ABCC2.ENSG000000238: RQLLCLGRALLRKSKILVLDEATAAVDLETDNLIQTTIQNEFAHCTVITIAHRLHTIMDSDKVMVLDNGKIIECGSPEELLQIPGPFYFMAKEAGIE : 1405
Xtr.ENSXETG00000026360: RQLVCLARALLRKTKILVLDEATAAVDLETDDLIQNTIRKEFEDCTIITIAHRLNTIMDYTRL---------------------------------- : 1362
Cel.WBGene00003410: RQLVCLARALLRKTRVLILDEATAAVDVSTDALIQKTIREEFANATVLTIAHRLNTIMDYDRIIVLNDGKVGEFDSPANLLSNNSEFYSMAKRAGLI : 1409
Ame.GB53134: ------------------------------------------------------------------------------------------------- : -
Pma.KM232931_1: RQLVCLAR----------------------------------------------------------------------------------------- : 1038
Gac.ENSGACG00000003037: RQLLCLARALLRKTRILVLDEATAAVDLKTDQLIQSTIRTQFDDCTVLTIAHRLNTIMDYNRVIVMDRGYIAEIDSPSELIRLQGFFYQMCAEAGLV : 1416
Loc.ENSLOCG00000007152: RQLVCLARALLRKTKVLVLDEATAAVDLETDNLIQSTIRSQFEECTVLTIAHRLNTIMDYTRVIVMDRGQITEMDTPSNLIASRGQFYRMCREAGLV : 1412
Gmo.ENSGMOG00000005748: RQLVCLARALLRKTKILVLDEATAAVDLETDTLIQSTIRTQFEDCTVLTIAHRLNTIMDYTRVIVMDKGLISETDSPANLITQRGQFYRMCREAGLV : 1374
Gac.ENSGACG00000019172: RQLVCLARALLRKTKILVLDEATAAVDLETDALIQSTIRTQFEDCTVLTVAHRLNTIMDYTRVIVMDRGHISEMDTPANLIAQRGQFYRMCREAGLM : 1417
Tni.ENSTNIG00000012067: RQLVCLARALLRKTKILVLDEATAAVDLETDTLIQSTIRTQFEDCTVLTIAHRLNTIMDYTRVIVMDKGHISEMDSPGNLIAQRGQFYRMCREAGLV : 1414
Ola.ENSORLG00000013429: RQLVCLARALLRKTKILVLDEATAAVDLETDTLIQSTIRTQFEDCTVLTIAHRLNTIMDYTRVIVMDRGYISEMDSPANLISQRGQFYRMCREAGLV : 1416
Oni.ENSONIG00000018866: RQLVCLARALLRKTKILVLDEATAAVDLETDTLIQSTIRTQFEDCTVLTIAHRLNTIMDYTRVIVMDRGHVSEMDSPANLISQRGQFYRMCREAGLV : 1416
Xma.ENSXMAG00000004906: RQLVCLARALLRKTKILVLDEATAAVDLETDTLIQSTIRTQFEHCTVLTIAHRLNTIMDYTRVIVMDRGHISEMDSPANLISQRGQFYRMCLEAGLV : 1412
Dla.DLAgn_00177430: RQLVCLARALLRKTKILVLDEATAAVDLETDTLIQSTIRTQFEDCTVLTIAHRLNTIMDYTRVIVMDRGHISEMDSPANLIAQRGQFYRMCREAGLV : 1412
Dre.ENSDARG00000016750: RQLVCLARALLRKTKVLVLDEATAALDLETDTLIQSTIRSQFEDCAVLTIAHRLNTIMDYTKVIVMDKGHVVEMDSPSNLIAKRGQFYYMCREAGLL : 1412
Ame.ENSAMXG00000004837: RQLVCLARALLRKTKILVLDEATAAVDLETDTLIQSTIRSQFEDCTVLTIAHRLNTIMDYTKVIVMDKGHIAEMDTPANLIAQRGQFYRMCREAGLA : 1409
Ame.ENSAMXG00000003085: RQLVCLARALLRKTKILVLDEATAAVDLETDNLIQSTIRTQFEDCTVLTIAHRLNTIMDYTRVIVMDNGYITEMDSPTNLIAQRGQFYCMCREAGLA : 1417
Dre.ENSDARG00000094901: RQLICLARALLRKTKILVLDEATAAVDLKTDNLIQSTIRTQFEDCTVLTIAHRLNTIMDYTRVIVMDRGNITEIDSPSNLISQHGQFYRMCREAGLV : 1384
Dre.ENSDARG00000095820: RQLVCLARALLRKTKILVLDEATAAVDLETDNLIQSTIRTQFEDCTVLTIAHRLNTIMDYTRVIVMDRGKITEVDSPSNLISQHGQFYRMCREAGLV : 1417
Lch.ENSLACG00000022117: RQLVCLARALLRKSKILVLDEATAAVDLETDDLIQSTIRTQFEDCTVLTIAHRLNTIMDCTRVMVLDRGQIVEFDAPAKLLLQKGLFYRLASDAGLT : 1417
Dpu.347281: RQLICLARALLRKTKVLILDEATAAVDLETDDLIQATIRKEFKEGTVITIAHRLNTILDSNRVMVLDKGEIKEYAPPNELLENESIFYGMARDAGLV : 1416
Lca.KE993868_1: RQLVCLARALLRRTRVLVLDEATAAVDVETDALIQGTIRTHFHACTVLTIAHRLHT----------------------------------------- : 633
Pma.KM232930_1: RQLACLARALLRRTRVLVLDEATAAVDVETDALIQGTIRTHFHACTVLTIAHRLHTVLDCTRVMVLSSGQIVEFDSPDVLLSRDSAFHKMARDAGIV : 1416
Csa.ENSCSAVG00000008135: RQLVCLARALLRKSKILVLDEATAAVDLETDDLIQATIRVQFEDCTTFTIAHRLNTIMDSTRVLVLDAGKVAEFDSPINLLKAKGIFYSMAKDAGL- : 1407
Csa.ENSCSAVG00000003792: RQLVCLARALLRKSKILVLDEATAAVDLETDDLIQATIREQFSDCTTFTIAHRLNTIMDSTRVLVLDAGKVAEFDTPDNLLQAKGIFFSMAKDAGLA : 1349
Cin.ENSCING00000020698: ------------------------------------------------------------------------------------------------- : -
Tca.TC012253: RQLICLSRALLRKTKVLILDEATAAVDLETDDLIQKTIRSEFKNCTVLTIAHRLNTIMDSDRVVVLDNGRIVEFDSPSNLLKQQSIFYSMCKDAGLV : 1410
Aga.AGAP009835: RQLICLARALLRKTKVLILDEATAAVDLETDDLIQRTIRTEFKDCTVLTIAHRLNTIMDSDKVIVLDKGQIVEFAPPAELLQSNSAFYSMAKDAGLV : 1406
Lgi.LotgiG107213: RQLICLSRALLRKSRILILDEATAAIDLETDDLIQATIKIEFESCTVLTIAHRLNTILDYDRIMVLDSGRIKELDDPHNLLRDNTVFYGMAKDAGLV : 1410
Bfl.90918: RQLVCLARALLKKSKILVLDEATAAVDLETDDLIQSTIRTQFADCTVLTIAHRLNTIMDSTRVLVLDAGQIEEFDTPENLISAKGMFYGMVKDAGLV : 1285
Dme.FBgn0032456: RQLVCLARALLRKTKVLVLDEATAAVDLETDDLIQKTIRTEFKECTVLTIAHRLNTILDSDKVIVLDKGQIIEFASPTELLDNKSAFYSMAKDANLV : 1409
Oan.ENSOANG00000013379: ------------------------------------------------------------------------------------------------- : -
Pma.AKC42143_1: ------------------------------------------------------------------------------------------------- : -
Lca.KE994284_1: RQLVCLARALLRRTRLLVLDEATAAIDLETDDLIQTTIRTHFQGCTVLTIAHRLNTIMDYTRVIVLDKGQIAEFDSPTNLIARRGIFQSMARDAGLV : 814
Cmi.SINCAMG00000015787: RQLICLARALLRKSKVLVLDEATAAVDLVTDKLIQSTIKSHFDQSTVLTIAHRLHTIMDYTRVLVLDKGEIIEFDTPANLLAKKGVFYHMAADSGLL : 1416
Ler.ctg13956: ------------------------------------------------------------------------------------------------- : -
Rty.XP_020377569_1: RQLLCLARALLRKSKILVLDEATAAVDLETDELIQSTIRNQFADCTVLTIAHRLKTIMDYTRIMVLDKGQIVEFDAIPALLHRKGAFYKMAKDSGLL : 868
Sca.ctg67278: RQLLCLARALLRKSKILVLDEATAAVDLETDELIQSTIRNQFAECTVLTIAHRLKTIIDYTRIMVLDRGRIVEFDTAPQLLHRKGAFHKMAVDSGI- : 1316
Lch.ENSLACG00000001471: ------------------------------------------------------------------------------------------------- : -
Tni.ENSTNIG00000005013: RQLLCLARALLRKSKVLVLDEATAAVDMETDHLIQATIRSQFEDCTVLTIAHRLNTIMDYSRVLVLDKGELVEFASPSNLLAEKGSFYQMAKDAGLV : 1305
Loc.ENSLOCG00000007196: RQLVCLARALLRKTKVLVLDEATAAVDLETDNLIQSTIRSQFEECTVLTIAHRLNTIMDYTRVLVLDKGQIVEFDSPSNLLAKKGIFYKMAKDSGLL : 1417
Dre.ENSDARG00000104719: RQLVCLARALLRKTKILVLDEATAAVDLETDNLIQSTIRTQFEDCTVLTIAHRLNTIMDYTRVLVLDKGQMAEFDSPSNLIAKKGIFYKMAKDSGLV : 1412
Ame.ENSAMXG00000002943: RQLVCLARALLRKTKILVLDEATAAVDLETDNLIQSTIRTQFEDCTVLTIAHRLNTIMDYTRVLVLDKGKMAEFDSPASLIAKKGIFYKMAKDSGLV : 1412
Xma.ENSXMAG7738/17319: ------------------------------------------------------------------------------------------------- : -
Ola.ENSORLG00000017141: RQLLCLARALLRKTKVLVLDEATAAIDMETDDLIQTTIRSQFEGCTVLTIAHRLNTIMDYTRVLVLDKGQMAEFDSPSNLIAQKGAFYRMAKDSGLI : 1414
Gac.ENSGACG00000000434: RQLLCLARALLRKTKILVLDEATAAVDMETDNLIQSTIRSQFEDCTVLTIAHRLNTVMDYTRILVLDKGEMAEFDAPHNLLAQRGAFYKMAKDAGLV : 1417
Oni.ENSONIG00000007824: RQLLCLARALLRKTRILVLDEATAAVDMETDNLIQSTIRSQFEDCTVLTIAHRLNTIMDYTRVLVLENGAMAEFDSPSNLISQRGAFYKMAKDSGLV : 1417
Dla.DLA_00195360: -----------------------------------------------------------------IDNGSATAF----------------------- : 776
Xtr.ENSXETG00000019661: RQLVCLARALLRKTKILVLDEATAAVDLETDGLIQSTIRKEFQDCTVITIAHRLNTIMDYTKVIVLDKGQVVEFDSPSNLLQQQGIFFNMAKDSGLV : 1399
Aca.ENSACAG00000005349: RQLVCLSRALLRRSKILVLDEATAAVDLETDCLIQATIRRQFEGCTVLTIAHRLNTIMDYTR----------------------------------- : 1377
Gga.ENSGALG00000006646: RQLVCLARALLRKSKILVLDEATAAVDLETDNLIQSTIKSQFEECTVLTIAHRLNTIMDYTRVLVLDRGEVVECDSPDNLLQAKGLFYSMAKDSGLA : 1415
Dno.ENSDNOG00000014990: RQLVCLARALLRKTKILVLDEATAAVDLETDDLIQSTIRTQFDDCTVLTIAHRLNTIMDYTRVIVLDKGEVREFGSPSELLQQRGLFYSMARDANLA : 1415
Mmu.ENSMUSG00000023088: RQLVCLARALLRKTKILVLDEATAAVDLETDNLIQSTIRTQFEDCTVLTIAHRLNTIMDYTRVIVLDKGEVRECGAPSELLQQRGIFYSMAKDAGLV : 1415
Ggo.ENSGGOG00000004324: RQLVCLARALLRKTKILVLDEATAAVDLETDDLIQSTIRTQFEDCTVLTIAHRLNTIMDYTRVIVLDKGEIQEYGSPSDLLQQRGLFYSMAKDAGLV : 1345
Hsa_ENSG00000103222: RQLVCLARALLRKTKILVLDEATAAVDLETDDLIQSTIRTQFEDCTVLTIAHRLNTIMDYTRVIVLDKGEIQEYGAPSDLLQQRGLFYSMAKDAGLV : 1415
Ptr.ENSPTRG00000007812: RQLVCLARALLRKTKILVLDEATAAVDLETDDLIQSTIRTQFEDCTVLTIAHRLNTIMDYTRVIVLDKGEIQEYGAPSDLLQQRGLFYSMAKDAGLV : 1369
Cluf.ENSCAFG00000018208: RQLVCLARALLRKTKILVLDEATAAVDLETDDLIQSTIRTQFDDCTVLTIAHRLNTIMDYTRVIVLDKGEIRECGQPSDLLQQRGLFYSMAKDAGLV : 1415
Bta.ENSBTAG00000021090: RQLVCLARALLRKTKILVLDEATAAVDLETDDLIQSTIRTQFDDCTVLTIAHRLNTIMDYTRVIVLDKGEIQEWGSPSDLLQQRGLFYSMAKDSGLV : 1415
Mdo.ENSMODG00000004194: RQLVCLARALLRKTKILVLDEATAAVDLETDNLIQSTIRTQFDDCTVLTIAHRLNTILDYTRVIVLDKGEIVECDSPPVLLQKKGIFYSMAKDAGLV : 1415
Oan.ENSOANG00000005124: RQLVCLARALLRKTKILVLDEATAAVDLETDDLIQSTIRTQFDDCTVLTIAHRLNTIMDYTRILVLDKGEVVECGSPSDLLQKKGIFYSMARDASLI : 1204
Bfl.232174: RQLVCLARALLRKSKILVLDEATAAVDLETDDLIQSTIRTQFADCTVLTIAHRLNTIMDSTRVLVLDAGSIAEFDSPQDLIASRGIFYRMAKDAGLA : 1416
Bfl.118638: RQLVCLARALLRKSKILVLDEATAAVDLETDDLIQSTIRTQFADCTVLTIAHRLNTIMDCLRVLVLDAGRIAEFDSPQDLIASRGIFYRMAKDAGLA : 1279
Bfl.118636: RQLVCLARALLRKSKILVLDEATAAVDLETDDLIQSTIRTQFADCTVLTIAHRLNTIMDSTRVLVLDAGRIAEFDSPQDLIASRGIFYGMAKDAGLA : 1403
Bfl.128060: RQLVCLARALLRKSKILVLDEATAAVDLETDDLIQSTIRTQFADCTVLTIAHRLNTIMDSTRVLVLDAGRIAEFDSPQDLIASRGIFYGMAKDAGLA : 1403
Dre.ENSDARG00000096662: RQLVCLARALLRKTRILVLDEATAAVDLETDDLIQSTIRTEFQDCTVFTIAHRLNTIMDYTRVLVLDKGQIAEFDTPTNLMNQKGLFFGMAKDAGLA : 1413
Ame.ENSAMXG00000016253: RQLICLARALLRKTRILILDEATAAIDLETDDLIQSTIRTQFEDCTVFTIAHRLNTIMDYTRILVLDKGQIAEFDTPVNLIAQKGIFYSMAKDAGLA : 1416
Ola.ENSORLG00000020741: RQLVCLARALLRKTRILILDEATAAIDLETDDLIQSTIRTQFENSTVFTIAHRLNTIMDYTRVLVLDKGKIAEFDTPTNLISKRGIFYGMAKDAGLV : 1413
Gmo.ENSGMOG00000010029: RQLVCLARALLRKTRILILDEATAAIDLETDDLIQSTIRTQFEDCTVFTIAHRLNTIMDYTRVLVLDKGEISEFDTPSQLLSQKGVFYAMAKDAGLV : 1364
Xma.ENSXMAG00000012203: RQLVCLARALLRKTRILVLDEATAAIDLETDDLIQSTIRTQFEDCTVFTIAHRLNTIMDYTRVLVLDKGQIAEFDTPTNLISKRGIFYGMAKDAGLV : 1415
Gac.ENSGACG00000005901: RQLVCLARALLRKTRILILDEATAAVDLETDDLIQSTIRTQFEDCTVFTIAHRLNTIMDYTRVLVLDKGQIAEFDTPANLISQRGIFYGMTKDAGLT : 1409
Oni.ENSONIG00000019586: RQLVCLARALLRKTRILILDEATAAIDLETDDLIQSTIRTQFEDCTVFTIAHRLNTIMDYTRVLVLDKGRIAEFDTPTNLISKRGIFYGMAKDAGLA : 1412
Dla.DLAgn_00098120: RQLVCLARALLRKTRILILDEATAAIDLETDDLIQSTIRTQFEDCTVFTIAHRLNTIMDYTRVLVLDKGQIAEFDTPTNLLSQRGIFYGMAKDAGLT : 1417
Cmi.SINCAMG00000013336: RQLVCLARALLRKTRILVLDEATAAVDLETDDLIQSTIRTQFKDSTVLTIAHRLNTIMDYTRVLVLDKGRIAEFDTPANLIAQRGVFYSMAKDAGLA : 1317
Ler.ctg12190: ------------------------------------------------------------------------------------------------- : -
Sca.ctg14163: RQLVCLARALLRKTRILILDEATAAVDLETDDLIQSTIRTQFDDCTVLTIAHRLNTIMDYTRVLVLEKGRIAEFDTPSNLIAQKGIFYSMVKDAGLA : 1196
Rty.XP_020375725_1: RQLVCLARALLRKTRILILDEATAAVDLETDDLIQSTIRTQFENCTVLTIAHRLNTIMDYTRVLVLERGRIAEFDTPSNLIAQKGIFYSMAKDAGLV : 836
Sac_EU250283: RQLVCLARALLRKTRILILDEATAAVDLETDDLIQSTIRTQFEGCTVLTIAHRLNTIMDYTRVLVLDKGSIAEFDTPSNLITQKGIFYSMAKDAGLA : 1416
Tni.ENSTNIG00000004171: RQLVCLARALLRKTRILILDEATAAIDLETDDLIQSTIRTQFEDCTVFTIAHRLNTIMDYTRVLVLDKGQIAEFDTPTNLIAQKGIFYGMAKDAGLT : 1365
Aca.ENSACAG00000001396: RQLVCLARALLRKTRILVLDEATAAIDLETDDLIQMTIRTQFEDCTVLTIAHRLNTIMDYTRVLVLDKGAIAEFDTPSRLIESKGIFYGMAKDAGLA : 1411
Gga.ENSGALG00000007522: RQLVCLARALLRKTRVLILDEATAAIDLETDDLIQMTIRTQFEDCTVLTIAHRLNTIMDYTRILVLDNGTIAEFDTPANLIASKGIFYGMAKDAGLV : 1362
Xtr.ENSXETG00000012239: RQLVCLARALLRKTRILILDEATAAIDLETDDLIQMTIRTQFEDCTVLTIAHRLNTIMDYTRVLVLDKGRIAEFDTPTNLIALKGIFYGMAKDAGLA : 1414
Loc.ENSLOCG00000010918: RQLVCLARALLRKTRILILDEATAAIDLETDDLIQSTIRTQFEDSTVFTIAHRLNTIMDYTRILVLDKGQIAEFDTTSNLLAMKGIFYGMAKDAGLA : 1414
Lch.ENSLACG7209/6619: RQLVCLARALLRKTKILVLDEATAAVDLETDDLIQSTIRTQFEDCTVLTIAHRLNTIMDYTRVLVLDKGEIAEFDTPTNLINKKGIFYGMVKDAGLA : 1397
Mdo.ENSMODG00000020910: ------------------------------------------------------------------------------------------------- : -
Dno.ENSDNOG00000046300: RQLVCLARALLHKSRVLILDEATAAIDLETDDLIQATIRTQFETRTVLTIAHRLNTIMDYTRVLVLDKGTVAEFDSPSNLIAARGIFYRMARDAGLA : 1408
Mmu.ENSMUSG00000020865: RQLVCLARALLRKSRVLVLDEATAAIDLETDDLIQGTIRTQFEDCTVLTIAHRLNTIMDYNRVLVLDKGVVAEFDSPVNLIAAGGIFYGMAKDAGLA : 1414
Ggo.ENSGGOP00000003354: RQLVCLARALLRKSRILVLDEATAAIDLETDNLIQATIRTQFDTCTVLTIAHRLNTIMDYTRVLVLDKGVVAEFDSPANLIAARGIFYGMARDAGLA : 1417
Hsa.ENSG00000108846: RQLVCLARALLRKSRILVLDEATAAIDLETDNLIQATIRTQFDTCTVLTIAHRLNTIMDYTRVLVLDKGVVAEFDSPANLIAARGIFYGMARDAGLA : 1417
Ptr.ENSPTRG00000009406: ------------------------------------------------------------------------------------------------- : -
Cluf.ENSCAFG00000017201: RQLVCLARALLRKSRILVLDEATAAIDLETDDFIQATIRTQFESCTVLTIAHRLNTIMDYTRVLVLDKGMIAEFDSPANLIAARGIFYGMARDAGLA : 1417
Bta.ENSBTAG00000020070: RQLVCLARALLRKSRILVLDEATAAIDLETDDLIQATIRTQFETCTVLTIAHRLNTIMDYTRVLVLDKGTIAEFDSPTNLIAARGIFYGMARDAGLA : 1417
